# Dietary fibre intake in the adult Swiss population: a comprehensive analysis of timing and sources

**DOI:** 10.1017/jns.2025.6

**Published:** 2025-03-24

**Authors:** Flurina von Blumenthal, Katja A. Schönenberger, Valentina V. Huwiler, Zeno Stanga, Giulia Pestoni, David Faeh

**Affiliations:** 1 Department of Diabetes, Endocrinology, Nutritional Medicine and Metabolism (UDEM), Inselspital Bern, University Hospital, Bern, Switzerland; 2 Division of Clinical Pharmacy and Epidemiology, Department of Pharmaceutical Sciences, University of Basel, Basel, Switzerland; 3 Nutrition Group, Swiss Distance University of Applied Sciences (FFHS)/University of Applied Sciences and Arts of Southern Switzerland (SUPSI), Zurich, Switzerland; 4 Division of Chronic Disease Epidemiology, Epidemiology, Biostatistics and Prevention Institute, University of Zurich, Zurich, Switzerland; 5 Health Department, Bern University of Applied Sciences, Bern, Switzerland

**Keywords:** Dietary fibre, Nutrition survey, Whole grains, 24h dietary recall, 24 HDRs, 24-hour dietary recalls, US, United States (of America), STROBE-nut, Strengthening the Reporting of Observational Studies in Epidemiology-Nutritional Epidemiology, BMI, body mass index, DACH, Germany (D), Austria (A), Switzerland (CH), AI, adequate intake

## Abstract

Recommended dietary fibre consumption is rarely met in high-income countries. Detailed analysis of fibre consumption patterns is essential to identify strategies for increasing intake. This study investigated the timing and sources of fibre intake in Switzerland, using data from the Swiss Nutrition Survey, *menuCH* (n = 2057, 18–75 years). Dietary characteristics were summarised for the adult population and for subgroups stratified by absolute (< 15 g/day, 15-<30 g/day, and >=30 g/day) and relative (< 10 g/1000 kcal/day, 10-<14 g/1000 kcal/day, and >=14 g/1000 kcal/day) fibre intake. Mean fibre intake of both 24 HDRs for each individual and contribution of food groups and timing (before breakfast, breakfast, during the morning, lunch, during the afternoon, dinner, after dinner/at night) was calculated. Fibre was mainly consumed at breakfast (4.1 g/day), lunch (6.0 g/day), and dinner (6.4 g/day). Intake at breakfast differed between the lowest and highest fibre intake groups by 6.4 g/day (absolute) and 4.3 g/day (relative). Breakfast skipping was more frequent among low-fibre intake groups (29% for absolute intake, 19% for relative intake) than in the overall population (15%). The main sources of dietary fibre were grain products (35.6%), followed by vegetables (18.3%) and fruits (18.2%), with whole grains accounting for 17.5% of grain intake. Legumes contributed only to 1% of total fibre intake. Public health efforts encouraging regular breakfast consumption, and intake of whole grains and legumes are recommended to improve fibre intake.

## Introduction

Dietary fibres are a heterogeneous group of carbohydrates mostly derived from plant cell walls and starch granules that resist digestion in the human intestine and confer various health benefits.^(1,2)^ Increased fibre consumption helps alleviate constipation,^(3)^ lowers blood pressure,^(4)^ improves glycaemic control in patients with type 2 diabetes^(5)^ and lowers blood cholesterol levels.^(4,6)^ Large prospective cohort studies have linked high fibre intake with a lower risk of cardiovascular disease,^(7,8)^ coronary heart disease,^(7)^ type 2 diabetes,^(8,9)^ and certain types of cancer.^(8,10–12)^

Recommendations for dietary fibre intake for adults in Europe and the US range between 30-35 g/day for men and 25-32 g/day for women.^(1)^ In the US, adequate dietary fibre intake for adults is defined as 14 g/1000 kcal/day.^(13)^ Daily dietary fibre consumption of 30 g is rarely achieved among adults in high-income countries.^(1)^ In Europe, average dietary fibre intake ranges from 18-24 g/day in adult men and 16-20 g/day in adult women.^(1)^ It is estimated that the current dietary fibre intake in the Western world is approximately one-third below the recommended amount.^(14)^ Correspondingly, 87% of Swiss adults do not meet the recommended daily intake of 30 g/day.^(15)^

Public health interventions are urgently needed to promote adequate fibre intake.^(8,10,16)^ The European Commission has implemented policies targeting insufficient dietary fibre intake in the general population. These include setting nutritional standards in public facilities, such as canteens in schools or workplaces, and releasing regulations regarding labelling of pre-packed foods.^(17)^

A detailed characterisation of dietary fibre consumption is essential to identify potential strategies for increasing intake. This study aimed to assess the main sources of dietary fibre intake and the timing of consumption during the course of the day in the adult Swiss population, using data from the National Nutrition Survey *menuCH*. We aimed to compare the timing of fibre intake between groups that meet and do not meet the recommended daily intake to understand how specific eating patterns may influence fibre consumption. We identified that grains serve as a primary source of dietary fibre. Since whole grains are particularly rich in dietary fibres^(1)^ and given the limited research on whole grain consumption in Switzerland, we extended our focus to encompass whole grain consumption.

## Methods

This report is in agreement with the Strengthening the Reporting of Observational Studies in Epidemiology-Nutritional Epidemiology (STROBE-nut).^(18)^

### Design and Sampling

The cross-sectional population-based Swiss National Nutrition Survey *menuCH* was conducted by The Institutes of Social and Preventive Medicine in Lausanne (IUMSP) and Bern (ISPM) on behalf of Swiss Federal Food Safety and Veterinary Office and Federal Office of Public Health, which funded the survey.

We used data from *menuCH* survey for the analyses. Before the implementation of *menuCH*, a pilot survey conducted from June to November 2013 tested recruitment, participation, and data management.^(19)^ The *menuCH* survey was conducted between January 2014 and February 2015 among Swiss residents aged 18-75 years.^(20)^ The stratified random sample composed by the Federal Statistical Office aimed to represent 35 strata: seven major regions of Switzerland (Lake Geneva region, Espace Mittelland, Northwestern Switzerland, Zurich, Eastern Switzerland, Central Switzerland, and Ticino) and five age groups (18-29, 30-39, 40-49, 50-64, and 65-75 years old).^(20)^ In total, 13,606 Swiss residents received invitations to participate in the study via a post-mailed letter with a response card. A 38% net response rate (2085 responders/5496 net sample) was achieved.^(21)^ After excluding non-eligible individuals and non-responders, data from 2,057 participants with two completed 24-hour dietary recall (24HDR) assessments were included in the analyses. Detailed sampling and recruitment processes, including information on participation rates and refusals, have been published previously.^(20)^

The *menuCH* survey was approved by the appropriate ethics committees (lead committee in Lausanne, Protocol 26/13, approved on February 12, 2013) and was conducted according to the Declaration of Helsinki. All participants provided a written informed consent. The survey was registered in the International Standard Randomised Controlled Trial Number (ISRCTN) registry.^(22)^

### Socio-demographic, lifestyle and anthropometric characteristics

Information on socio-demographic characteristics, dietary behaviour, and physical activity was collected using a 49-item questionnaire.^(21)^ Participants completed the questionnaire at home prior to their face-to-face interview. Nationality was classified as Swiss, Swiss binational, and non-Swiss. Based on the home address, participants were classified into three language regions: German, French, and Italian. During face-to-face interviews, anthropometric characteristics were measured, and measured height and weight were used to calculate the body mass index (BMI). Self-reported height and weight were used if measurements were not possible. Measurements taken before pregnancy were used for pregnant and lactating women. The categorisation of BMI groups followed the World Health Organization classification (underweight: BMI < 18·5 kg/m^2^; normal weight: 18·5 kg/m^2^ ≤ BMI < 25·0 kg/m^2^; overweight: 25·0 kg/m^2^ ≤ BMI < 30·0 kg/m^2^; obese: BMI ≥ 30·0 kg/m^2(21)^).

### Dietary assessment

After six weeks of training and regular retraining during the survey, 15 field dietitians recorded food consumption using two non-consecutive 24HDRs. The first interview was conducted face-to-face, and the second was conducted by phone two to six weeks later. Both recalls were scattered across weekdays and seasons.^(20)^ Dietitians assessed food intake using the trilingual Swiss version (0.2014.02.27) of GloboDiet^®^ software (formerly EPIC-Soft®, International Agency for Research on Cancer, Lyon, France,^(23,24)^ adapted for Switzerland by the Federal Food Safety and Veterinary Office, Bern, Switzerland). Dietitians assisted interviewees in quantifying food consumption with an illustrated book containing a 119 series of graduated portion-size pictures,^(25)^ 12 pictures of common household measures, and a collection of approximately 60 real dishes available at each study centre.^(19)^ Information about the nutrient content of the assessed food intake was completed using FoodCASE software (Premotec GmbH, Winterthur, Switzerland), which matched foods, recipes, and ingredients from the GloboDiet^®^ software with the most suitable item from the Swiss Food Composition Database.^(26)^ The quality control concept of the survey and data cleaning process have been described elsewhere.^(19,20)^ Approximately 2% of food items lacked information on dietary fibre content. Several sources, including the Swiss Food Composition Database, manufacturer’s nutrition facts label, and the German Nutrient Database were used to complete missing data on dietary fibre content. Dietary fibre intake from fibre supplements has not been recorded.^(15)^

### Dietary fibre intake

We followed the classification system proposed by Schönenberger et al. to categorise participants according to their dietary fibre intake, with the difference that we decomposed recipes into their ingredients in this study, while Schönenberger et al. considered whole recipes as they also investigated the consumption of ultra-processed food.^(15)^ Participants were classified into low, medium, and high dietary fibre intake groups based on absolute daily dietary fibre intake (< 15 g/day, 15-<30 g/day, and >=30 g/day) and dietary fibre intake relative to energy intake (< 10 g/1000 kcal/day, 10-<14 g/1000 kcal/day, and >=14 g/1000 kcal/day). The reference value for absolute dietary fibre intake was based on the DACH guidelines (Germany, Austria, Switzerland), with a minimal dietary fibre intake of 30 g/day for normal laxation and cardio metabolic health.^(27)^ The reference value for relative dietary fibre intake was based on the U.S Food and Nutrition Board, which set the adequate intake (AI) for dietary fibre to 14 g/1000 kcal/day, followed by findings from observational studies, where an AI of 14 g/1000 kcal/day was associated with the lowest risk of coronary heart disease.^(28)^

### Timing of dietary fibre consumption

During 24HDRs, study participants were asked to report timing of food consumption chronologically, based on common food consumption occasions (i.e., meals or between meals food consumption occasions): ‘before breakfast’, ‘breakfast’, ‘during the morning’, ‘lunch’, ‘during the afternoon’, ‘dinner’, ‘after dinner/at night’. This standardisation enabled the entire coverage of food consumption during the day to provide cognitive memory for the interviewees and to detect possible omitted food consumption occasions.^(29,30)^ Skipping breakfast was defined as a mean energy intake < 100 kcal at the food consumption occasions ‘before breakfast’ and ‘breakfast’ of both 24HDRs as proposed by Chatelan et al.^(31)^

### Sources of dietary fibre and whole grain definition

The GloboDiet^®^ software classifies food items into 19 food groups. The category ‘Recipes’ was omitted since their ingredients were considered. We distinguished four additional food groups and analysed the dietary fibre sources from 22 food categories. ‘Meat, meat products, and meat substitutes’ were split into ‘meat, meat products’, and ‘meat substitutes’. ‘Milk, milk products, and milk substitutes’ were divided into ‘milk, milk products’ and ‘dairy substitutes’. Additionally, food items in the category ‘fruits, nuts, and seeds’ were grouped into ‘fruits’, and ‘nuts and seeds’. Finally, the food items in the category ‘Grain and grain products’ were divided into ‘Refined grains’ and ‘Whole grains’. A description of the food items included in each food category are presented in Supplementary Table 1. We used the whole grain definition of Mozzaffarian et al., which follows the classification of the American Heart Association Goals 2020, to identify whole grain consumption in the overall study population and subgroups.^(32)^ Cereal products were defined as whole grains if the ratio of total carbohydrate to fibre by weight was less than or equal to 10. The carbohydrate-to-fibre ratio of 10:1 is approximately equal to that of whole-wheat flour.^(33,34)^ We calculated the mean whole grain consumption in g/day of both 24 HDRs for each participant and classified participants in whole grain consumers (whole grain consumption > 0g/day) and those who did not consume whole grain products (whole grain consumption = 0g/day).

### Statistical analysis

This study is a secondary analysis of the *menuCH* survey.^(20)^ All statistical analyses were based on the calculated mean food, energy, and dietary fibre intake of the two 24HDRs for each individual. The mean dietary fibre intake for each participant was then further stratified by sources and timing of dietary fibre. Using classification of timing and sources of dietary fibre intake of each individual, mean dietary fibre intake for the overall study population as well as the absolute and relative dietary fibre intake groups were determined. We conducted a sensitivity analysis excluding pregnant and lactating women from the study sample.

The *menuCH* weighting strategy was implemented to represent the general population of Switzerland. The detailed documentation is available elsewhere.^(35)^ The mean values were weighted for age, sex, marital status, major area of Switzerland, nationality, and household size to adjust for sampling design and non-response. We further weighted our results for seasons and weekdays to consider the distribution of the two 24HDRs across seasons and weekdays.

We used R Version 4.2.1: A language and environment for statistical computing for statistical analysis. R Foundation for Statistical Computing, Vienna, Austria. URL https://www.R-project.org) using the following packages: tidyverse (version 2.0.0) for data sorting and data visualisation; gtsummary (version 1.7.0), survey (version 4.1-1), srvy (version 1.2.0), and robsurvey (version 0.5-2) for the weighted mean and data tables.

## Results

A total of 2,057 individuals with two completed 24HDRs were included in the analysis. Table 1 presents the socio-demographic, anthropometric, and lifestyle characteristics based on whole grain consumption. The study population included 50.2% females, with most participants being Swiss nationals (61.4%). The majority came from the German-speaking region (69.2%), followed by the French-speaking region (25.2%) and the Italian-speaking region (5.6%). Tertiary education was most common (52.7%), and 54.3% had a normal BMI. Most reported good to very good health (87.3%), with 43% never having smoked, 33.7% being former smokers, and 23.3% being current smokers. Overall, 64% of participants reported consuming whole grain products. Non-consumers of whole grains were more likely to be male, aged 18-29 years, from the Italian- and French-speaking regions, to have a primary or secondary education, to be overweight or obese, and current smokers.

**Table 1.** Socio-demographic, lifestyle, and anthropometric characteristics of the *menuCH* participants (n = 2,057)

Characteristic		Consumed whole grains, n (%)	
	Overall, n (%)	Yes	No
	(n = 2,057)	(n = 1,305)	(n = 752)
Sex			
Female	1124 (50.2)	764 (54.6)	360 (42.4)
Male	933 (49.8)	541(45.4)	392 (57.6)
Age group			
18-29 years	400 (18.8)	217 (16.4)	183 (23.0)
30-44 years	533 (29.9)	336 (29.0)	197 (31.4)
45-59 years	625 (29.8)	400 (30.6)	225 (28.4)
60-75 years	499 (21.5)	352 (24.1)	147 (17.1)
Nationality			
Swiss only	1492 (61.4)	959 (62.2)	533 (60.0)
Swiss binational	297 (13.8)	187 (14.0)	110 (13.3)
Non-Swiss	268 (24.8)	159 (23.8)	109 (26.6)
Language region			
German	1341 (69.2)	893 (72.3)	448 (63.8)
French	502 (25.2)	315 (23.9)	187 (27.6)
Italian	214 (5.6)	97 (3.8)	117 (8.7)
Highest Education			
Primary	89 (4.7)	46 (3.7)	43 (6.5)
Secondary	968 (42.6)	592 (40.1)	376 (47.1)
Tertiary	997 (52.7)	666 (56.2)	331 (46.4)
BMI^*^ groups			
Underweight	50 (2.4)	33 (2.5)	17 (2.2)
Normal weight	1096 (54.3)	738 (57.0)	358 (49.5)
Overweight	621 (30.7)	372 (29.2)	249 (33.3)
Obese	256 (12.6)	140 (11.3)	116 (14.9)
Currently on a diet			
Yes	113 (5.4)	72 (5.5)	41 (5.3)
Self-reported health status			
Very bad to Medium	272 (12.7)	137 (10.5)	135 (16.7)
Good to very good	1781 (87.3)	1167 (89.5)	614 (83.3)
Smoking status			
Current	451 (23.3)	243 (18.9)	208 (31.2)
Former	688 (33.7)	445 (34.8)	243 (31.8)
Never	914 (43.0)	616 (46.3)	298 (37.0)

* BMI, Body Mass Index.

Numbers (*n*) are unweighted. Percentages (%) are weighted for age group, sex, marital status, major region of Switzerland, nationality and household size following the *menuCH* weighting strategy^(35)^.

Table 2 illustrates mean dietary fibre consumption based on food consumption occasions for the overall *menuCH* population and for the absolute and relative dietary fibre intake groups. In the overall study population, dietary fibre was mainly consumed at breakfast (4.1 g/day), lunch (6.0 g/day) and dinner (6.4 g/day). Overall, and within subgroups, dietary fibre intake was lowest before breakfast and highest at dinner. Individuals in the lowest and highest fibre intake groups differed by 6.4 g (absolute intake) and 4.3 g (relative intake) in their dietary fibre intake at breakfast. In the medium and high dietary fibre intake groups, fibre intake was more evenly distributed throughout the day, with one meal contributing a minimum of 19.6% and a maximum of 30.8% (Supplementary Fig. 1). In the *menuCH* population, 308 of 2,057 individuals skipped breakfast (15%), with breakfast skipping being most prevalent among the low dietary fibre intake groups (29% for absolute intake and 19% for relative intake). In contrast, the prevalence of breakfast skipping was 10% in the medium group and 6% to 9% in the high absolute and relative dietary fibre intake groups, respectively.

**Table 2.** Mean dietary fibre intake on food consumption occasions in the *menuCH* population overall and by absolute and relative dietary fibre intake groups (n = 2,057)

Food consumption occasion	Dietary fibre intake [g/day], mean (SD)						
	Overall	Absolute dietary fibre intake			Relative dietary fibre intake		
		< 15 g/day	15-<30 g/day	>= 30 g/day	< 10 g/1000 kcal/day	10-<14 g/1000 kcal/day	>=14 g/1000 kcal/day
	(*n* = 2,057)	(*n* = 535)	(*n* = 1,257)	(*n* = 265)	(*n* = 1,206)	(*n* = 606)	(*n* = 245)
Total	20.7 (8.8)	11.5 (2.5)	20.9 (3.9)	37.1 (7.8)	17.1 (6.0)	24.1 (7.5)	31.5 (11.3)
Before breakfast	0.1 (0.5)	0.1 (0.2)	0.1 (0.6)	0.2 (0.5)	0.1 (0.4)	0.2 (0.7)	0.1 (0.5)
Breakfast	4.1 (3.6)	1.9 (1.7)	4.1 (2.9)	8.3 (5.0)	3.0 (2.6)	5.2 (3.6)	7.3 (5.0)
During the morning	1.2 (2.1)	0.6 (1.0)	1.2 (1.7)	2.8 (3.7)	0.9 (1.4)	1.5 (2.4)	2.0 (3.2)
Lunch	6.0 (3.6)	3.7 (2.0)	6.1 (3.0)	9.6 (5.0)	5.1 (2.9)	6.6 (3.6)	9.0 (4.8)
During the afternoon	1.9 (2.3)	1.1 (1.2)	2.0 (2.0)	3.5 (3.8)	1.6 (1.9)	2.3 (2.6)	2.8 (3.0)
Dinner	6.4 (4.0)	3.8 (1.9)	6.4 (3.1)	11.0 (5.7)	5.5 (3.2)	7.2 (4.0)	8.9 (5.8)
After dinner	1.0 (1.8)	0.4 (0.8)	1.1 (1.7)	1.8 (2.9)	0.9 (1.5)	1.1 (2.0)	1.3 (2.3)

Values are weighted for age group, sex, marital status, major region of Switzerland, nationality, household size, season, and weekdays following the *menuCH* weighting strategy^(35)^.

Table 3 presents the mean dietary fibre intake from food groups for the overall *menuCH* population and the absolute and relative dietary fibre intake groups. Grain and grain products were the primary sources of dietary fibre in the Swiss population (7.4 g/day), followed by vegetables (3.8 g/day) and fruits (3.8 g/day), with these proportions remaining consistent across subgroups. In the overall study population, 17.5% of the grain products consumed were whole grains, with percentages varying by intake group: 9% and 10% in the low, 15.7% and 25.4% in the medium, and 31.8% and 38.4% in the high absolute and relative dietary fibre intake groups, respectively.

**Table 3.** Mean dietary fibre intake from food categories in the *menuCH* population overall and by absolute and relative dietary fibre intake groups (n = 2,057)

Food category^*^	Dietary fibre intake from food category [g/day], mean (SD)							Standard fibre content of food category based on Swiss Food Composition Database [g/100g], mean^†^
	Overall (*n* = 2,057)	Absolute dietary fibre intake			Relative dietary fibre intake			
		< 15 g/day (*n* = 535)	15-<30 g/day (*n* = 1,257)	>= 30 g/day (*n* = 265)	< 10 g/1000 kcal/day (*n* = 1,206)	10-<14 g/1000 kcal/day (*n* = 606)	>= 14 g/1000 kcal/day (*n* = 245)	
Refined grains	4.4 (3.3)	3.1 (2.0)	4.7 (3.2)	5.4 (4.9)	4.8 (3.3)	4.0 (3.1)	3.2 (3.4)	5.5
Whole grains	3.0 (4.0)	1.0 (1.5)	2.8 (3.0)	7.6 (6.4)	1.7 (2.5)	4.4 (4.3)	6.1 (6.0)	15.4
Fruits	3.8 (3.9)	1.5 (1.7)	3.7 (3.2)	8.2 (5.5)	2.2 (2.3)	5.3 (3.8)	8.1 (5.3)	4.4
Vegetables	3.8 (2.9)	2.3 (1.7)	3.9 (2.5)	6.2 (4.0)	3.0 (2.1)	4.5 (2.8)	6.4 (4.2)	3.3
Potatoes, other starchy root tuber	1.1 (1.4)	0.7 (1.1)	1.2 (1.5)	1.3 (1.8)	1.1 (1.4)	1.1 (1.5)	0.9 (1.5)	2.3
Cakes, cookies, patisserie	0.8 (1.2)	0.6 (0.8)	0.9 (1.2)	1.0 (1.6)	0.9 (1.3)	0.7 (1.1)	0.6 (0.9)	2.7
Sugar, chocolate, sweets	0.8 (1.0)	0.4 (0.6)	0.8 (1.0)	1.1 (1.1)	0.8 (1.0)	0.7 (1.0)	0.7 (0.8)	2.9
Nuts, seeds	0.6 (1.6)	0.2 (0.5)	0.5 (1.1)	1.6 (3.3)	0.3 (0.7)	0.7 (1.4)	1.7 (3.5)	10.4
Seasoning, spices, herbs, sauces	0.5 (0.7)	0.3 (0.4)	0.5 (0.6)	0.8 (1.3)	0.4 (0.6)	0.5 (0.9)	0.5 (0.9)	5.5
Non-alcoholic beverages	0.5 (1.0)	0.4 (0.4)	0.5 (0.7)	0.7 (2.2)	0.5 (0.6)	0.6 (1.3)	0.6 (1.9)	5.6
Salty snacks	0.4 (1.5)	0.2 (0.5)	0.3 (1.0)	0.9 (3.4)	0.4 (1.1)	0.3 (1.9)	0.6 (2.2)	6.0
Soups	0.3 (0.6)	0.2 (0.4)	0.3 (0.7)	0.3 (0.8)	0.2 (0.5)	0.3 (0.7)	0.4 (0.8)	1.2
Milk products	0.3 (0.7)	0.2 (0.4)	0.4 (0.8)	0.4 (0.9)	0.3 (0.6)	0.3 (0.8)	0.4 (1.0)	0.1
Legumes	0.2 (1.1)	0.1 (0.4)	0.2 (1.0)	0.6 (2.1)	0.1 (0.6)	0.3 (1.4)	0.7 (1.9)	11.5

Values are weighted for age group, sex, marital status, major region of Switzerland, nationality, household size, season, and weekdays following the *menuCH* weighting strategy^(35)^.

* Categories accounting for less than 0.2% of total dietary fibre intake were not shown: ‘alcoholic beverages’, ‘fats and oils’, ‘eggs and egg products’, ‘diary substitutes’, ‘meat substitutes’, ‘meat products’, ‘fish and seafood’, ‘various foods’

† Values were obtained from the Swiss Food Composition Database.^(26)^

**Fig. 1.**
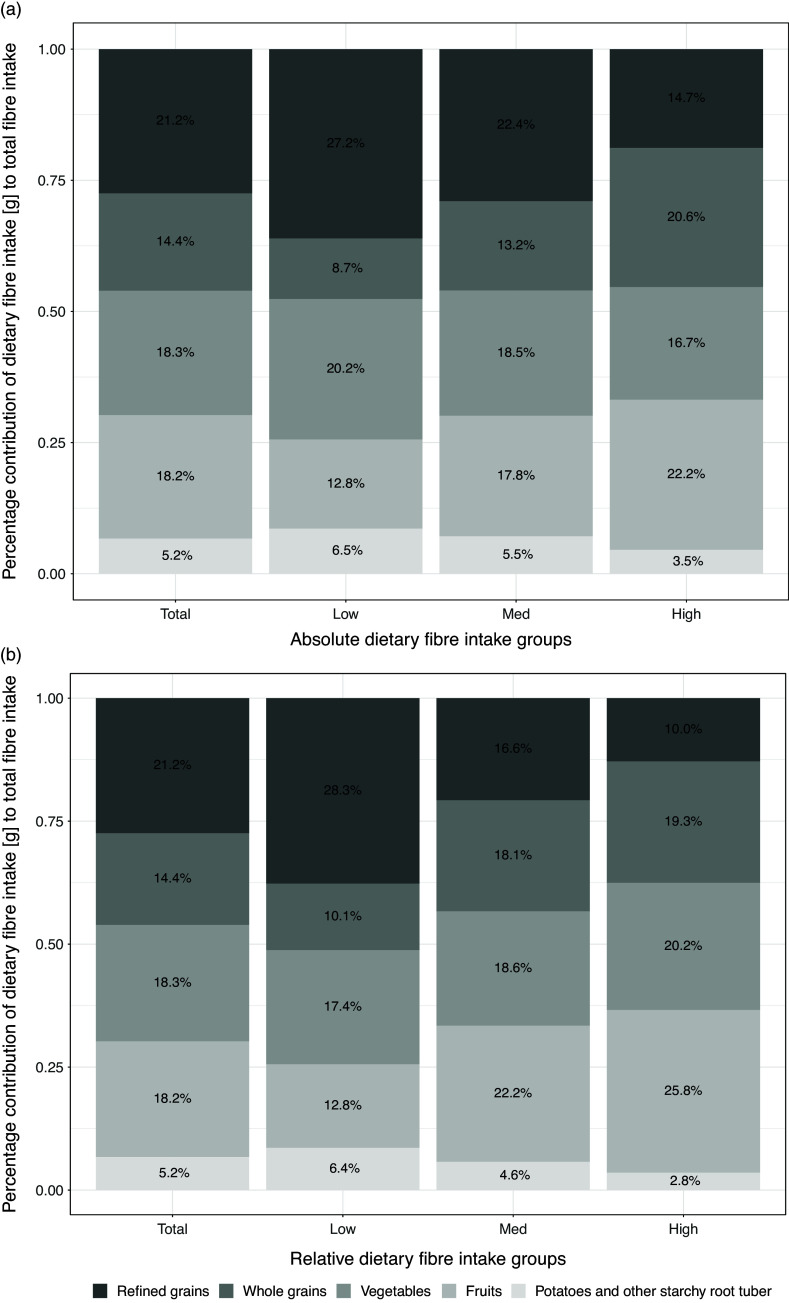
Dietary fibre intake from food categories for the overall study population for the absolute (a) and relative (b) dietary fibre intake groups. The bar plot represents the contribution of food categories, shown as a percentage (%) of the total dietary fibre intake. Total dietary fibre intake refers to the mean dietary fibre intake of both 24 HDRs displayed in Table 2. Food categories contributing to less than 5 % of the total amount were not displayed.

Figure 1 depicts the five main sources of dietary fibre for the overall population and the absolute and relative dietary fibre intake groups: refined grains, whole grains, vegetables, fruits, potatoes and other starchy root tuber. In the total population, refined grains contributed 21.2% of dietary fibre, while whole grains accounted for 14.4%. The intake of dietary fibre from whole grains differed by 11.9% and 9.2% between low and high-fibre intake groups for absolute and relative intake, respectively. Mean intake of whole grains differed by 92 g/day and 60 g/day between low and high dietary fibre groups for absolute and relative intake (Supplementary Table 2). Fruits contributed 18.2% of total daily dietary fibre, with differences in intake of 9% and 13% between low and high dietary fibre groups for absolute and relative intake, respectively (Fig. 1). Mean fruit intake varied by 277 g/day and 234 g/day between the low and high dietary fibre groups for absolute and relative intake (Supplementary Table 2).

The sensitivity analysis excluding pregnant and lactating women from the study population did not yield different results (see Supplementary Table 3, Supplementary Table 4 and Supplementary Table 5).

## Discussion

This cross-sectional, population-based representative study using two 24HDRs showed that fibre intake was highest during the three main meals: breakfast, lunch, and dinner. Breakfast skipping was more common among low-fibre consumers than the overall study population, suggesting a link between breakfast skipping and low daily fibre intake. Grains were the largest source of dietary fibre, followed by vegetables and fruits.

In the present study, dietary fibre intake was the highest at dinner, followed by lunch and breakfast. Low dietary fibre consumers tended to cover less of their daily fibre intake at breakfast than the other groups and the overall study population. This may be partly due to skipping breakfast more frequently than in the overall study population (29% low absolute and 19% low relative intake groups compared to 15% in the overall study population). Our findings suggest that skipping breakfast may be linked to a lack of daily dietary fibre intake. To date, no comparable studies on the contribution of different meals to the total dietary fibre consumption are available.

Grain and grain products were the main sources of dietary fibres in the Swiss population. Grains provided 35.6% of total fibre intake, aligning with findings from other European countries and Australia.^(1,36)^ Whole grains, however, contributed only to 17.5% of total grain intake, likely due to inconsistent labelling standards that make it difficult for consumers to identify whole grain foods.^(37–39)^ Global labelling standards and indicators like the 10:1 carbohydrate-to-fibre ratio could help consumers select healthier carbohydrate-rich foods.^(32,40–42)^ Low-fibre groups obtained a greater portion of their fibre intake from refined grains than the overall study population (27.2% and 28.3% for absolute and relative intake groups, respectively), highlighting a need to promote whole grains over refined grains.

Vegetables (18.3%), fruits (18.2%), and potatoes (5.2%) were other primary sources of dietary fibre, a pattern similar to that seen in Europe and the U.S.^(1)^ This variation reflects climatic differences across Europe, with southern countries covering more dietary fibre from fruits and northern countries covering more from potatoes.^(1)^ Interestingly, Switzerland, with its central European location, follows a more southern-oriented diet, with fruits contributing to a greater amount of total dietary fibre consumption than potatoes. High-fibre consumers in our study ate three times more fruit than low-fibre consumers (Supplementary Table 2), mirroring findings from Australia that link higher fibre intake with greater fruit and whole grain consumption.^(36)^ Unlike in Australia, we found no significant differences in fibre intake from vegetables across groups.^(36)^

Legumes contributed only to 1% of the total dietary fibre intake (0.2 g/day). Stephen et al. report that legume fibre intake varies in Europe from 0.8% in Belgium to 12% in Spain,^(1)^ and Hughes et al. note that Europe has the lowest legume consumption globally, with all investigated countries below the recommended 50 g/day.^(43,44)^ Industrialisation may have reduced legume consumption in western countries, where they’ve been replaced by animal proteins and refined foods.^(45)^ This trend may extend to Switzerland, with an average legume intake of 9.4 g/day and diets rich in red and processed meats.^(46)^ Legumes, with 11.5 g of fibre per 100 g, present a valuable opportunity to increase fibre intake, supporting both human health and environmental sustainability.^(47)^

### Strength and Limitations

Our study provides information on dietary fibre sources and the timing of their consumption. Additionally, whole grains as an important source of dietary fibre were more extensively studied. The *menuCH* survey is based on a stratified random sample that allows the representation of the Swiss population. Statistical weighting accounted for the non-response and uneven distribution of 24HDRs across seasons and weekdays.

Limitations include the possible effect of participation bias since people with a generally healthy lifestyle might have responded to study invitations more frequently. If participation bias occurred, we would expect the overall dietary fibre intake to be overestimated in our study. Although standardised and quality-approved dietary assessments were used, recall bias as well as over- and under-reporting may have influenced the data on dietary fibre intake timing and sources. Although the 10:1 carbohydrate-to-fibre ratio performed well in estimating the quality of the grain and grain products, it does not fully reflect their health benefits. For instance, simply from labels on food products, naturally occurring fibre and added fibre cannot be distinguished, which may have fewer beneficial health effects.^(32)^ Further we did not include intake of dietary supplements. Lastly, we examined the data descriptively without performing any statistical testing.

### Conclusion

Dietary fibre intake in Swiss adults was highest at breakfast, lunch, and dinner, with low-fibre consumers more likely to skip breakfast. Grain products, fruits, and vegetables were the primary sources of dietary fibres. Subgroups differed in their dietary fibre intake stemming from grain products and fruits. Generally, whole grain and legume consumption were low in the adult Swiss population. Given the beneficial effects of high fibre intake on health, public health initiatives encouraging the consumption of dietary fibres, especially promoting the intake of whole grains over refined grains as well as a higher intake of legumes, are needed.

## Supporting information

von Blumenthal et al. supplementary material 1 von Blumenthal et al. supplementary material

von Blumenthal et al. supplementary material 2 von Blumenthal et al. supplementary material

## Supplementary material

The supplementary material for this article can be found at https://doi.org/10.1017/jns.2025.6
