# Supplementary material for: Dietary fibre intake in the adult Swiss population: a comprehensive analysis of timing and sources
Source: J Nutr Sci. 2025 Mar 24;14:e27. doi: 10.1017/jns.2025.6 (PMC11950702; doi:10.1017/jns.2025.6)
Supplement: von Blumenthal et al. supplementary material 1 — von Blumenthal et al. supplementary material [file S2048679025000060sup001.zip › AEE style sheet_24-07 (1) (1).docx]

Style sheet – Australian Journal of Environmental Education (AEE)

Last updated March 24, 2023

# Level of edit

Level I

# Standard queries

*Please use these standard queries as applicable*

- N/a

# Article elements

## Article template

- Cambridge Standard Template Small

## Article types

- Article
- Editorial
- Communication
- Review – Book
- Review – Other
- Report Synopsis
- Thesis Synopsis
- Obituary

## Article types – special formatting

- Reviews have no abstract or keywords
- Thesis and Report synopses have no abstracts

## Special issue/symposium/collection headings

- N/A

## Article title

- Capitalization style: Title case – Updated on October 17
- Subtitle: Run in and separated from the title by a colon
- Footnote not allowed against article title

*Examples*

The Effectiveness of a Community-Based Playgroup in Inspiring Positive Changes in the Environmental Attitudes and Behaviours of Children and their Parents: A Qualitative Case Study

## Author name(s)

Full first name and last name, plus any other initials

- Initials, where used, are followed by a period. No space between initials.
- No serial comma
- “and” before last author name
- No academic degrees or accreditations in byline

#### Examples

- Single author: John Smith
- Two authors: John Smith and Jane R. Smith
- Multiple authors: John Smith, Jane R. Smith and Jeremy K. L. Smith

## ORCIDs

- Required for corresponding authors, optional for all other authors. If missing raise query at first proof.
  - Std. Query: If you would like to list the ORCID for the authors "XXXX", please provide them.
- Placement of ORCID icon: Next to author name in byline

## Author affiliations

- Department/school, university/institution, city, state/province, country, email
- Abbreviate USA and UK
- Use two-letter postal codes for state/province names
- If an author has multiple affiliations or if there are multiple authors, use superscript Arabic numerals to differentiate affiliations.
- Affiliations are run in, separated by semicolons. There is no semicolon preceding the “and” before the last affiliation.
- Country required, query if missing
- States should be abbreviate for USA, Canada, Australia
- Preferably no street addresses or zip codes.
- If all authors have the same affiliation, no superscript numbering is necessary.

#### Examples

- Timothy Callaghan^1^ and Andrew Karch^2^
  - ^1^Texas A&M University, College Station, TX, USA and ^2^University of Minnesota, Minneapolis, MN, USA
- Jenalee R. Doom^1^, Kenia M. Rivera^1^, Estela Blanco^2,3^, Raquel Burrows^4^, Paulina Correa-Burrows^4^, Patricia L. East^3^, Betsy Lozoff^5^ and Sheila Gahagan^3^
  - ^1^Department of Psychology, University of Denver, Denver, CO, USA; ^2^Department of Psychology, St. John’s University, New York, NY, USA; ^3^Department of Psychology, University of Michigan, Ann Arbor, MI, USA; ^4^Serious Mental Illness Treatment Resource and Evaluation Center, Office of Mental Health and Suicide Prevention, Department of Veterans Affairs, Ann Arbor, MI, USA and ^5^Survey Research Center of the Institute for Social Research, University of Michigan, Ann Arbor, MI, USA

## Corresponding author

- No asterisks allowed to signify the corresponding author. Only list the words “corresponding author” if there is more than one author. Styling now as shown:
- Single author - Email:
- Multiple authors - **Corresponding author:** Author; Email:

## Abstract

- No more than 250 words
- “Abstract” heading in bold
- Unstructured

## History dates

Required

**If required, specify format:**

Run in

(Received [date month year]; revised [date month year]; accepted [date month year])

#### Example

(Received 24 November 2019; revised 14 July 2020; accepted 17 August 2020)

## Keywords

- Authors should provide up to 5 keywords.
- One word (keywords not key words)
- Keywords must be in alphabetical order; Sentence case
- Keywords are separated by semicolons, with no end period
- Lowercase except for proper nouns

**Keywords**: Coronary artery disease; stroke; vascular risk factor

## MSC Codes (data/math)

- N/a

# Main body elements

## Right running head

- Journal title

## Left running head

- Single author: [First name] [Surname]
- Two authors: [First name] [Surname] and [First name] [Surname]
- Three or more authors: [Surname] et al.

## Headings

- unnumbered headings
- Heading levels allowed: H1 and H2 are permitted. H3 and above are not allowed.

**Heading 1**

Bold and in sentence case

***Heading 2***

Bold and italic and in sentence case

## Figures

- All figures must be called out in text, in sequential order.
- Any journal-specific rules for numbering style: 1a, 1b, 1c…

### Captions

- Sentence capitalization style
- Ending period
- Figure should be unabbreviated (i.e., Figure 1 not Fig. 1) in the caption
- Label style: **Figure 1.**

#### Examples

Figure 2. Student responses about (a) optimism towards solving environmental problems, (b) sense of stewardship towards the environment and (c) sense of agency towards addressing environmental problems.

### Credits

- Format: *Source*: Author-date citation.

### Figure notes

- Format: *Note* in italics and followed by a colon
- Note marker style for notes applying to specific parts of the figure (if applicable)? *,†,‡, §,∥,#
- Use “=” to define abbreviations: CI = confidence interval; HS&B = High School & Beyond; NELS88 = National Education Longitudinal Study of 1988; UK = United Kingdom.

#### Examples

*Indicates negatively worded questions.

## Tables

- All tables must be called out in text, in sequential order.
- Column headings and the rows of the stub column should be sentence case.

### Titles

- Sentence case
- Label style: “**Table [number]**” in bold; no full stop at the end of the title

### Credits

- Author-date citation style as a footnote

#### Examples

Source: Haribon Foundation for the Conservation of Natural Resources, Inc. (2006); Galang et al. (2003).

### Table notes

- Format: *Note* in italics and followed by a colon.
- Note marker style for notes applying to specific parts of the table? *Examples*: 1, 2, 3; a, b, c;*,†,‡, §,∥,#
- Order of elements? (1) source notes, (2) general table notes, (3) specific data notes, and (4) notes on significance levels

#### Examples

**Correlation is significant at 0.05 level (2-tailed).

## Footnotes/endnotes

Footnotes

## Extracts (block quotes)

- Omit quotation marks around quotations set as extracts.
- Set quotations of more than 50 words as extracts

## Lists

- Follow format in manuscript.

## Equations

- Follow format in manuscript.

# Back matter – No BM headings for 'book-review' and 'product-review'

- Sentence case
- Singular, not plural, EXCEPT for Acknowledgments and Competing interests.
- Ending with a period
- **Conflicts of interest** should be changed to **Competing interests**

1. Supplementary material
   - - **Supplementary material.** The supplementary material for this article can be found at [https://doi.org/](https://doi.org/10.1017/). . .
2. Acknowledgements (Mandatory heading)
3. Financial support (Mandatory heading)
4. Competing interests -- if the competing interest has a **negative statement**, it can be removed
5. Ethical standard (Mandatory heading): When statement is negative use “Nothing to note”.
6. References
7. Appendices
   - For more than one print appendix, Appendix A, Appendix B, etc.
     - In Appendix A: Table A1, Figure A1, etc.
     - In Appendix B: Table B1, Table B2, etc.
8. Author Biographies (Please add bio of each author under the heading “Author Biographies”). Please remove author designation like Dr, Prof., etc.

****NOTE: Thesis Synopses have a separate short section at the end before the other back matter containing full thesis citation and the supervisors.**

*Example*

Please find full thesis, Jukes, S. (2022). *Enacting more-than-human pedagogies in response to ecological precarity: An immanent praxiography* [Doctoral dissertation]. La Trobe University. https://doi.org/10.26181/20400702.v1

A thesis submitted for the award of Doctor of Philosophy in the School of Education at La Trobe University.

**Supervisor:**

**Dr Marcus Morse**

La Trobe University

## Cite this article

#### Follow journal reference style.

#### Examples

Mongar, K. (2023). The impacts of environmental science on Bhutanese students’ environmental sustainability competences. *Australian Journal of Environmental Education*, 1–15. https://doi.org/10.1017/aee.2023.2

# Reference style

## Standard Style Guide(s)

American Psychological Association (APA)

## Citations

In-text citations

### General style

#### Examples

(Deleuze & Guattari, 1987)

(St. Pierre *et al.*, 2023)

## References

### General style

#### Examples

# Style

## Spelling

- British-English spellings/Australian spellings (i.e. UK ise)

## Quotation marks

US style: “Double” quotation marks, with periods and commas inside marks and ‘single’ quotation marks for quotes within quotes.

## Dashes

Em dash, one letter space on each side

## Order of parentheses in text

([ ])

## Serial comma

no

## Abbreviations and acronyms

## Capitalization

## Dates, time, eras

## Hyphenation

## Measurements and units

## Numbers

## Punctuation

## Possessives

## Translations and Transliterations

## Other society- or journal-specific notes

## Word list, spelling preferences, common acronyms

| a | b | c |
| --- | --- | --- |
| d | e | f |
| g | h | i |
| j | k | l |
| m | n | o |
| p | q | r |
| s | t | u |
| v | w | x y z |
