# Supplementary material for: Dietary fibre intake in the adult Swiss population: a comprehensive analysis of timing and sources
Source: J Nutr Sci. 2025 Mar 24;14:e27. doi: 10.1017/jns.2025.6 (PMC11950702; doi:10.1017/jns.2025.6)
Supplement: von Blumenthal et al. supplementary material 1 — von Blumenthal et al. supplementary material [file S2048679025000060sup001.zip › AEE-2200038_ONLINE-Thesis Synopsis (1).pdf]

Australian Journal of Environmental Education (2022), 1–3
doi:10.1017/aee.2022.38

CAMBRIDGE UNIVERSITY PRESS

THESIS SYNOPSIS

Enacting more-than-human pedagogies in response to ecological precarity: an immanent praxiography

Scott Jukes

Institute of Education, Arts and Community, Federation University Australia, Berwick, Australia
E-mail: s.jukes@federation.edu.au

(Received 08 July 2022; revised 26 July 2022; accepted 28 July 2022)

Keywords: outdoor environmental education; confronting environmental problems; place-responsive pedagogies; posthumanism in education; new materialisms

Synopsis

Our current epoch is a time of mass extinction, changing climates, socio-ecological inequality and widespread precarity — these are troubling times. Scholars such as Haraway (2016) highlight that drivers of our ecological predicament include the problematic cultural ideas of anthropocentrism, bounded individualism and human exceptionalism. Such problematic ideas and the precarious conditions of the present are what compelled me, an outdoor environmental educator, to explore and confront these issues in the places I work. Thus, this doctoral inquiry was performed from a situated perspective where I asked: How might I, an outdoor environmental educator, address these precarious times in my field and through my practice?

The situated approach to the inquiry was a purposeful strategy. The mess of ecological precarity can be described in broad brush stroke statements; however, my approach to confronting such issues required delving into the nuanced complexity of problems in specific ways, places and times. This approach, influenced by the place-responsive turn in outdoor environmental education (OEE) (e.g., Mannion & Lynch, 2016; Stewart, 2020; Wattchow & Brown, 2011), led me to eschew universal fixes or final solutions, but instead undertake a series of place and context-responsive experiments in practice. The guiding heuristic for these experiments was the notion of more-than-human pedagogies.

The notion of more-than-human pedagogies was provoked by posthumanist (e.g., Braidotti, 2013), new materialist (e.g., Barad, 2007; St. Pierre, Jackson, & Mazzei, 2016) and Deleuzo-Guattarian (Deleuze & Guattari, 1987) theories. In keeping with such theories, I embraced multiplicity and attempted a pluralistic enactment of more-than-human pedagogical experimentation. I attempted to put such ideas to work by challenging human exceptionalism, individualism and hierarchical anthropocentrism in my everyday practice as an educator. I published the work as I went resulting in a thesis by publication. Through this process, the various chapters emerged in different conditions, with different foci and in relation to different concepts and theories.

The context for the empirical elements of the inquiry included bushwalking and ski-touring journeys in the Australian Alps, remake activities on university campuses and canoe journeys on various rivers in the Murray–Darling basin. Furthermore, other chapters explored more personal elements of my (and others) thinking and life. Through my practice in these contexts, I produced a varied assortment of empirical materials, which I drew upon, analysed and/or put to work in different ways. Empirical materials included a picture-story book created by a student, field-based photographic images and videos, research journal entries, student essays, creative writing and artefact making.

© The Author(s), 2022. Published by Cambridge University Press. This is an Open Access article, distributed under the terms of the Creative Commons Attribution licence (https://creativecommons.org/licenses/by/4.0/), which permits unrestricted re-use, distribution, and reproduction in any medium, provided the original work is properly cited.

Overall, through these empirical materials, I attempted to avoid normative approaches to conventional humanist qualitative inquiry (St. Pierre, 2011). In doing so, the various chapters, and empirical materials utilised within, explore experiments for engaging with and/or evoking more-than-human worlds.

Each of my published articles from this project were weaved into the thesis, along with other unpublished work, to create the research narrative. To help bring the thesis by publication together, I developed my own take on an emergent methodology, which I called immanent praxiography. Mol (2002) first coined the term praxiography, in an exploration into practices (*praxis*) rather than culture (*ethno*) alone. I deployed praxiography (rather than ethnography or auto-ethnography) as a term to help describe the non-anthropocentric focus on ontological practices and processes. In addition, I described the praxiography as immanent, as it was within and through practices, without a teleological goal, fixed set of methods or pre-conceived image of the final product, that I undertook the project.

In summary, the thesis provides examples of putting post-humanist and new materialist theoretical ideas to work in empirical settings. The two main conceptual offerings from this work include the notion of more-than-human pedagogies for OEE and the emergent methodological idea of immanent praxiography as an approach to educational research.

Please find full thesis, Jukes, S. (2022). *Enacting more-than-human pedagogies in response to ecological precarity: An immanent praxiography* [Doctoral dissertation]. La Trobe University. https://doi.org/10.26181/20400702.v1

A thesis submitted for the award of Doctor of Philosophy in the School of Education at La Trobe University.

Supervisors:

Dr Marcus Morse
La Trobe University

Professor Noel Gough
La Trobe University
(Initial supervision also by **Dr Alistair Stewart**)

Acknowledgements. Thank you to my supervisers, my wife Tiffany and everyone else who helped me along the way.

Conflicts of Interest. None.

Financial Support. This research was supported by an Australian Government Research Training Scheme Scholarship.

Ethical Standards. Research ethics approval was given by the La Trobe University Human Ethics Committee.

References

Barad, K. (2007). *Meeting the universe halfway: Quantum physics and the entanglement of matter and meaning.* Durham, UK: Duke University Press.

Braidotti, R. (2013). *The posthuman.* Cambridge, UK: Polity Press.

Deleuze, G., & Guattari, F. (1987). *A thousand plateaus: Capitalism and schizophrenia* (B. Massumi, Trans.). London, UK: Bloomsbury.

Haraway, D. (2016). *Staying with the trouble: Making kin in the Chthulucene.* Durham UK: Duke University Press.

Mannion, G., & Lynch, J. (2016). The primacy of place in education in outdoor settings. In B. Humberstone, H. Prince & K. Henderson (Eds.), *Routledge international handbook of outdoor studies* (pp. 85–94). Abingdon, UK: Taylor and Francis.

Mol, A. (2002). *The body multiple: Ontology in medical practice.* Durham, UK: Duke University Press.

St. Pierre, E. (2011). Post qualitative research: The critique and the coming after. In N.K. Denzin & Y.S. Lincoln (Eds.), *The Sage handbook of qualitative research* (Vol. 4, pp. 611–625). Thousand Oaks, CA: Sage.

St. Pierre, E., Jackson, A., & Mazzei, L. (2016). New empiricisms and new materialisms: Conditions for new inquiry. *Cultural Studies ↔ Critical Methodologies*, *16*(2), 99–110.

Stewart, A. (2020). *Developing place-responsive pedagogy in outdoor environmental education: A rhizomatic curriculum autobiography.* Cham, Switzerland: Springer Nature.

Wattchow, B., & Brown, M. (2011). *A pedagogy of place: Outdoor education for a changing world.* Clayton, VIC, Australia: Monash University Publishing.

Publications arising from the thesis

Jukes, S. (2020). Review of Developing place-responsive pedagogy in outdoor environmental education: A rhizomatic curriculum autobiography by Alistair Stewart. *Journal of Outdoor and Environmental Education*, *23*(3), 323–327. DOI 10.1007/s42322-020-00067-2.

Jukes, S. (2020). Posthuman insights for environmental education. A review of Mental health and wellbeing in the Anthropocene: A posthuman inquiry, by Jamie Mcphie. 2019, Singapore: Springer Nature. €69.99, ISBN 978-981-13-3326-2. *The Journal of Environmental Education*, *51*(5), 395–398. DOI 10.1080/00958964.2020.1813678.

Jukes, S., & Reeves, Y. (2020). More-than-human stories: Experimental co-productions in outdoor environmental education pedagogy. *Environmental Education Research*, *26*(9-10), 1294–1312. DOI 10.1080/13504622.2019.1699027.

Jukes, S. (2020). Thinking through making: Junk paddles, distant forests and pedagogical possibilities. *Environmental Education Research*, *26*(12), 1746–1763. DOI 10.1080/13504622.2020.1806991.

Jukes, S. (2021). Thinking with a landscape: The Australian Alps, horses and pedagogical considerations. *Australian Journal of Environmental Education*, *37*(2), 89–107. DOI 10.1017/aee.2020.26.

Jukes, S., Stewart, A., & Morse, M. (2021). Following lines in the landscape: Playing with a posthuman pedagogy in outdoor environmental education. *Australian Journal of Environmental Education*, 1–16. DOI 10.1017/aee.2021.18. Advance online publication.

Stewart, A., Jukes, S., Mikaels, J., & Mangelsdorf, A. (2021). Reading landscapes: Engaging with places. In G. Thomas, J. Dyment & H. Prince (Eds.), *Outdoor environmental education in higher education: International perspectives* (pp. 201–213). Cham: Springer International Publishing. https://link.springer.com/chapter/10.1007%2F978-3-030-75980-3_17

Jukes, S., Clarke, D., & Mcphie, J. (2021). The wisp of an outline ≈ Storying ontology as environmental inquiry↔education : –). *Australian Journal of Environmental Education*, 1–17. DOI 10.1017/aee.2021.31.

Scott Jukes is a lecturer in Outdoor Environmental Education at Federation University, Australia. His research explores pedagogical development and experimentation in outdoor environmental education, inspired by post-humanist and new materialist theories. He is particularly interested in ways we may grapple with place-specific environmental problems and engage with more-than-human worlds. He has a passion for the river, mountain, and coastal environments of south-eastern Australia and enjoys teaching and spending time in these places. Scott was recently nominated for the Australian Association for Research in Education's Ray Debus Award for doctoral research in education. He is also the Media Editor for the Australian Journal of Environmental Education.

Cite this article: Jukes, S. (2022). Enacting more-than-human pedagogies in response to ecological precarity: an immanent praxiography. *Australian Journal of Environmental Education* **0**, 1–3. https://doi.org/10.1017/aee.2022.38
