# Supplementary material for: Dietary fibre intake in the adult Swiss population: a comprehensive analysis of timing and sources
Source: J Nutr Sci. 2025 Mar 24;14:e27. doi: 10.1017/jns.2025.6 (PMC11950702; doi:10.1017/jns.2025.6)
Supplement: von Blumenthal et al. supplementary material 1 — von Blumenthal et al. supplementary material [file S2048679025000060sup001.zip › Supplementary material 1-4_AU.pdf]

British Journal of Nutrition

Nutrient intake variability and number of days needed to estimate usual intake in children and adolescents with autism spectrum disorder

Nutrient variability in patients with autism

Supplementary material 1-4

Eduarda Silva[1]; Laura Hoffmann[1]; Kamila Castro[1,2]; Eliseu Verly Junior[3]; Sandra Valle[1,4]; Juliana Vaz[1,4]

[1]Programa de Pós-Graduação em Nutrição e Alimentos, Universidade Federal de Pelotas – Pelotas (RS), Brasil.

[2]Serviço de Neuropediatria do Hospital de Clínicas de Porto Alegre - Porto Alegre (RS), Brasil.

[3]Departamento de Epidemiologia, Instituto de Medicina Social, Universidade do Estado do Rio de Janeiro - Rio de Janeiro (RJ), Brasil.

[4]Faculdade de Nutrição, Universidade Federal de Pelotas - Pelotas (RS), Brasil.

Supplementary material 1. Distribution of usual energy, macronutrient, and micronutrient intake according to sex of children and adolescents with autism spectrum disorder. Pelotas, Brazil, PANA Study (n=284).

Variables	Boys (n=235)					Girls (n=49)				
	**Mean**	**SD**	**P25**	**P50**	**P75**	**Mean**	**SD**	**P25**	**P50**	**P75**
Energy (kJ)	7705.5	1926.5	6265.0	7595.2	8994.9	6279.4	1989.6	4747.2	5838.8	7265.3
Carbohydrates (g)	257.5	72.7	205.0	249.7	309.2	206.0	64.5	156.9	196.1	257.1
Proteins (g)	61.4	17.4	49.5	59.7	70.7	49.3	16.0	37.2	47.8	57.2
Lipids (g)	63.1	20.3	48.2	60.3	73.6	53.4	23.3	37.0	49.2	59.5
SFA (g)	22.1	6.8	17.0	21.1	26.4	18.8	8.1	12.7	18.3	23.5
MUFA (g)	19.4	7.0	14.1	18.4	23.8	16.2	7.1	11.3	14.2	18.7
PUFA (g)	13.9	6.4	9.5	12.6	17.0	11.6	7.5	7.0	9.0	12.9
Cholesterol (mg)	197	97.9	125.5	182.7	257.1	173.9	91.3	110.3	149.2	220.5
Fiber (g)	17.3	7.6	15.9	21.4	27.3	12.9	8.1	7.5	10.5	18.2
Retinol (mcg)	311.8	207	165.1	282.4	415.9	273.8	156.2	133.3	264.8	372.3
Thiamine (mg)	1.2	0.4	0.9	1.1	1.4	1.0	0.4	0.7	0.9	1.2
Riboflavin (mg)	1.6	0.7	1.2	1.5	2.0	1.3	0.7	0.8	1.3	1.7
Pyridoxine (mg)	1.0	0.4	0.7	0.9	1.2	0.7	0.3	0.5	0.7	0.9
Niacin (mg)	11.8	5.4	8.2	11.2	14.9	9.0	3.3	7.0	8.6	10.3
Vitamin C (mg)	86.9	71.6	33.9	66	118.8	69.0	53.5	25.3	58.7	98.8
Ca (mg)	760.8	364.1	483.4	699.1	1004.7	672.3	422.1	347.1	587.2	868.2
Mg (mg)	223.4	63.0	178.6	218.2	252.1	174.7	64.4	135.5	160.1	209.4
Fe (mg)	8.7	3.3	6.4	8.2	10.7	6.4	2.5	4.4	5.9	7.8
Phosphorus (mg)	1052.2	273.7	858.3	1026.3	1232.6	878.7	370.8	590.6	902.2	1081.8
Na (mg)	2265.5	699.2	1736.5	2193.3	2722.1	1710.1	717.8	1153.7	1652.3	2021.2
Potassium (mg)	2021.2	499.9	1681.5	1955.8	2342.5	1736.7	636.8	1335.2	1665.4	2084.6
Cu (mg)	0.9	0.4	0.7	0.9	1.1	0.7	0.4	0.5	0.7	0.9
Zn (mg)	9.0	3.4	6.6	8.5	10.5	6.9	2.7	4.9	6.4	8.4

Note: Values estimated using the Multiple Source Method® program. Abbreviations: SD = standard deviation; P = percentil.

Supplementary material 2. Distribution of usual energy, macronutrient, and micronutrient intake according to age group of children and adolescents with autism spectrum disorder. Pelotas, Brazil, PANA Study (n=284).

Variables	2-5 years (n=118)					6-9 years (n=97)					10-18 years (n=69)				
	Mean	SD	P25	P50	P75	Mean	SD	P25	P50	P75	Mean	SD	P25	P50	P75
Energy (kJ)	6704.6	2019.3	5361.6	6347.6	7986.8	7659.7	1809.6	6378.3	7543.6	8574.3	8471.1	1729.5	7394.0	8456.9	9652.1
Carbohydrates (g)	218.2	71.4	169.2	205.9	255.8	257.0	71.9	207.1	254.5	291.2	289.0	55.3	247.5	286.7	324.7
Proteins (g)	55.2	16.7	42.7	53.5	65.3	57.9	16.2	48.0	57.0	66.5	68.2	19.3	55.1	66.8	78.6
Lipids (g)	56.7	21.8	39.8	52.9	71.4	63.7	17.8	50.6	59.6	73.2	66.3	23.0	49.8	61.5	79.0
SFA (g)	21.5	8.1	15.6	20.5	26.9	21.4	5.0	18.0	20.7	24.5	21.9	8.2	16.2	20.5	26.2
MUFA (g)	16.7	6.9	11.7	15.0	21.3	19.8	5.8	14.9	19.0	23.9	21.1	7.8	15.9	19.3	25.8
PUFA (g)	11.3	6.2	7.3	10.1	13.7	14.8	7.8	9.6	12.2	18.9	15.5	4.3	12.4	15.4	17.8
Cholesterol (mg)	178.9	101.1	113.2	162.1	209	199.5	104.6	123.7	190.8	257.4	209.2	76.4	159.1	206.9	259.0
Fiber (g)	14.6	7.8	9.0	12.7	19.2	16.6	7.3	11.1	15.8	19.7	19.8	7.4	14.6	18.9	21.8
Retinol (mcg)	337.9	203.7	189.2	311.6	463.4	293	207.6	167.4	260	395.8	266.1	159.0	143.9	234.3	385.7
Thiamine (mg)	1.1	0.5	0.8	1.0	1.4	1.1	0.3	0.9	1.1	1.3	1.2	0.4	0.9	1.2	1.4
Riboflavin (mg)	1.7	0.8	1.1	1.7	2.2	1.5	0.6	1.1	1.4	1.7	1.3	0.5	0.9	1.3	1.6
Pyridoxine (mg)	0.9	0.4	0.6	0.8	1.1	1.0	0.4	0.7	0.9	1.1	1.0	0.3	0.8	1.0	1.2
Niacin (mg)	9.4	4.5	5.7	8.9	12.4	11.5	4.3	8.4	10.6	14.2	14.4	5.7	10.7	13.7	17.1
Vitamin C (mg)	64.5	54	27.9	45.9	90.3	87.1	75.8	31.3	66.5	127.7	111.1	68.1	65.4	97.5	154.9
Ca (mg)	901.4	438.4	603.6	863.8	1163.2	677.3	275.1	478.2	665.7	845.3	573.7	271.8	370.9	538.8	688.0
Mg (mg)	205.5	66.7	158.9	195.9	239	213.3	62.1	175.0	203.1	243.8	233.3	68.1	185.3	226.0	257.4
Fe (mg)	7.3	3.3	4.9	6.6	9.1	8.5	3.3	6.2	7.9	10.7	9.7	2.3	8.2	9.5	11.2
Phosphorus (mg)	1063	341.6	806.7	1023.7	1300	979.6	249.1	837.9	973.6	1120.6	1012.1	288.0	812.8	994.6	1210.2
Na (mg)	1805.6	625.3	1368.7	1764.8	2277.1	2233.9	606.2	1789.1	2178.8	2646.2	2702.1	725.1	2089	2633.3	3243.0
Potassium (mg)	1962.4	596.7	1572.6	1867.4	2328.2	1930.8	437.8	1678.9	1857.0	2189.8	2045.2	568.9	1585.3	2073.9	2435.9
Cu (mg)	0.8	0.4	0.5	0.7	1.0	0.9	0.4	0.7	0.9	1.2	1.1	0.4	0.9	1.0	1.2
Zn (mg)	7.9	3.1	5.7	7.7	9.3	8.7	3.5	6.3	8.5	10.1	9.8	3.5	7.5	9.2	11.1

Note: Values estimated using the Multiple Source Method® program. Abbreviations: SD = standard deviation; P = percentil.

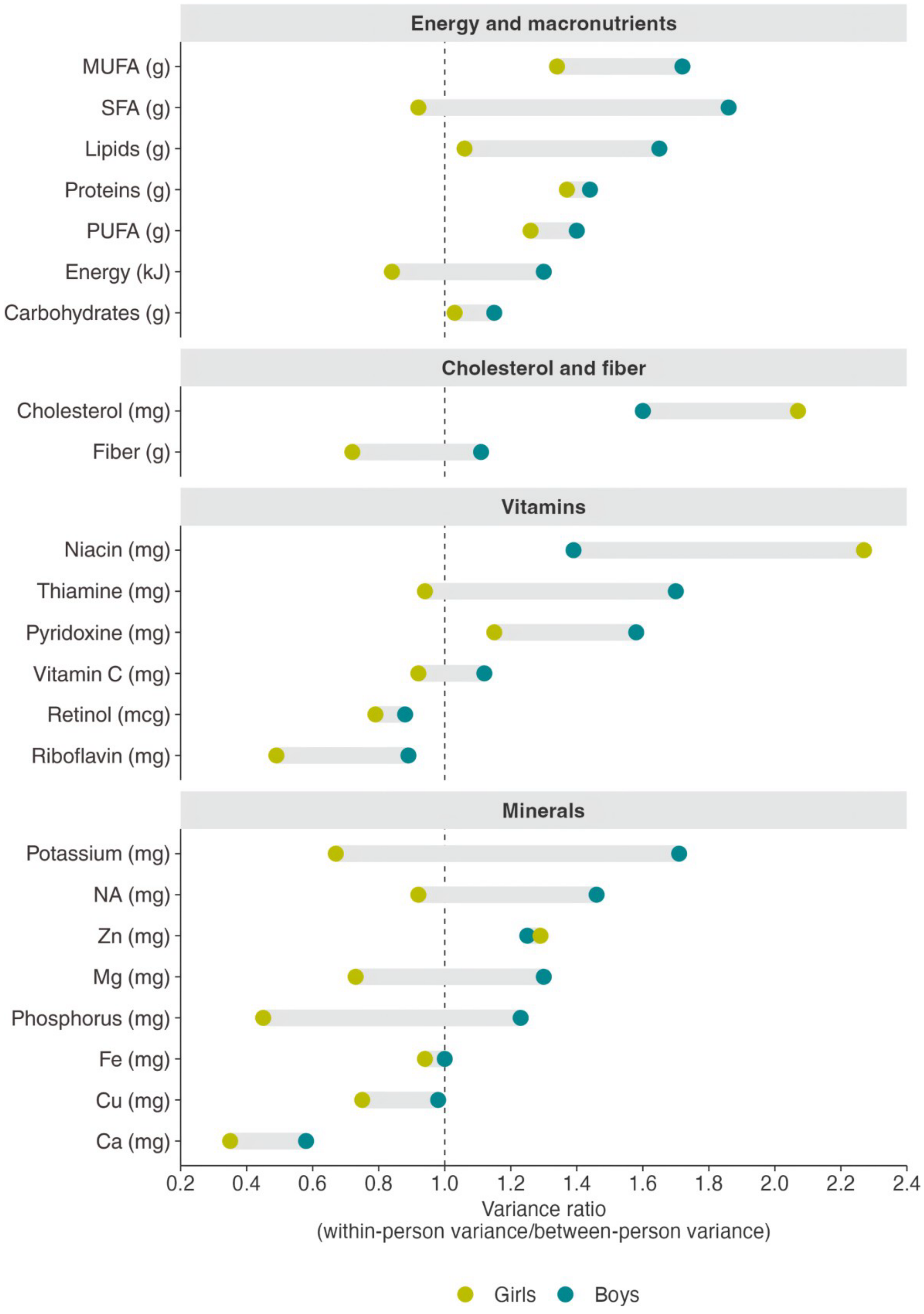

Supplementary material 3. Variance ratio of energy, macronutrients, and micronutrients according to sex of children and adolescents with autism spectrum disorder. Pelotas, Brazil, PANA Study (n=284)

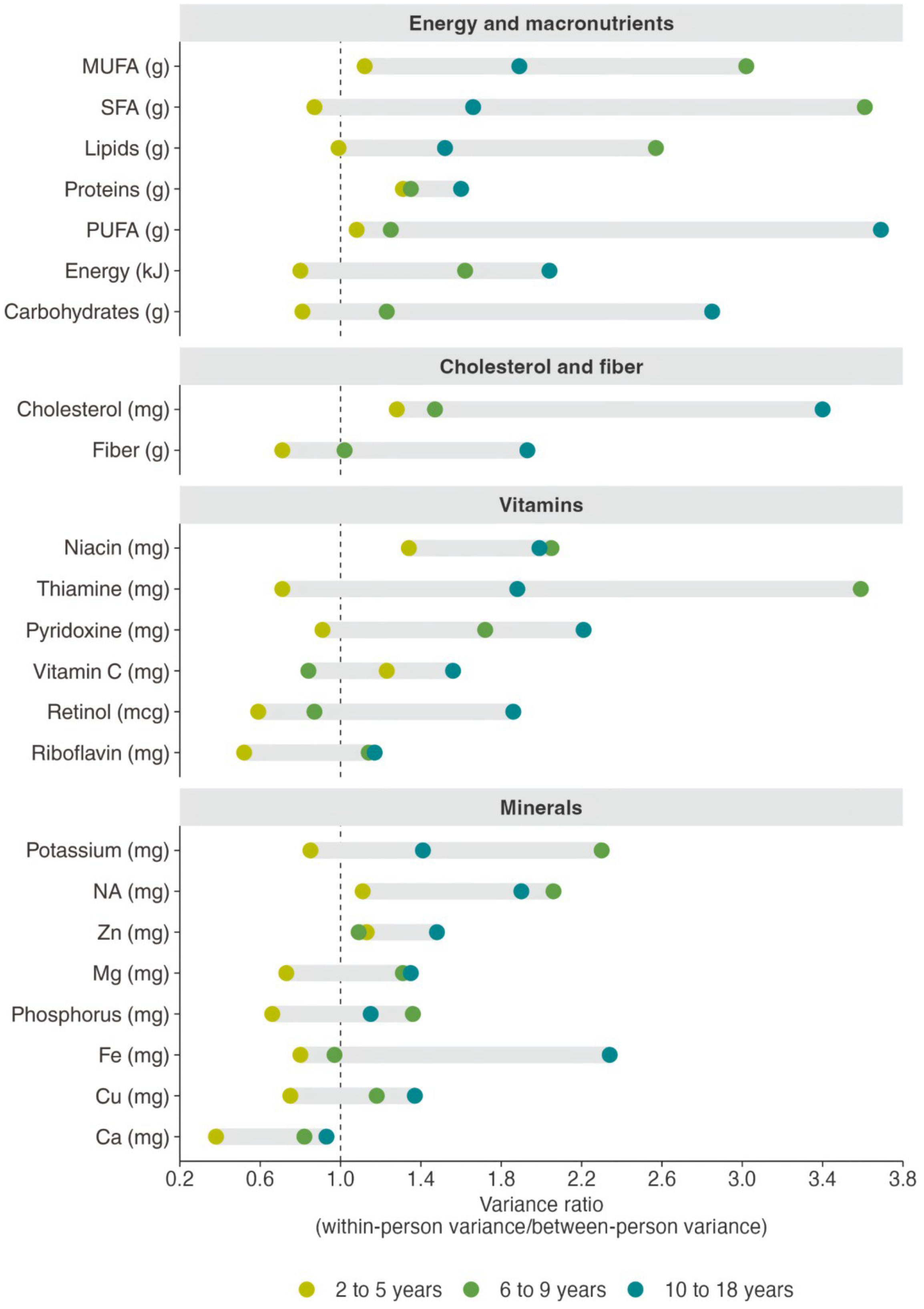

Supplementary material 4. Variance ratio of energy, macronutrients, and micronutrients according to age group of children and adolescents with autism spectrum disorder. Pelotas, Brazil, PANA Study (n=284)
