# Supplementary material for: Dietary fibre intake in the adult Swiss population: a comprehensive analysis of timing and sources
Source: J Nutr Sci. 2025 Mar 24;14:e27. doi: 10.1017/jns.2025.6 (PMC11950702; doi:10.1017/jns.2025.6)
Supplement: von Blumenthal et al. supplementary material 1 — von Blumenthal et al. supplementary material [file S2048679025000060sup001.zip › Anthropometric characteristics and indicators of eating and physical activity behaviors in the Swiss adult population 2014-2015.pdf]

menuCH

Nationale Ernährungserhebung
Enquête nationale sur l'alimentation
Sondaggio nazionale sull'alimentazione

Anthropometric characteristics and indicators of eating and physical activity behaviors in the Swiss adult population

Results from menuCH 2014-2015

Report on behalf of the Federal Office of Public Health and the Food Safety and Veterinary Office
Contract Nr. OFSP 16.010537/204.0001/-1476 and contract Nr. FSVO 0714000510.

Authors
Murielle Bochud*, Angéline Chatelan and Juan-Manuel Blanco
CHUV, Institut Universitaire de Médecine Sociale et Préventive (IUMSP), Lausanne

Sigrid Beer-Borst
Universität Bern, Institut für Sozial und Präventivmedizin (ISPM), Bern

*Address for Correspondence:
Prof. Murielle Bochud, MD, PhD
Institut Universitaire de Médecine Sociale et Préventive (IUMSP)
Route de la Corniche 10
CH-1010 Lausanne

14.03.2017

Table of contents

LIST OF TABLES 4

LIST OF FIGURES 4

ABSTRACT (ENGLISH) 7

ABSTRACT (GERMAN) 8

ABSTRACT (FRENCH) 10

ABSTRACT (ITALIAN) 12

EXECUTIVE SUMMARY (ENGLISH) 14

EXECUTIVE SUMMARY (GERMAN) 17

EXECUTIVE SUMMARY (FRENCH) 20

EXECUTIVE SUMMARY (ITALIAN) 23

1. INTRODUCTION 26

2. STUDY DESIGN AND METHODS 26

2.1. Sampling frame and sample weighting 26

2.2. Questionnaire - Participant characteristics & Eating and Physical activity behaviors 27

2.3. Anthropometrics 28

2.4. Food consumption 28

2.5. Statistical analysis 29

3. RESULTS **29**

3.1. Description of the male and female population **29**

3.2. Body weight status and body image **33**

3.2.1. Measured anthropometric data *33*

3.2.2. Measured compared to self-reported anthropometric data *36*

3.2.3. Waist circumference data *38*

3.2.4. Body weight satisfaction and weight management *41*

3.3. Nutrition **50**

3.3.1. Diet related behaviors *50*

3.3.2. Cooking and eating habits *55*

3.4. Physical activity and sedentarity **68**

3.4.1. Physical activity levels *68*

3.4.2. Walking time *70*

3.4.3. Sedentary lifestyle *71*

4. DISCUSSION **72**

5. CONCLUSIONS **76**

ACKNOWLEDGEMENT **77**

6. REFERENCES **78**

List of tables

Table 1 Socio-demographic and health related characteristics of the survey population (menuCH). . 30
Table 2. Socio-demographic and health related characteristics in the Swiss Health Survey 2012. 31

List of figures

Figure 1 Weighting and calibrating for target population representative data. 27
Figure 3 Average body mass index (BMI in kg/m^2; measured weight, height) overall, by sex linguistic region. 33
Figure 4 Average body mass index (BMI in kg/m^2; measured weight, height), overall, by sex and age groups. 33
Figure 7 Relative frequency (%) of body mass index categories (measured data) overall, by sex and age groups. 35
Figure 8 Relative frequency (%) of body mass index categories (measured data) by linguistic regions and educational level. 36
Figure 9 Distribution of measured and self-reported weight (kg) and height (cm), by sex. 37
Figure 10 Bland-Altman plot for body mass index based on self-reported vs measured weight and height. 37
Figure 11 Relative frequency (%) of waist circumference risk categories by linguistic regions for men, women. 38
Figure 12 Relative frequency (%) of waist circumference risk categories by age groups overall............ 39
Figure 13 Relative frequency (%) of waist circumference risk categories by age groups for men and women. 39
Figure 14 Relative frequency (%) of waist circumference risk categories by educational level for men, women and overall. 40
Figure 15 Relative frequency (%) of body mass index categories by waist circumference risk categories (normal vs increased) overall, in men and in women.. 41
Figure 16 Relative frequencies (%) of body weight satisfaction categories, overall, by sex and age groups. 42
Figure 17 Relative frequencies (%) of body weight satisfaction categories, by age groups, in men and women. 42
Figure 18 Relative frequencies (%) of body weight satisfaction categories, overall, by sex and educational levels. 43
Figure 19 Relative frequencies (%) of body weight satisfaction categories across linguistic regions, in men and in women 43
Figure 20 Relative frequencies (%) of body mass index categories by level of body weight satisfaction, overall, in men and women 44

Figure 21 Relative frequencies (%) of weight management plan categories, overall, by sex and age groups. 45
Figure 22 Relative frequencies (%) of weight management plan categories, by age groups, for men and women. 45
Figure 23 Relative frequencies (%) of weight management plan categories, by educational levels for men, women and overall. 46
Figure 24 Relative frequencies (%) of weight management plan categories, by linguistic regions, overall, for men and women. 46
Figure 25 Percentage (%) reporting to be on a weight loss diet, overall, by sex and by age groups. 47
Figure 26 Percentage (%) reporting to be on a weight loss diet, by age groups for men and women. 48
Figure 27 Percentage (%) reporting to be on a weight loss diet, by educational levels, overall, for men and women. 48
Figure 28 Percentage (%) reporting to be on a weight loss diet, by linguistic regions, overall, for men and women. 49
Figure 29 Relative frequencies (%) of body mass index categories by weight loss plan, overall, for men and women. 50
Figure 30 Percentage (%) reporting any type of special diet, overall, by sex, age groups, linguistic region and educational level. 51
Figure 31 Percentage (%) reporting selected special diets (reported at least 20 times), overall, by sex, age groups, linguistic region and educational level. 51
Figure 32 Percentage (%) reporting other special diets, overall, by sex, age groups, linguistic region and educational level. 52
Figure 33 Percentage (%) of overall supplement use, overall, by sex, age groups, linguistic region and educational level. 53
Figure 35 Percentage (%) of people having heard about the Swiss food pyramid, overall, by sex, age groups, linguistic region and educational level. 54
Figure 36 Percentage (%) of people having heard about the 5-a-day campaign, overall, by sex, age groups, linguistic region and educational level. 55
Figure 37 Relative frequencies (%) of never self-cooking hot meals at home for lunch or for dinner, overall, by sex, age groups, linguistic region and educational level. 56
Figure 38 Percentage (%) of days with self-cooking hot meals at home during weekdays (Monday to Friday) and weekends (Saturday and Sunday), overall, by sex, age groups, linguistic region and educational level. 56
Figure 39 Average time (in minutes) spent to prepare hot meals, overall, by sex, age groups, linguistic region and educational level. 57
Figure 40 Relative frequencies (%) of spending a long time (i.e. >40 minutes) cooking hot meals, overall, by sex, age, linguistic region and educational level. 58
Figure 41 Average number of days (out of 7 days) having breakfast, lunch or dinner, overall, by sex, age groups, linguistic region and educational level. 58

Figure 42 Relative frequencies (%) of never having breakfast, lunch or dinner, overall, by sex and age groups. 59
Figure 43 Relative frequencies (%) of never having breakfast, lunch or dinner, by linguistic region and educational level. 60
Figure 44 Relative frequencies (%) of never having breakfast, lunch or dinner out of home, overall, by sex and age groups. 60
Figure 45 Relative frequencies (%) of never having breakfast, lunch or dinner out of home, by linguistic region and educational level. 61
Figure 46 Relative frequencies (%) of always having breakfast, lunch or dinner at home alone during weekdays, overall, by sex, age, linguistic region and educational level. 62
Figure 47 Relative frequencies (%) of always having breakfast, lunch or dinner at home alone during weekends, overall, by sex, age, linguistic region and educational level. 62
Figure 48 Snacking (solid foods) at least 3 times a day any day, during weekdays or weekend days, overall, by sex, age groups, linguistic region and educational level. 63
Figure 50 Type of salt use at home, overall, by sex, age groups, linguistic and region educational level. 65
Figure 51 Discretionary salt use at home, overall, by sex and age groups. 66
Figure 52 Discretionary salt use at home, overall, by linguistic region and educational level. 66
Figure 53 Discretionary salt use outside home, overall, by sex and age groups. 67
Figure 54 Discretionary salt use outside home, by linguistic region and educational level. 67
Figure 55 Physical activity categories derived from IPAQ-SF questionnaire, among people who know, overall, by sex and age groups. 69
Figure 56 Physical activity categories derived from IPAQ-SF questionnaire, among people who know, by linguistic region and educational level. 69
Figure 57 Relative frequencies of walking time categories, overall, by sex and age groups. 70
Figure 58 Relative frequencies of walking time categories, by linguistic region and educational level. . 70
Figure 59 Categories of average sitting or lying time, without sleeping, during weekdays, overall, by sex and age groups. 71
Figure 60 Categories of average sitting or lying time, without sleeping, during weekdays, by linguistic region and educational level. 71

Abstract (English)

Background: So far, Switzerland could not rely on nationally representative data on measured anthropometric data and eating behaviors when establishing health related strategies and guidelines. The data from the first National Nutrition Survey for adults (menuCH) now complement information from other surveys, to inform public health policies and health professionals.

Setting: One-year cross-sectional nutrition survey conducted from January 2014 till February 2015. Data were collected on 2085 participants aged 18-75 years representing 4'622'018 inhabitants (49.9% men and 50.1% women) residing in the three main linguistic regions of Switzerland (German, French and Italian).

Methods: Interviews were carried out in German, French or Italian by trained dieticians in 10 study centers. Participants provided written informed consent. Respondent completed a self-administered paper-pencil dietary and physical activity behavior questionnaire including reported anthropometric and sociodemographic characteristics. Body weight, height and waist circumference were measured using standardized procedures. Body mass index (BMI) and waist circumference were categorized using WHO criteria. After sample weighting and calibration, descriptive stratified statistical analysis was performed, considering linguistic regions, sex, age groups and educational levels.

Results: The net response rate was 38%. Average BMI was 25.9 kg/m^2 for men and 24.1 kg/m^2 for women, with little differences across linguistic regions. Mean BMI was 23.5, 25.0, 25.9 and 26.1 kg/m^2 in the 18-34, 35-49, 50-64 and 65-75 year categories, respectively. The prevalence of overweight and obesity was 41.6% and 13.9% in men, 19.7% and 11.3% in women, 31.0% and 12.5% in the German-speaking region, 29.9% and 12.3% in the French-speaking region, and 30.1% and 15.6% in the Italian-speaking region, respectively. The prevalence of waist circumference at increased and highly increased metabolic risk was 16.7% and 16.5% overall, 18.6% and 16.4% in men as well as 14.8% and 16.6% in women, respectively. About 53% of the population wishes to reduce body weight. Three out of four people in the population have heard about the food pyramid and two-third about five fruits and vegetables a day. Among special diets, 4.9% of the population report to follow a vegetarian diet, 4.1% an energy restriction diet, 3.3% a fat restriction diet and 2.6% a lactose-free diet. A substantial proportion of the population (56.4% of women and 38.1% of men) reports to take vitamin or mineral supplements. The majority of the population (>80%) takes a snack at least once per day, with similar pattern during weekdays and weekends. Women are more likely to spend a long time cooking (>40 minutes) than men (50.3% vs 30.7%). The most frequently consistently skipped meal is breakfast for 5.2% of the population, followed by lunch (2.2%) and dinner (0.6%). Nearly 50% of the population report to walk at least 30 minutes per day, five days per week. The majority of the population (87.0%) reports to be trained, regularly physically active or irregularly active, thereby meeting current recommendations. One third of the population reports a sitting time higher than 8h30 minutes per day, which reflects a high level of sedentarity.

Conclusion: menuCH, the first National Nutrition Survey for adults in Switzerland, provides important novel information on overweight, obesity and waist circumference based on measured data, in the population aged 18-75 years. The survey also shows that knowledge about dietary recommendations is very good, vitamin and mineral supplements are frequently consumed and self-reported cooking habits differ by sex. Reported physical activity levels are quite high, despite a high level of sedentarity.

Abstract (German)

Hintergrund: Bisher konnte man in der Schweiz bei der Entwicklung von gesundheitsbezogenen Strategien und Empfehlungen auf keine national repräsentativen, objektiv gemessenen anthropometrischen Daten sowie Daten zum Ernährungsverhalten zurückgreifen. Die Erkenntnisse der ersten Nationalen Ernährungserhebung bei Erwachsenen (menuCH) ergänzen Informationen aus früheren Erhebungen, um in Strategien zur Förderung der öffentlichen Gesundheit einzufliessen und Gesundheitsfachpersonen in ihrer Tätigkeit zu unterstützen.

Setting: Die vorliegende Ernährungserhebung ist eine Querschnittsstudie, die während eines Jahres, von Januar 2014 bis Februar 2015, durchgeführt wurde. Es wurden Daten von 2085 Teilnehmenden im Alter von 18 bis 75 Jahren erfasst, welche 4’622’018 Einwohner/-innen (49.9% Männer und 50.1% Frauen) mit Wohnsitz in den drei grössten Sprachregionen der Schweiz (Deutsch, Französisch und Italienisch) repräsentieren.

Methoden: Die Befragungen wurden von geschulten Ernährungsberatern/-innen in deutscher, französischer oder italienischer Sprache in 10 Studienzentren durchgeführt. Vor der Teilnahme musste jede Person eine schriftliche Einverständniserklärung unterzeichnen. Die Teilnehmenden füllten handschriftlich einen Fragebogen aus, der Fragen zum Ess- und Bewegungsverhalten aber auch anthropometrische Angaben und sozio-demographische Charakteristika umfasste. Körpergewicht, Körpergrösse und der Bauchumfang wurde Standardprotokollen folgend gemessen. Der Body Mass Index (BMI) und der Bauchumfang wurden anhand anerkannter WHO-Definitionen kategorisiert. Die deskriptive statistische Auswertung erfolgte nach Gewichtung der Daten und stratifiziert nach Sprachregionen, Geschlecht, Altersgruppen und Bildungsniveau.

Ergebnisse: Die Netto-Antwortquote lag bei 38%. Der durchschnittliche BMI der Männer betrug 25.9 kg/m^2 und für die Frauen 24.1 kg/m^2, bei nur geringen sprachregionalen Unterschieden. Nach Altersgruppen betrug der BMI 23.5 (18-34 J.), 25.0 (35-49 J.), 25.9 (50-64 J.) und 26.1 (65-75 J.) kg/m^2. Die Prävalenz von Übergewicht/Adipositas betrug bei den Männern 41.6%/13.9%, bei den Frauen 19.7%/11.3%, sowie nach Sprachregionen 31.0%/12.5% in D-CH, 29.9%/12.3% in F-CH und 30.1%/15.6% in I-CH. Die Prävalenz eines Bauchumfangs, der mit einem erhöhten/erheblich erhöhten metabolischen Risiko verbunden ist betrug gesamthaft betrachtet 16.7%/16.5%, für Männer 18.6%/16.4% sowie für Frauen 14.8%/16.6%. Gut die Hälfte (53%) der Bevölkerung wollte gerne Gewicht verlieren. Drei von vier Personen in der Bevölkerung haben bereits von der „Schweizer Lebensmittelpyramide“ gehört, und zweidrittel der Bevölkerung von der „5-am-Tag“-Kampagne. Befragt nach speziellen Ernährungsformen gaben 4.9% der Bevölkerung an sich vegetarisch zu ernähren, 4.1% berichteten eine energiearme und 3.3% eine fettreduzierte Ernährung einzuhalten und 2.6% ernährten sich Lactose frei. Ein beachtlicher Anteil der Bevölkerung (56.4% der Frauen und 38.1% der Männer) gaben an Vitamin- oder Mineralstoffpräparate einzunehmen. Die Mehrheit der Bevölkerung (>80%) nehmen mindestens eine Zwischenmahlzeit pro Tag zu sich, unter der Woche wie am Wochenende. Mehr Frauen (50.3%) als Männer (30.7%) wenden viel Zeit (>40 Minuten) für das Kochen warmer Mahlzeiten auf. Die am häufigsten und regelmässig ausgelassene Mahlzeit ist für 5.2% der Bevölkerung das Frühstück, gefolgt vom Mittagessen (2.2%) und Abendessen (0.6%). Fast 50% der Bevölkerung berichteten mindestens während 30 Minuten pro Tag an mehr als fünf Tagen pro Woche zu Fuss zu gehen. Ein Grossteil (87%) der Bevölkerung gab an zu trainieren resp. regelmässig oder unregelmässig körperlich aktiv zu sein,

womit sie geltende Bewegungsempfehlungen erfüllten. Ein Drittel der Bevölkerung gab an täglich mehr als 8 Stunden und 30 Minuten sitzend zu verbringen, was einem hohen Niveau der Sedentarität entspricht.

Schlussfolgerung: menuCH, die erste Nationale Ernährungserhebung in der Schweiz, liefert wichtige neue Informationen zur Prävalenz von Übergewicht und Adipositas in der erwachsenen, 18 bis 75-jährigen Bevölkerung, u.a. basierend auf objektiven Messungen des Bauchumfangs. Die Befragung zeigte zudem, dass der Bekanntheitsgrad der Ernährungsempfehlungen sehr gut ist, Vitamin- und Mineralstoffpräparate von vielen Personen konsumiert werden und sich die Kochgewohnheiten von Frauen und Männern unterscheiden. Das berichtete Ausmass an körperlicher Aktivität ist ziemlich hoch, trotz des hohen Sedentaritätsniveaus.

Abstract (French)

Contexte: Jusqu'à ce jour, la Suisse ne disposait pas de données nationales représentatives de la population sur des paramètres anthropométriques mesurés, ainsi que sur les comportements alimentaires pour établir des stratégies de santé et des recommandations. Les données de la première Enquête Nationale sur l'Alimentation (menuCH) complètent désormais celles d'enquêtes précédentes, comme les Enquêtes Suisses sur la Santé ou l'Enquête Suisse sur le Sel, pour informer les politiques de santé publique et les professionnels de la santé.

Cadre: Enquête nationale de population, transversale, conduite de janvier 2014 à février 2015. Les données collectées sur 2085 participants âgés de 18-75 ans représentent 4'622'018 habitants (49.9% hommes et 50.1% femmes) qui résident dans les trois régions linguistiques principales de la Suisse (alémanique, romande et italienne).

Méthodes: Les entretiens ont été réalisés en allemand, français ou italien par des diététiciennes formées dans 10 centres d'étude. Après avoir signé un consentement éclairé, les participants ont complété un questionnaire papier, portant sur les comportements alimentaires et sur l'activité physique ainsi que sur des caractéristiques anthropométriques et sociodémographiques. Le poids, la taille et le tour de taille ont été mesurés de façon standardisée. L'indice de masse corporelle (IMC) et le tour de taille ont été catégorisés selon les définitions de l'OMS. Des analyses descriptives pondérées ont été faites, en stratifiant par sexe, âge, régions linguistiques et niveau d'éducation.

Résultats: Le taux de réponse net était de 38%. L'IMC moyen était de 25.9 kg/m^2 chez les hommes et de 24.1 kg/m^2 chez les femmes, avec peu de différences interrégionales. L'IMC moyen, basé sur des mesures, était de 23.5, 25.0, 25.9 et 26.1 kg/m^2 dans les tranches d'âge de 18-34, 35-49, 50-64 et 65-75 ans, respectivement. La prévalence de surpoids et l'obésité était de 41.6% et 13.9% chez les hommes, 19.7% et 11.3% chez les femmes, 31.0% et 12.5% en Suisse alémanique, 29.9% et 12.3% en Suisse romande, et 30.1% et 15.6% en Suisse italienne, respectivement. La prévalence d'un tour de taille à risque cardio-métabolique élevé, et très élevé, était de 16.7% et 16.5% globalement, 18.6% et 16.4% chez les hommes ainsi que 14.8% et 16.6% chez les femmes, respectivement. Environ 53% des personnes dans la population souhaitent perdre du poids. Trois personnes sur 4 dans la population ont entendu parler de la pyramide alimentaire suisse et deux tiers des personnes connaissaient la campagne «cinq par jour». Parmi les régimes particuliers, 4.9% de la population rapporte suivre un régime végétarien, 4.1% un régime de restriction calorique, 3.3% un régime pauvre en graisse et 2.6% un régime sans lactose. Une proportion substantielle de la population (56.4% des femmes et 38.1% des hommes) a rapporté prendre des suppléments vitaminiques et/ou minéraux. La majorité de la population (>80%) prend un snack au moins une fois par jour, avec des comportements semblables durant les jours de la semaine ou ceux du week-end. Plus de femmes que d'hommes rapportent passer un long temps (c.à.d. >40 minutes) à cuisiner des repas chauds (50.3% vs 30.7%, respectivement). Le repas le plus fréquemment sauté est le petit déjeuner (5.2%), suivi du repas de midi (2.2%) et de celui du soir (0.6%). Environ la moitié de la population rapporte marcher au moins 30 minutes par jour, plus de 5 jours par semaine. La majorité des personnes dans la population (87%) rapporte être entraînée, régulièrement physiquement active ou irrégulièrement active, et remplit ainsi les recommandations actuelles. Un tiers de la population rapporte passer plus de 8h30 par jour en position assise, ce qui reflète un haut niveau de sédentarité.

Conclusion: menuCH, la première Enquête Nationale sur l'Alimentation, apporte de nouvelles informations importantes sur le surpoids, l'obésité et le tour de taille, se basant sur des données mesurées, chez les personnes âgées de 18 à 75 ans vivant dans les trois régions linguistiques principales de la Suisse. L'enquête montre également que les connaissances sur les recommandations nutritionnelles sont très bonnes, que des suppléments vitaminiques et/ou minéraux sont fréquemment consommés, et que les façons de cuisiner rapportées par les hommes sont différentes de celles rapportées par les femmes. Les niveaux d'activité physique rapportés sont très élevés, malgré un haut niveau de sédentarité.

Abstract (Italian)

Quadro generale: Finora, nella concezione di strategie di salute pubblica e nella determinazione di linee guida la Svizzera non ha potuto fare affidamento su dati nazionali rappresentativi della popolazione su misurazioni antropometriche oggettive, alimentazione ed attività fisica. I dati del primo Sondaggio Nazionale sulla Nutrizione per adulti (menuCH) sono ora disponibili e completano l'informazione ottenuta da sondaggi precedenti nell' informare le politiche sanitarie pubbliche e gli operatori sanitari.

Contesto: Un'indagine nutrizionale trasversale di un anno condotta tra il Gennaio del 2014 e il Febbraio del 2015. L'indagine include dati raccolti su 2085 participanti di età compresa tra i 18 e i 75 anni e rappresentativi della popolazione di 4'622'018 abitanti (49.9% uomini e 50.1% donne) residenti nelle tre principali regioni linguistiche della Svizzera (Tedesca, Francese e Italiana).

Metodi: Le interviste sono state condotte in 10 centri in tedesco, francese o italiano da nutrizioniste specificamente formate. I partecipanti hanno firmato un consenso informato scritto ed hanno completato un questionario scritto sulle loro abitudini alimentari e di attività fisica, dove hanno inoltre riportato le loro caratteristiche antropometriche e socio-demografiche. Il peso corporeo, l'altezza e la circonferenza della vita sono stati misurati utilizzando procedure standardizzate, con rigorose procedure di controllo. L'indice di massa corporea e l'Indice di massa corporea (BMI) e la circonferenza della vita sono stati categorizzati seguendo le raccomandazioni dell'OMS. L'analisi statistica descrittiva stratificata ponderata è stata eseguita considerando le regioni linguistiche, il sesso, l'età e il livelli di istruzione.

Risultati: Il tasso di risposta netto è stato del 38%. Il BMI medio misurato era di 25,9 kg / m2 per gli uomini e di 24,1 kg / m2 per le donne, con piccole differenze tra le regioni linguistiche. Il BMI medio misurato era di 23.5, 25.0, 25.9 e 26.1 kg/m2 nelle categorie d'età 18-34, 35-49, 50-64 e 65-75, rispettivamente. La prevalenza di sovrappeso e obesità, in base ai dati misurati, è stata del 41,6% e del 13,9% negli uomini, del 19,7% e del 11,3% nelle donne, del 31,0% e del 12,5% nella regione di lingua tedesca, del 29,9% e 12,3% nella regione linguistica francese , e del 30,1% e 15,6% nella regione di lingua italiana, rispettivamente. La prevalenza di circonferenza della vita ad aumentato e altamente aumentato rischio metabolico è stata del 16,7% e del 16,5% in totale, del 18,6% e del 16,4% negli uomini e del 14,8% e del 16,6% nelle donne, rispettivamente. Il 53% della popolazione dichiara di voler ridurre il proprio peso corporeo. Tre persone su quattro avevano già sentito parlare della "piramide alimentare svizzera" e due terzi della campagna "5 porzioni di frutta e verdura al giorno". Tra le diete speciali, il 4.9% della popolazione dichiara di seguire una dieta vegetariana, il 4.1% una dieta a restrizione calorica, il 3.3% una dieta a riduzione dell'apporto di grassi e il 2.6% una dieta senza lattosio. Una parte consistente della popolazione (56,4% delle donne e il 38,1% degli uomini) riferisce di prendere integratori vitaminici o minerali. La maggioranza della popolazione fa uno spuntino almeno una volta durante la giornata (>80%), con proporzioni simili durante la settimana o il fine settimana. Molte più donne che uomini hanno riferito di spendere molto tempo (definito come più di 40 minuti) a cucinare (50.3% vs 30.7%). Il pasto più frequentemente saltato è risultato essere la colazione (5.2% della popolazione), seguito dal pranzo (2.2%) e dalla cena (0.6%). Nel complesso, il 50% della popolazione ha dichiarato di camminare almeno 30 minuti al giorno, per 5 giorni a settimana. La maggior parte della popolazione (87,0%) riferisce di essere allenata o di praticare regolarmente o irregolarmente attività fisica, in linea con le raccomandazioni attuali. Un terzo della popolazione riferisce di passare più di 8 ore e trenta al giorno in posizione seduta, il che riflette un elevato livello di sedentarietà.

Conclusioni: MenuCH, la prima indagine nazionale sulla nutrizione degli adulti Svizzeri, fornisce nuove ed importanti informazioni sul sovrappeso, l'obesità e la circonferenza vita della popolazione Svizzera tra i 18 e i 75 anni di età, basate su dati misurati. L'indagine mostra anche che la popolazione ha una buona conoscenza delle raccomandazioni nutrizionali, che supplementi di vitamine e minerali sono consumati con frequenza e che le abitudini di preparazione dei pasti sono diverse negli uomini e nelle donne. I livelli di attività fisica riportati sono inoltre relativamente alti, nonostante un livello di sedentarietà altrettanto elevato.

Executive Summary (English)

Chapter 1 presents a short introduction of the context. So far, Switzerland could not rely on national representative data on measured anthropometric data and eating behaviors behaviors when establishing health related strategies and guidelines. The data from the first National Nutrition Survey for adults (menuCH) now complement information from prior surveys, such as the Swiss Health Survey and the Swiss Salt Study, to inform public health policies and health professionals. This report will help policy makers in preparing actions for the new nutrition strategy (2017-2020) as well as for the national strategy for the prevention of non-communicable diseases (2017-2024).

Chapter 2 presents the study design and methods. menuCH was a cross-sectional population survey in 18 to 75 years old adults residing in the three main linguistic regions of Switzerland (short German D-CH, French F-CH, Italian I-CH). Participation was voluntary and travel costs/time investment compensated. The survey was approved by regional ethical committees and participants signed a written informed consent. The sampling frame was established to provide a final sample of 2000 free-living individuals aged 18 to 75 years that is representative of the three main linguistic regions of Switzerland (German, French and Italian), balanced with respect to the predefined sex and age strata within each linguistic region. The multiple step stratified sampling targeted 4'627'878 free-living 18-75 years old women and men residing in the 11 most populous cantons of the seven administrative regions of Switzerland. Participants completed a 49-item paper-pencil questionnaire at home, prior to their face-to-face interview in the study center. The questionnaire was available in three languages and provided information on socio-demographic characteristics, health-related issues, body weight satisfaction, cooking habits as well as on eating and physical activity behaviors (IPAQ-SF). Body weight, height and waist circumference were measured using standardized procedures, with stringent quality control procedures. Body mass index (BMI) and waist circumference were categorized following WHO definitions. Weighted descriptive stratified statistical analysis was performed, considering linguistic regions, sex, age groups and educational levels.

Chapters 3 and 4 present the results and discussion. The net response rate was 38%, which is not high, yet similar to other surveys including face-to-face interviews conducted in Switzerland. Data included in the present report are likely to inadequately reflect the situation of non-Swiss citizens. Overall 87.3% of the population perceived their general health state as being good or very good, in line with what was reported in the Swiss Health Survey 2012 (83%).

Average measured BMI was 25.9 kg/m^2 for men and 24.1 kg/m^2 for women, with little differences across linguistic regions. Mean measured BMI was 23.5, 25.0, 25.9 and 26.1 kg/m^2 in the 18-34, 35-49, 50-64 and 65-75 year categories, respectively. The prevalence of overweight and obesity was 41.6% and 13.9% in men, 19.7% and 11.3% in women, 31.0% and 12.5% in the German-speaking region, 29.9% and 12.3% in the French-speaking region, and 30.1% and 15.6% in the Italian-speaking region, respectively. The prevalence of waist circumference at increased and highly increased metabolic risk was 16.7% and 16.5% overall, 18.6% and 16.4% in men as well as 14.8% and 16.6% in women, respectively.

Overall 55% of women and 50% of men would like to lose weight, whereas only 5% of the population wishes to gain weight. Weight maintenance becomes more important with increasing age. Overall, vegetarian diet is the most prevalent form of a special diet, followed by energy and fat restriction, which may be related to constant weight control endeavors.

A substantial proportion of the population (56.4% of women and 38.1% of men) reports to take vitamin or mineral supplements, with similar figures across educational levels and higher proportion among the older age groups. Minerals are more frequently reported to be used than vitamins, or combined products.

The federal authorities promote different tools and runs nutrition campaigns to support the population in implementing a health-promoting lifestyle. Three out of four people in the population had heard about the “Swiss food pyramid“. Awareness about the “Swiss food pyramid“ was higher in women than in men, and in highly educated people than in people with lower level of education. Awareness of the food pyramid was much higher in younger than in older people. Two-third of people knew about the “5 per day” campaign, in particular in the French-speaking region and among women.

When asked about cooking hot meals at home during a usual week, 35% report to never cook a hot meal themselves for lunches and 19% for dinners. Sex differences were observed with 45% of men vs 25% of women reporting to never cook a hot lunch themselves at home. A sharp contrast was observed between lunch and dinner with respect to age groups. About one third of participants never cook a hot lunch at home themselves irrespective of age group, while much less young people (15% for the 18-34 years old group, 13% for the 35-49 years old group) than older people (35% for the 65-75 years old group) report never to cook a hot dinner at home themselves. More women reported to spend a long time, defined as more than 40 minutes, cooking hot meals than men (50% vs 31%, respectively). The most frequently consistently skipped meal is breakfast for 5.2% of the population, followed by lunch (2.2%) and dinner (0.6%).

People consume foods during the traditional meals of breakfast, lunch and dinner but also outside/between these meals. These eating occasions between main meals are called “snacks” (in German ‘Zwischenmahlzeiten’) and we use the term “snacking” as suggested when referring to the “act of eating a snack, regardless of whether healthful choices or ‘snack foods’ are consumed”. The majority of the population (>80%) takes a snack at least once per day (excluding drinks), with similar pattern during weekdays or weekends. For soft drinks, 20% overall reported to consume them during snacks, with a much higher prevalence in men (27%) than in women (12%) and with a steep age-related decrease (32%, 18%, 14%, 8% for 18-34, 35-49, 50—64 and 65-75 years old, respectively). These results highlight the importance of targeting young people to decrease the consumption of energy-dense soft drinks between meals.

Physical activity is good for health. According to WHO, insufficient physical activity is responsible for 6% of all deaths worldwide and physical inactivity is ranked as the fourth leading risk factor for global mortality. The majority of the population (87.0%) reports to be trained, regularly physically active or irregularly active, thereby meeting current recommendations. Overall, 48% (44% of men and 52% of women) report walking at least 30 minutes per day, more than 5 days a week, which means at least 150 minutes walking per week. People aged 65-75 years reported longer walking time (68% with at least 150 minutes per week) than the other age groups. There were little differences across language regions.

Prolonged sitting time increases cardio-metabolic risk and all-cause mortality. One third of the population reported a sitting time higher than 8h30 per day, which reflects a high level of sedentarity. People with tertiary education level more frequently reported prolonged sitting time than people with other educational levels.

As highlight in **chapter 5**, menuCH provides important new information on overweight, obesity and waist circumference based on measured data, across the three main language regions of Switzerland. Novel data on cooking and eating habits provide interesting insights into age, sex and regional differences that will be important to inform future nutrition policies in Switzerland.

Executive Summary (German)

Kapitel 1 fasst den Kontext der Nationalen Ernährungserhebung resp. des Berichts zusammen.

Bisher konnte man in der Schweiz bei der Entwicklung von gesundheitsbezogenen Strategien und Empfehlungen auf keine national repräsentativen, objektiv gemessenen anthropometrischen Daten sowie Daten zum Ernährungsverhalten zurückgreifen. Die Erkenntnisse der ersten Nationalen Ernährungserhebung bei Erwachsenen (menuCH) ergänzen Informationen aus früheren Erhebungen, wie jener der Schweizer Gesundheitsbefragung und der Schweizer Erfassung des Salzkonsums, um in Strategien zur Förderung der öffentlichen Gesundheit einzufliessen und Gesundheitsfachpersonen in ihrer Tätigkeit zu unterstützen. Dieser Bericht wird die politischen Entscheidungsträger dabei unterstützen Aktionen für die Umsetzung der neuen Ernährungsstrategie (2017-2020) sowie der Nationalen Strategie zur Prävention nichtübertragbarer Krankheiten festzulegen.

Kapitel 2 erläutert das Studiendesign und die Methoden der Nationalen Ernährungserhebung.

Bei menuCH handelt es sich um eine Querschnittserhebung der erwachsenen Bevölkerung im Alter von 18 bis 75 Jahren, die in den drei grössten Sprachregionen der Schweiz wohnen (kurz Deutsch D-CH, Französisch F-CH, Italienisch I-CH). Die Teilnahme an der Erhebung war freiwillig und für Reisekosten/Zeitaufwand wurde eine Pauschalentschädigung ausgerichtet. Die Erhebung war von den zuständigen kantonalen Ethikkommissionen genehmigt; nur Personen, die eine schriftliche Einverständniserklärung unterzeichneten konnten teilnehmen.
Die Stichprobenplanung sah vor, dass insgesamt 2000 unabhängig lebende Personen im Alter von 18 bis 75 Jahren an der Erhebung teilnehmen, und dass diese Stichprobe für die Bevölkerung in den drei grössten Sprachregionen der Schweiz (Deutsch, Französisch, Italienisch) repräsentativ ist, unter Beachtung der regionalen Verteilung von Frauen/Männern und Altersgruppen (sog. Schichtung). Zudem galt es die Ernährungserhebung über die Jahreszeiten und Wochentage gleichmässig verteilt durchzuführen. Die mehrstufige stratifizierte Stichprobenziehung tangierte somit 4’627’878 unabhängig lebende, 18 bis 75 Jahre alte Frauen und Männer, die in den 11 bevölkerungsstärksten Kantonen der sieben sog. Verwaltungsregionen der Schweiz wohnhaft waren.
Die Teilnehmenden füllten zu Hause einen 49-item Fragebogen handschriftlich aus, bevor sie in einem Studienzentrum persönlich befragt wurden. Der Fragebogen war in drei Sprachen verfügbar und erfasste Informationen zu sozio-demographischen Charakteristika, gesundheitlichen Aspekten, Zufriedenheit mit dem Körpergewicht, Kochgewohnheiten aber auch zum Ess- und Bewegungsverhalten (IPAQ-SF).
Körpergewicht, Körpergrösse sowie der Bauchumfang wurden Standardprotokollen folgend gemessen und die korrekte Umsetzung regelmässig kontrolliert. Der Body Mass Index (Körpermassen Index BMI) und der Bauchumfang wurden anhand anerkannter WHO-Definitionen klassiert/beurteilt.
Die deskriptive statistische Auswertung erfolgte nach Gewichtung der Daten und stratifiziert nach Sprachregionen, Geschlecht, Altersgruppen und Bildungsniveau.

Kapitel 3 und 4 ist den Ergebnissen der Befragung und deren Diskussion gewidmet.

Die Netto-Antwortquote lag bei 38%, was nicht sehr hoch ist, aber vergleichbar mit anderen Erhebungen in der Schweiz, die ein persönliches (face-to-face) Interview beinhalten. Die dem Bericht zugrundeliegenden Daten geben höchstwahrscheinlich die Situation der Bevölkerung ohne Schweizer Staatsbürgerschaft unzureichend wieder. Insgesamt haben 87,3% der Bevölkerung ihren Gesundheitszustand als gut bis sehr gut beurteilt, wie das auch die Gesundheitsbefragung zeigte (83%).

Basierend auf den durchgeführten Körpermessungen war der durchschnittliche berechnete BMI der Männer 25.9 kg/m^2 und der Frauen 24.1 kg/m^2, bei nur geringen sprachregionalen Unterschieden. Nach Altersgruppen betrug der BMI 23.5 (18-34 J.), 25.0 (35-49 J.), 25.9 (50-64 J.) und 26.1 (65-75 J.) kg/m^2. Die Prävalenz von Übergewicht/Adipositas betrug bei den Männern 41.6%/13.9%, bei den Frauen 19.7%/11.3%, 31.0%/12.5% in D-CH, 29.9%/12.3% in F-CH und 30.1%/15.6% in I-CH. Die Prävalenz eines Bauchumfangs, der mit einem erhöhten/erheblich erhöhten metabolischen Risiko verbunden ist betrug gesamthaft betrachtet 16.7%/16.5%, für Männer 18.6%/16.4% sowie für Frauen 14.8%/16.6%.

Insgesamt wollten 55% der Frauen und 50% der Männer gerne Gewicht verlieren, aber nur 5% der Bevölkerung war an einer Gewichtszunahme interessiert. Ein stabiles Körpergewicht war eher für die älteren Personen von Bedeutung.

Unter den Ernährungsformen wurde die vegetarische Ernährung von 4.9% der Befragten genannt, gefolgt von einer energiearmen und fettreduzierten Ernährung, die mit Bemühungen zur Gewichtskontrolle zu tun haben könnten.

Insgesamt, gaben 56.4% der Frauen und 38.1% der Männer an, Vitamin- oder Mineralstoffpräparate einzunehmen. Die Häufigkeit war vergleichbar nach Bildungsniveau aber höher bei den älteren Bevölkerungsgruppen. Die Einnahme von Mineralstoffen wurde häufiger genannt als die Einnahme von Vitaminen oder kombinierten Präparaten und scheint vor allem mit zunehmendem Alter an Bedeutung zu gewinnen.

Die Bundesbehörden fördern verschiedene Instrumente und Ernährungskampagnen um die Bevölkerung bei der Umsetzung eines gesundheitsförderlichen Lebensstils zu unterstützen. So haben bereits drei von vier Personen (75%) in der Bevölkerung von der „Schweizer Lebensmittelpyramide“ gehört. Mehr Frauen als Männern, mehr Personen von höherem als von niedrigerem Bildungsstand und viel mehr jüngeren als älteren Personen war die Pyramide bekannt. Zweidrittel der Personen hatten schon von der „5-am-Tag“-Kampagne gehört, dies vor allem in der F-CH und unter Frauen.

Auf die Frage ob sie während einer üblichen Woche zu Hause selbst warme Mahlzeiten zubereiten, gaben 35% resp. 19% der Bevölkerung an, dies am Mittag bzw. Abend nie zu tun. Dabei war ein Unterschied zwischen Männern (45%) und Frauen (25%) zu beobachten, die nie zuhause eine warme Mittagsmahlzeit kochen. Es war zwischen den Altersgruppen ein deutlicher Unterschied festzustellen, was das Kochen zu Mittag bzw. zu Abend angeht. Unabhängig vom Alter gaben rund ein Drittel der Befragten an nie selbst zu Mittag eine warmes Essen zu kochen, aber deutlich weniger junge Personen (15% der 18-34 und 13% der 35-49 jährigen) als ältere Personen (35% der 65-75 jährigen) kochen nie selbst ein warmes Abendessen. Mehr Frauen (50%) als Männer (31%) gaben an viel Zeit (definiert als mindestens 40 Minuten) für das Kochen warmer Mahlzeiten aufzubringen.

Die am häufigsten und regelmässig ausgelassene Mahlzeit ist für 5.2% der Bevölkerung das Frühstück, gefolgt vom Mittagessen (2.2%) und Abendessen (0.6%).

Lebensmitteln und Getränken werden nicht nur zu den drei traditionellen Hauptmahlzeiten (Frühstück, Mittagessen, Abendessen) verzehrt, sondern immer wieder auch zwischendurch. Diese Verzehrsereignisse werden im Englischen als „snack“, im Deutschen als ‘Zwischenmahlzeit’ bezeichnet. Im Bericht verwenden wir, wie in der Literatur vorgeschlagen, den Begriff „Snacking“ wenn es um „den Verzehr einer Zwischenmahlzeit/eines snacks“ geht, unabhängig davon ob „gesundheitsförderliche Produkte“ oder „snack foods“ konsumiert werden. Die Mehrheit (>80%) der Bevölkerung nehmen

mindestens eine Zwischenmahlzeit pro Tag zu sich, unter der Woche wie am Wochenende. Softdrinks werden von 20% der Bevölkerung zu Zwischenmahlzeiten (zwischendurch) getrunken und das von mehr Männern (27%) als Frauen (12%) und am häufigsten in der jüngsten Altersgruppe (32% (18-34 J.), 18% (35-49 J.), 14% (50-64 J.), 8% (65-75 J.)). Diese Resultate verdeutlichen, dass der zwischendurch Konsum energiereicher Softdrinks vor allem bei jungen Menschen zu senken ist.

Bewegung ist gesundheitsförderlich. Gemäss WHO ist ein Mangel an körperlicher Aktivität für 6% der Todesfälle weltweit verantwortlich und körperliche Inaktivität belegt Platz vier auf der Liste der Risikofaktoren für die Sterblichkeit weltweit. Ein Grossteil (87%) der Bevölkerung gab an zu trainieren resp. regelmässig oder unregelmässig körperlich aktiv zu sein, womit sie die geltenden Schweizer Bewegungsempfehlungen erfüllten. Insgesamt berichteten 48% der Bevölkerung (Männer 44%, Frauen 52%) mindestens während 30 Minuten pro Tag an mehr als fünf Tagen pro Woche zu Fuss zu gehen, also mind. 150 Minuten pro Woche. Personen im Alter von 65-75 Jahren verbrachten mehr Zeit mit Laufen/Spazieren gehen (68% für mind. 150 Minuten pro Woche) als die jüngeren Personen. Zwischen den Sprachregionen gab es keine nennenswerten Unterschiede.

Dauerhaftes Sitzen ist schlecht für Herzkreislauf und Stoffwechsel und trägt zu einer erhöhten Gesamtmortalität bei. Ein Drittel der Bevölkerung gab an täglich mehr als 8 Stunden und 30 Minuten sitzend zu verbringen, was einem hohen Niveau der Sedentarität entspricht. Personen mit höherer Bildung (Tertiärstufe) verbrachten längere Zeiten ohne Bewegung/sitzend als Personen der anderen Bildungsstufen.

Kapitel 5 fasst abschliessend zusammen, dass menuCH wichtige neue Informationen zur Prävalenz von Übergewicht und Adipositas in den drei grössten Sprachregionen der Schweiz liefert, die auf Grundlage von objektiven Messungen des Gewichts, der Grösse und des Bauchumfangs basieren. Erstmals stehen auch Informationen zu den Koch- und Essgewohnheiten der Bevölkerung zur Verfügung. Es sind interessante Unterschiede zwischen Frauen und Männern, Altersgruppen und Sprachregionen erkennbar, die in Hinblick auf die Formulierung zukünftiger Ernährungsstrategien wichtig sind.

Executive Summary (French)

Le chapitre 1 présente une courte introduction du contexte. Jusqu'à ce jour, la Suisse ne disposait pas de données nationales représentatives de la population sur des données anthropométriques mesurées, ainsi que sur les comportements alimentaires pour établir des stratégies de santé et des recommandations. Les données de la première Enquête Nationale sur l'Alimentation (menuCH) complètent désormais celles d'enquêtes précédentes, comme les Enquêtes Suisses sur la Santé ou l'Enquête Suisse sur le Sel, pour informer les politiques de santé publique et les professionnels de la santé. Ce rapport aidera les responsables de politiques publiques à préparer les actions pour la nouvelle stratégie nationale de nutrition (2017-2020) ainsi que pour la stratégie nationale de prévention des maladies non-transmissibles (2017-2024).

Le chapitre 2 présente le design d'étude et les méthodes. menuCH est une enquête de population chez les adultes âgés de 18 à 75 ans résidant dans les trois régions linguistiques principales de la Suisse (Suisse allemande, Suisse romande et Suisse italienne). La participation était volontaire et les coûts de transport/temps consacré à l'étude ont été compensés. L'enquête a été approuvée par les commissions d'éthique régionales et les participants ont signé un consentement écrit éclairé. Le cadre d'échantillonnage a été conçu pour viser une taille d'échantillon final de 2000 individus âgés de 18 à 75 ans représentatif des trois régions linguistiques principales de la Suisse (alémanique, romande et italienne), équilibré par rapport à des strates prédéfinies d'âge et de sexe à l'intérieur de chaque région linguistique. L'échantillonnage stratifié en plusieurs étapes cherchait à couvrir une population de 4'627'878 hommes et femmes non-institutionnalisés âgés de 18 à 75 ans résidant dans les 11 cantons les plus peuplés des sept régions administratives de la Suisse. Les participants ont complété un questionnaire papier de 49 items à la maison, avant un entretien face-à-face dans un centre d'étude. Le questionnaire était disponible en trois langues et portait sur des caractéristiques sociodémographiques, des questions sur la santé, la satisfaction par rapport au poids corporel, les façons de cuisiner, les habitudes alimentaires et l'activité physique (IPAQ-SF). Le poids, la taille et le tour de taille ont été mesurés de façon standardisée, avec des contrôles de qualité stricts. L'indice de masse corporelle (IMC) et le tour de taille ont été catégorisés selon les définitions de l'OMS. Des analyses descriptives pondérées ont été faites, en stratifiant par sexe, âge, régions linguistiques et niveau d'éducation.

Les **chapitres 3 et 4** présentent les résultats et la discussion. Le taux de réponse net était de 38%, ce qui n'est pas très élevé, mais néanmoins semblable à d'autres enquêtes incluant des entretiens en face-à-face en Suisse. Les résultats présentés dans ce rapport ne reflètent probablement pas de façon adéquate la situation parmi les personnes de nationalité étrangère. Globalement, 87.3% de la population ciblée perçoit son état de santé général comme étant bon ou très bon, ce qui correspond aux résultats de l'Enquête Suisse sur la Santé 2012 (83%).

L'IMC moyen, basé sur des mesures, était de 25.9 kg/m^2 chez les hommes et de 24.1 kg/m^2 chez les femmes, avec peu de différences interrégionales. L'IMC moyen était de 23.5, 25.0, 25.9 et 26.1 kg/m^2 dans les tranches d'âge de 18-34, 35-49, 50-64 et 65-75 ans, respectivement. La prévalence de surpoids et l'obésité était de 41.6% et 13.9% chez les hommes, 19.7% et 11.3% chez les femmes, 31.0% et 12.5% en Suisse alémanique, 29.9% et12.3% en Suisse romande, et 30.1% et 15.6% en Suisse italienne, respectivement. La prévalence d'un tour de taille à risque cardiométabolique élevé, et très élevé, était

de 16.7% et 16.5% globalement, 18.6% et 16.4% chez les hommes ainsi que 14.8% et 16.6% chez les femmes, respectivement.

Globalement, 55% des femmes et 50% des hommes souhaitent perdre du poids, alors que seul 5% de la population souhaite prendre du poids. Le maintien du poids devient de plus en plus important avec l'âge. Globalement, un régime végétarien est la forme la plus fréquente de régime spécial rapporté (4.9%) par les participants, suivi du régime de restriction calorique ou pauvre en graisse, ce qui peut refléter des tentatives de contrôle du poids corporel.

Une proportion substantielle de la population (56.4% des femmes et 38.1% des hommes) a rapporté prendre des suppléments vitaminiques et/ou minéraux, avec des résultats semblables dans tous les niveaux d'éducation et une proportion plus élevée parmi les plus âgés. Les suppléments minéraux sont plus fréquemment rapportés que les suppléments en vitamines, ou les produits mixtes.

Les autorités fédérales font la promotion de différents outils et lancent des campagnes de nutrition pour soutenir la population à adopter des styles de vie bons pour la santé. Trois personnes sur 4 dans la population ont entendu parler de la pyramide alimentaire suisse. La connaissance cette pyramide était plus élevée chez les femmes que chez les hommes, chez les personnes avec un niveau d'éducation élevé que chez celles avec un autre niveau d'éducation, chez les jeunes que chez les plus âgés. Deux tiers des personnes connaissaient la campagne «cinq par jour», notamment en Suisse romande et parmi les femmes.

Dans la population, 35% des personnes rapportent ne jamais cuisiner habituellement un repas chaud par eux-mêmes à midi, alors que cette proportion atteint 19% pour le repas du soir. Une plus grande proportion d'hommes (45%) que de femmes (25%) rapportent ne jamais cuisiner un repas chaud à midi. Une grande différence entre repas de midi et du soir a été observée à travers les tranches d'âge. Un tiers de la population ne cuisine jamais de repas chaud à midi, dans toutes les tranches d'âge, alors que moins de jeunes que de plus âgés ne cuisinent jamais de repas chaud le soir (15% des 18-34 ans et 13% des 35-49 ans vs 35% des 65-75 ans). Plus de femmes que d'hommes rapportent passer un long temps (c.à.d. >40 minutes) à cuisiner des repas chauds (50% vs 31%, respectivement). Le repas le plus fréquemment sauté est le petit déjeuner (5.2%), suivi du repas de midi (2.2%) et de celui du soir (0.6%).

Les personnes consomment des aliments (y compris des boissons) durant les repas traditionnels du petit déjeuner, du repas de midi et de celui du soir, mais aussi en dehors de ces repas. Ces consommations alimentaires entre les repas sont appelées «snacks» (en allemand 'Zwischenmahlzeiten') et nous utilisons le terme de "snacking" quelle que soit la nature «saine» ou pas de l'aliment (ou de la boisson) consommé. La majorité de la population (>80%) prend un snack au moins une fois par jour (sans considérer les boissons), avec des comportements semblables durant les jours de semaine ou du week-end. Pour les boissons sucrées, 20% en consomment entre les repas, avec une plus grande prévalence chez les hommes (27%) que chez les femmes (12%), et avec une forte diminution avec l'âge (32%, 18%, 14%, 8% pour les tranches d'âge 18-34, 35-49, 50—64 et 65-75 ans, respectivement). Ces résultats soulignent l'importance de cibler les jeunes pour diminuer la consommation de boissons sucrées denses en énergie entre les repas.

L'activité physique est bonne pour la santé. Selon l'OMS, une activité physique insuffisante est responsable de 6% des décès dans le monde et l'inactivité physique représente le quatrième facteur de risque pour la mortalité globale. La majorité de la population (87%) rapporte être entraînée,

régulièrement physiquement active ou irrégulièrement active, remplissant ainsi les recommandations actuelles. Globalement, 48% (44% des hommes et 52% des femmes) rapporte marcher au moins 30 minutes par jour, plus de 5 jours par semaine, ce qui correspond à plus de 150 minutes de marche par semaine. Les personnes âgée de 65-75 ans rapportent marcher plus longtemps que les autres groupes d'âge (68% rapporte au moins 150 minutes par semaine). Peu de différences interrégionales ont été détectées.

Un temps prolongé en position assise augmente le risque cardiométabolique et la mortalité totale. Un tiers de la population rapporte passer plus de 8h30 par jour en position assise, ce qui reflète un haut niveau de sédentarité. Les personnes avec un niveau d'éducation tertiaire rapportent plus fréquemment que les personnes avec un autre niveau d'éducation un temps prolongé en position assise.

Comme évoqué dans le **chapitre 5**, menuCH apporte de nouvelles informations importantes sur le surpoids, l'obésité et le tour de taille, sur la base de données mesurées dans les trois régions linguistiques principales de la Suisse. De nouvelles données sur la façon de cuisiner et les habitudes alimentaires apportent des renseignements intéressants sur les différences entre les groupes d'âge, le sexe et les régions linguistiques qui seront importants pour mettre en place les futures politiques nutritionnelles en Suisse.

Executive Summary (Italian)

Il **Capitolo 1** presenta una breve introduzione del contesto dello studio. Finora, nella concezione di strategie di salute pubblica e nella determinazione di linee guida e linee la Svizzera non ha potuto fare affidamento su dati nazionali rappresentativi della popolazione e su dati antropometrici oggettivamente misurati, di alimentazione e di attività fisica. I dati del primo Sondaggio Nazionale sulla Nutrizione per adulti (menuCH) sono ora disponibili e completano l'informazione ottenuta da sondaggi precedenti come l'Indagine sulla salute in Svizzera e l'indagine svizzera sul sale, nell'informare le politiche sanitarie pubbliche e gli operatori sanitari. Questo rapporto aiuterà i responsabili politici nella preparazione della nuova strategia di nutrizione (2017-2020), così come nella preparazione della la strategia nazionale per la prevenzione delle malattie non trasmissibili (2017-2024).

Il **Capitolo 2** presenta il disegno e i metodi dello studio menuCH. MenuCH è un'indagine popolazionale trasversale di adulti tra i 18 e i 75 anni di età residenti nelle tre principali regioni linguistiche della Svizzera (tedesco D-CH, Francese F-CH, Italiano I-CH). La partecipazione era su base volontaria e le spese di viaggio come il tempo investito sono stati ricompensati. L'indagine è stata approvata dalle rispettive commissioni etiche regionali e i partecipanti hanno firmato un consenso informato scritto. Il campionamento è stato stabilito in modo da fornire un campione finale di 2000 individui non istituzionalizzati di età compresa tra 18 e 75 anni, che fosse rappresentativo delle tre principali regioni linguistiche della Svizzera (tedesco, francese e italiano), equilibrato rispetto agli strati di sesso ed età predefiniti all'interno di ogni regione linguistica, nonché rappresentativo delle diverse stagioni e giorni della settimana. Il campionamento stratificato a stadi multipli rappresenta 4'627'878 uomini e donne non istituzionalizzati tra i 18 ei 75 anni de età che risiedevano negli 11 cantoni più popolosi delle sette regioni amministrative della Svizzera. I partecipanti hanno completato a domicilio un questionario scritto di 49 punti, prima di partecipare ad un'intervista faccia a faccia nel centro di studio. Il questionario era disponibile in tre lingue e ha fornito informazioni sulle caratteristiche socio-demografiche, sulle questioni relative alla salute, la soddisfazione riguardo al peso corporeo, le abitudini culinarie, nonché sui comportamenti alimentari e l'attività fisica (IPAQ-SF). Il peso corporeo, l'altezza e la circonferenza della vita sono stati misurati utilizzando procedure standardizzate, con rigorose procedure di controllo. L'Indice di massa corporea (BMI) e la circonferenza della vita sono stati categorizzati seguendo le raccomandazioni dell'OMS. L'analisi statistica descrittiva stratificata ponderata è stata eseguita considerando le regioni linguistiche, il sesso, l'età e il livelli di istruzione.

I **Capitoli 3 e 4** presentano i risultati e la loro discussione. Il tasso di risposta netto è stato del 38%, che seppur non alto è risultato simile a quello di indagini precedenti che includevano interviste faccia a faccia. E' probabile che i dati inclusi in questo rapporto non riflettano adeguatamente la situazione dei cittadini non Svizzeri. In generale, l'87.3 % della popolazione ha dichiarato di percepire il proprio stato di salute come buono o molto buono, in linea con i risultati dell'Indagine Svizzera sulla Salute del 2012 (83%). Il BMI medio misurato era di 25,9 kg / m2 per gli uomini e di 24,1 kg / m2 per le donne, con piccole differenze tra le regioni linguistiche. Il BMI medio misurato era di 23.5, 25.0, 25.9 e 26.1 kg/m2 nelle categorie d'età 18-34, 35-49, 50-64 e 65-75, rispettivamente. La prevalenza di sovrappeso e obesità, in base ai dati misurati, è stata del 41,6% e del 13,9% negli uomini, del 19,7% e del 11,3% nelle donne, del 31,0% e del 12,5% nella regione di lingua tedesca, del 29,9% e 12,3% nella regione linguistica francese , e del 30,1% e 15,6% nella regione di lingua italiana, rispettivamente. La prevalenza di circonferenza della vita ad aumentato e altamente aumentato rischio metabolico è stata del 16,7% e del

16,5% in totale, del 18,6% e del 16,4% negli uomini e del 14,8% e del 16,6% nelle donne, rispettivamente. Nel complesso, il 55% delle donne e il 50% degli uomini vorrebbero perdere peso, mentre solo il 5% della popolazione vorrebbe aumentare di peso. Il mantenimento del peso diventa più importante con l'aumentare dell'età. Nel complesso, la dieta vegetariana è la forma più diffusa di dieta speciale, seguita da diete di restrizione di energia e di grassi, che possono essere correlati a sforzi costanti di controllo del peso costante. Una parte consistente della popolazione (56,4% delle donne e il 38,1% degli uomini) riferisce di prendere integratori vitaminici o minerali, con percentuali simili nei diversi livelli di istruzione e in proporzione maggiore nelle fasce di età più avanzate. Gli integratori minerali sono più frequentemente consumati di quelli vitaminici o di prodotti combinati, e il loro consumo sembra aumentare rapidamente con l'età.

Le autorità federali promuovono diversi strumenti e hanno implementato diverse campagne di nutrizione per sostenere la popolazione nell'adozione di uno stile di vita sano. Nel complesso, tre persone su quattro nella popolazione avevano già sentito parlare della "piramide alimentare svizzera", con proporzioni maggiori tra le donne rispetto agli uomini, e più alte nei partecipanti più istruiti che nelle persone con basso livello di istruzione. La consapevolezza riguardo alla piramide alimentare era molto più alta tra i giovani che tra le persone anziane. Due terzi dei partecipanti era a conoscenza della campagna "5 al giorno", in particolare nella regione di lingua francese e tra le donne.

Quando interrogati riguardo al cucinare piatti caldi a casa durante una settimana normale, il 35% dei partecipanti ha riportato di non cucinare mai personalmente un piatto caldo per pranzo e il 19% per cena. Abbiamo osservato differenze di genere, con il 45% degli uomini contro il 25% delle donne che hanno riportato di non cucinare mai un pranzo caldo per sé stessi a casa. Grandi differenze sono state osservate tra pranzi e cene rispetto ai gruppi di età. Circa un terzo dei partecipanti hanno riportato di non preparare mai un pranzo caldo a casa indipendentemente dall'età, ma molti meno giovani (15% per il gruppo 18-34 anni, 13% per i 35-49 anni) che persone anziane (35% tra i 65-75 anni di età) hanno riferito di non cucinare mai una cena calda a casa loro. Molte più donne che uomini hanno riferito di spendere molto tempo (definito come più di 40 minuti) a cucinare piatti caldi (50% contro 31% rispettivamente). Il pasto più frequentemente saltato è risultato essere la colazione (5.2% della popolazione), seguito dal pranzo (2.2%) e dalla cena (0.6%).

Le persone consumano alimenti (bevande incluse) durante i pasti tradizionali di colazione, pranzo e cena, ma anche al di fuori e tra questi pasti. I momenti in cui si consumano cibo o bevande al di fuori dei pasti tradizionali sono chiamati snacks ('Zwischenmahlzeit' in tedesco, "spuntini" in italiano), e come suggerito abbiamo usato il termine "snacking" o fare uno spuntino quando ci riferiamo "all'atto di fare uno spuntino, indipendentemente dal fatto che lo spuntino sia sano o no". La maggioranza della popolazione fa uno spuntino almeno una volta durante la giornata (>80%), con proporzioni simili durante la settimana o il fine settimana.

Riguardo alle bevande analcoliche, il 20% nel complesso ha riferito di consumarle durante gli spuntini, con una prevalenza molto più alta negli uomini (27%) rispetto alle donne (12%) e con una diminuzione ripida correlata all'età (32%, 18%, 14%, 8% per gli 18-34, 35-49, 50-64 e 65-75 anni, rispettivamente). Questi risultati sottolineano l'importanza di puntare sui giovani per diminuire il consumo di bevande analcoliche ad alta densità energetica tra i pasti.

L'attività fisica fa bene alla salute. Secondo l'OMS, la scarsa attività fisica è responsabile del 6% di tutti i decessi in tutto il mondo e l'inattività fisica è classificata come il quarto fattore di rischio per la mortalità

globale. La maggior parte della popolazione (87,0%) riferisce di essere allenata o di praticare regolarmente o irregolarmente attività fisica, in linea con le racommandazione attuale. Nel complesso, il 48% (44% degli uomini e 52% delle donne) ha dichiarato di camminare almeno 30 minuti al giorno, per più di 5 giorni a settimana, il che significa almeno 150 minuti a settimana. Le persone di età compresa tra i 65 e i 75 anni hanno riportato di camminare per più tempo (68% riferiscono almeno 150 minuti a settimana) che le persone di altre fasce di età. C'erano differenze minori tra le regioni linguistiche.

Passare molto tempo seduti aumenta il rischio cardiometabolico e di mortalità. Un terzo della popolazione riferische di passare più di 8 ore e trenta minuti al giorno in posizione seduta, il che riflette un elevato livello di sedentarietà. Le persone con livello di istruzione universitaria passano più tempo in posizione seduta rispetto alle persone con altri livelli di istruzione.

Come descritto nel **capitolo 5**, menuCH fornisce nuove importanti informazioni sul sovrappeso, l'obesità e la circonferenza della vita sulla base di dati misurati, nelle tre principali regioni linguistiche della Svizzera. Nuovi dati sulle abitudini alimentari e culinarie forniscono interessanti spunti di riflessione riguardo l'età, il sesso e le differenze regionali che saranno importanti per informare le future politiche di nutrizione in Svizzera.

1. INTRODUCTION

Until recently, Switzerland could not rely on national representative food consumption data and information on eating behaviors when establishing health related strategies and guidelines. Data needs of the Swiss authorities, namely the Federal Office of Public Health (FOPH) and the Food Safety and Veterinary Office (FSVO), and of various stakeholders, such as non-governmental institutions (e.g. the Swiss Society for Nutrition SSN), of researchers, health professionals and food industry were identified when establishing the 6th Swiss Nutrition Report (1) and are reflected in the Swiss Nutrition Policy 2013-2016 (2). All players require an up-to-date and sound body of national representative nutrition data for their activities related to food safety, prevention, health promotion and health care. Aiming for monitoring, the first National Nutrition Survey (menuCH) was developed and launched for adults to complement already existing national surveys such as the Swiss Health Survey (3). This report will help policy makers in preparing actions for the new nutrition strategy (2017-2020) as well as the strategy of non-communicable diseases prevention (2017-2024) (4); it further serves FOPH to update indicators of their monitoring system on diet and physical activity MOSEB (5).

2. STUDY DESIGN AND METHODS

The first National Nutrition Survey (hereafter called menuCH) was a cross-sectional population survey among 18 to 75 years old adults residing in the three main linguistic regions of Switzerland (short German D-CH, French F-CH, Italian I-CH). Participation was voluntary and travel costs/time investment compensated. Food consumption and subsequently nutrient intake is known for inter- and intra- (day-to-day) individual variations, and estimating usual intake of individuals is methodologically challenging (6). Therefore, individual food consumption data were collected twice, and the survey was conducted from January 2014 till February 2015 covering food intakes during all seasons, on weekdays as well as weekend days.

Methods were harmonized internationally to allow basic data comparisons. In particular, the 2009 Guidance of the European Food Safety Authority (EFSA) on General principles for the collection of national food consumption data in view of a pan European dietary survey (7) was largely followed. This requested at least one dietary face-to-face and one phone interview per person and the collection of additional information on socio-demographic/economic and health-related information including physical activity. For this reason a Swiss-wide system of study centers was installed in 10 cities across the three linguistic regions, where 15 repeatedly trained dieticians worked as interviewers. Interviews were carried out in German, French or Italian.

2.1. Sampling frame and sample weighting

The sampling frame was established in collaboration with the Federal Statistics Office (FSO) to provide a final sample/data set of 2000 free-living individuals aged 18 to 75 years that is

- representative of the three main linguistic regions of Switzerland (French, German and Italian);
- balanced with respect to the predefined sex and age strata within each linguistic region;

- balanced across seasons (similar number of participants in winter, spring, summer and autumn) and across days of the week (aiming at a similar number of interviews for each day of the week).

The multiple step stratified sampling thus targeted 4’627’878 free-living 18-75 years old women and men residing in the 11 most populous cantons (Aargau, Basel Stadt und Land, Bern, Jura, Neuchatel, Luzern, Zurich, St. Gallen, Vaud, Genève, and Ticino) of the seven administrative regions of Switzerland. FSO provided five representative sub-samples based on the sample frame for person and household surveys (Stichprobenrahmen für Personen- und Haushalterhebungen SRPH) which is relying on cantonal and community registry data. These person data were matched with phone numbers provided by phone companies, with an approximate 75% matching rate (8). Considering menuCH-pilot survey experience (13% gross and 28% net response rate), a gross sample of almost 14’000 individuals was contacted. A 38% net response rate (2085 responders/5496 net sample) was achieved.

Since not all people residing in the above mentioned cantons had the same chance to become part of the sample, a three-stage model for weighting was established and applied in the data analysis presented in this report (9). Thus, the survey results were extrapolated to the target population, representing about 4’627’878 free-living women and men aged 18-75 years as explained with Figure 1.

n is the **number** of men (M), women (W) and in total who provided specific data

N is the corresponding **number** of men and women in the **target population**. Data shown in tables or figures were extrapolated, that is weighting factors were applied. In this example, 946 men and 1139 women represent 2’305141 men (49.9%) and 2’316’876 women (50.1%) of the target population, respectively.

Sex	W	M	All
n	1139	946	2085
N	2’316’876	2’305’141	4’622’018
%	50.1	49.9	100

Figure 1 Weighting and calibrating for target population representative data.

2.2. Questionnaire - Participant characteristics & Eating and Physical activity behaviors

Participants completed a 49-item paper-pencil questionnaire at home, prior to their face-to-face interview in the study center. The questionnaire was available in three languages and provided information on socio-demographic and -economic characteristics, health-related issues including reported body weight and height, as well as on eating and physical activity behaviors.

The section on eating and physical activity behavior was developed by FOPH and FSVO and pre-tested for length and comprehensibility using cognitive interviews (10, 11). For health related questions reference was made to questions of the Swiss Health Survey (12) whereas most diet related questions (e.g. knowledge) were newly developed. For physical activity, the short version of the IPAQ – International Physical Activity Questionnaire was included (13). The behavior part was amended by a selection of socio-economic and -demographic questions from the Swiss Health Survey (12), with minor changes applied due to experiences from regional surveys in Switzerland (CoLaus (14, 15) and Bus santé studies(16, 17)).

2.3. Anthropometrics

menuCH provides objectively measured anthropometric measures, complemented by self-reported body weight and height from the questionnaire.

Interviewers were intensively trained to measure participants' body weight, body height as well as waist and hip circumference in a standardized way following WHO-MONICA protocol (18). No measures were taken for pregnant or breastfeeding women, immobile people or people refusing a specific measure. People removed shoes and heavy outer garments and emptied pockets prior to measurements.

Body weight and height were measured to the nearest 0.1 kg and 0.1 cm using a calibrated electronic scale (Seca 701, Hamburg, Germany) equipped with a stadiometer (Seca 220 telescopic rod). Prior to data analysis, 0.8 kg / 1.2 kg were subtracted from registered body weight for women / men to account for average weight of light clothing/garments (19). For weight status evaluation Body Mass Index (BMI) was calculated (body weight divided by height squared (kg/m^2)) and categorized according to WHO (20): underweight below 18.50 kg/m^2; normal weight 18.50-24.99 kg/m^2; overweight 25.00-29.99 and obesity 30.00 and more kg/m^2.

Waist circumference (WC) and hip circumference were measured using a calibrated Gulick unstretchable tape, equipped with a dynamometer (North Coast Medical, CA, USA). Each tape was individually calibrated for accurate and reproducible body measurements. WC was measured halfway between the iliac crest and the lower rib and was taken directly on the skin. Hip circumference was the maximum circumference over the buttocks in centimeters (to the nearest 0.0 or 0.5 cm) and was taken while participants were wearing pants or skirts. If the latter were thick, the dietician pressed more on the Gulick tape. The measure was taken three times in a row, to the nearest 0.1 centimeter and the mean per person was considered. According to WHO (21) participants were classified to be at risk for metabolic complications according to the WC cut-offs or subgroups, for men (M) and women (W) respectively: (i) no risk ≤ 94 cm (M), ≤ 80 cm (W); (ii) increased risk 94.1-101.9 (M), 80.1-87.9 (W); (iii) substantially increased risk ≥ 102 cm (M), ≥ 88 cm (W). To note, BMI and WC are used as indicators of obesity and abdominal overweight, respectively (20, 21).

2.4. Food consumption

Individual food intake was assessed conducting two non-consecutive 24-Hour Dietary Recalls (24HDR). The first face-to-face, the second by phone two to six weeks later. To start, participants provided general information about their diet; then they were asked and probed by the interviewer to remember and report kind and amount of all foods and beverages they consumed between waking time on the preceding day and waking time on the interview day. For in-depth information about the method see (22). The structured multiple–pass computer-assisted interview was conducted using the software GloboDiet® (GD, formerly EPIC-Soft®) (23, 24) which was complemented with a comprehensive picture book (25) and a set of real dishes to support survey participants in quantifying amounts of consumed foods. Both, software and picture book had been adapted for the Swiss specific food market and requirements of FSVO/FOPH and other stakeholders; they were provided in German, French and Italian.

In the present report no food consumption data are presented, only information on reported special diets is used.

2.5. Statistical analysis

For the present report, descriptive statistics were performed using Stata 14.1 (StataCorp. 2015, Stata Statistical Software: Release 14. College Station, TX, USA). All the analyses have been corrected by sample weights provided within the study and accounting for non-response and national population representativeness. The presented results are therefore population weighted data (see above, (9)). We present descriptive results and only tested for statistical significance for data presented in Tables 1 and 2, in which we compared the distribution between men and women using a Pearson's Chi-square test. In tables and figures continuous variables are shown as the mean or median (range). Categorical data are presented in absolute and relative (%) frequencies.

Physical activity was analyzed applying the official IPAQ data processing and analysis guidelines (26). The method by Bland and Altman (27) was used to evaluate how well BMI calculated based on reported and measured body weight and height agreed.

We present stratified analyses by linguistic regions, sex, age groups, and educational levels. We considered three educational levels: primary (compulsory schooling terminated), secondary (baccalaureate, apprenticeship) and tertiary level (higher education requesting baccalaureate). The analysis of certain eating behavior variables requested to build best informed categories. For example, a long time to cook hot meals was defined as spending more than 40 minutes, which is the median duration. Where applicable, the definition is explained in the result section/tables.

We compare selected demographic characteristic of the menuCH population with telephone interview data from the Swiss Health Survey (SHS) 2012 (Federal Statistical Office, www.admin.bfs.ch). The initial SHS 2012 sample size included 21'597 individuals. After exclusion of the age categories not corresponding to those of MenuCH, the sample reduced to 18'991 observations. We then selected only the participants for which we had information on all the characteristics of interest (linguistic region, educational level, and smoking habits). After pairwise exclusion of missing values, we had a final sample of n=18'894 (n=5 had no answers on smoking habits, n=92 on education).

3. RESULTS

3.1. Description of the male and female population

Selected socio-demographic and-economic as well as health-related characteristics of the survey population in menuCH are presented in Table 1 and Figure 2. Some of the characteristics/subgroups were used for stratification purposes (see below). Table 2 presents similar socio-demographic information in the Swiss Household Survey 2012 for comparative purposes. In menuCH, 2085 individuals, 1139 women and 946 men, were interviewed, who represented 4'622'018 people (50% men and women each) of the target population, as described above.

As shown in Table 1, men and women were comparably represented in the three linguistic regions and in all age groups. The distribution across age groups is very similar to the one observed in the Swiss Health Survey 2012 (Table 2).

Table 1 Socio-demographic and health related characteristics of the survey population (menuCH).

Characteristics	TOTAL	WOMEN	MEN	P-value (χ^2)
Total n (survey)	n= 2085	n = 1139	n = 946	
Total N (%)	4’622’018 (100.0)	2’316’876 (50.1)	2’305’141 (49.9)	
Linguistic region*				
D-CH	3‘199‘861 (69.2)	1‘560‘917 (67.4)	1‘638‘944 (71.1)	0.17
F-CH	1‘165‘232 (25.2)	622‘326 (26.9)	542‘906 (23.6)	
I-CH	256‘925 (5.6)	133‘633 (5.8)	123‘291 (5.4)	
Age group (years)				
18-34	1‘319‘373 (28.6)	679‘029 (29.3)	640‘344 (27.8)	0.42
35-49	1‘407‘444 (30.5)	730‘868 (31.6)	676‘576 (29.4)	
50-64	1‘245‘315 (26.9)	607‘813 (26.2)	637‘502 (27.7)	
65-75	649‘886 (14.1)	299‘166 (12.9)	350‘719 (15.2)	
Educational level				
Primary	211‘650 (4.6)	103‘678 (4.5)	107‘971 (4.7)	0.03
Secondary	1‘974‘347 (42.8)	1‘066‘078 (46.1)	908‘269 (39.5)	
Tertiary	2‘427‘288 (52.6)	1‘143‘836 (49.4)	1‘283‘452 (55.8)	
Smoking categories				
Never smoker	1‘970‘157 (42.7)	1‘143‘605 (49.4)	826‘552 (34.0)	<0.0001
Former smoker <100 cig eq.	366‘421 (8.0)	209‘405 (9.1)	157‘016 (6.8)	
Former smoker >100 cig eq.	1‘178‘255 (25.6)	579‘271 (25.0)	598‘984 (26.1)	
Social smoker	412‘666 (9.0)	144‘798 (6.3)	267‘868 (11.7)	
Daily smoker	682‘740 (14.8)	236‘513 (10.2)	446‘227 (19.4)	

*D-CH=cantons of BE, BL, BS, LU, ZH, SG, AG; F-CH=cantons of GE, VD, NE, JU; I-CH=canton of TI. P values are from a chi-square test. Population weighted data (percentages). n=sample size for the survey. N=size of the corresponding target population.

Table 2. Socio-demographic and health related characteristics in the Swiss Health Survey 2012.

Characteristics	TOTAL	WOMEN	MEN	P-value (χ^2)
Total n (survey)	n= 18‘894	n = 9‘852	n = 9‘042	
Total N (%)	5'950'280 (100.0)	2'978'565 (50.1)	2971715 (49.9)	
Linguistic region				
D-CH	4‘240‘241 (71.3)	2‘088‘497 (70.1)	2‘151‘744 (72.4)	0.004
F-CH	1‘439‘469 (24.2)	746’046 (25.0)	693’423 (23.3)	
I-CH	270’570 (4.5)	144’022 (4.8)	126’548 (4.3)	
Age group (years)				
18-34	1’724’119 (29.0)	848’744 (28.5)	875’376 (29.5)	0.07
35-49	1‘880‘699 (31.6)	936‘912 (31.5)	943‘788 (31.8)	
50-64	1‘518‘763 (25.5)	754‘355 (25.3)	764‘408 (25.7)	
65-75	826‘699 (13.9)	438‘555 (14.7)	388‘144 (13.1)	
Educational level				
Primary	840‘548.8 (14.1)	470‘836 (15.8)	369‘713 (12.4)	<0.0001
Secondary	325‘6018 (54.7)	1‘778‘258 (59.7)	1‘477‘761 (49.7)	
Tertiary	1‘853‘713 (31.2)	729‘472 (24.5)	1‘124‘241 (37.8)	
Smoking categories				
Never smoker	2‘874‘239 (48.3)	1‘620‘111 (54.4)	1‘254‘128 (42.2)	<0.0001
Former smoker	1‘274‘790 (21.4)	578‘088 (19.4)	696‘703 (23.4)	
Social smoker	487‘960 (8.2)	198‘090 (6.7)	289‘871 (9.8)	
Daily smoker	1‘313‘290 (22.1)	582’277 (19.5)	731’014 (24.6)	

Weighted analyses of Swiss Health Survey 2012, restricting the age group 18 to 75 years. The analysis was based on interview data.

The survey population reported a high level of education (Table 1). Less than 5% of the population report to have achieved compulsory education or less, which is substantially less than Swiss Health Survey 2012 with 14% of people with primary education only (Table 2). More than half of the population (53% overall; 56% for men and 49% for women) completed a tertiary education/university level and slightly less people (43% overall; 40% for men and 46% for women) reported a secondary educational level.

Table 1 further shows, that almost 24% of the overall survey population were smoking at time of the interview, with twice as much men (31%) than women (16%) being daily or social smokers. However, a third of people had smoked in the past and 43% had never smoked. Compared to the Swiss Health Survey 2012 population (Table 2) in which 30% were smokers (34% men, 26% women), the female menuCH population may have been a bit more health conscious.

General state of health

Overall 87.3% of the population perceived their general health state as being good or very good, which is slightly higher than what was reported in the SHS 2012 (83%). By contrast with what was reported in the SHS 2012 (84% of men and 81% of women), a larger proportion of women (88.8%) perceived their general health as being good or very good than men (85.8%). We observed regional differences in self-rated general health, with 31.2%, 36.8% and 25.9% of the population from the German-, French- and-Italian-speaking regions perceiving their health as very good, respectively (Figure 2). General health was perceived as very good by 37.0%, 34.3%, 31.5% and 20.2% across the 18-34, 35-49, 50-64 and 65-75 age groups (Figure 2). Such age-related decrease in self-reported general health is of course expected and was also observed in SHS 2012. Although, self-rated general health reflects the individual's perception and not any objectively measured medical state, it is considered as a valid indicator of general health, both at individual and population level.

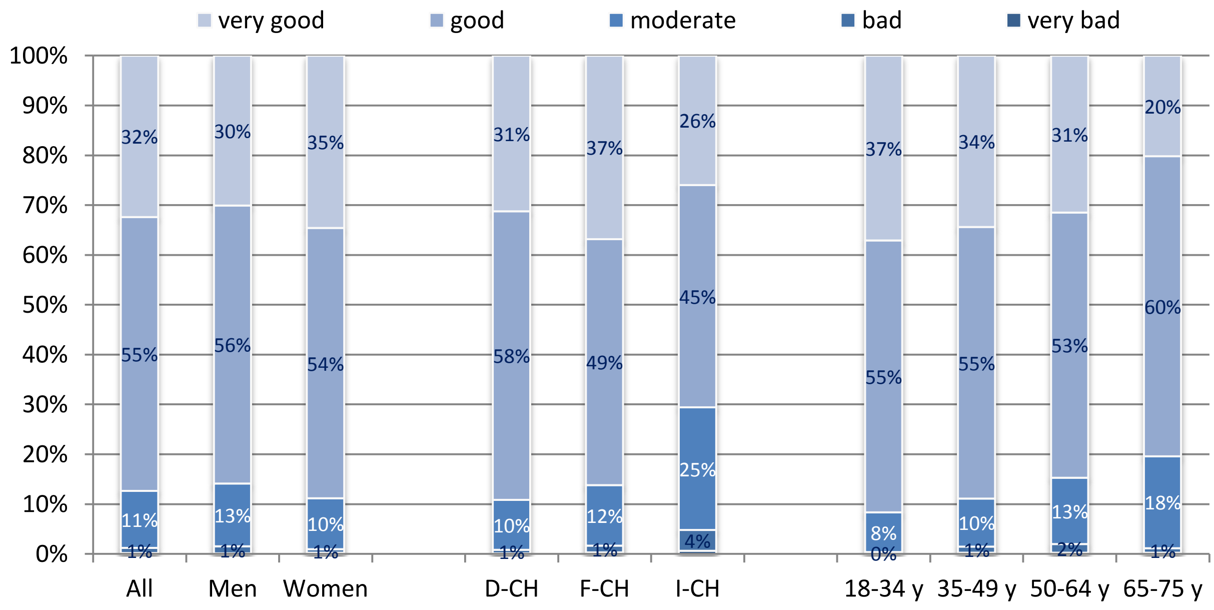

Figure 2 Self-rated health of women and men by linguistic region and age groups (menuCH).

3.2. Body weight status and body image

3.2.1. Measured anthropometric data

Weight status according to BMI (in kg/m^2) calculated from *measured* weight and height, is shown by linguistic region (Figure 3), age groups (Figure 4), educational (Figure 5) and physical activity (Figure 6) levels for all, men and women.

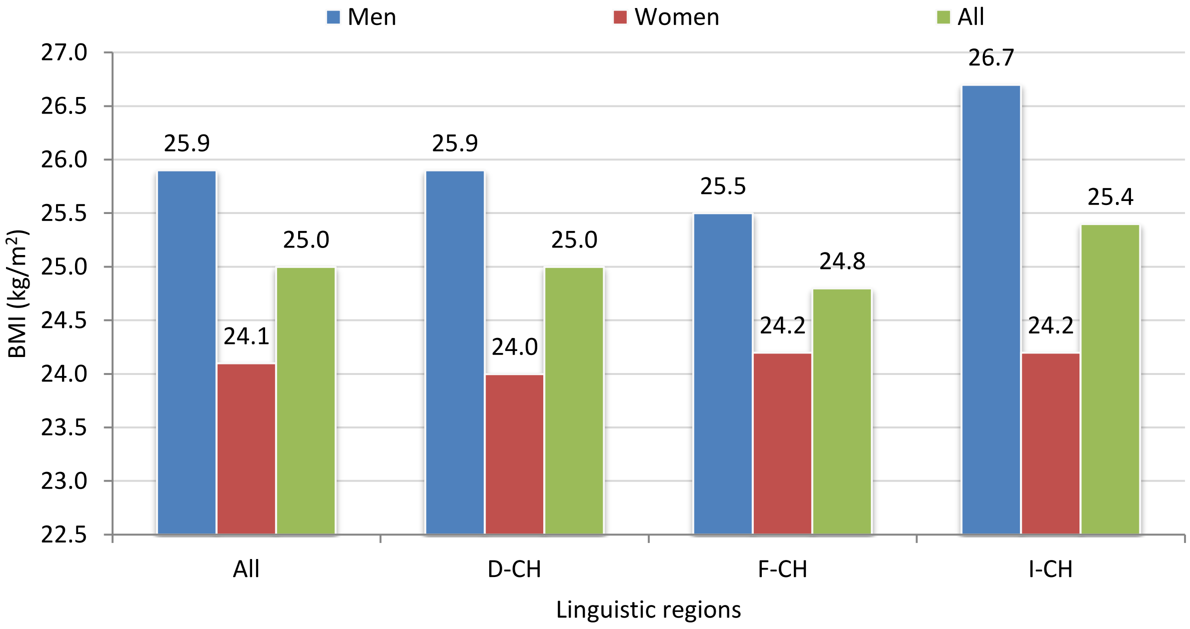

Figure 3 Average body mass index (BMI in kg/m^2; measured weight, height) overall, by sex linguistic region.

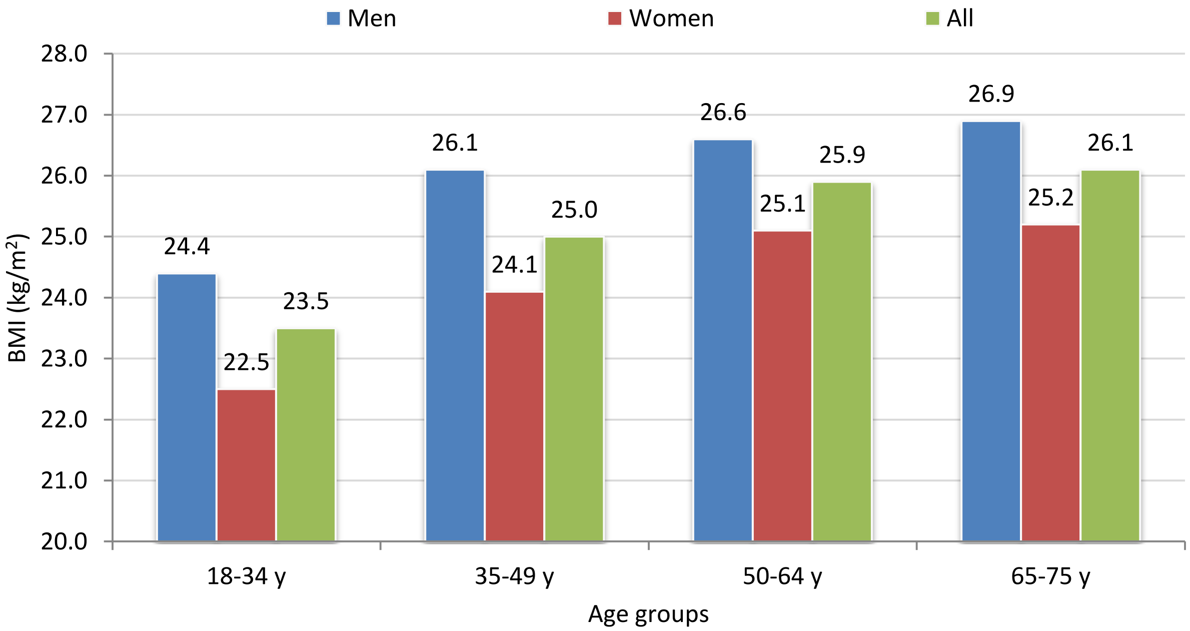

Figure 4 Average body mass index (BMI in kg/m^2; measured weight, height), overall, by sex and age groups.

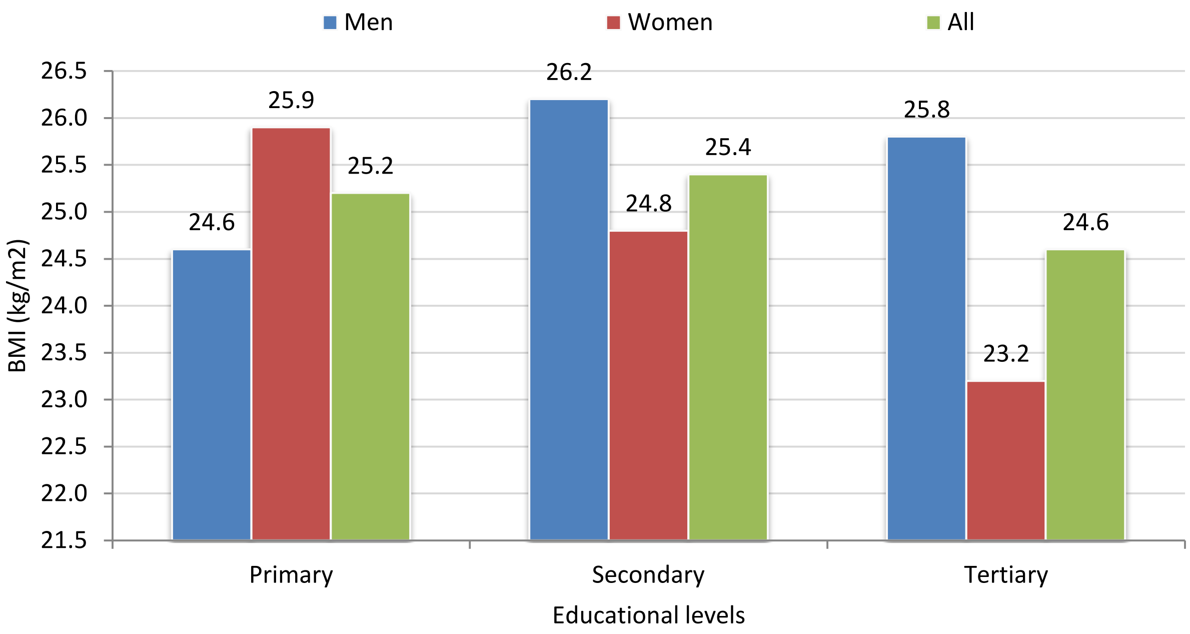

Figure 5 Average body mass index (BMI in kg/m^2; measured weight, height), overall, by sex and educational levels.

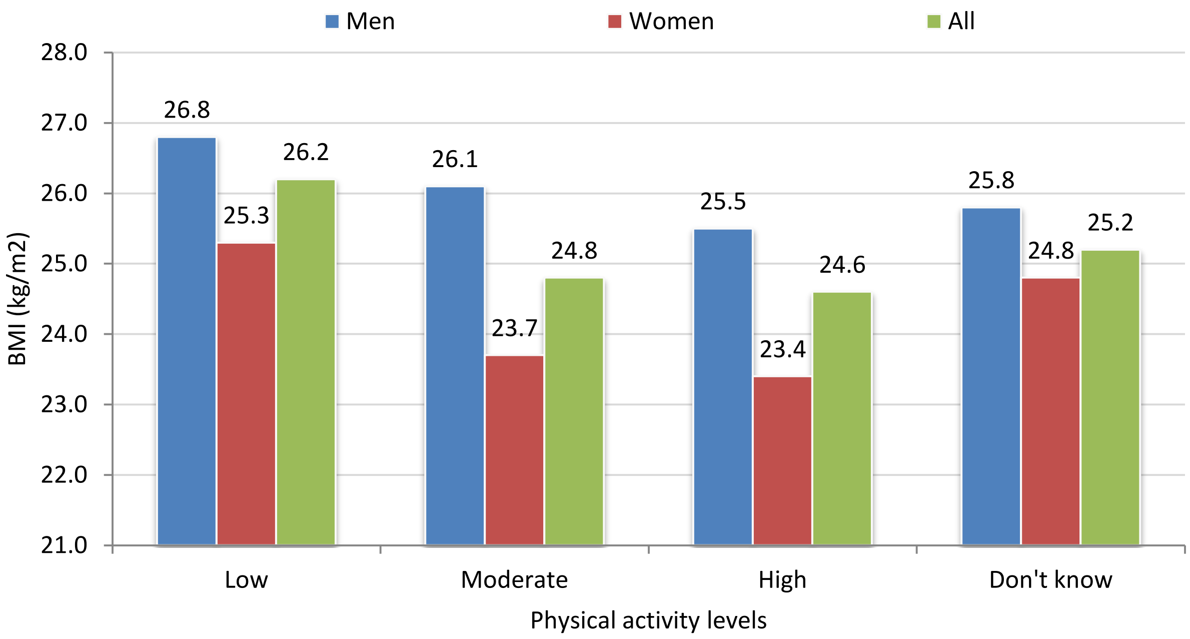

Figure 6 Average body mass index (BMI in kg/m^2; measured weight, height) by sex and physical activity levels.

In none of the presented categories was average BMI above 30 kg/m^2. At group level, men had higher mean BMI than women, overall and across all subgroups, except men with only compulsory education (Figure 5). Further, men were generally classified overweight, with men in Ticino (I-CH) having higher mean BMI (26.7 kg/m^2) than their counterparts in D-CH and F-CH. Only in the youngest age group (18-34 years) were men, on average, normal weight (mean 24.4 kg/m^2), which is in line with recent reports from

military-aged men (28). We suggest careful interpretation, because muscular men may have been misclassified as overweight; BMI is no direct indicator of abdominal fat mass. As anticipated, average BMI was lower for people being more active than for others (Figure 6). Nevertheless, the group differences (low vs. moderate and high physical activity level) were less pronounced in men than in women, and moderate and high active men were classified on average overweight.

Women's average BMI was overall, and for almost all subgroups, in the normal range (BMI 18.5-24.9 kg/m^2); no difference was found across linguistic regions (average 24 kg/m^2). Average BMI was higher in older people, in both women and men. Women, however, reached the cut-off value of BMI 25 kg/m^2 not till age 50 years, and average BMI stayed at that borderline level also for older women.

Average BMI of women with tertiary level education was clearly lower (23.2 kg/m^2) than for women with secondary or primary level education, the latter being overweight. In men differences between educational subgroups were less pronounced and particularly, it's the men with secondary and tertiary education being on average overweight (ca. 26 kg/m^2). This confirms the observation from a regional survey in Switzerland (29) that the inverse relationship of overweight and educational level tends to weaken in men but persists in women. Also the Swiss Health Survey, which relies on self-reported anthropometric data, reported a more distinct gradient of overweight by educational level for women (30).

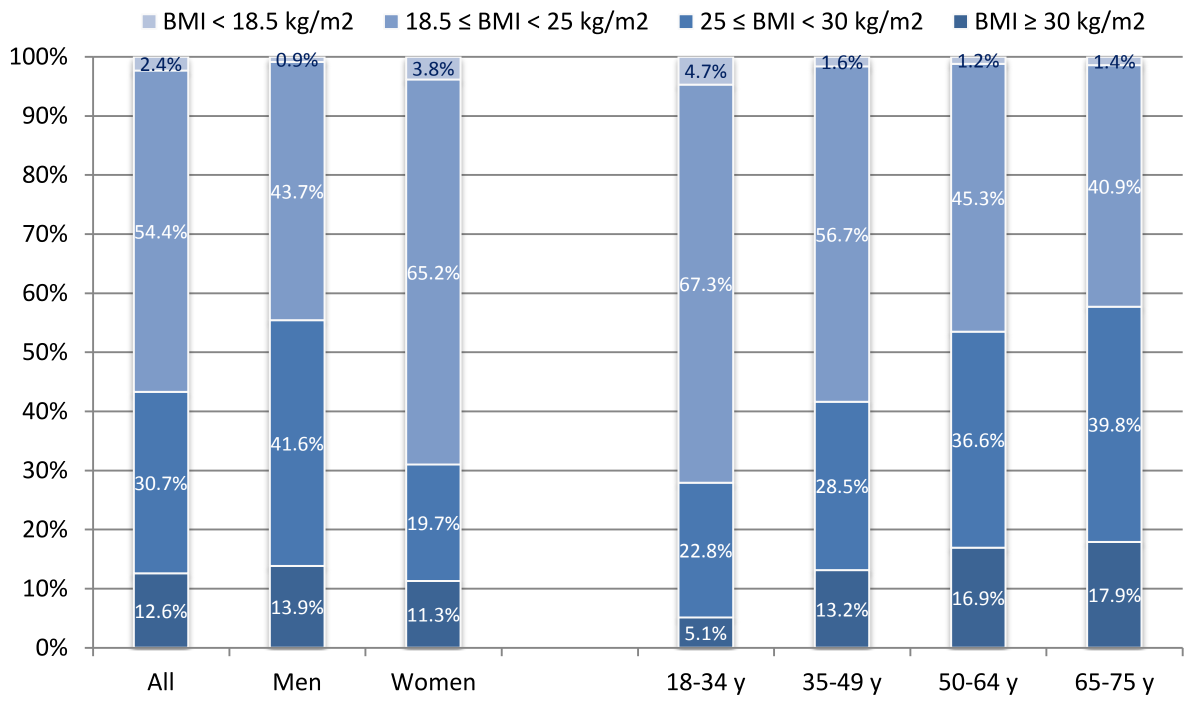

Figure 7 Relative frequency (%) of body mass index categories (measured data) overall, by sex and age groups.

Overweight was present in 30.7% of the population, 19.7% of women and 41.6% of men (Figure 7). The prevalence of obesity (BMI ≥ 30 kg/m^2) was 12.6% overall, 13.9% in men and 11.3% in women (Figure 7). As expected, obesity was higher in older age groups than in younger people, ranging from 5.1%, to 13.2%, 16.9% and 17.9% across the 18-34, 35-49, 50-64and 65-75 year categories, respectively.

Overall 2.4% of the population presented underweight, but this was more common in women (3.8%) than in men (0.9%) (Figure 7). The prevalence of underweight went from 4.7% in the 18-34 year age group to 1.4% in the 65-75 year age group.

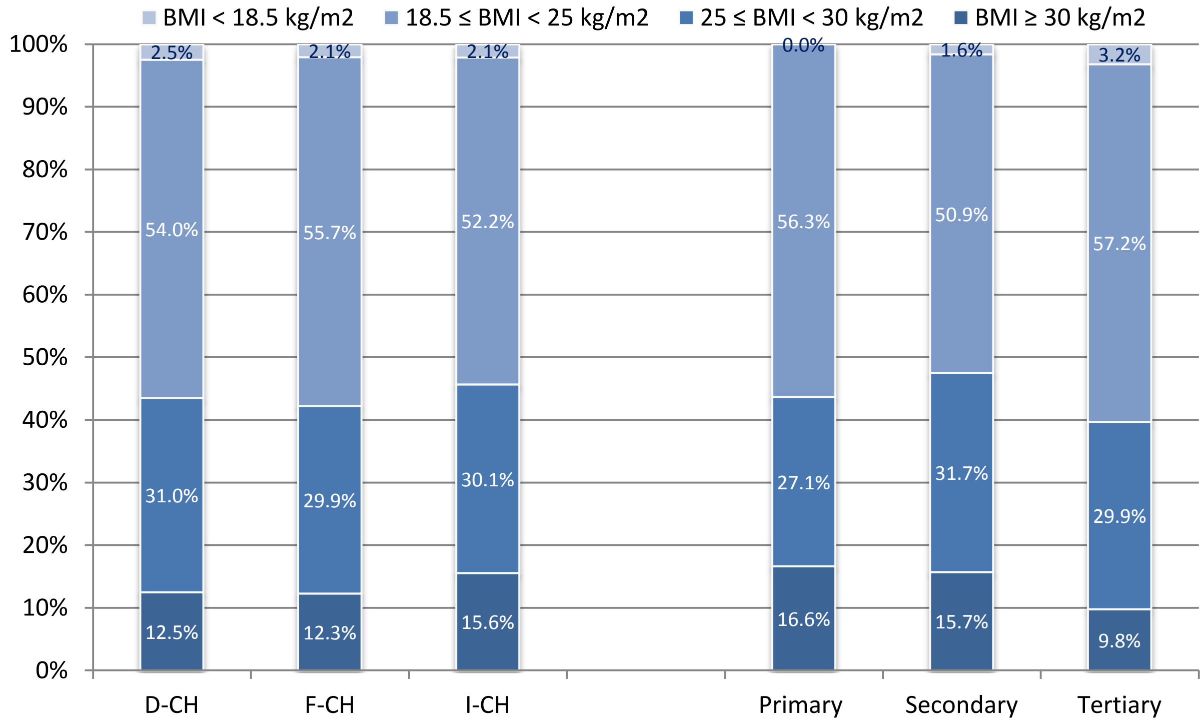

Figure 8 Relative frequency (%) of body mass index categories (measured data) by linguistic regions and educational level.

The prevalence of obesity (based on measured data) was 12.5% in the German-speaking, 12.3% in the French-speaking and 15.6% in the Italian-speaking regions (Figure 8). The prevalence of obesity was 16.6%, 15.7% and 9.8% in participants with primary, secondary and tertiary educational levels (Figure 8), which is in line with the SHS 2012 (8).

3.2.2. Measured compared to self-reported anthropometric data

Comparisons of the distributions of measured (solid line) and self-reported (dotted line) weight and height by sex (Figure 9) showed small differences. Still body weight was rather underestimated (self-report [72.3 kg] < measured [73.4 kg]) and body height overestimated (self-report [171 cm] > measured [170 cm]), both resulting in *slightly lower* BMI based on anthropometric self-report than on measured data (24.6 vs 25.0 kg/m^2, respectively).

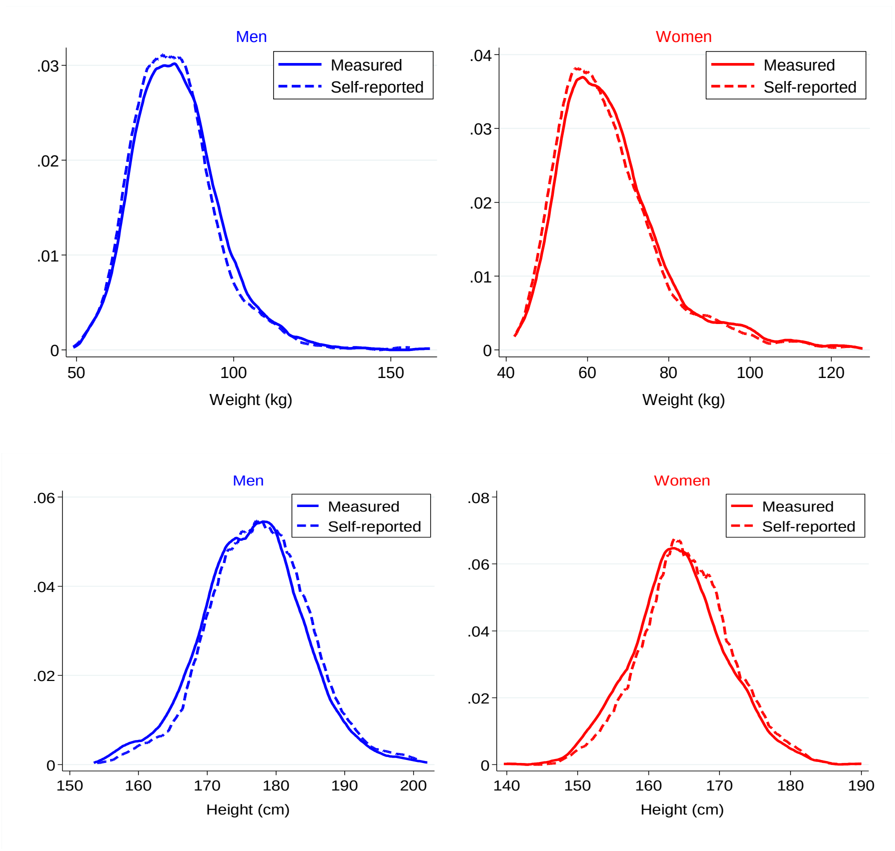

Figure 9 Distribution of measured and self-reported weight (kg) and height (cm), by sex.

The Bland-Altman plot (Figure 10) further highlights that BMI based on measured and self-reported weight and height disagree more in people with higher BMI. This confirms observations from other studies that overweight and obese people tend to misperceive and underestimate their weight (31, 32).

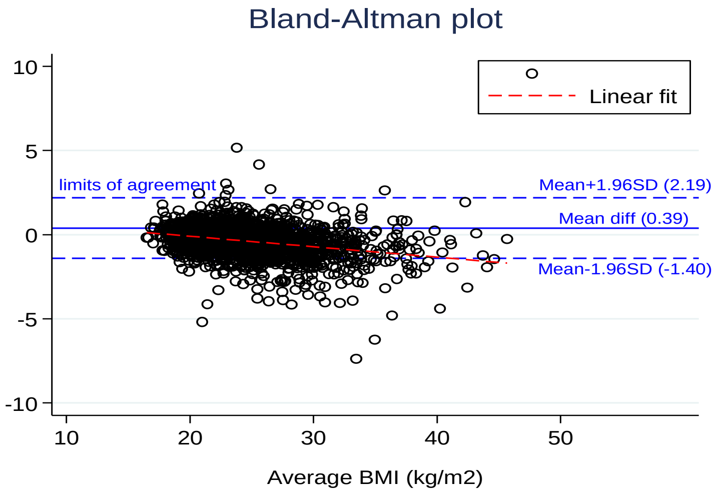

Figure 10 Bland-Altman plot for body mass index based on self-reported vs measured weight and height.

3.2.3. Waist circumference data

The measure of waist circumference (WC, in cm) provided additional information to BMI by better identifying people with abdominal obesity. Participants were classified to be at risk for metabolic complications according to the WC cut-offs or subgroups, for men (M) and women (W) respectively: (i) no risk ≤ 94 cm (M), ≤ 80 cm (W); (ii) increased risk 94.1-101.9 (M), 80.1-87.9 (W); (iii) substantially increased risk ≥ 102 cm (M), ≥ 88 cm (W).

We present the relative frequencies of participants who were classified at no, increased or substantially increased risk for metabolic complications, according to WC measurement. Overall, 16.5% and 16.6% had WC placing them at highly increased or increased risk, respectively. The prevalences of WC at highly increased risk and increased risk were16.4% and 18.6% in men, and 16.7% and 14.7% in women, respectively. In Figure 11, we present the relative frequencies by linguistic regions, separately for men and women. In Figure 12, we present the relative frequencies by age groups overall. In Figure 13, we present the relative frequencies by age groups, separately for men and women. In Figure 14, we present the relative frequencies by educational levels, separately for men and women.

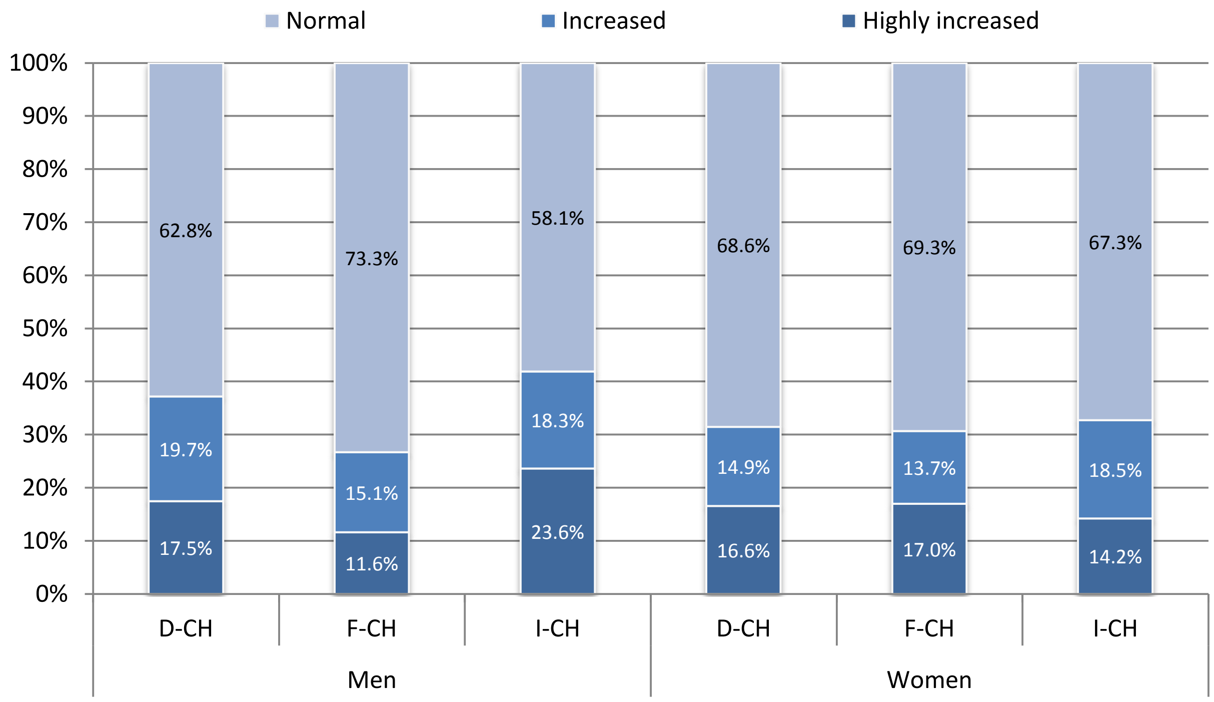

Figure 11 Relative frequency (%) of waist circumference risk categories by linguistic regions for men, women.

Women residing in D-CH, F-CH or I-CH showed quite similar risk profiles (Figure 11): about 30% had a WC that placed them at increased or substantially increased risk of future ill health. A difference situation is observed in men. In F-CH about 10% fewer men were at metabolic risk than in D-CH and I-CH, that is 30% compared to 40%. The difference was particularly visible in the group of men with highly increased risk or having a WC of at least 102 cm.

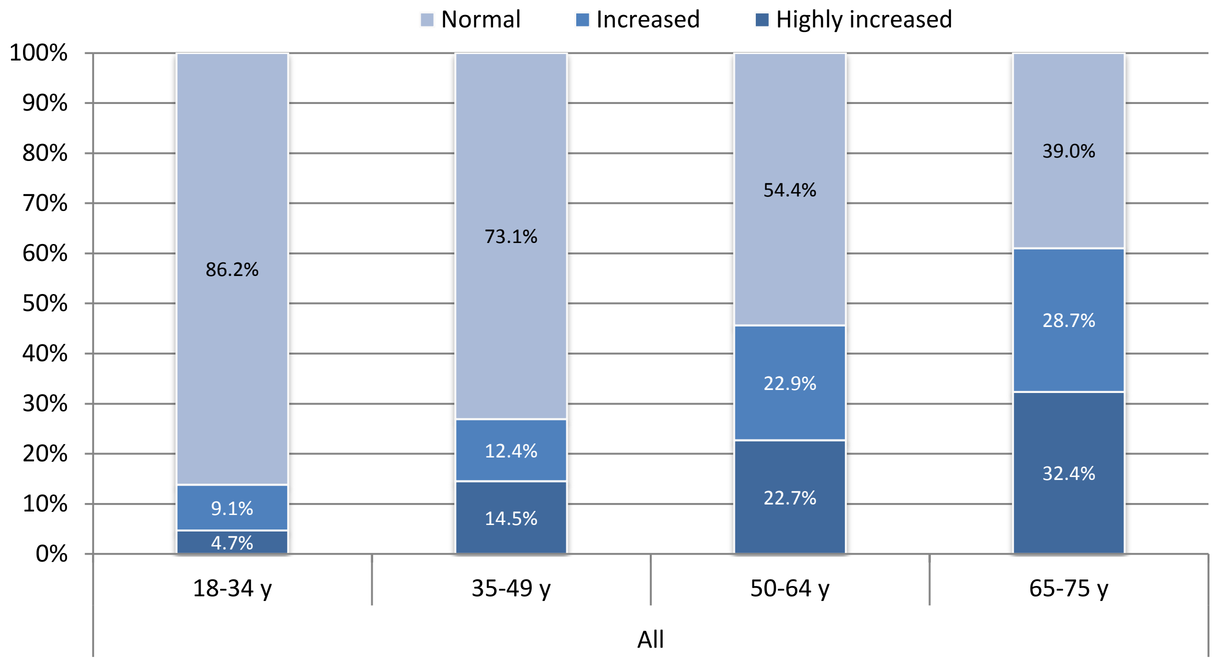

Figure 12 Relative frequency (%) of waist circumference risk categories by age groups overall.

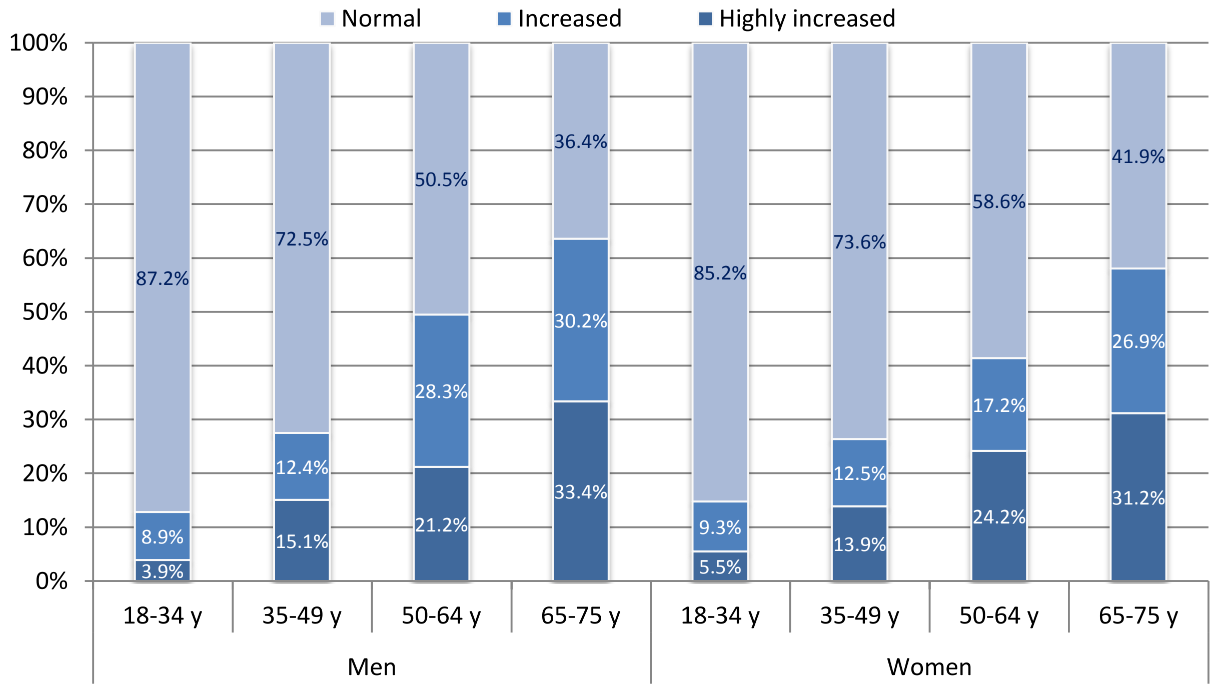

Figure 13 Relative frequency (%) of waist circumference risk categories by age groups for men and women.

The relative frequencies of WC risk categories by age groups overall (Figure 12) show a steep age-related gradient. The older people were, the higher was the proportion of people (61% at age 65-75 years) with an increased or substantially increased metabolic risk due to abdominal obesity. The drop in no risk prevalence is strongest (19%) between age groups 35-49 years and 50-64 years although the observed

increase in obesity based on BMI was not as marked (Figure 7). This age dependency was stronger in men than in women, as illustrated in Figure 13.

The metabolic risk prevalence by educational level (Figure 14) was different for men and women. In men, we found no major differences; men of secondary or tertiary educational level had 3-5% higher prevalence of substantially increased metabolic risk compared to men with compulsory education.

In women we observed a rise in the proportion of no metabolic risk by educational level, particularly from secondary to tertiary level (+16%). The prevalence of substantial metabolic risk was highest in women with compulsory education (32%), which reflects the picture of mean BMI (Figure 5).

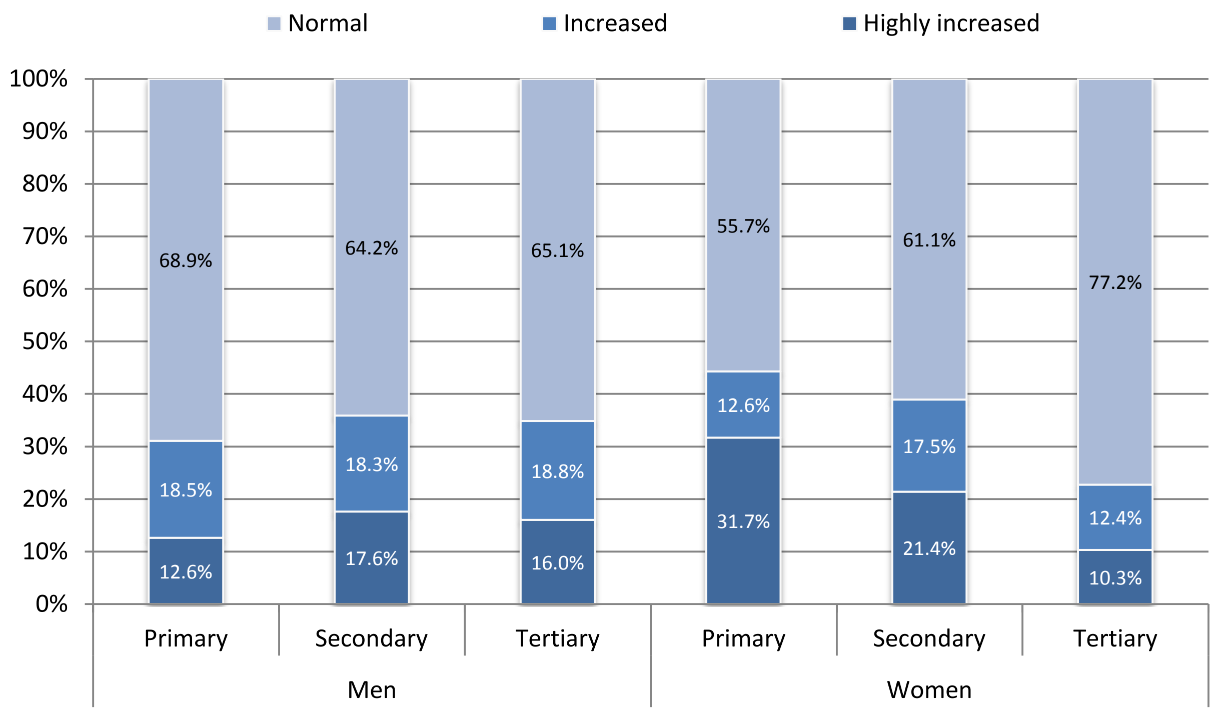

Figure 14 Relative frequency (%) of waist circumference risk categories by educational level for men, women and overall.

We compared the distribution of BMI categories between people with normal WC risk category and those with WC that put them at increased or highly increased metabolic risk (combining these two risk categories to ease the interpretation) (Figure 15). Overall, in men and in women, the prevalence of obesity was close to zero in those at normal risk category for WC. In men at increased risk category for WC, less than 4% were neither overweight nor obese. In women at increased risk category for WC, 20.2% were normal weight according to BMI.

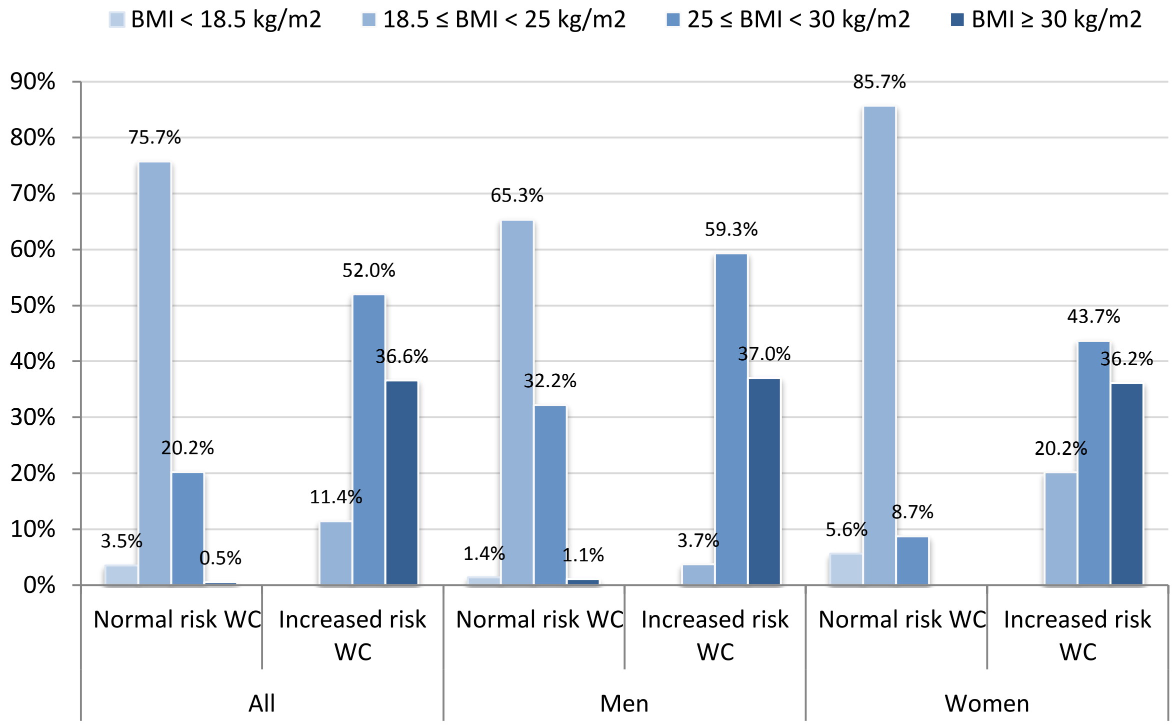

Figure 15 Relative frequency (%) of body mass index categories by waist circumference risk categories (normal vs increased) overall, in men and in women.

3.2.4. Body weight satisfaction and weight management

Body weight satisfaction

Beside the above described objective measures of body shape, menuCH asked the population about weight related attitudes and perceptions. Figure 16 shows the prevalence of people who were (un-) satisfied with their body weight at time of the survey overall by sex and age groups. Overall, slightly more men (60%) than women (57%) were satisfied with their body weight. This was also observed in the Swiss Health Survey 2012 (30). Figure 17 highlights the differences across age groups in men and women separately. In the age group 35-49 years, 46% of men were not satisfied with their current body weight (Figure 17). In the other age groups at least 60% of men were satisfied. In women, the two younger age groups, i.e. women aged 18-34 and 35-49 years were the least satisfied. Overall, 10% of women were not at all satisfied with their weight (Figure 16), and this was particularly true for 13% of women in the age group 35-49 years (Figure 17).Body weight satisfaction tended to be higher in women with tertiary education level than in women with secondary or primary (Figure 18). No such clear gradient across educational levels was observed in men. Differences in weight satisfaction across linguistic regions were rather small (Figure 19). In Ticino (I-CH) 10% of men were not at all satisfied compared to less than 5% in the other two linguistic regions. The prevalence of women extremely satisfied with their weight was lower in F-CH (15%) compared to D-CH (21%) and I-CH (19%).

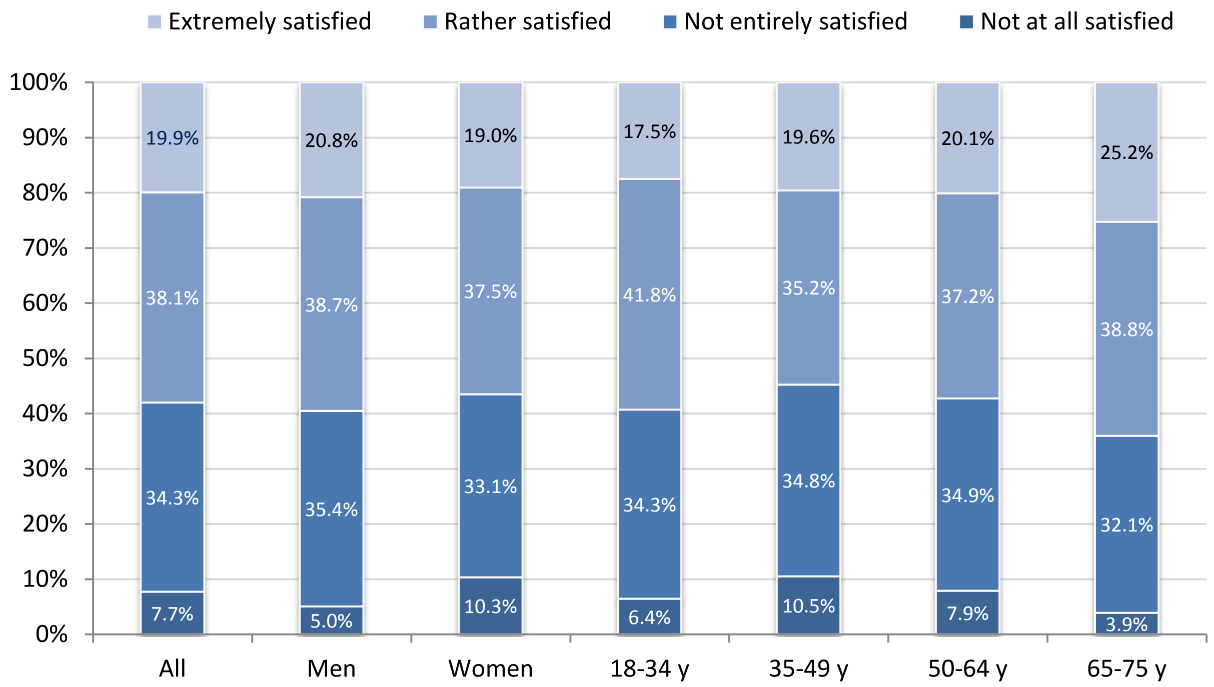

Figure 16 Relative frequencies (%) of body weight satisfaction categories, overall, by sex and age groups.

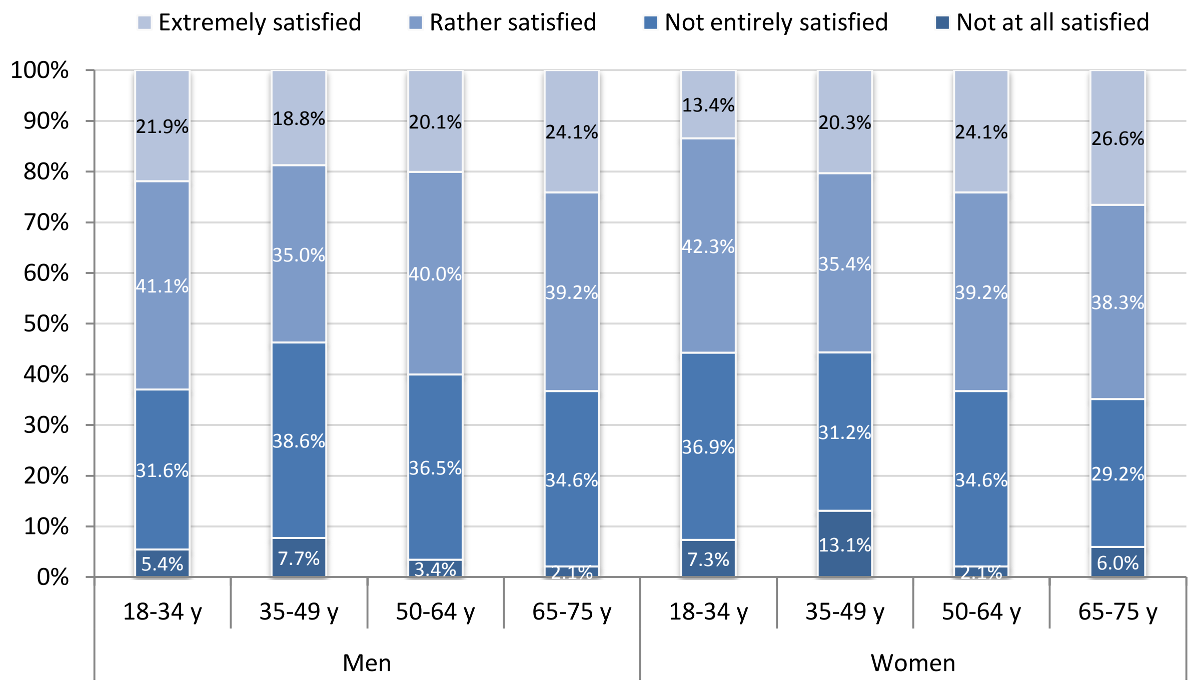

Figure 17 Relative frequencies (%) of body weight satisfaction categories, by age groups, in men and women.

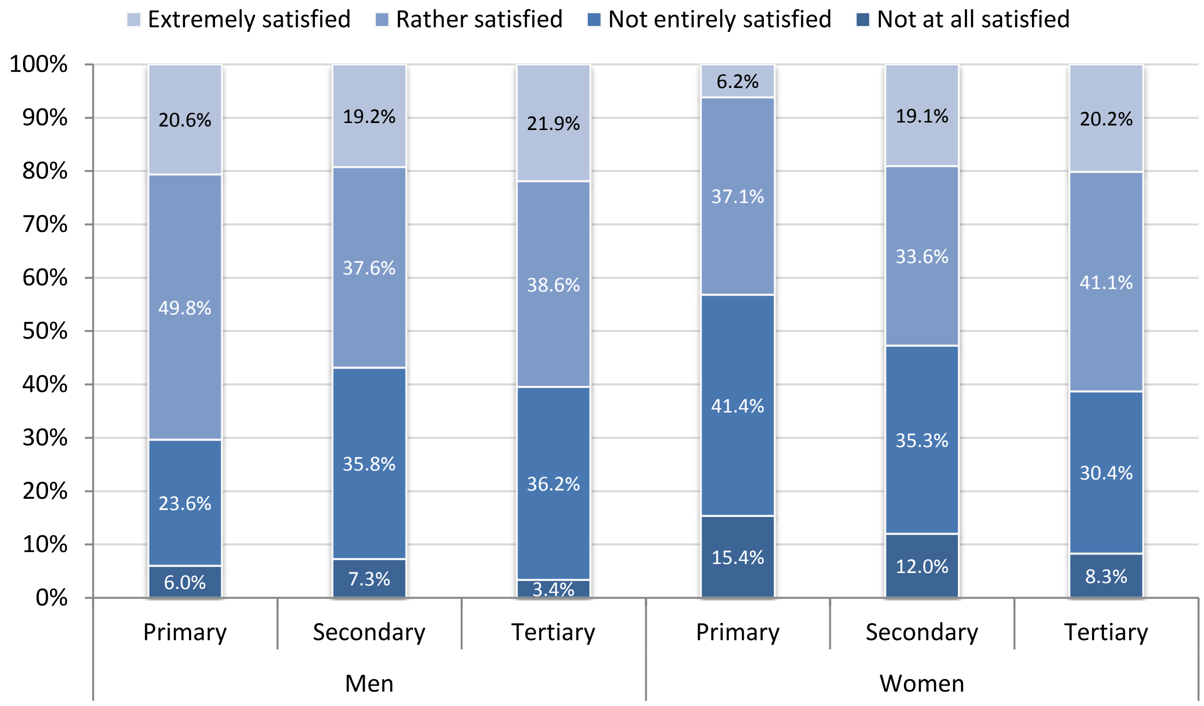

Figure 18 Relative frequencies (%) of body weight satisfaction categories, overall, by sex and educational levels.

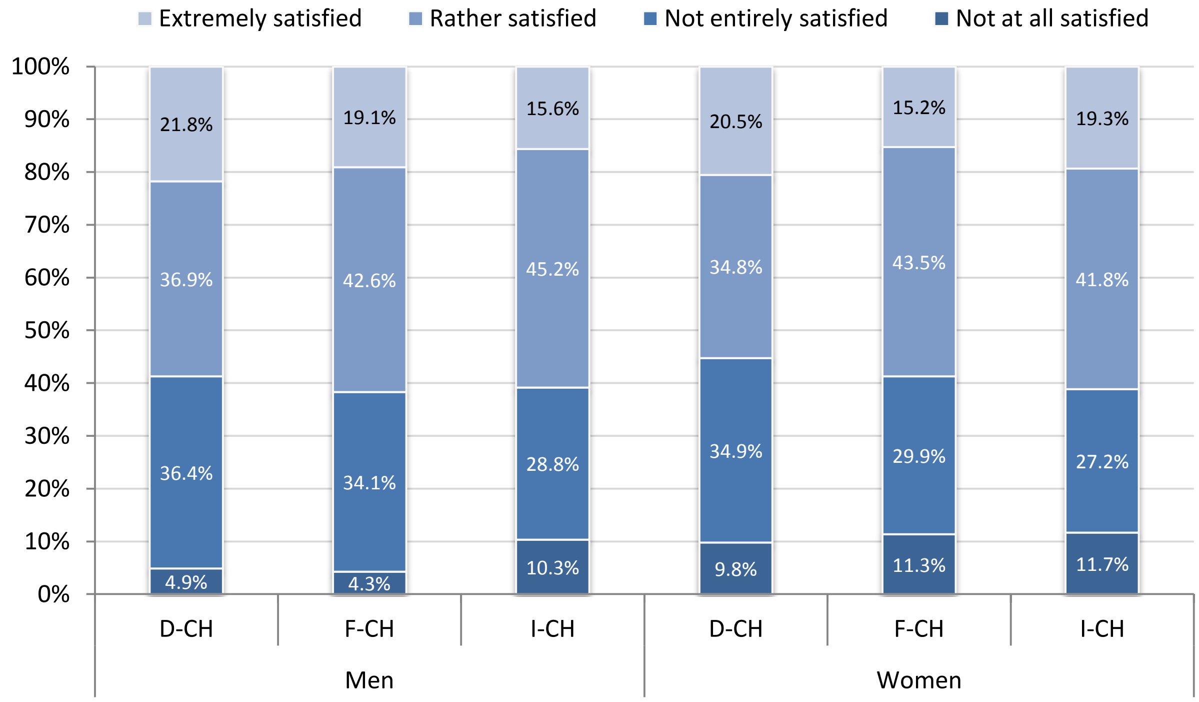

Figure 19 Relative frequencies (%) of body weight satisfaction categories across linguistic regions, in men and in women

Comparison of body weight satisfaction with measured BMI categories

We grouped people into those who were rather satisfied or extremely satisfied with their body weight (labeled as "satisfied") and those who were not entirely satisfied or not at all satisfied (labeled as "not satisfied") and compared the distributions of the BMI categories in those two groups, as illustrated in Figure 20. The prevalence of obesity in those who reported to be satisfied of their body weight is very low, overall, in men and in women. By contrast, the prevalence of obesity in those who reported not be satisfied with their body weight was high (about 25-28%) overall, in men and women.

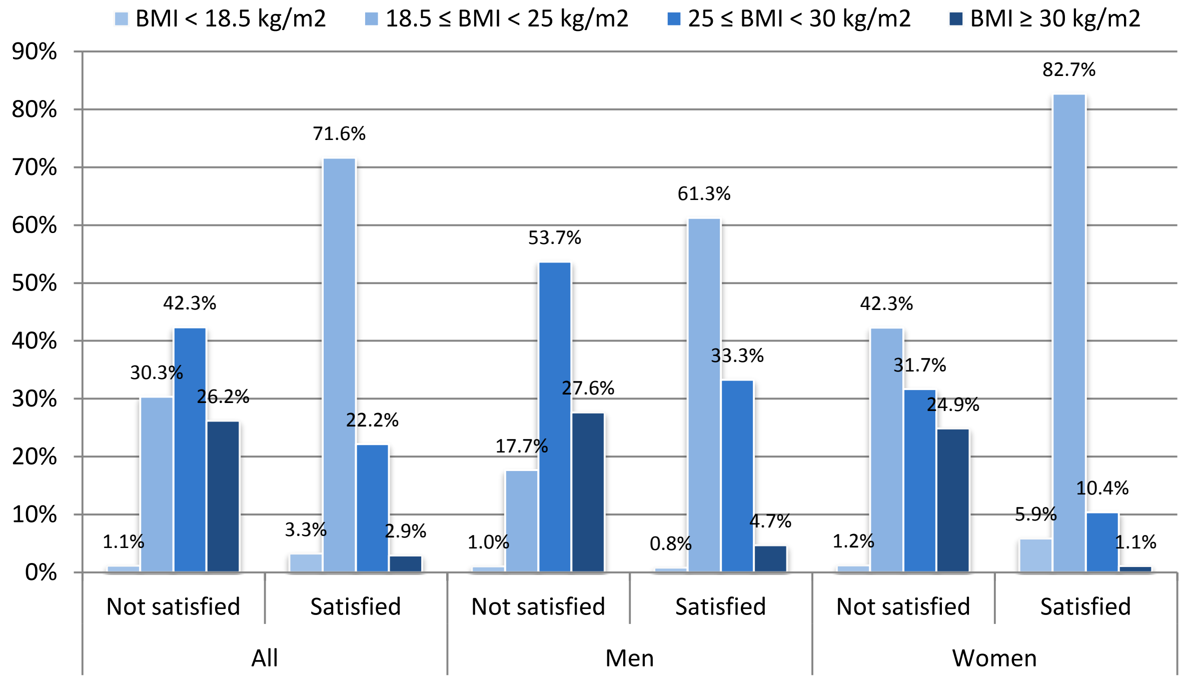

Figure 20 Relative frequencies (%) of body mass index categories by level of body weight satisfaction, overall, in men and women

Weight management plans and practice

Respondents were also asked if they wish to lose weight, maintain their current body weight or gain weight. A slightly larger proportion of women (55%) than men (51%) would like to lose weight (Figure 21). Figure 21 also shows that weight maintenance becomes more important with increasing age. The proportion of people wishing to reduce weight (53%) is slightly higher than the proportion of people being dissatisfied with their body weight (42%, Figure 16).

According to Figure 22, about 18% of the 18-34 years old men were preoccupied by gaining weight. We may speculate that this concerned gain in muscle mass. In the same age group, 43% of men but 58% of women would like to lose weight. The proportion of men who wished losing weight was highest in the age group 35-49 years (58%) and became steadily lower until age 65-75 years (46%). In women, across three age groups, i.e. from 18 to 64 years, about 56%-57% wished losing weight; only in the oldest age group was the prevalence lower by 15%.

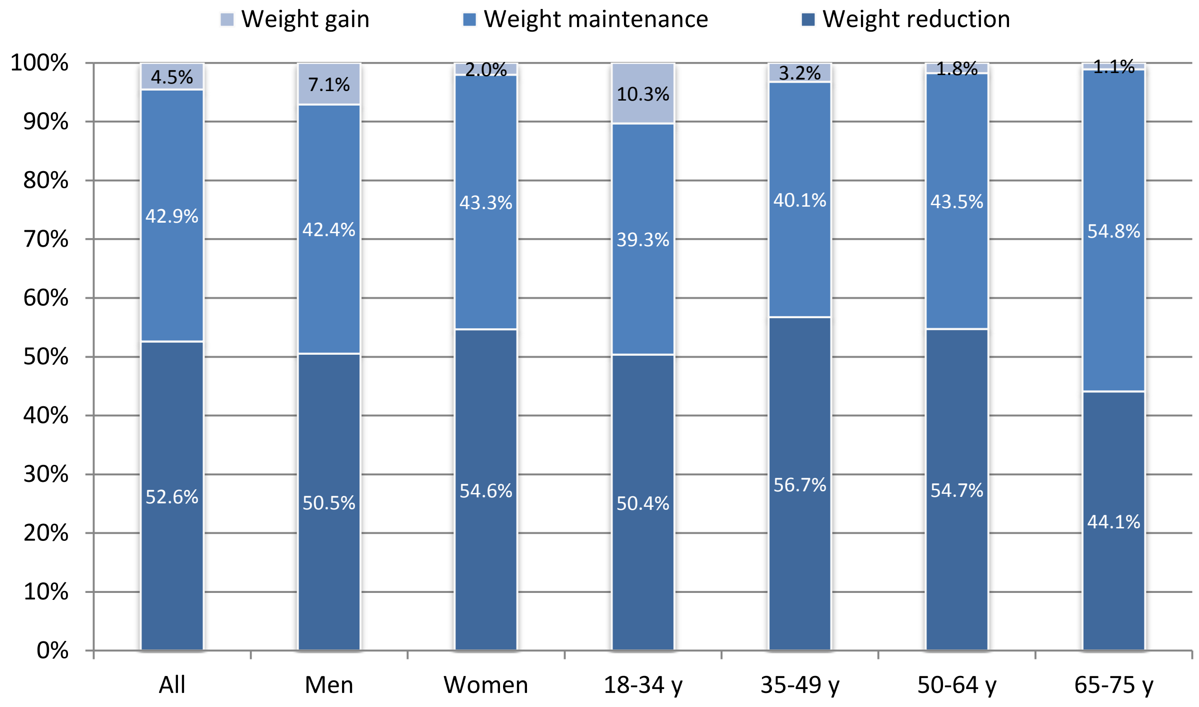

Figure 21 Relative frequencies (%) of weight management plan categories, overall, by sex and age groups.

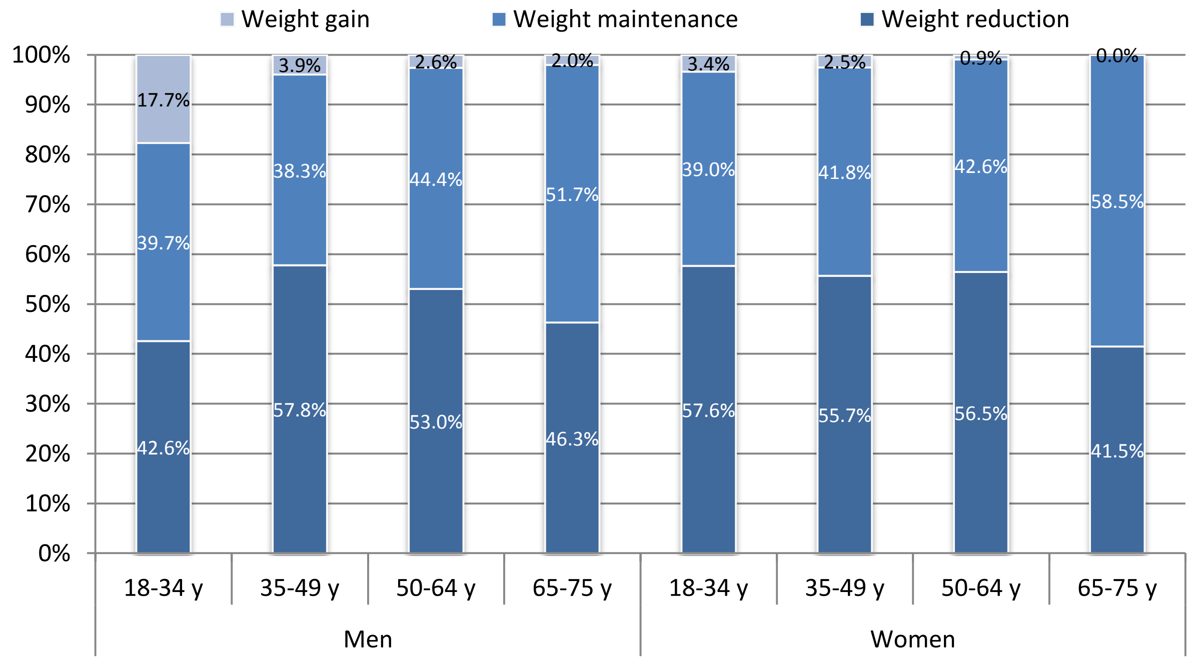

Figure 22 Relative frequencies (%) of weight management plan categories, by age groups, for men and women.

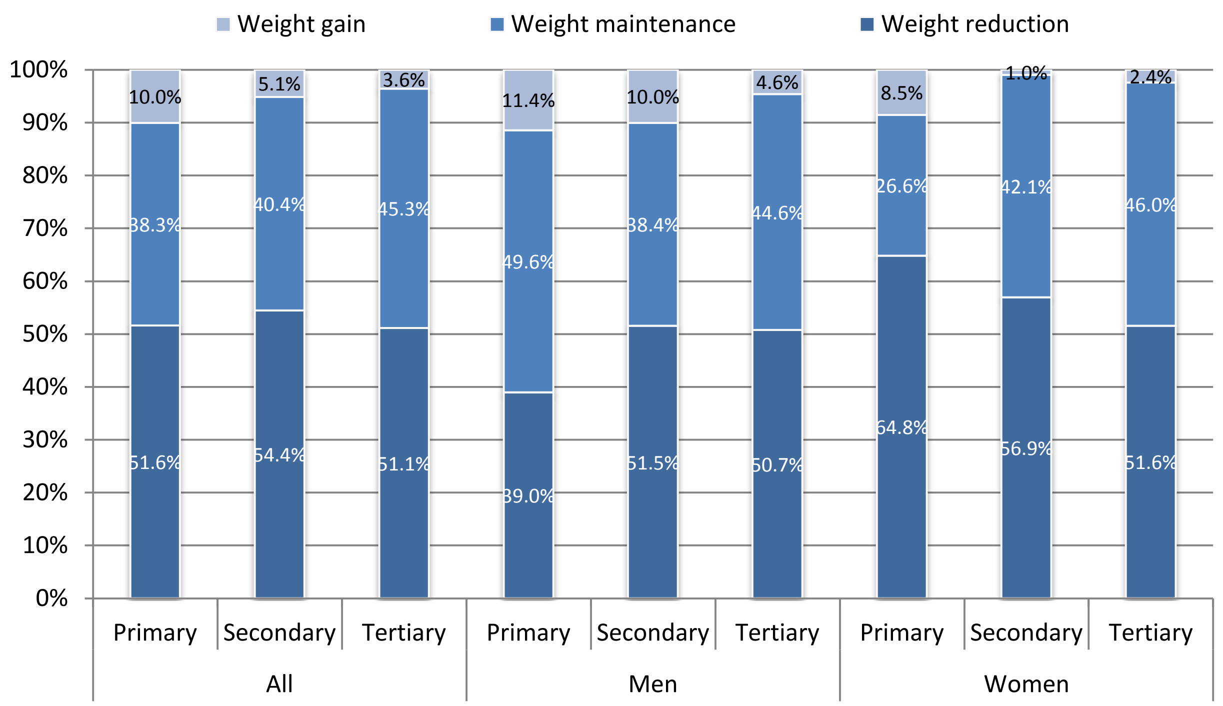

Figure 23 Relative frequencies (%) of weight management plan categories, by educational levels for men, women and overall.

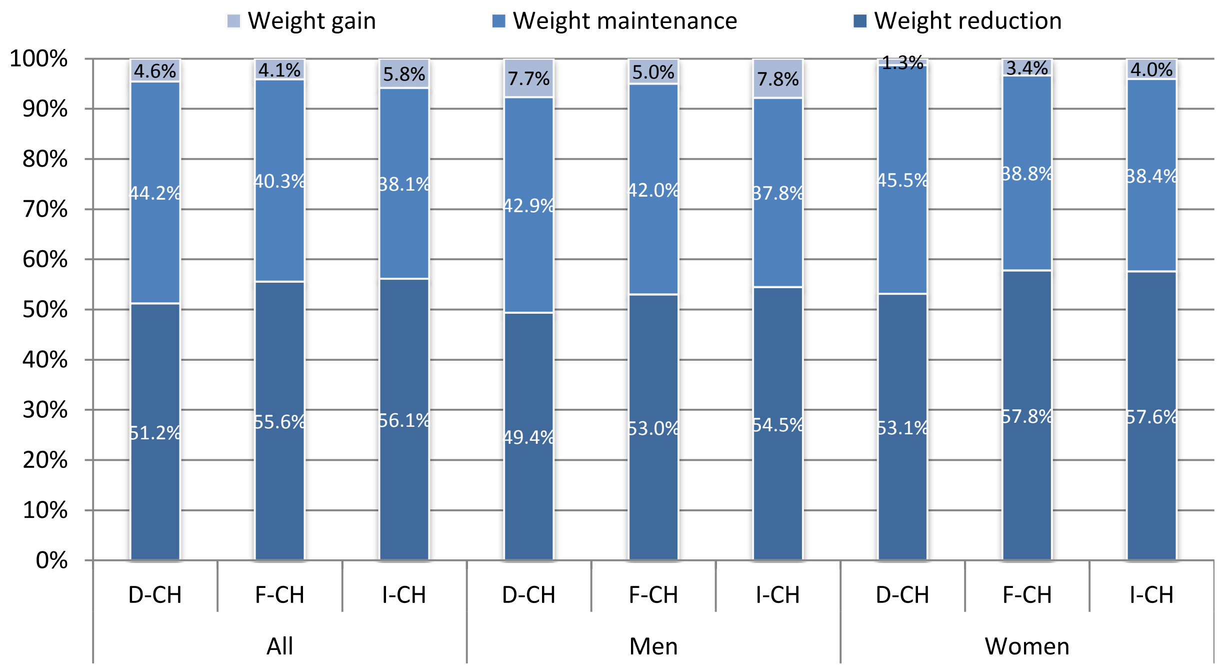

Figure 24 Relative frequencies (%) of weight management plan categories, by linguistic regions, overall, for men and women.

The general picture of Figure 23 resembles the one from body weight satisfaction. Overall, there were no large differences in weight loss desire across educational levels. However, weight maintenance was more important in people with secondary and tertiary education, particularly in women. A small proportion (10%) of women and men at the primary educational level and men at the secondary educational levels wanted to gain weight.

The prevalence of people wishing to gain weight is similar (about 5%) in the three linguistic regions (Figure 24). In each region, more men than women would like to gain weight and slightly more women than men would like to lose weight.

About 50% of the population wished to reduce body weight (Figure 21). The next figure (Figure 25) shows the proportion of respondents who reported to be on a diet at time of the survey (current) (5.5%) or to have been on a diet in the previous 12 months (12.5%). Generally, more people have tried losing weight during the past year than did at time of interview. A larger proportion of women than men, reported to be currently on a diet or to have been on a diet during the past 12 months (Figure 25), and this is observed across all age groups (Figure 26). Much more people in the age group 18-34 years than people from the older age groups reported to have been on a diet during the past 12 months, overall (Figure 25) and by sex (Figure 26).

Across all educational levels, more women than men reported to be currently on a weight loss diet (Figure 27), but the sex difference was particular marked for people with primary education. Sex differences also differ by educational level for being on a weight loss diet during the past 12 months (Figure 27): a similar proportion of men and women with tertiary education reported to have been on a diet during the past 12 months, which contrast with a higher proportion of women than men for the other educational levels.

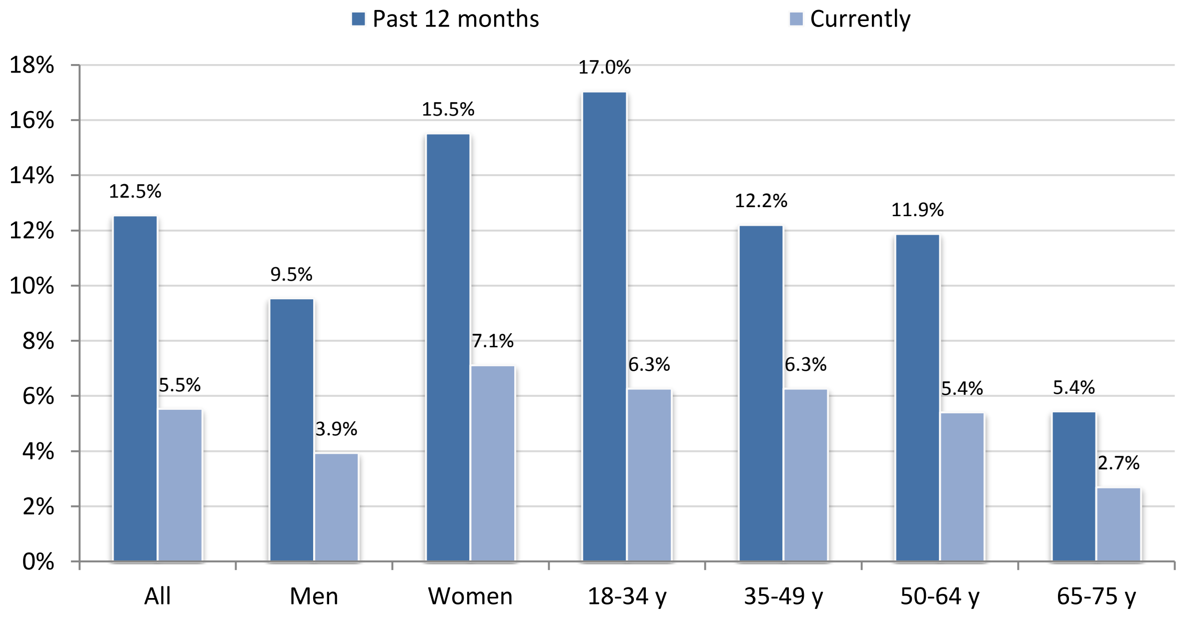

Figure 25 Percentage (%) reporting to be on a weight loss diet, overall, by sex and by age groups.

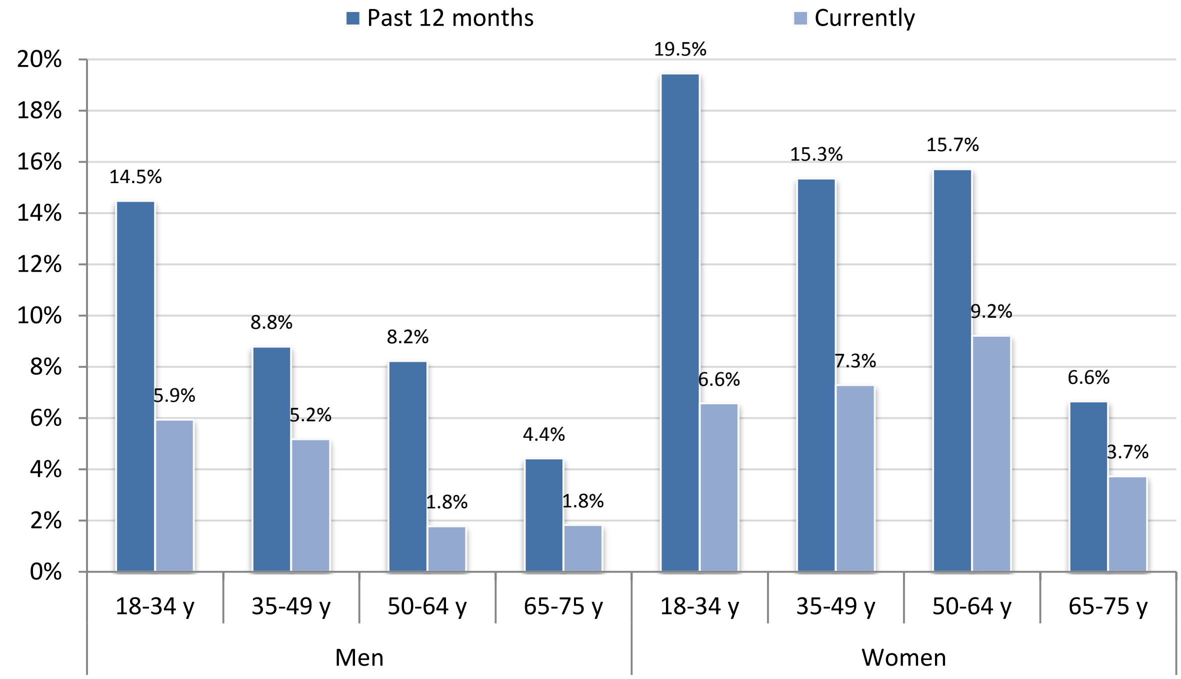

Figure 26 Percentage (%) reporting to be on a weight loss diet, by age groups for men and women.

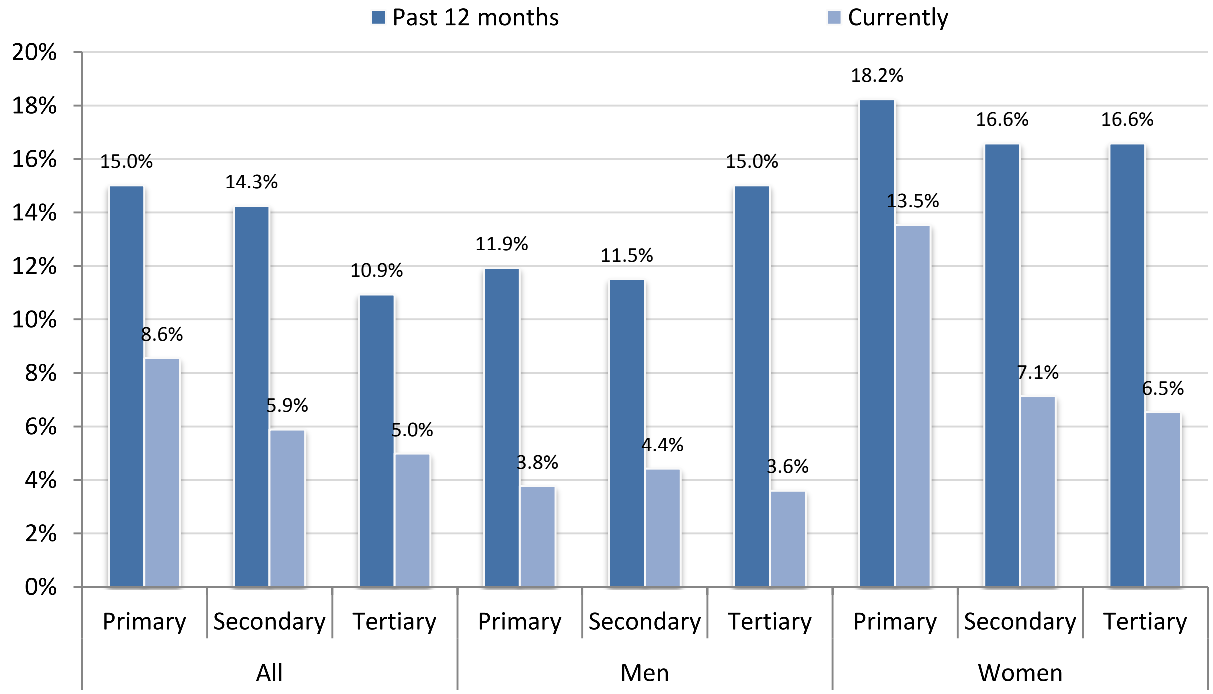

Figure 27 Percentage (%) reporting to be on a weight loss diet, by educational levels, overall, for men and women.

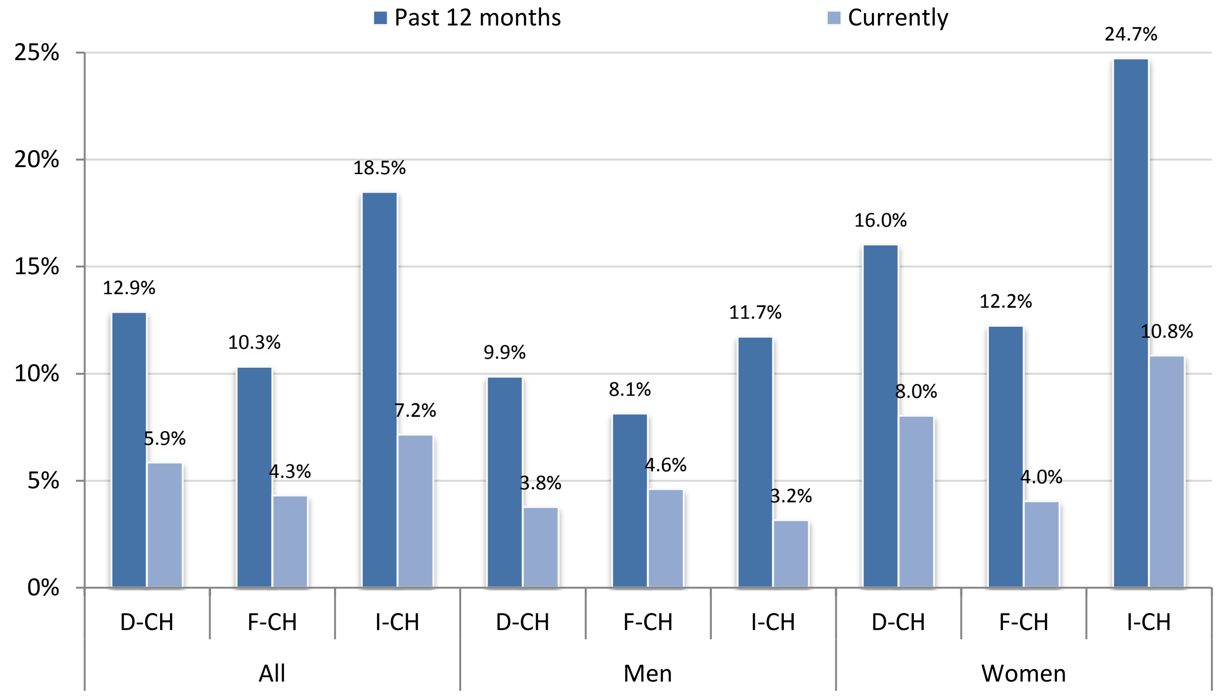

Figure 28 Percentage (%) reporting to be on a weight loss diet, by linguistic regions, overall, for men and women.

For men and women, keeping a weight loss diet during the previous 12 months was more frequent in I-CH than in D-CH and F-CH (Figure 28). This regional difference was also true for women keeping a weight losing diet at time of the interview. But slightly more men from F-CH and from D-CH than from I-CH had reported being currently on a diet.

Weight management plans versus body mass index categories

We compare BMI categories between those who reported to wish to lose weight and the others (who reported to want to maintain their weight or to gain weight) (Figure 29). The prevalence of obesity was low (< 4%) among those who do not wish to lose weight, overall, in men and women. By contrast, the prevalence of obesity was over 20% among those who wish to lose weight. Figures were similar in men and women. About half the men and one third of women who reported to wish to lose weight were overweight (Figure 29). Of note, 50% of women with a BMI within the normal range (i.e. 18 – 25 kg/m^2) would like to lose weight.

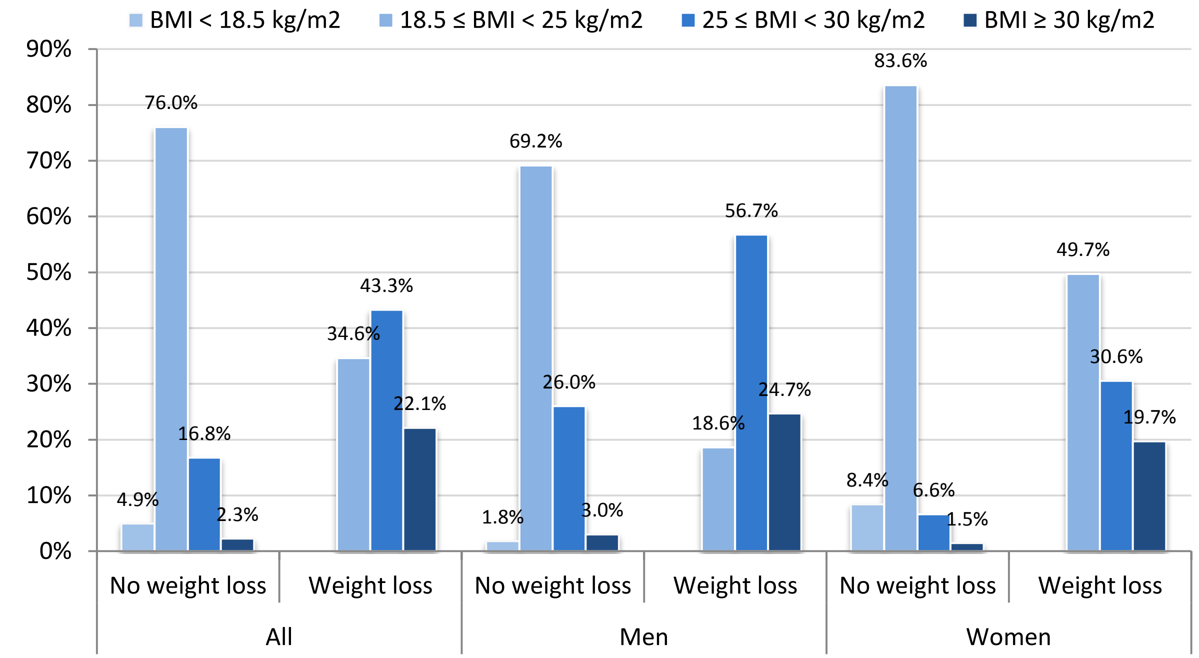

Figure 29 Relative frequencies (%) of body mass index categories by weight loss plan, overall, for men and women.

3.3. Nutrition

3.3.1. Diet related behaviors

Special diet

When asked at the beginning of the 24HDR interview, 34% of respondents stated to habitually follow a special diet. As shown below (Figure 30), more women (41%) than men (28%) did so. Differences were also observed across linguistic regions, with about 10% more people in D-CH than in F-CH and I-CH reporting to eat a special diet. No large differences were observed across educational levels but the older the people the more they reported to follow a special diet, which could be related to higher prevalence of certain nutrition-related diseases and risk factors like for example diabetes mellitus and arterial hypertension. However, results should be interpreted with caution, since interviewers (dieticians) may have interpreted the often narrative reports differently.

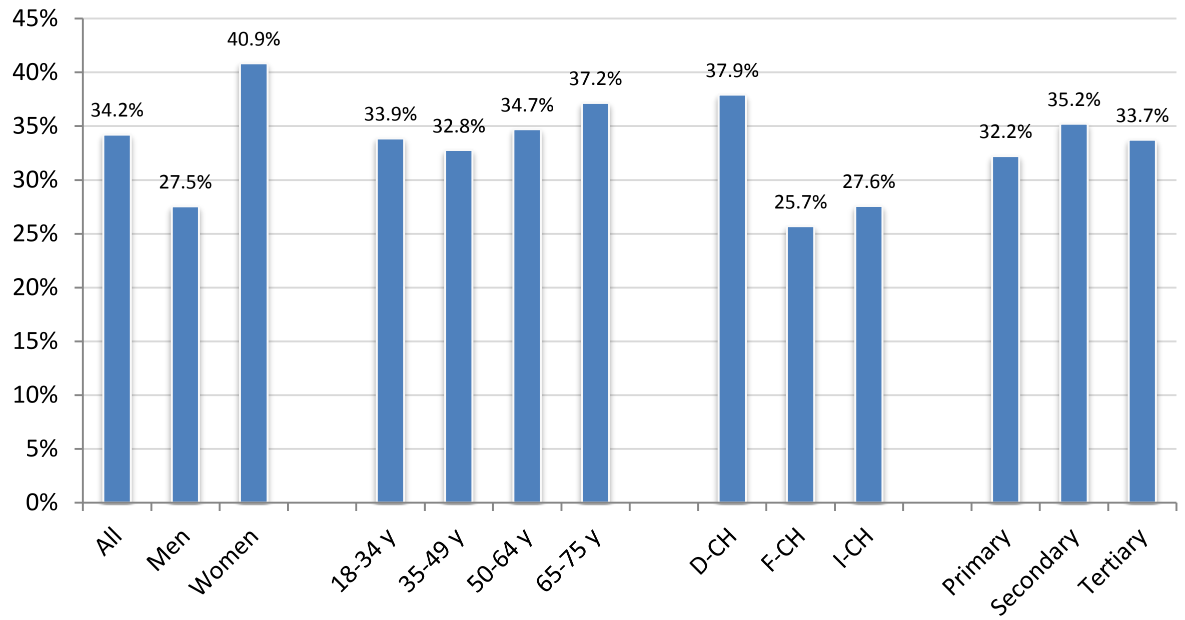

Figure 30 Percentage (%) reporting any type of special diet, overall, by sex, age groups, linguistic region and educational level.

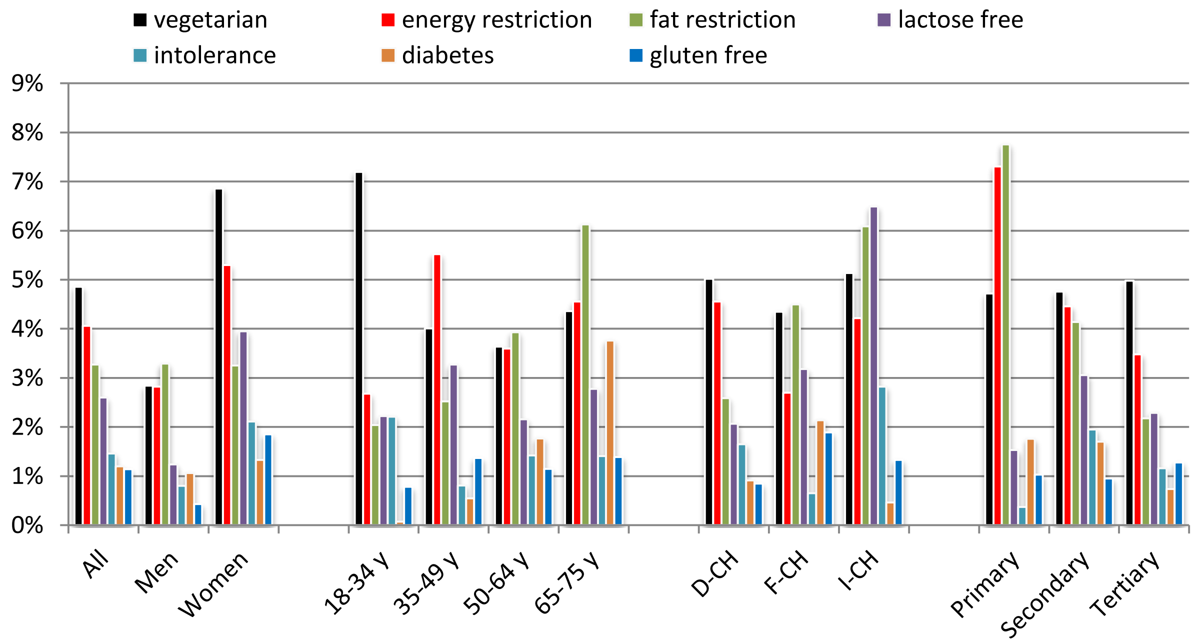

Figure 31 Percentage (%) reporting selected special diets (reported at least 20 times), overall, by sex, age groups, linguistic region and educational level.

In Figure 31 we show those special diets that were reported by at least 20 participants. We did not group lactose-free and gluten-free with intolerance since we cannot rule out that a certain proportion of people do not have an intolerance but follow such diet due to the growing market/marketing of gluten free and lactose free foods. Overall, vegetarian diet is the most prevalent form of a special diet, followed

by energy and fat restriction, which may be related to constant weight control endeavors. As suggested above, in the oldest age group diabetes and fat-restricted diets were reported more often. Participants from Ticino (I-CH) were more concerned about fat restriction and cutting out lactose of their diet. As shown in Figure 27, people with primary education had reported more often to be on a diet, which seems to be most often energy/fat restricted diets according to Figure 31.

The remaining majority of reported diets were summarized in the category "other special diets" (Figure 32) since representing many single case reports. The category "other diets" includes for example reported daily protein restriction, salt restriction, choosing easily digestible foods, eating a diet enriched in fiber, following anthroposophical, macrobiotic, Muslim or Jewish philosophies of eating, but also eating differently during pregnancy or breast feeding. Higher educated people may have elaborated more on this issue and, as already mentioned above, we may assume that dieticians noted details/specifications somewhat differently, particularly in D-CH.

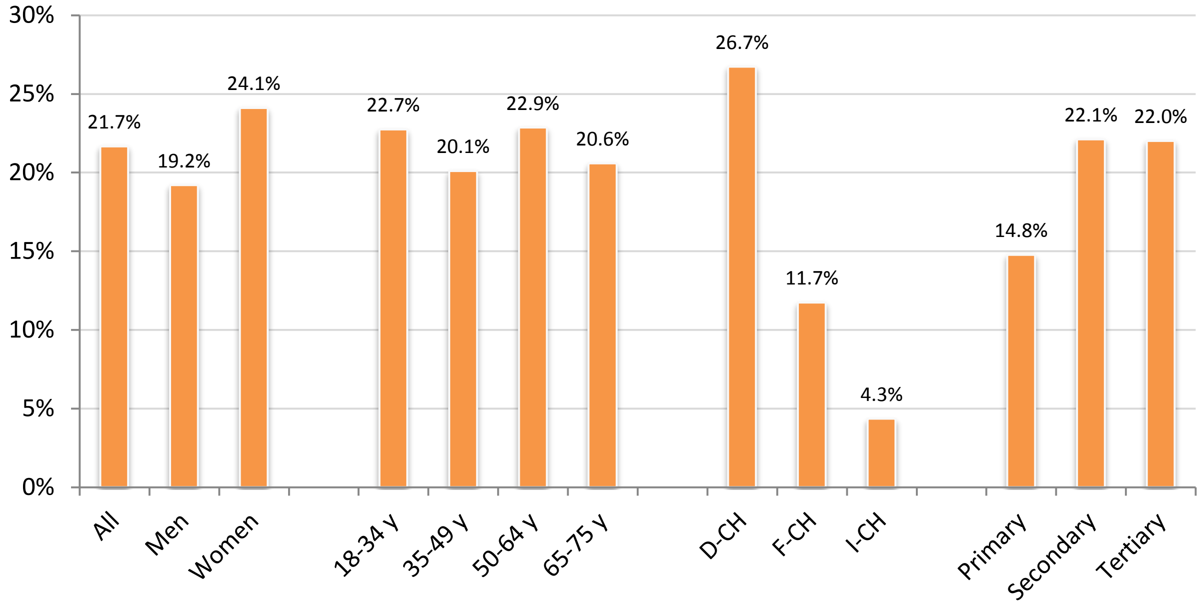

Figure 32 Percentage (%) reporting other special diets, overall, by sex, age groups, linguistic region and educational level.

Supplement use

According to Figure 33, the overall supplement use, which represents the total of vitamins, minerals, combined products or other supplements, was shown to be quite common in Switzerland and more frequent in D-CH than in F-CH and I-CH. Almost half of the overall population (47%) reported such use, with more women (56%) than men (38%) and more people above 50 years of age (50% and 55% for the 50-64 year and 65-75 year age groups, respectively). Differences by educational level were small.

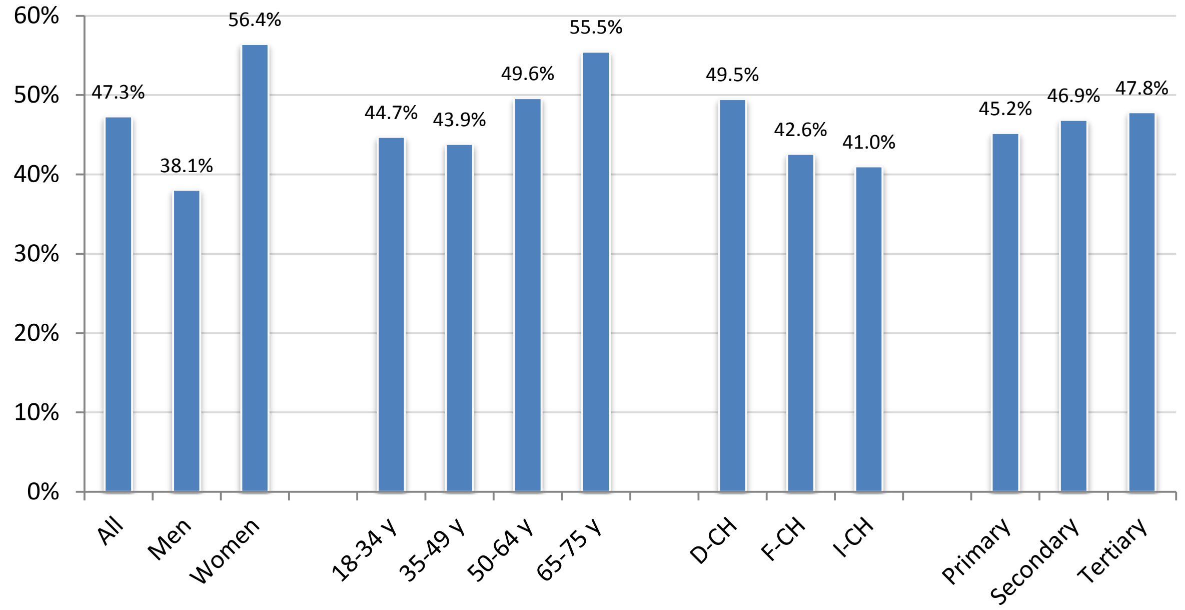

Figure 33 Percentage (%) of overall supplement use, overall, by sex, age groups, linguistic region and educational level.

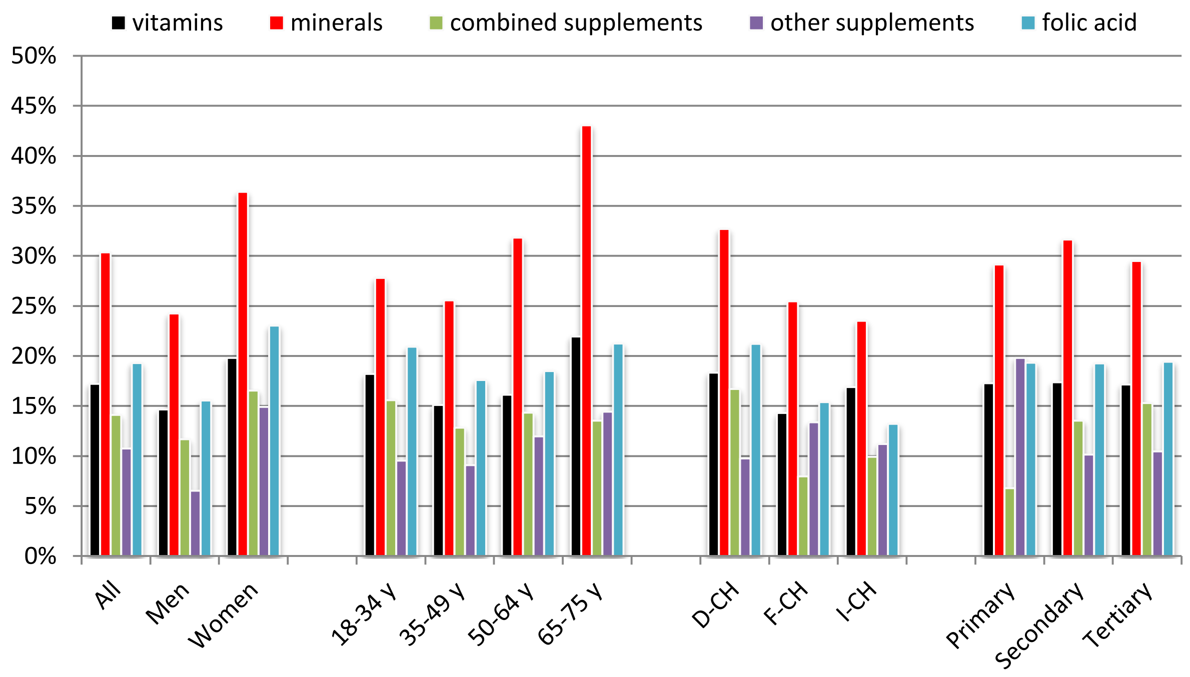

Figure 34 Percentage (%) of specific supplement use, overall, by sex, age groups, linguistic region and educational level.

Looking at products separately (Figure 34), minerals are more frequently reported to be used than vitamins and combined products and their use sharply increases with age. The overall observed differences across linguistic regions (Figure 33) persisted. A range of other supplements were reported to

be consumed by 10% of the population, more in women than in men. A separate question was asked for the use of products containing folic acid. As expected, more women (23%) than men (16%) reported to do so, and in the youngest and oldest age group intake was a bit more frequent (21% vs. 18%).

Awareness of food guidelines and campaigns

The federal authorities promote different tools and run nutrition campaigns to support the population in implementing a health-promoting lifestyle. According to the menuCH survey (Figure 35), 77% have heard about the “Swiss food pyramid”. However, in D-CH, 10 percentage points less people were aware about the food pyramid than in the other linguistic regions. Further, more women (86%) than men (67%), more people with secondary (80%) or tertiary (75%) than primary (66%) education had heard about the “Swiss food pyramid”. Awareness of the food pyramid was much higher people aged 18-34 years (83%) than in people aged 65-75 years (66%), with intermediate awareness in middle-age groups.

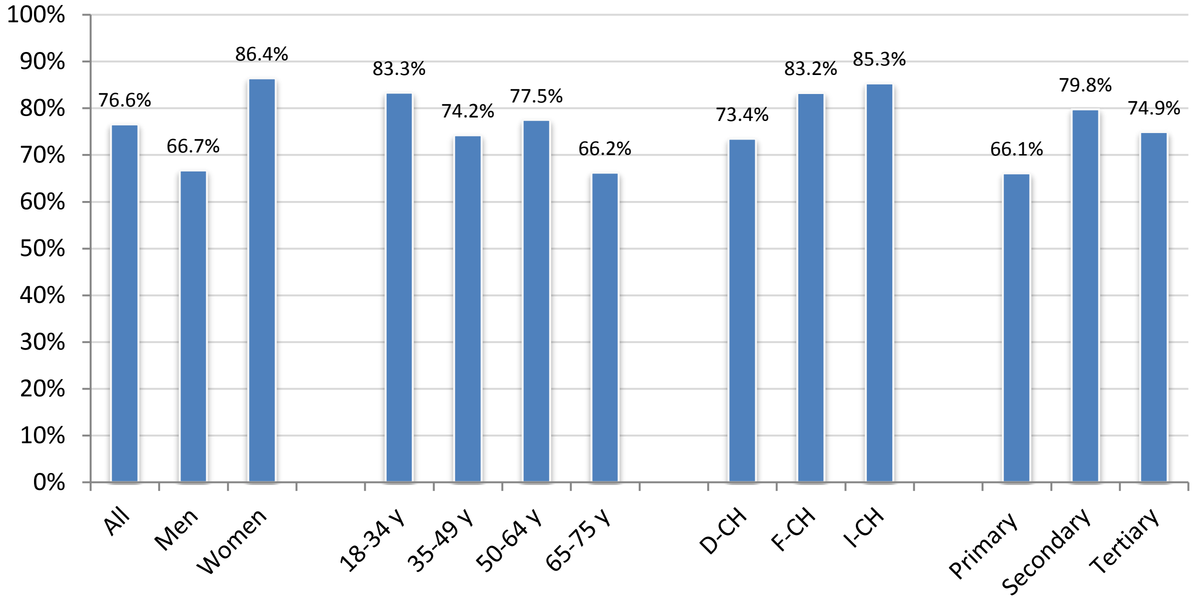

Figure 35 Percentage (%) of people having heard about the Swiss food pyramid, overall, by sex, age groups, linguistic region and educational level.

Asked about the “5 per day” campaign, 65% of people responded to have already heard about it, particularly people residing in F-CH (87%) (Figure 36). Again, more women than men and people with higher education said to be aware about the campaign. Among all people having heard about the campaign, 90% indicated the correct meaning of “Eat three servings of vegetables and two servings of fruits per day”, in F-CH even 97.5% (D-CH 87%; I-CH 80%) did know.

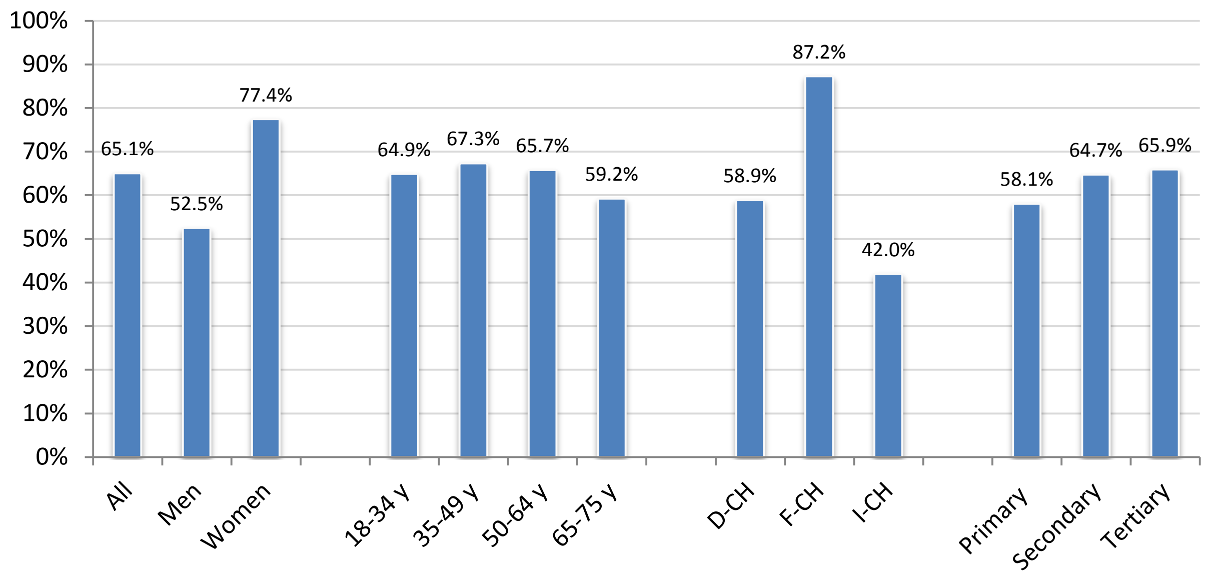

Figure 36 Percentage (%) of people having heard about the 5-a-day campaign, overall, by sex, age groups, linguistic region and educational level.

As the questionnaire was completed at home prior to the appointment in a study center, it is possible that a certain percentage of people checked on the internet about 'Swiss food pyramid' and '5-a-day'.

3.3.2. Cooking and eating habits

Cooking habits

When asked about cooking hot meals at home during a usual week, 35% report to never cook a hot meal themselves for lunches and 19% for dinners (Figure 37). Expected sex differences were observed with 45% of men vs 25% of women reporting to never cook a hot lunch themselves at home, whereas the prevalences were 27% and 10%, respectively, for dinner (Figure 37). A sharp contrast was observed between lunch and dinner with respect to age groups. About one third of participants never cook a hot lunch at home themselves irrespective of age group, while much less young people (15% for the 18-34 years old group, 13% for the 35-49 years old group) than older people (35% for the 65-75 years old group) report never to cook a hot dinner at home themselves. It seems that people from D-CH less frequently cook hot lunches themselves at home than people from F-CH or I-CH.

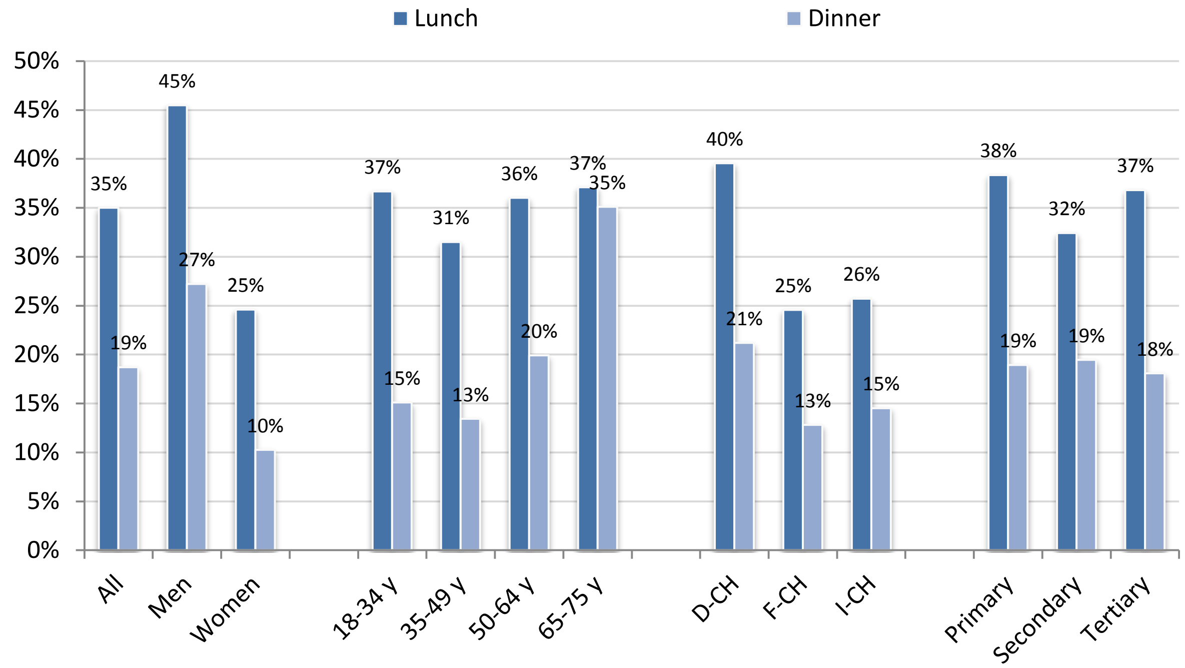

Figure 37 Relative frequencies (%) of never self-cooking hot meals at home for lunch or for dinner, overall, by sex, age groups, linguistic region and educational level.

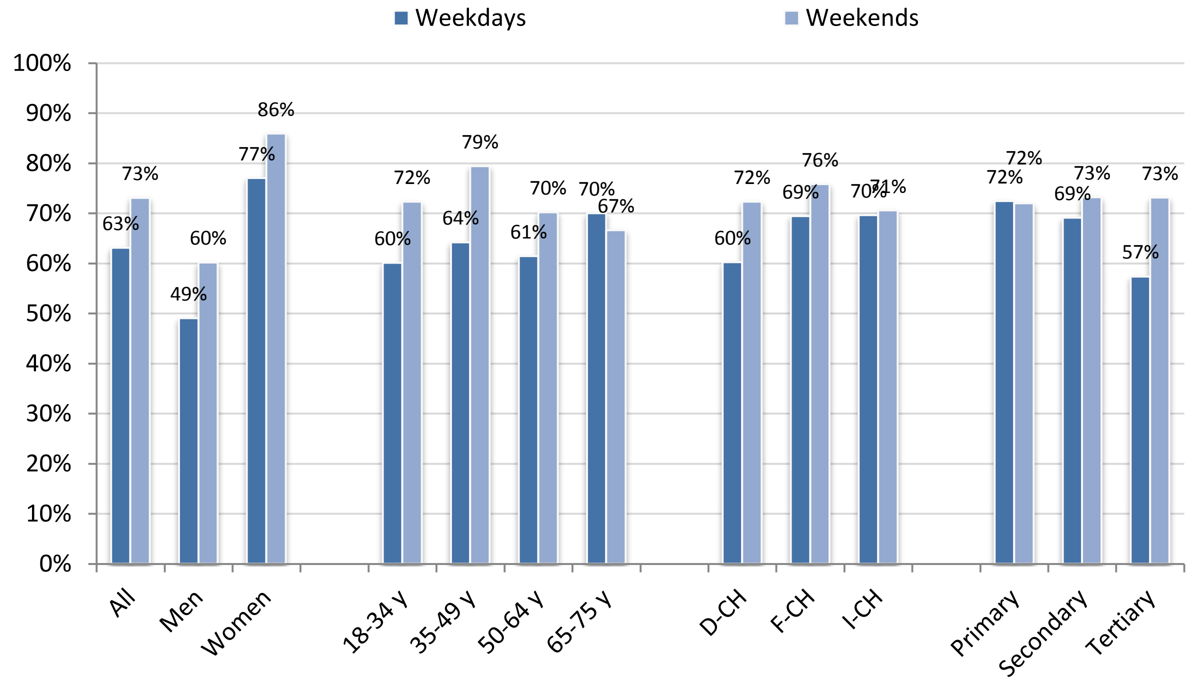

Figure 38 Percentage (%) of days with self-cooking hot meals at home during weekdays (Monday to Friday) and weekends (Saturday and Sunday), overall, by sex, age groups, linguistic region and educational level.

When exploring separately weekend days and other weekdays (i.e. working days), we calculated the proportion of days when participants reported to self-cook a hot meal (either at lunch or at dinner) and expressed it in percentages. Self-cooking hot meals at home was more frequent on weekends days (73% of days) than on other weekdays (i.e. Monday to Friday) (63% of days) (Figure 38). Women, on average, cook more hot meals themselves than men. There is more difference between working days and weekend days for young people than for older people, who are usually retired. People with a tertiary educational level cook more hot meals themselves on weekends than during working days.

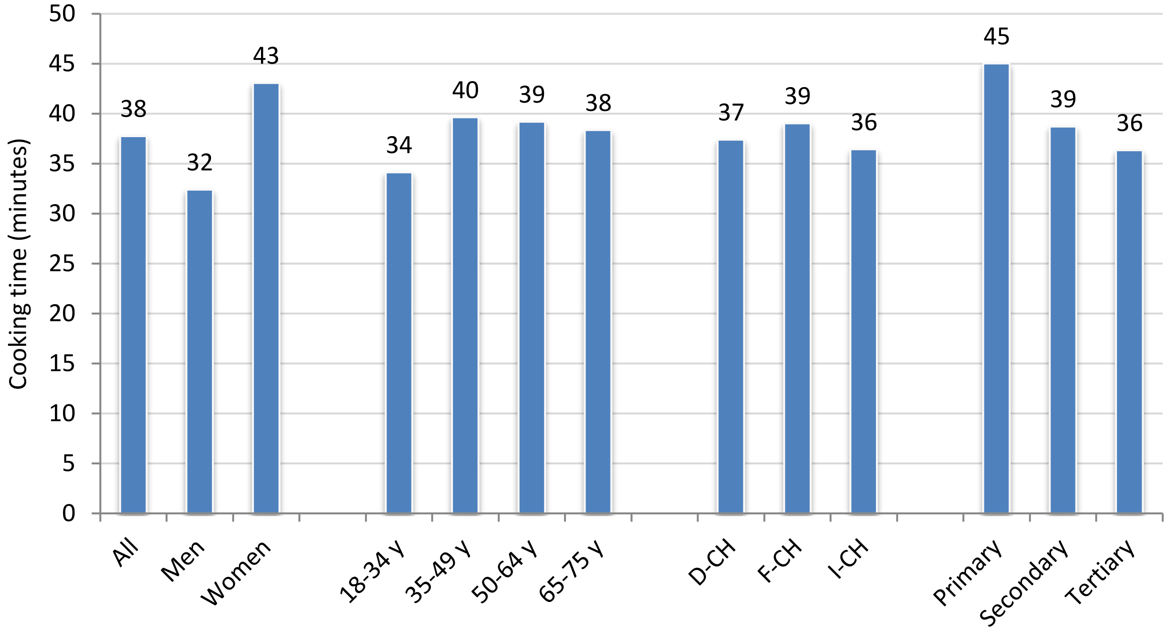

Figure 39 Average time (in minutes) spent to prepare hot meals, overall, by sex, age groups, linguistic region and educational level.

The average reported time spent cooking hot meals was 38 minutes overall, but it was longer for women (43 minutes) than for men (32 minutes) (Figure 39). We observed little differences across linguistic regions or age groups, but more contrast was found across educational levels (45 minutes for primary, 39 minutes for secondary and 36 minutes for tertiary level). More women spend a long time, defined as more than 40 minutes, cooking hot meals than men (50% vs 31%, respectively) (Figure 40). Spending a long time cooking a hot meal was more frequently reported by people with primary educational level (62%) than by those with secondary (44%) or tertiary (36%) educational levels (Figure 40).

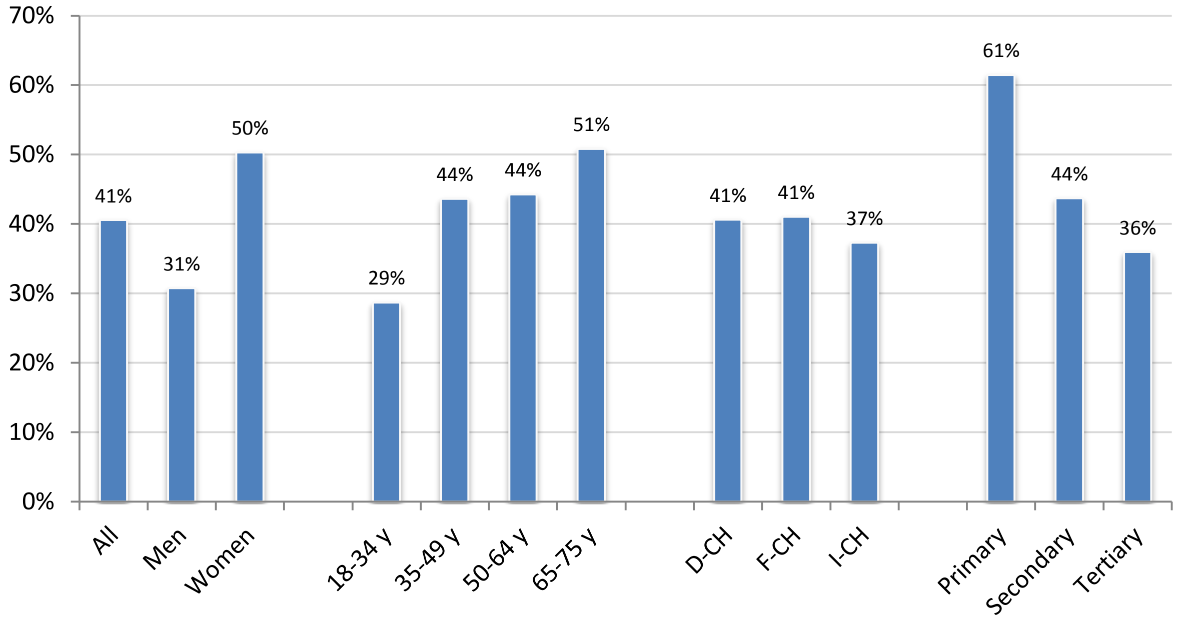

Figure 40 Relative frequencies (%) of spending a long time (i.e. >40 minutes) cooking hot meals, overall, by sex, age, linguistic region and educational level.

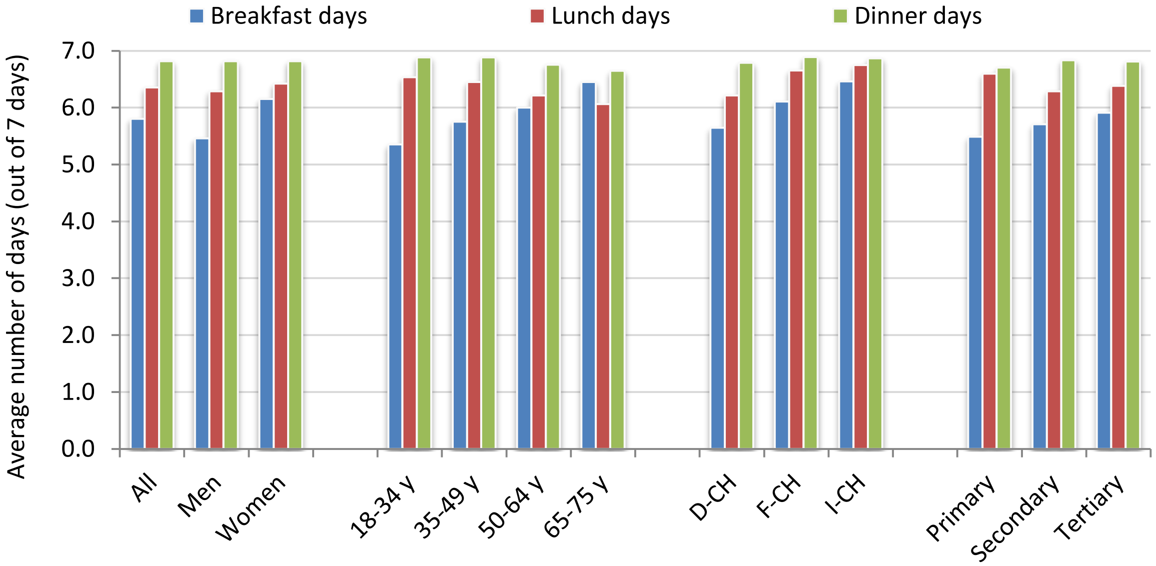

Figure 41 Average number of days (out of 7 days) having breakfast, lunch or dinner, overall, by sex, age groups, linguistic region and educational level.

Participants were asked to report whether or not they take each meal (breakfast, lunch or dinner) in a usual week, and if they take it, whether it is at home or outside of home. To describe behaviors related to the structure of meals in the population, we explored the average number of days in a week people report to have breakfast, lunch or dinner (Figure 41). The average number of days in a week (max. 7 days) that people report to take a specific meal was 5.8 for breakfast, 6.4 for lunch and 6.8 for dinner.

Women more frequently take their breakfast than men (6.2 vs 5.5 out of 7 days, respectively), whereas no such difference is observed for dinner (6.8 vs 6.8 out of 7 days, respectively). The differences across age groups strongly differed by meal type: the average number of breakfast days was 5.4, 5.8, 6.0 and 6.5 days for the 18-34, 35-49, 50-64 and 65-75 year old groups, respectively. By contrast, the average number of dinner days slightly decreased with increasing age, with 6.9, 6.9, 6.8 and 6.6 out of 7 days for the 18-34, 35-49, 50-64 and 65-75 year old groups, respectively. Breakfast is less frequently taken in D-CH (5.6 days) than in F-CH (6.1 days) or in I-CH (6.5 days). A similar, although less marked, trend across linguistic regions was observed for lunch.

Overall, 5% report never to have breakfast, 2% never to have lunch and 1% never to have dinner in a week (Figure 42). Lunch skipping is very rare among young people (0.4% in the 18-34 years old group) and much more common in older people (8.3% in the 65-75 year old group). Breakfast is more frequently skipped in D-CH (5.7%) than in F-CH (4.4%) or in I-CH (3.1%) (Figure 43). Substantial differences were also observed across educational levels: breakfast is always skipped by 10.8% of people with primary education, 6.4% with secondary education and 3.7% with tertiary education (Figure 44).

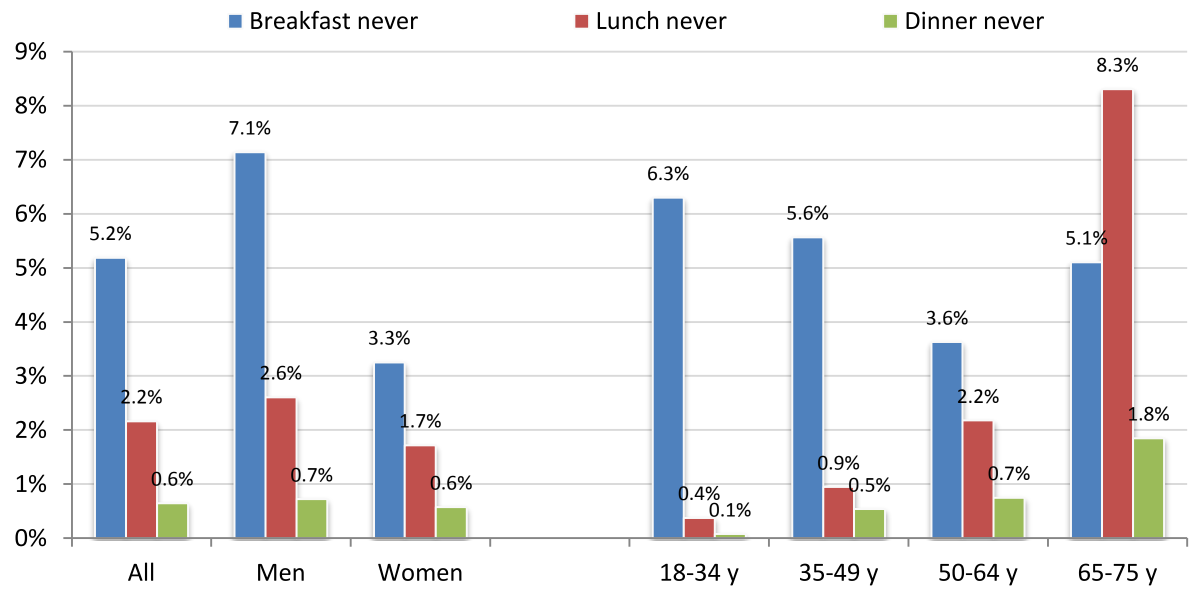

Figure 42 Relative frequencies (%) of never having breakfast, lunch or dinner, overall, by sex and age groups.

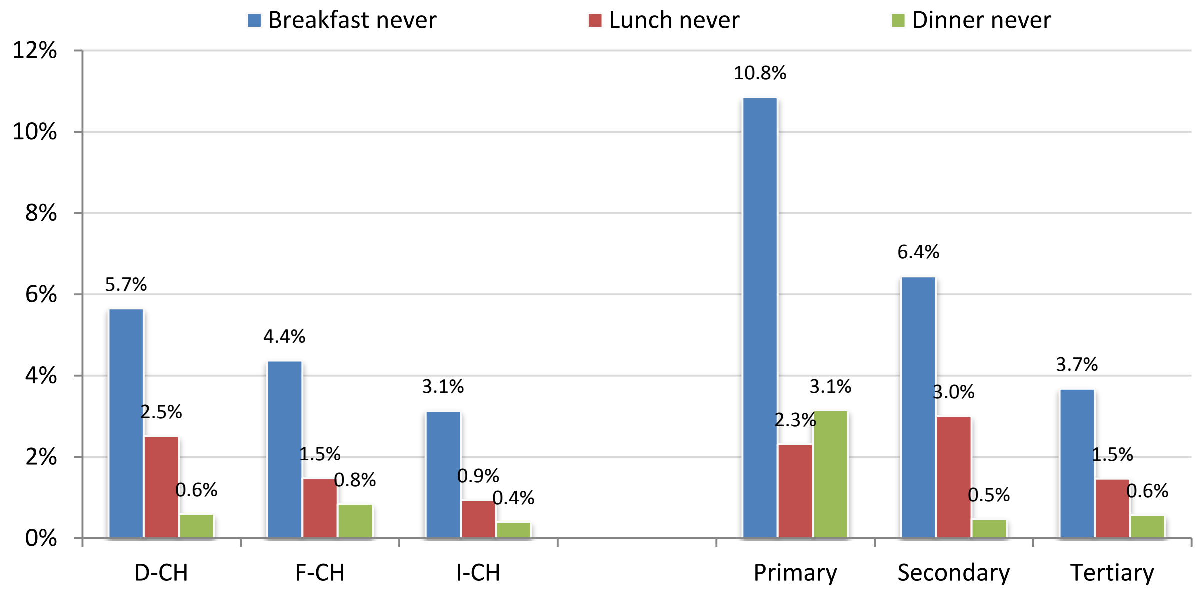

Figure 43 Relative frequencies (%) of never having breakfast, lunch or dinner, by linguistic region and educational level.

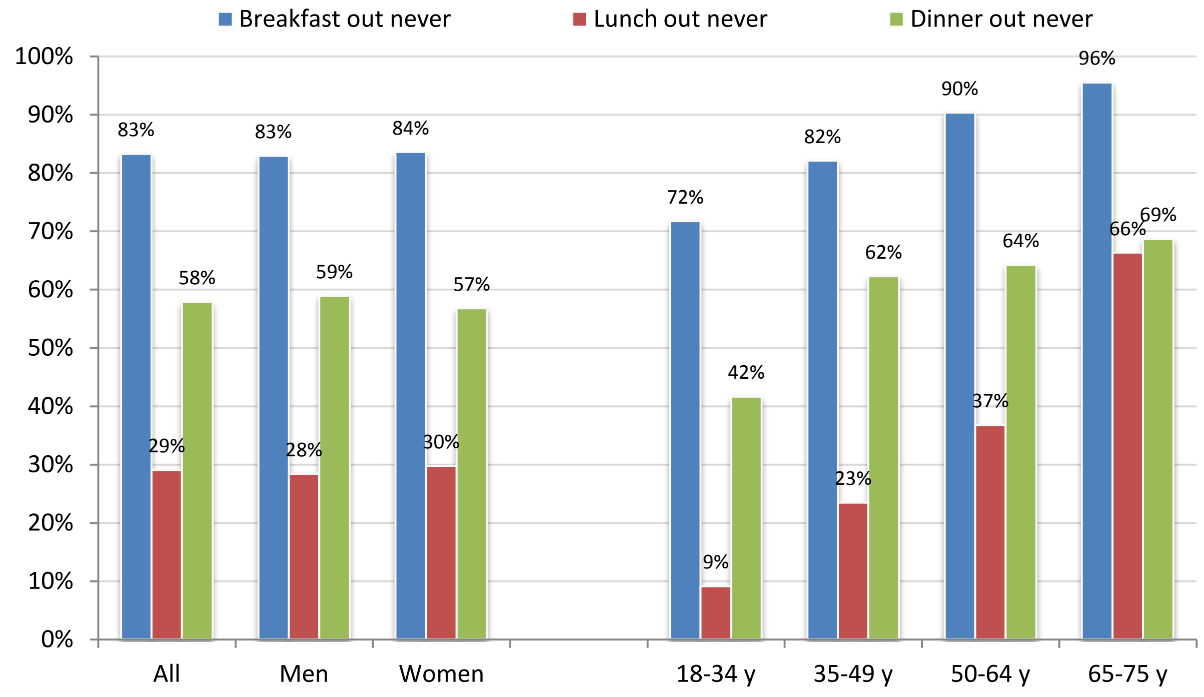

Figure 44 Relative frequencies (%) of never having breakfast, lunch or dinner out of home, overall, by sex and age groups.

The majority of people never have their breakfast out of home (83%), with similar results in men and women (Figure 44). Slightly less than one third never have lunch out of home, whereas 58% report never to have dinner out of home. Young people more frequently have breakfast, lunch or dinner out of home than older people. Regional differences were modest (Figure 45). Compared to people with higher educational levels, people with primary education less frequently have breakfast, lunch or dinner out of home (Figure 45).

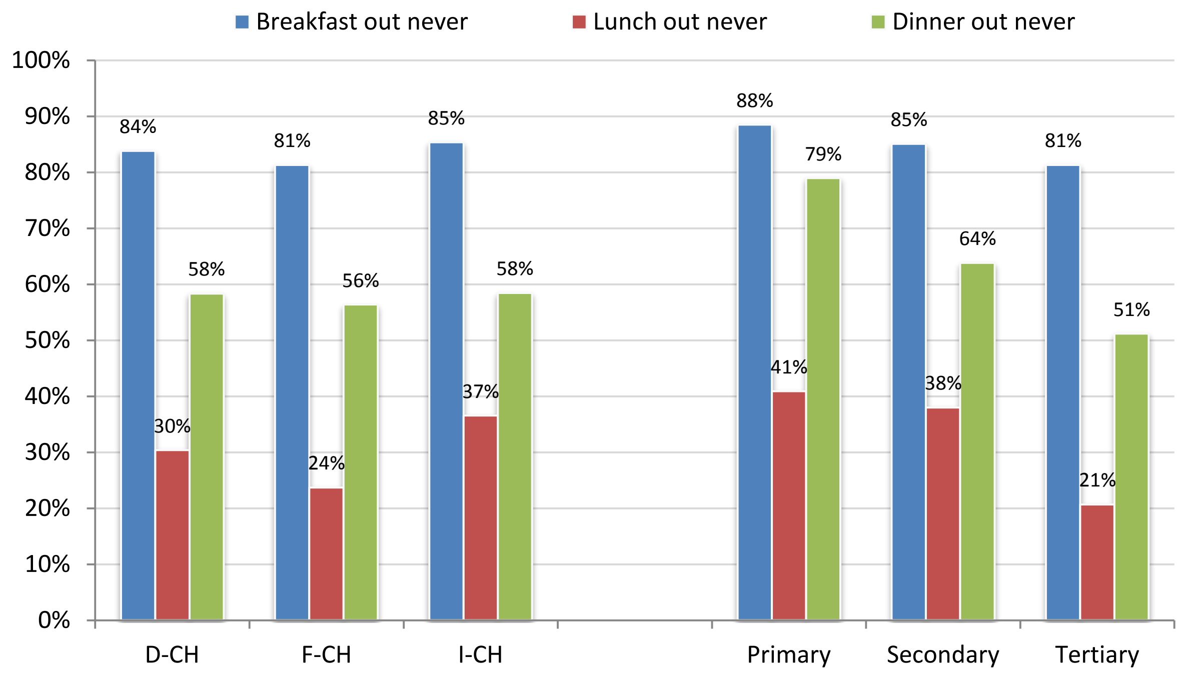

Figure 45 Relative frequencies (%) of never having breakfast, lunch or dinner out of home, by linguistic region and educational level.

When asked about whether they take a given meal at home alone, breakfast was more frequently concerned than lunch or dinner both during weekdays (Figure 46) and weekend days (Figure 47). For breakfast, important regional differences were observed with 47% of people in I-CH reporting to always take breakfast at home alone during weekdays (Figure 46) and 35% during weekend days (Figure 47), compared to 28% and 15% in D-CH and 33% and 23% in F-CH, respectively. For lunch and dinner, taking a given meal at home alone was more frequent in the older age groups than in the younger ones.

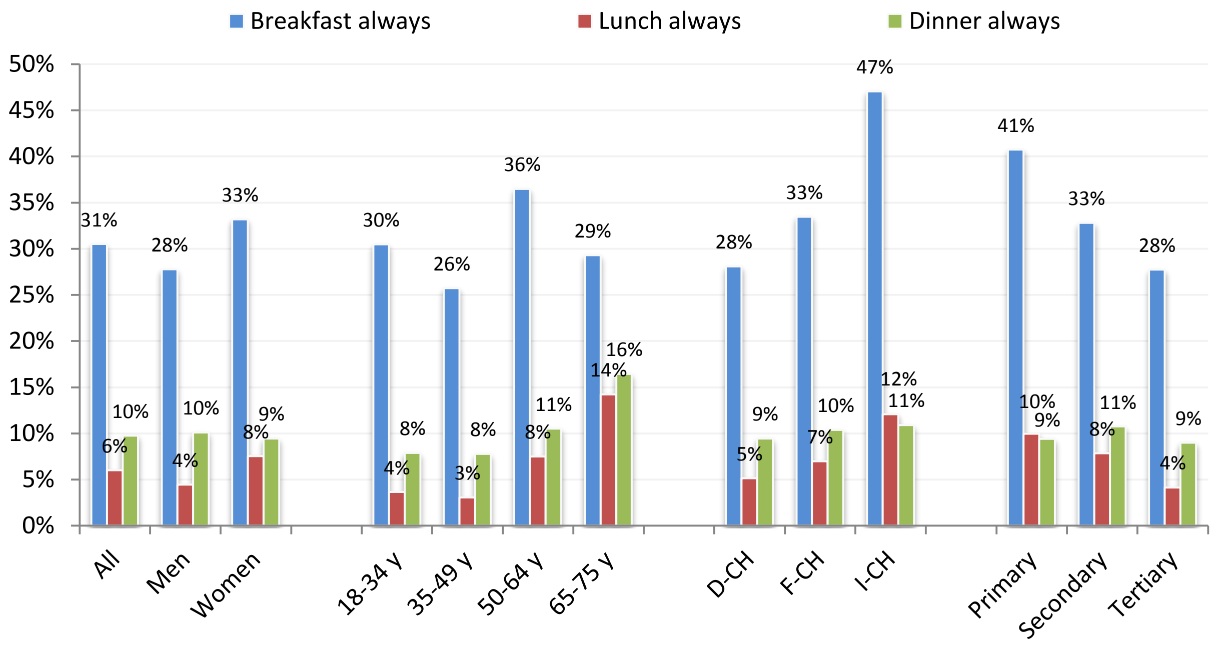

Figure 46 Relative frequencies (%) of always having breakfast, lunch or dinner at home alone during weekdays, overall, by sex, age, linguistic region and educational level.

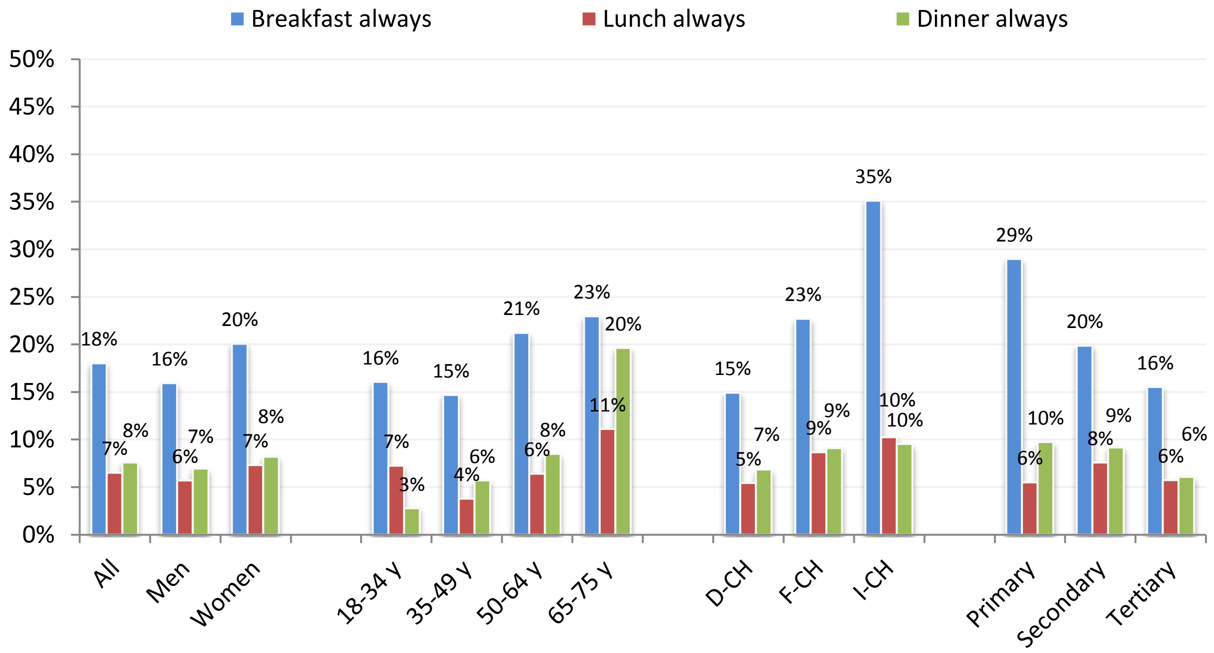

Figure 47 Relative frequencies (%) of always having breakfast, lunch or dinner at home alone during weekends, overall, by sex, age, linguistic region and educational level.

Snacking

People consume foods (including drinks) during the traditional meals of breakfast, lunch and dinner but also outside/between these meals. These eating occasions between main meals are called “snacks” (in German ‘Zwischenmahlzeiten’) and we use the term “snacking” as suggested by Hess et al. (33) when referring to the “act of eating a snack, regardless of whether healthful choices or ‘snack foods’ are consumed”. The latter are characterized as energy-dense, nutrient-poor foods (including drinks) high in sodium, sugar and/or fat (33). In Switzerland three snacks may be considered traditional, at recess/coffee breaks at around 9AM and 4PM, but also in leisure time after dinner.

Figure 48 therefore shows snacking of at least three times at any day, further comparing working days (Monday to Friday) and weekend days (Saturday and Sunday). To note, the question focused on *eating a solid food during snack; drinks are not considered*. About a third of the respondents reported to eat at least three times a day a snack, with barely any difference between men and women. Overall and for almost all selected strata slightly more people reported snacking on weekend days than on the other week (i.e. usually working days). Exceptions were people from I-CH, those with primary education and the oldest age group, who may probably focus more on main meals with the family on weekends. Still, differences are minor.

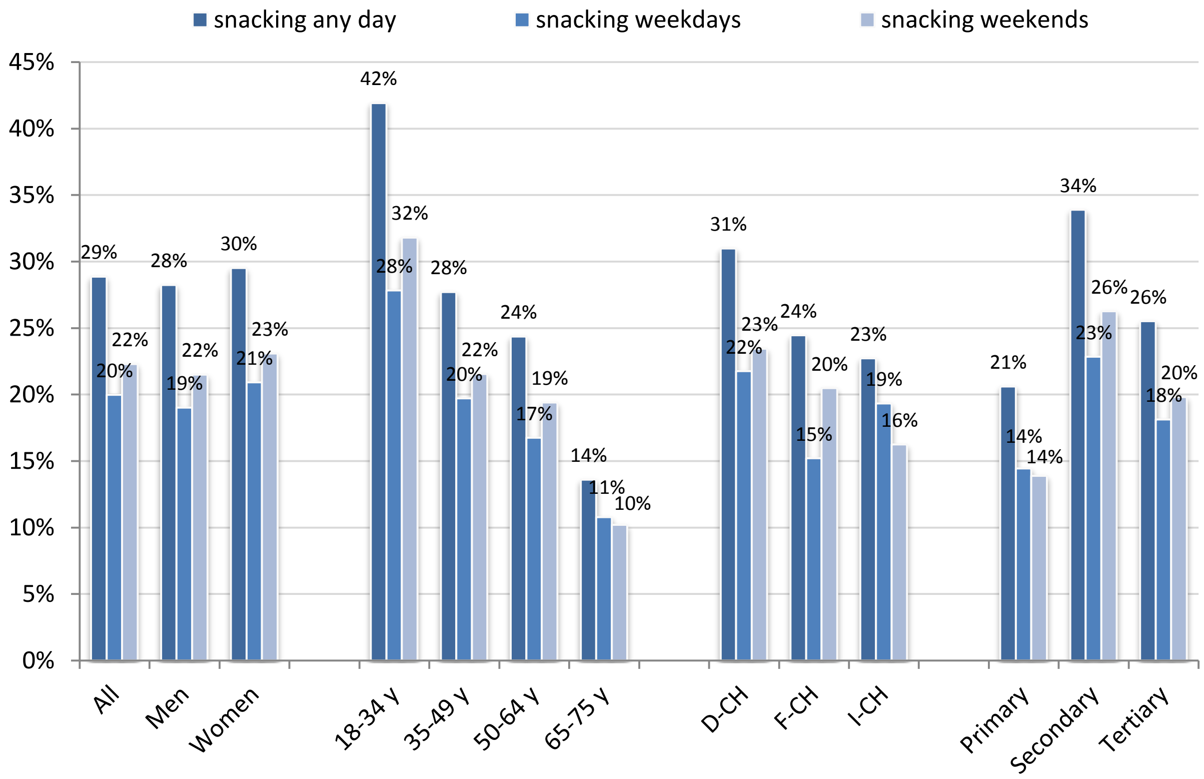

Figure 48 Snacking (solid foods) at least 3 times a day any day, during weekdays or weekend days, overall, by sex, age groups, linguistic region and educational level.

Snacking solid foods at least three times a day was most prevalent in D-CH (31%), followed by F-CH (24.5%) and I-CH (23%). We observed a clear decrease in the frequency of snacking with age, with 42% of the younger people (18-34 years old) reporting to take regular snacks compared to 28% and 24% for the people age 35-64 years, and only 14% for the retired people age 65-75 years. However, the present data

do not allow any qualitative statement about the consumed solid foods; to this end more sophisticated analysis of the 24HDR is needed.

A proportion of 17% (weekday) and 14% (weekend) of respondents stated to never eat solid food snacks during a habitual week (data not shown). By contrast, only 1.4% of the population reported never to consume beverages between main meals/at snacks. For those who reported to consume beverages between meals, the most popular types of drinks were water [tap water, mineral water with or without gas and flavored water] (93.6%), coffee [expresso, cappuccino, latte, etc] (73.0%), tea [black or green tea, herbal teas] (56.7%), alcoholic beverages [wine, beer, spirits] (31.7%), juices [fruits or vegetables] (25.5%), soft drinks [diet or regular cola, Pepsi®, red or blue Rivella®, ice tea, etc] (19.6%) and milk [milk, Ovomaltine®, energy milks, etc] (15.6%).

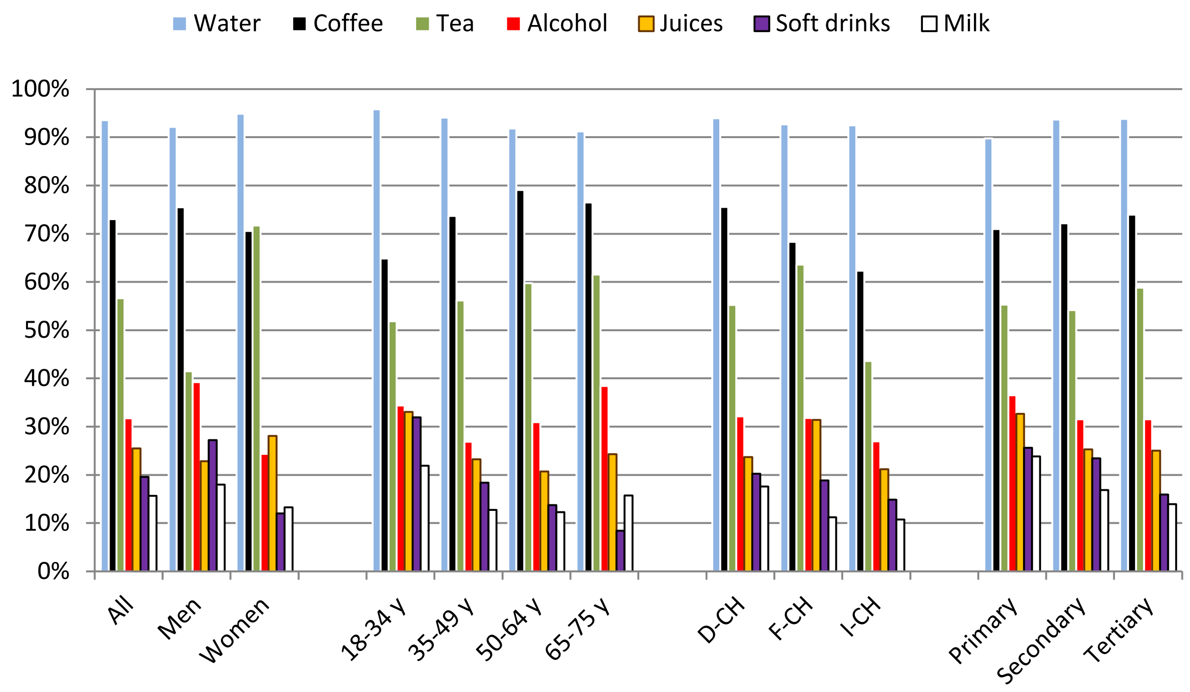

Figure 49 Snacking (drinks) – overall, by sex, age groups, linguistic regions and educational levels.

By far, the most commonly consumed drink between meals is water, across all strata (Figure 49). Consumption of coffee during snacks increases with age and is more common in the German-speaking part than the other linguistic regions of Switzerland (Figure 30). We observed a substantial sex-difference for the consumption of tea during snacks (41% of men vs 72% of women) as well as regional differences (55% in German-speaking, 64% in French-speaking and 44% in Italian-speaking regions) (Figure 49). We found sex differences (39% of men and 24% of women) and age differences (34%, 27%, 31% and 38% for 18-34, 35-49, 50—64 and 65-75 years old, respectively) for the consumption of alcohol between meals. Juices, when including both fruit and vegetable juices, were more commonly reported by people in the younger age group than by the other age groups. For soft drinks (Figure 49), 20% overall reported to consume them out of meals, with a much higher prevalence in men (27%) than in women (12%) and with a steep age-related decrease (32%, 18%, 14%, 8% for 18-34, 35-49, 50—64 and 65-75 years old, respectively).

Discretionary salt use and type of salt used at home

High salt consumption is recognized as one of several lifestyle risk factors for developing high blood pressure. Hypertension is a major modifiable risk factor for cardiovascular disease, including stroke and heart attack (34, 35). Although there is a controversy regarding the target for dietary recommendations, because the amount of dietary salt intake associated with the lowest all-cause and cardiovascular mortality is unclear, the evidence that very high salt intake is detrimental to health is undisputed. In earlier studies, it has been shown that the Swiss population has salt intakes above recommendation (36, 37) and major salt intake sources were identified (36). The salt added at the table (salt shaker) is an important source of sodium intake which could be reduced directly by every person unlike the content of salt in processed foods, which depends on action by the food industry. Salt is also used as a carrier of iodine and fluorine in Switzerland to prevent nutritional deficiencies and their adverse health consequences (38).

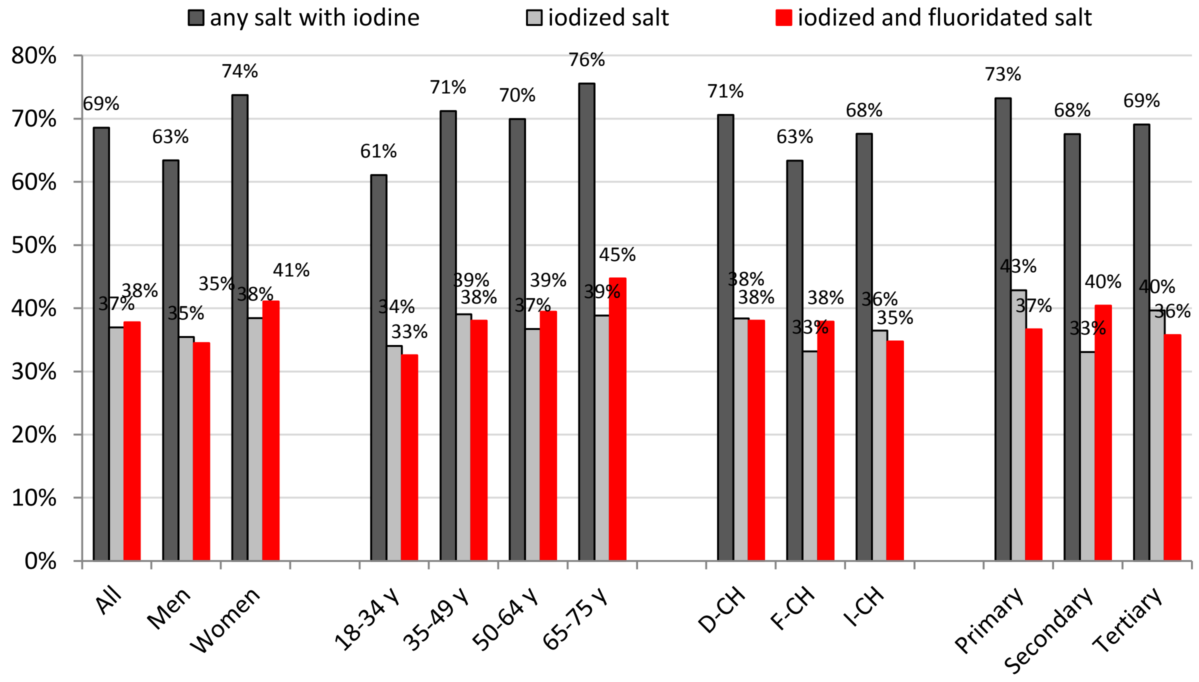

Figure 50 Type of salt use at home, overall, by sex, age groups, linguistic and region educational level.

Figure 50 gives the percentages of the population using any type of iodized salt at home [i.e. the sum of iodized salt and combined salt], only iodized salt or combined iodized and fluoridated salt. Participants could report the use of one or several types of salt at home. Overall, 69% of the population uses some type of iodized salt at home, with a higher percentage of women (74%) than men (63%). The percentage of the population reporting to use a specific type of salt at home was 37.0% for only iodized salt and 37.8% for iodized and fluoridated salt, which is in line with results from the Swiss Salt Study (36.3% and 31.4%, respectively) (37).

We observed some differences in discretionary salt use at home (Figure 51). Overall, 10% of respondents indicated to use the salt shaker during at least 6 out of 10 meals (often, always). This is slightly higher

that what was observed (7.4%) in the Swiss Salt Study (37). The higher the educational level the more frequent was adding salt at the table and more men (53%) than women (42%) reported to do so. Slightly more persons in F-CH than in the other linguistic regions said to add salt at the table when eating at home (Figure 52).

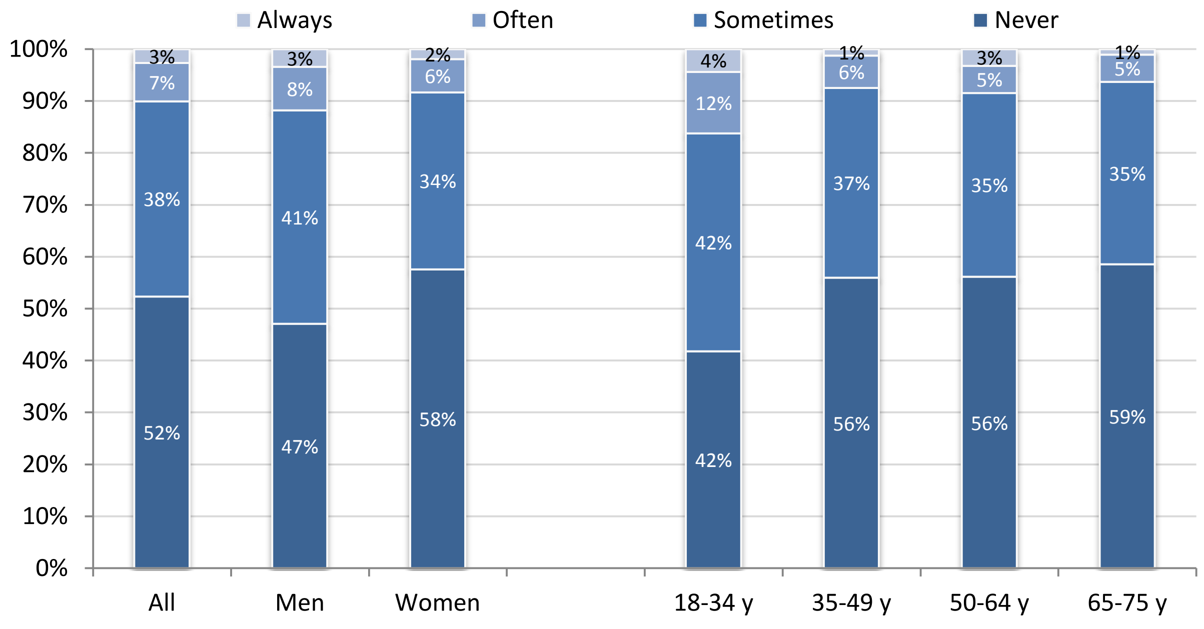

Figure 51 Discretionary salt use at home, overall, by sex and age groups.

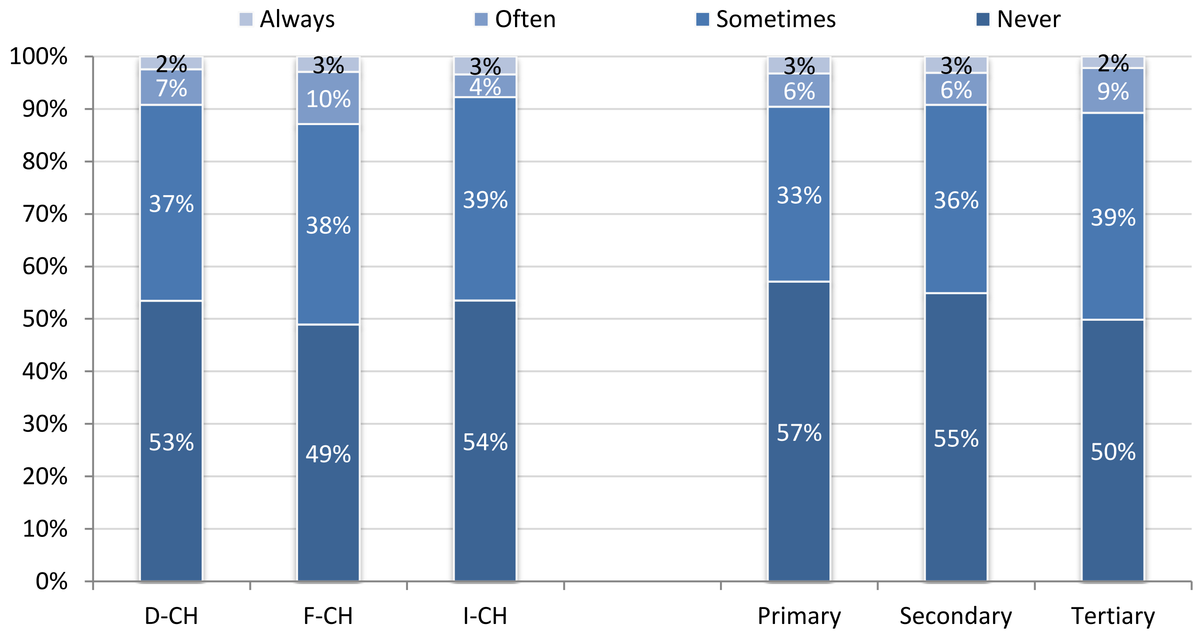

Figure 52 Discretionary salt use at home, overall, by linguistic region and educational level.

The picture of adding salt at the table when eating out of home (restaurant etc.) is quite similar, but overall more people said to never use the salt shaker outside home (Figure 53, Figure 54).

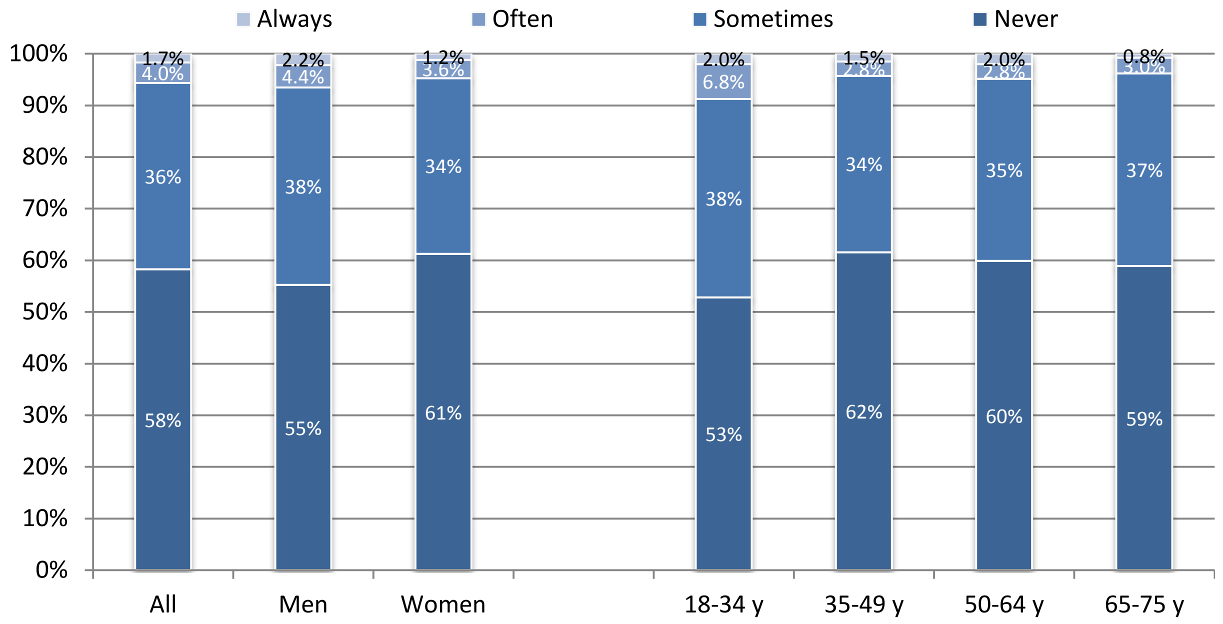

Figure 53 Discretionary salt use outside home, overall, by sex and age groups.

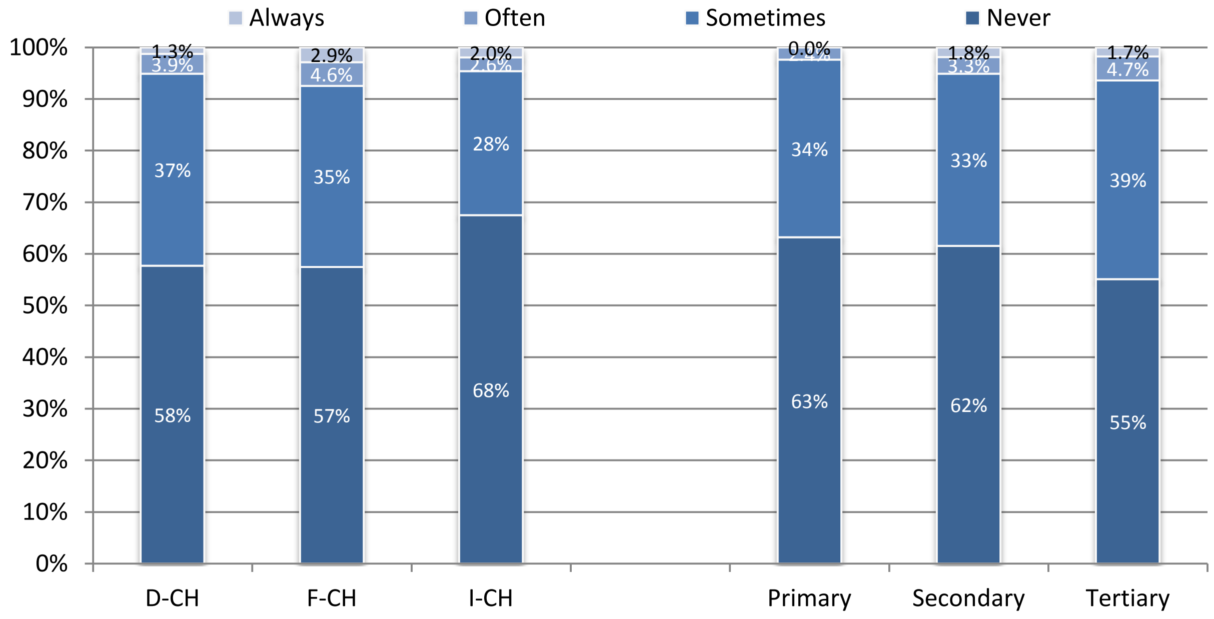

Figure 54 Discretionary salt use outside home, by linguistic region and educational level.

3.4. Physical activity and sedentarity

3.4.1. Physical activity levels

The Federal Statistics Office categorized reported physical activity levels from the Swiss Health Survey 2012 into 5 categories: inactive, partly inactive, irregularly active, regularly active and trained. The categories were defined as follows:

- **inactive:** intensive physical activity (i.e. sufficient to work out sweat) less than once a week or <30 minutes of moderate physical activity (i.e. sufficient to run out of breath) per week.
- **partly active:** 30-149 minutes of moderate physical activity per week or intensive activity (i.e. sufficient to work out sweat) once a week.
- **irregularly active:** at least 150 minutes of moderate physical activity (i.e. sufficient to run out of breath) or 2 days of intensive physical activity (i.e. sufficient to work out sweat).
- **regularly active:** ≥30 minutes of moderate physical activity (i.e. sufficient to run out of breath) at least 5 days per week.
- **trained:** intensive physical activity (i.e. sufficient to break into a sweat) at least 3 days per week.

People are considered to be sufficiently active if they carry out an intensive physical activity (i.e. sufficient to break into a sweat) at least at least twice a week or if they carry out a moderate physical activity (i.e. sufficient to run out of breath) at least 150 minutes per week. The combination of the categories trained, regularly active and irregularly active provides the proportion of people who are considered to be sufficiently active.

According to menuCH results, 87% of the Swiss population (86% of men and 88% of women) aged 18 to 75 years meet current recommendations for sufficient physical activity level. Overall, 25% report to be trained (28% of men and 22% of women) (Figure 55). More than 60% of people are at least regularly active. In any of the explored strata, the prevalence of inactivity was less than 5%. Sufficient data to generate these categories were available for 1550 participants, representing a population of 3’489’571 Swiss residents aged 18 to 75 years.

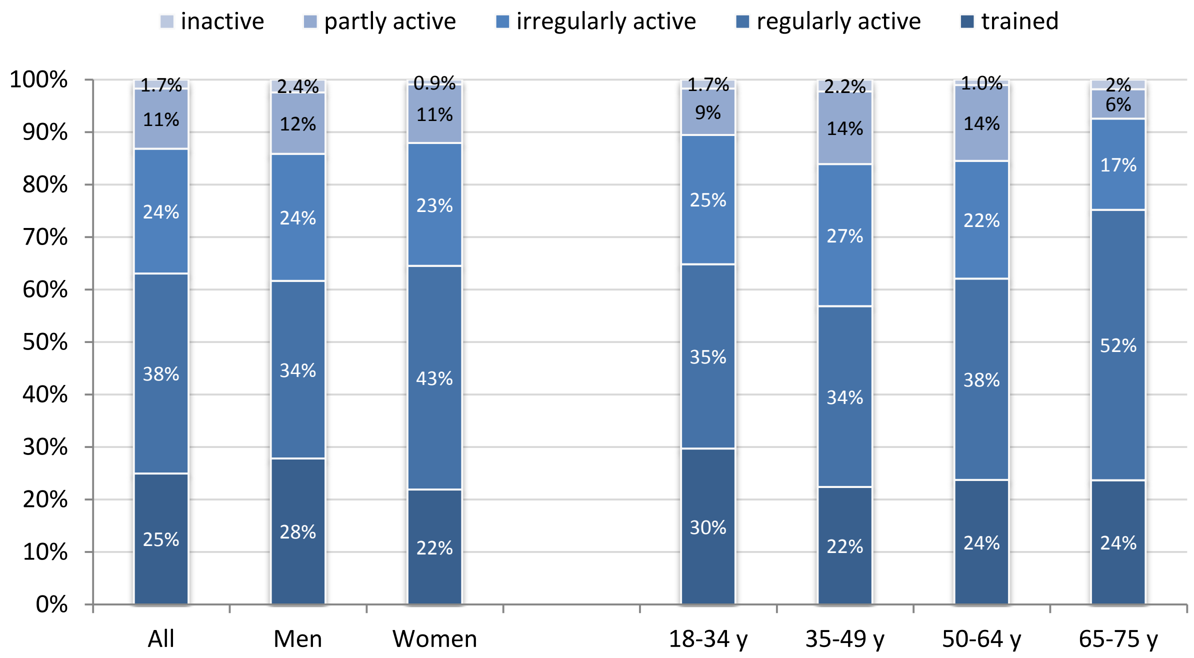

Figure 55 Physical activity categories derived from IPAQ-SF questionnaire, among people who know, overall, by sex and age groups.

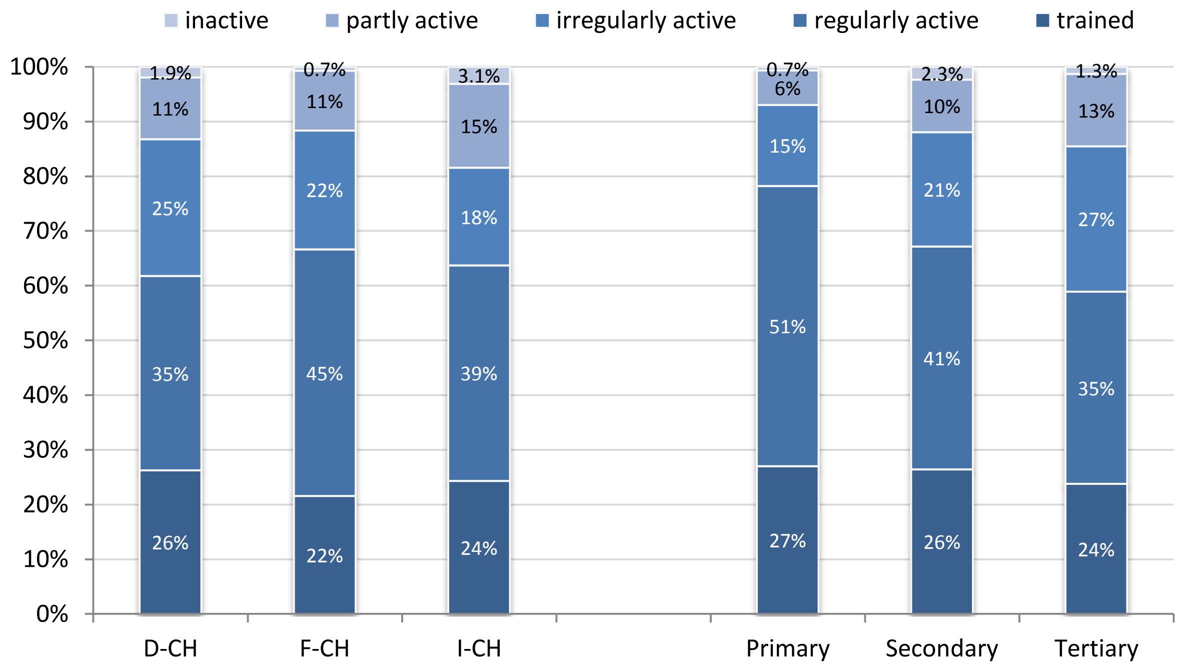

Figure 56 Physical activity categories derived from IPAQ-SF questionnaire, among people who know, by linguistic region and educational level.

3.4.2. Walking time

Overall, 48% (44% of men and 52% of women) report walking at least 30 minutes per day, more than 5 days a week, which means at least 150 minutes walking per week (Figure 57). People aged 65-75 years report longer walking time (68% with over 150 minutes per week) than the other age groups. There were little differences across linguistic regions.

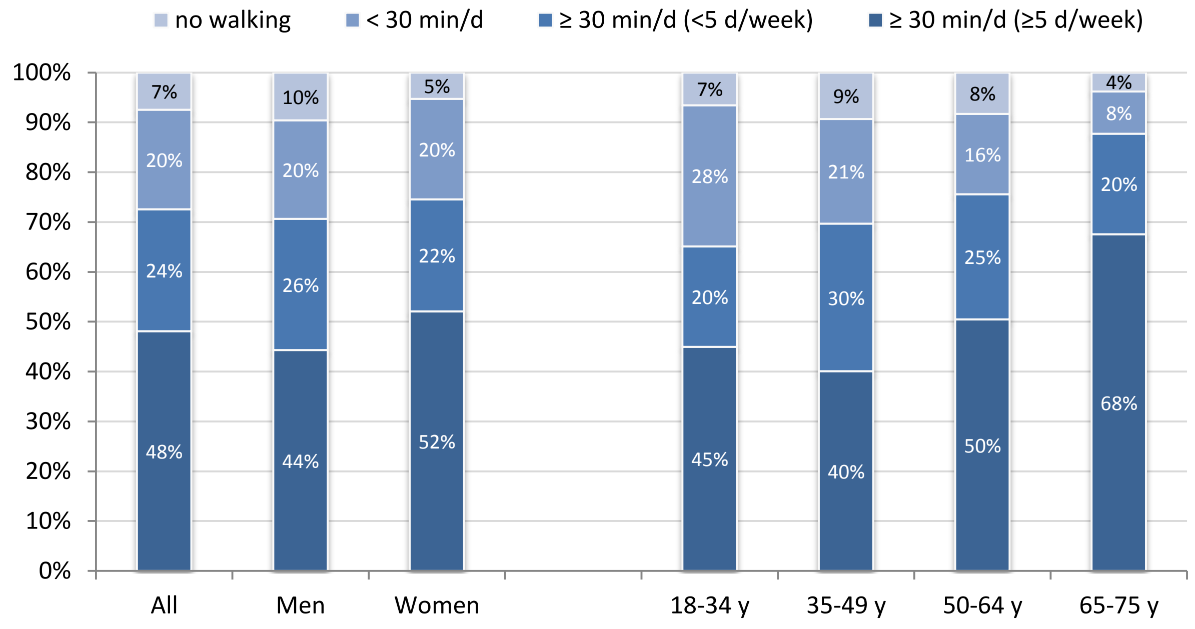

Figure 57 Relative frequencies of walking time categories, overall, by sex and age groups.

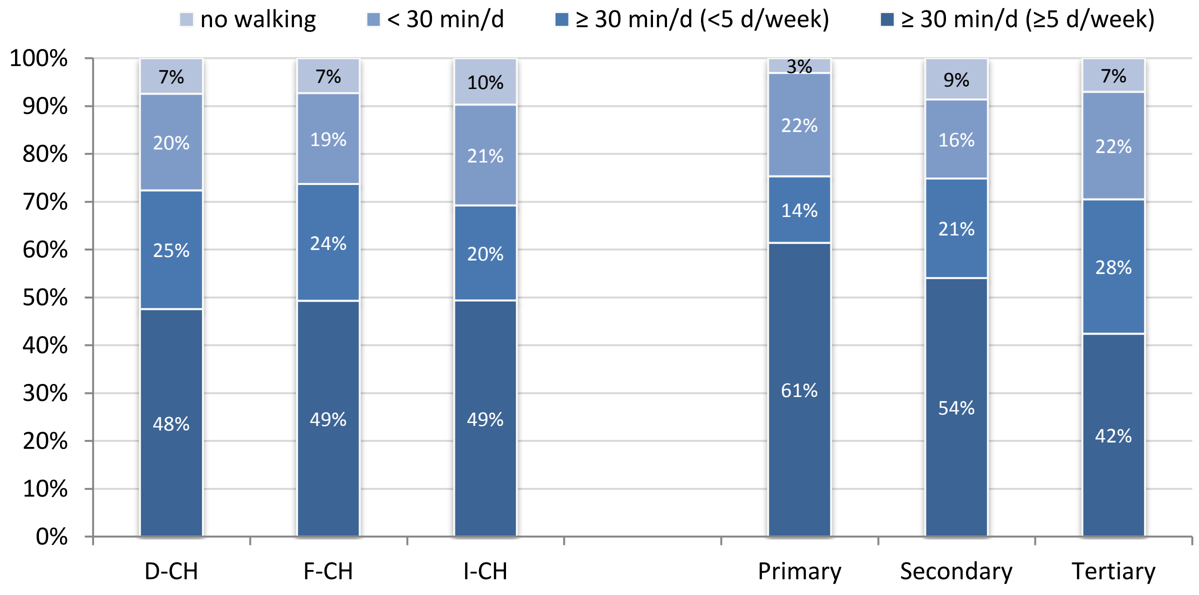

Figure 58 Relative frequencies of walking time categories, by linguistic region and educational level.

3.4.3. Sedentary lifestyle

The average sitting or lying time, without sleeping, during weekdays was split into 4 categories: 2h30 minutes or less, 2h31 to 5h30, 5h31 to 8h30 and more than 8h30. The relative frequencies in each of these categories are presented overall and by selected strata in Figure 59. Overall, 31% of the population spends more than 8h30 sitting or lying on average during weekdays. A clear decreasing trend of prolonged sitting time is observed across age groups. People with tertiary education level more frequently report prolonged sitting time than people with other education levels.

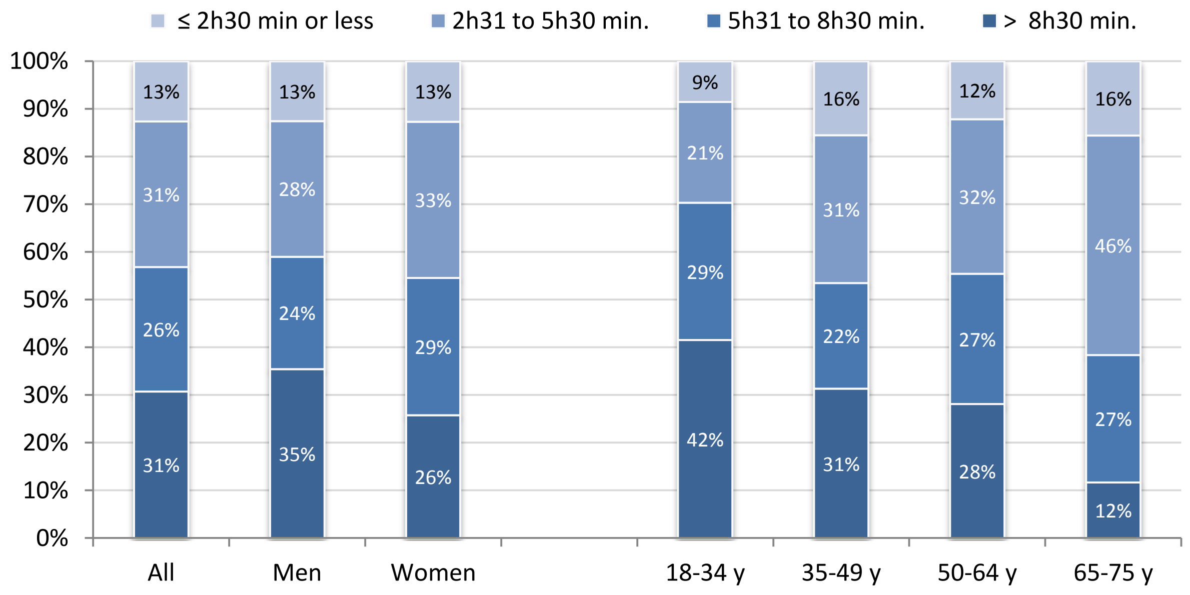

Figure 59 Categories of average sitting or lying time, without sleeping, during weekdays, overall, by sex and age groups.

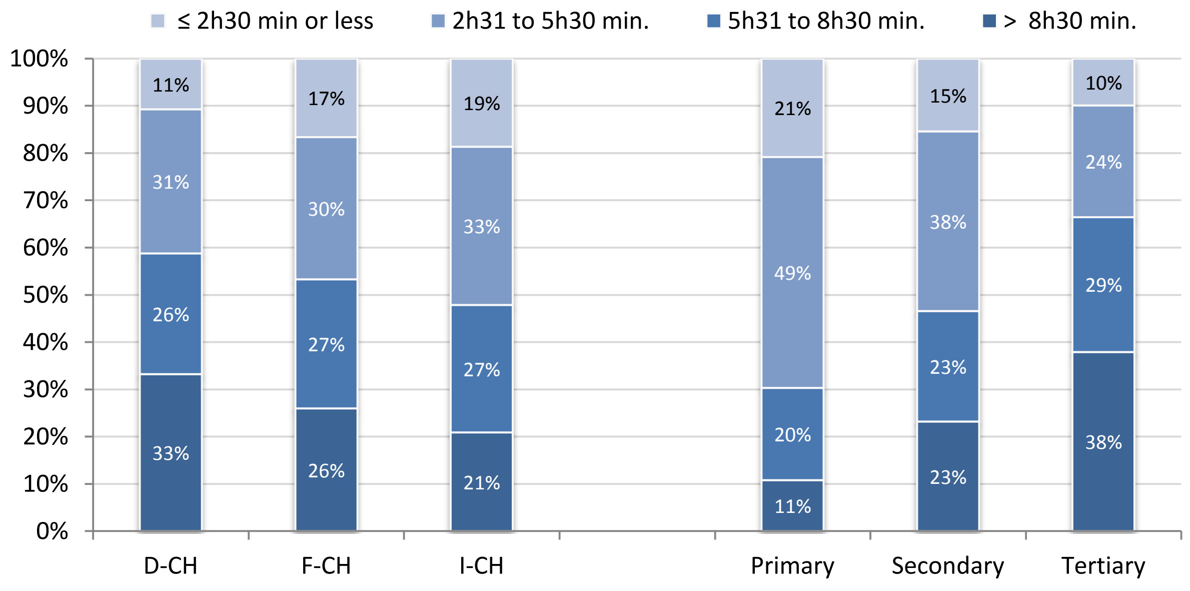

Figure 60 Categories of average sitting or lying time, without sleeping, during weekdays, by linguistic region and educational level.

4. DISCUSSION

The first National Nutrition Survey, menuCH, was a cross-sectional population survey including 2085 adults aged 18 to 75 years residing in the three main linguistic regions of Switzerland. A net response rate of 38% was achieved. The included participants represent a population of 4'627'878 people covering the 11 most populated Swiss cantons.

The survey (menuCH) provides important measured data on anthropometric parameters, as well as questionnaire-based data on diet-related behaviors, which has been tested in a pilot phase. The survey also included two 24-hour dietary recalls, one face-to-face and one telephone interview six weeks later, which are not the focus of the present report.

The distributions by age groups, sex and linguistic regions observed in menuCH were similar to those observed in the Swiss Health Survey 2012. Compared to the Swiss Health Survey 2012, menuCH included a higher proportion of well educated people, half of whom report a university level. Overall, we may assume that the topic of diet and nutrition as well as the request to have an in-person interview attracted more educated people and more health interested participants in menuCH than does a phone-administered general health survey, such as the Swiss Health Survey.

Furthermore, challenging tasks (i.e. to complete a questionnaire and two 24-hour dietary recalls) may have restrained less educated people from participation. Also, participants had to be sufficiently fluent in German, French or Italian to be able to complete the 24-hour dietary recalls with the assistance of a dietician. As a consequence, data included in the present report are likely to inadequately reflect the situation of non-Swiss citizens. The time burden/investment to come to a central mostly urban study center may explain some of the refusals observed in large administrative regions and more rural cantons. It is possible that people with high interest in diet-related issues were more prone to participate than people with less interest in such issues. Healthy participant bias may also have occurred considering that the proportion of daily smokers was lower than the one observed in Swiss Health Survey 2012 and that self-rated health was slightly better than in the Swiss Health Survey 2012.

In menuCH, average BMI was 25.0 kg/m^2, overall. In the population-based Swiss Salt Study (2010-2011), which also relied on measured anthropometric data and included 1505 participants aged 15 years and over, average BMI was 25.2 kg/m^2 (39). In the population-based CoLaus study, including more than 6100 adults residing in the city of Lausanne and aged 35 to 75 years, average BMI at baseline (2003-2006) was 25.8 kg/m^2 (14).

In menuCH, as expected, men had higher average BMI than women (25.9 kg/m^2 vs 24.0 kg/m^2, respectively). In the Swiss Salt Study (37), average BMI was 26.0 kg/m^2 in men and 24.3 kg/m^2 in women. In CoLaus (14), average BMI at baseline was 26.6 kg/m^2 in men and 25.1 kg/m^2 in women. In menuCH, average BMI was higher in older age groups than in younger ones, for both men and women. Similar values and patterns across age groups were reported in the Swiss Salt Study (37). In menuCH, average BMI was similar across linguistic regions, also in line with results from the Swiss Salt Study (37).

In menuCH, the prevalence of obesity was similar in the German-speaking (12.5%) and French-speaking (12.3%) regions and slightly higher (15.6%) in the Italian-speaking region, which somewhat contrasts with findings from the Swiss Salt Study (39) (obesity prevalence: 13.6%, 16.2% and 12.1% in the German-, French- and Italian-speaking regions, respectively). It should however be noted that participation rate in the Swiss Salt Study (10%) was lower than the one observed in menuCH, which resulted, in part, from the fact that participants had to collect urine for 24-hour in the Swiss Salt Study. In menuCH, the prevalence

of obesity tended to be lower in people with high education level compared to those with other educational levels, in line with the Swiss Health Survey 2012 (8).

Although the distributions of self-reported body weight and height were globally very similar to the distributions of measured weight and height, in both men and women, BMI based on self-reported data tended to differ from BMI based on measured data primarily for overweight and obese people, who tend to misperceive and underestimate their weight.

About one third of participants had a waist circumference that places them at increased or substantially increased risk of future ill health. In both men and women, the prevalence of at-risk waist circumference sharply increased with age. Waist circumference is a measure of abdominal obesity that is of particular interest in men to differentiate fat and muscle mass (40). However, it is very challenging to measure WC (41) and time-consuming. Repeated trainings of interviewers/health professionals can ensure low intra-interviewer WC measurement variance but a certain inter-interviewer variance may always persist.

About one third of women, across linguistic regions had a WC that placed them at increased or substantially increased risk of future ill health. This contrasts with what was observed in men, for whom important regional differences were observed. In F-CH about 10 percentage points fewer men were at metabolic risk than in D-CH and I-CH, that is 30% compared to 40%. The difference was particularly visible in the group of men with highly increased risk or having a WC of at least 102 cm. By contrast to these findings, F-CH participants from the Swiss Salt study were shown to be more often at substantial metabolic risk than their regional counterparts (37).

When comparing the distribution of BMI categories between people with normal WC risk category and those with WC that put them at increased or highly increased metabolic risk, we observe that people with WC associated with normal risk are not obese according to BMI classification. In men at increased risk category for WC, less than 4% were not overweight or obese. In women at increased risk category for WC, 20% were normal weight according to BMI. One in five women at increased metabolic risk based on WC is considered as having normal weight according to BMI categories. These results highlight the usefulness of WC to identify increased metabolic risk in women. Longitudinal studies have shown WC to be association with all-cause mortality independently of BMI (42, 43). Yet the choice of the best adiposity measure depends on the outcome of interest (44).

During the 12 months preceding the menuCH survey, about 13% of the population reported to have kept a weight loss diet. In comparison, only 8% of the population reported to be on a weight loss diet the year preceding the Swiss Health Survey 2012 (30). Presumably menuCH attracted slightly more individuals preoccupied with their diet. Having kept a weight loss diet during the past 12 months was much more frequently reported by people from the 18-34 years age group (17%) than by older people (5.4% in the 65-75 years group). Such age difference was observed in men and women.

About one third of participants reported to habitually follow a special diet, but this was more frequent in women than in men and more frequent in the German-speaking regions compared to the two other linguistic regions. Among the special diets reported by a substantial number of participants (e.g. vegetarian, energy restriction, fat restriction, etc), important differences across age groups, linguistic regions and educational levels as well as between men and women were observed. Vegetarian diet, the most commonly reported special diet, was common in the younger age groups, whereas fat restriction diet tended to be frequent in older age groups.

Almost half of the population reported the use of supplements, either vitamins, minerals or a combination of those. Minerals represent the most commonly used supplements. Supplement use was more commonly reported by women (56%) than by men (38%) and by participants from the German-speaking region compared to the two other linguistic regions. Differences by educational level were

small. The very high prevalence of supplement use is in line with other Swiss surveys. A regional survey of a small convenience sample from Lausanne in 2011 found that 49% used one all-in-one supplement (45). A large surveillance project conducted in the same region reported in 2007 that about 20-30% used vitamin-mineral supplements (46). Questions were not identical and product classifications differed. Still, we may assume that supplement use will become increasingly popular in the Swiss population.

A large proportion of participants were aware of food guidelines and campaigns, but regional differences were observed with higher awareness in the French-speaking region for the 5-a-day campaign. As the questionnaire was completed at home prior to the appointment in a study center, we cannot exclude that a certain percentage of people checked on the internet before completing the questionnaire. In comparison, a telephone survey conducted in 2011 in D-CH and F-CH (47) asked more specifically if people had heard or read about the food pyramid of the Swiss Nutrition Society (SNS) or another food pyramid. In total, 81% had heard about any food pyramid, but only 35% of the SNS-pyramid which is supported by the authorities and therefore called “Swiss food pyramid”. Also, more people in F-CH (40%) had been aware about the SNS-pyramid than in D-CH (35%) but overall, 15% less people from F-CH than D-CH had been aware about any pyramid (47). Despite different survey techniques and sample sizes, we have to keep in mind that results from menuCH may not exclusively show awareness about the “Swiss food pyramid” edited by the SNS.

Asked about the “5 per day” campaign, 65% of people responded to have already heard about it, particularly people residing in F-CH (87%). We may assume that the campaign is implemented differently in F-CH and was able to raise the awareness of more people than in D-CH and I-CH. The campaigns from neighboring countries may also influence such awareness and knowledge. Nonetheless, we cannot infer anything about fruit and vegetable consumption from this information. This needs to be further investigated using the collected food consumption data. For the 5-a-day complain, menuCH results were comparable to those reported from the above mentioned telephone survey (47).

Regarding cooking habits, men less frequently report cooking hot meals. Participants less frequently cook a hot meal at lunch than at dinner. Young people often report to cook a hot meal for dinner, which is less the case for older age groups. The average time spent cooking hot meals was 38 minutes overall and was longer for women (43 minutes) than for men (32 minutes), with little differences across linguistic regions. Overall, people appear to rarely skip the main meals (breakfast, lunch and dinner). The most frequently skipped meal was breakfast (5%), in particular in the younger age group, in men and in the German-speaking region.

Slightly less than one third of participants reported to snack solid foods, with little difference between men and women. This was particularly commonly reported by young people and gradually decreased across increasing age. Snacking solid foods appeared as common during weekdays than during weekend days.

Water consumption between meals was reported by more than 90% of the population, across all explored strata. Only 1.4% of the population reported never to consume beverages between main meals/at snacks. Men more commonly report to consume coffee than tea between meals, whereas the proportion was similar in women. About one third of participants report to consume alcohol between meals and this was more frequent in men than in women. Young people more frequently reported to consume soft drinks between meals (about one third) than older age groups. These results highlight the importance of targeting young people to decrease the consumption of energy-dense soft drinks between meals. Consumption of sugar-sweetened beverages is associated with increased risk of type 2 diabetes (48) and long-term weight gain (49). The estimated disease burden attributable to sugar-sweetened beverages is substantial worldwide (50). Juices, when including both fruit and vegetable juices, were more commonly reported by people in the younger age group than by the other age groups. Milk

consumption between meals was also more commonly reported by young people and by people from the German-speaking region.

Two thirds of the population report to use some type of iodized salt at home, with a higher percentage of women than men. Whereas half of people appear to never add salt at the table at home, this was less the case for younger people, compared to older age groups. The regular use of discretionary salt outside of home appears to be rare, being reported by less than 10% of the population.

Physical activity is good for health. According to WHO, insufficient physical activity is responsible for 6% of all deaths worldwide and physical inactivity is ranked as the fourth leading risk factor for global mortality (51). Physical activity prevents chronic diseases across age groups, countries, socio-economic and educational levels as well as BMI categories (51). WHO considers that one third of the world adult population fails to achieve the recommended 150 minutes of moderate-intensity aerobic physical activity or 75 minutes of vigorous intensity per week or a combination of both (51).

Physical activity is difficult to accurately assess, either to capture total physical activity or different intensity levels in day-to-day life and over the long run (52). In population-based studies, questionnaires are the most commonly used form to assess physical activity. The IPAQ questionnaire has been designed to assess physical activity for adults aged 18 to 65 years and captures physical activity in four main domains: (1) during transportation, (2) at work, (3) at home and (4) during leisure time (13). IPAQ has been validated, including its short form (13). In menuCH, the short-form of the IPAQ questionnaire (SF-IPAQ) was used. menuCH data on physical activity level should be interpreted with caution as one fourth of participants had incomplete data from the SF-IPAQ questionnaire. In menuCH, one in four people reported to be physically trained and physical inactivity was very rarely reported.

Overall, 87% of the Swiss population (86% of men and 88% of women) aged 18 to 75 years meet current recommendations for sufficient physical activity level, based on menuCH results. For comparison, 72% of the population (76% of men and 69% of women) meet the recommendations for physical activity in the Swiss Health Survey 2012. Overall, 25% report to be trained (i.e. sufficient to break into a sweat at least 3 days per week and for a total duration of at least 75 minutes), but this tended to be more common in men (28%) than in women (22%). In menuCH, more than 60% of people reported to be at least regularly physically active. Nearly one in two people in the population reported to walk at least 30 minutes per day, more than 5 days a week, which means at least 150 minutes walking per week, and there were little differences across linguistic regions. Highly educated people reported shorter daily walking times than people with secondary or primary educational levels. People aged 65-75 years reported longer walking time (68% with more than 150 minutes per week) than the other age groups. In the younger age categories, less than 50% (45% in the 18-34 years group and 40% in the 35-49 years group) reported to walk at least 30 minutes per day, more than 5 days a week. The latter result underscores the importance of interventions to promote physical activity that target young age groups and professionally active people.

During the past decades, office work has considerably changed, leading to increasingly sedentary behaviors at work. It has been recently shown that women who are physically active do not sit less during the day than women who are less active (53). Information on physical inactivity therefore provides added value to information on physical activity levels. Physical inactivity increases the risk of many non-communicable diseases such as coronary heart disease, type 2 diabetes, selected cancers, as well as all-cause mortality (51). Prolonged sitting time increases cardiometabolic risk and all-cause mortality (54, 55). A dose-response analysis suggests a nonlinear relationship between sedentary time and increased cardiovascular risk, with increased risk only at high levels of sedentarity (56). High levels of physical activity (at least 60 minutes per day) appear to compensate for the increased risk of death associated with prolonged sitting time (57). Experimental studies have shown that breaking up

prolonged sitting time improves metabolic outcomes (58). In menuCH, one third of the population reported a sitting time higher than 8h30 minutes per day, which reflects a high level of sedentarity. People with tertiary education level more frequently reported prolonged sitting time than people with other educational levels. There is growing interest in creating less sedentary working environments to improve health, such as by implementing workstations that promote low-intensity non-exercise physical activity to raise daily energy expenditure (59, 60). Yet the quality of currently available evidence that selected workplace interventions can reduce sitting time at work, for instance, appears to be low (61).

5. CONCLUSIONS

The first National Nutrition Survey, menuCH, provides important measured data on anthropometric parameters, as well as questionnaire-based data on diet-related behaviors, which has been tested in a pilot phase. The included participants represent a population of 4’627’878 people covering the 11 most populated Swiss cantons.

Overall, menuCH provides important new information on overweight, obesity and waist circumference based on measured data, across the three main linguistic regions of Switzerland. Novel data on cooking and eating habits provide interesting insights into regional differences that will be important to inform future nutrition policies in Switzerland.

Acknowledgement

We acknowledge our partner institution Berner Fachhochschule (BFH), Fachbereich Gesundheit. Our special thanks goes to all survey collaborators at IUMSP/CHUV, ISPM Bern and BFH as well as the numerous participants, who made data collection, data cleaning and analysis possible.

Development and implementation of the National Nutrition Survey (menuCH) was funded by the Federal Food Safety and Veterinary Office and the Federal Office of Public Health (contract 12.006650/414.000/-10. And 13.001456/414.000/-103 and 13.005454/414.0000/-103 and 13.007870/414.000/-103).

6. REFERENCES

1. Keller U, Battaglia Richi E, Beer M, Darioli R, Meyer K, Renggli A, et al. Sechster Schweizerischer Ernährungsbericht (6th Swiss Nutrition Report). Bern: Bundesamt für Gesundheit; 2012.

2. Federal Office of Public Health, FOPH, editors. Swiss Nutrition Policy 2013–2016 based on the main findings of the 6th Swiss Nutrition Report. Bern: FOPH; 2012.

3. Federal Statistical Office. Swiss Health Survey - Schweizerische Gesundheitsbefragung - Enquête Suisse sur la Santé [Available from: http://www.bfs.admin.ch/bfs/portal/de/index/infothek/erhebungen__quellen/blank/blank/ess/04.html.

4. Bundesamt für Gesundheit (BAG) und Schweizerische Konferenz der kantonalen Gesundheitsdirektorinnen und -direktoren (GDK), editor. Nationale Strategie Prävention nichtübertragbarer Krankheiten (NCD-Strategie) 2017–2024,: BAG, GDK; 2016.

5. Bundesamt für Gesundheit (BAG). Kurzbeschrieb des Monitoring-Systems Ernährung und Bewegung (MOSEB) und Indikatorensammlung [Available from: http://www.bag.admin.ch/themen/ernaehrung_bewegung/05190/index.html?lang=de ; http://www.bag.admin.ch/themen/ernaehrung_bewegung/05190/07835/index.html?lang=de.

6. Gibson Rosalind S. Measuring food consumption of individuals. In: Gibson Rosalind S, editor. Principles of Nutritional Assessment. New York: Oxford University Press; 2005. p. 41-64.

7. European Food Safety Authority. General principles for the collection of national food consumption data in the view of a pan-European dietary survey. EFSA Journal. 2009;7(12):1435 [51pp.].

8. Bundesamt für Statistik (BFS). Abteilung Gesundheit und Soziales. Die Schweizerische Gesundheitsbefragung 2012 in Kürze. Konzept, Methode, Durchführung. Neuchâtel: BFS; 2013.

9. Pasquier Jérôme. Pondération de l'enquête MenuCH. Lausanne: Institut de Medicine Sociale et Préventive (IUMSP); 2016.

10. M.I.S. Trend S.A. für das Bundesamt für Gesundheit. Nationale Ernährungserhebung. Test Fragebogendauer,. Lausanne/Bern 17.11.2011.

11. M.I.S. Trend S.A. für das Bundesamt für Gesundheit. Nationale Ernährungserhebung. Kognitiver Re-Test,. Lausanne/Bern 28.10.2011.

12. Bundesamt für Statistik (BFS). Abteilung Gesundheit und Soziales. Schweizerische Gesundheitsbefragung 2012. Telefonischer und schriftlicher Fragebogen. Neuchatel: BFS Sektion Gesundheit; 2014.

13. Craig CL, Marshall AL, Sjostrom M, Bauman AE, Booth ML, Ainsworth BE, et al. International physical activity questionnaire: 12-country reliability and validity. Med Sci Sports Exerc. 2003;35(8):1381-95.

14. Firmann M, Mayor V, Vidal PM, Bochud M, Pecoud A, Hayoz D, et al. The CoLaus study: a population-based study to investigate the epidemiology and genetic determinants of cardiovascular risk factors and metabolic syndrome. BMC Cardiovasc Disord. 2008;8:6.

15. Marques-Vidal P, Waeber G, Vollenweider P, Bochud M, Stringhini S, Guessous I. Sociodemographic and Behavioural Determinants of a Healthy Diet in Switzerland. Ann Nutr Metab. 2015;67(2):87-95.

16. Abreu D, Cardoso I, Gaspoz JM, Guessous I, Marques-Vidal P. Trends in dietary intake in Switzerland, 1999 to 2009. Public Health Nutr. 2014;17(3):479-85.

17. Marques-Vidal P, Rousi E, Paccaud F, Gaspoz JM, Theler JM, Bochud M, et al. Dietary Intake according to Gender and Education: A Twenty-Year Trend in a Swiss Adult Population. Nutrients. 2015;7(11):9558-72.

18. World Health Organization (WHO). MONICA Manual Part III: Population Survey, Section 1: Population Survey Data Component, Chapter 4.6 Height, weight, waist and hip measurement, : WHO; 1998 [Available from: http://www.thl.fi/publications/monica/manual/part3/iii-1.htm#s4-6.

19. Whigham LD, Schoeller DA, Johnson LK, Atkinson RL. Effect of clothing weight on body weight. Int J Obes (Lond). 2013;37(1):160-1.

20. World Health Organization (WHO). Obesity: Preventing and managing the global epidemic. Report of a WHO Consultation on obesity. Geneva: WHO; 2000.

21. World Health Organization (WHO). Waist circumference and waist-to-hip ratio. Report of a WHO Expert Consultation, Geneva 8-11 2008,. Geneva: WHO; 2011.

22. Thompson Frances E, Subar Amy F. Dietary Assessment Methodology. In: Coulston Ann M, Boushey Carol J, Ferruzzi Mario G, editors. Nutrition in the prevention and treatment of disease. 3rd ed. USA: Elsevier Academic Press; 2013. p. 5-46.

23. Slimani N, Casagrande C, Nicolas G, Freisling H, Huybrechts I, Ocke MC, et al. The standardized computerized 24-h dietary recall method EPIC-Soft adapted for pan-European dietary monitoring. Eur J Clin Nutr. 2011;65 Suppl 1:S5-15.

24. Slimani N, Deharveng G, Charrondière RU, van Kappel AL, Ocké MC, Welch A, et al. Structure of the standardized computerized 24-h diet recall interview used as reference method in the 22 centers participating in the EPIC project. Computer Methods and Programs in Biomedicine. 1999;58(3):251-66.

25. Camenzind-Frey E, Zuberbühler C. menuCH - SCHWEIZERISCHES FOTOBUCH / LIVRE PHOTO SUISSE / MANUALE FOTOGRAFICO SVIZZERO. Bern: Bundesamt für Gesundheit (BAG) & Bundesamt für Lebensmittelsicherheit und Veterinärwesen (BLV); 2014.

26. IPAQ. Guidelines for Data Processing and Analysis of the International Physical Activity Questionnaire (IPAQ) - Short and Long Forms. Nov 2005. p. 1-15. Available: http://www.ipaq.ki.se/scoring.pdf.

27. Bland JM, Altman DG. Measuring agreement in method comparison studies. 1999;8(2):135-60.

28. Floris J, Koepke N, Bender N, Rühli F, Staub K. Der Body Mass Index der Schweizer Stellungspflichtigen 2015 (Teil A). Fünfter Wissenschaftsaustausch MOSEB; Bern: Bundesamt für Gesundheit (BAG); 2016.

29. Wolff H, Delhumeau C, Beer-Borst S, Golay A, Costanza MC, Morabia A. Converging prevalences of obesity across educational groups in Switzerland. Obesity (Silver Spring). 2006;14(11):2080-8.

30. Budesamt für Statistik (BFS), Bundesamt für Gesundheit (BAG). Schweizerische Gesundheitsbefragung 2012: Übergewicht und Adipositas. Neuchâtel: BFS; 2014.

31. Rouiller N, Marques-Vidal P. Prevalence and determinants of weight misperception in an urban Swiss population. Swiss Med Wkly. 2016;146:w14364.

32. Robinson E, Oldham M. Weight status misperceptions among UK adults: the use of self-reported vs. measured BMI. BMC Obesity. 2016;3(1):21.

33. Hess JM, Jonnalagadda SS, Slavin JL. What Is a Snack, Why Do We Snack, and How Can We Choose Better Snacks? A Review of the Definitions of Snacking, Motivations to Snack, Contributions to Dietary Intake, and Recommendations for Improvement. Adv Nutr. 2016;7(3):466-75.

34. Bachmann M, Burnier M, Daeniker Roth C, Exl-Preysch B-M, Imfeld T, Lüthy J, et al. Salzkonsum und Bluthochdruck: Expertenbericht der Eidgenössischen Ernährungskommission (Salt consumption and blood pressure: expert report of the Federal Commission for Nutrition). Bern, Switzerland; 2004.

35. World Health Organization, editor. Guideline: Sodium intake for adults and children. Geneva: WHO; 2012.

36. Beer-Borst S, Costanza MC, Pechère-Bertschi A, Morabia A. Twelve-year trends and correlates of dietary salt intakes for the general adult population of Geneva, Switzerland. European Journal Of Clinical Nutrition. 2009;63(2):155–64.

37. Chappuis A, Bochud M, Glatz N, Vuistiner P, Paccaud F, Burnier M. Swiss survey on salt intake: main results. Lausanne; 2011.

38. Haldimann M, Bochud M, Burnier M, Paccaud F, Dudler V. Prevalence of iodine inadequacy in Switzerland assessed by the estimated average requirement cut-point method in relation to the impact of iodized salt. Public Health Nutr. 2015;18(8):1333-42.

39. Ogna A, Forni Ogna V, Bochud M, Paccaud F, Gabutti L, Burnier M, et al. Prevalence of obesity and overweight and associated nutritional factors in a population-based Swiss sample: an opportunity to analyze the impact of three different European cultural roots. Eur J Nutr. 2014;53(5):1281-90.

40. Camhi SM, Bray GA, Bouchard C, Greenway FL, Johnson WD, Newton RL, et al. The relationship of waist circumference and BMI to visceral, subcutaneous, and total body fat: sex and race differences. Obesity (Silver Spring). 2011;19(2):402-8.

41. Sebo P, Beer-Borst S, Haller DM, Bovier PA. Reliability of doctors' anthropometric measurements to detect obesity. Prev Med. 2008;47(4):389-93.

42. Chen Z, Klimentidis YC, Bea JW, Ernst KC, Hu C, Jackson R, et al. Body Mass Index, Waist Circumference, and Mortality in a Large Multiethnic Postmenopausal Cohort-Results from the Women's Health Initiative. J Am Geriatr Soc. 2017.

43. Pischon T, Boeing H, Hoffmann K, Bergmann M, Schulze MB, Overvad K, et al. General and abdominal adiposity and risk of death in Europe. N Engl J Med. 2008;359(20):2105-20.

44. Hartz A, He T, Rimm A. Comparison of adiposity measures as risk factors in postmenopausal women. J Clin Endocrinol Metab. 2012;97(1):227-33.

45. Troxler DS, Michaud PA, Graz B, Rodondi PY. Exploratory survey about dietary supplement use: a hazardous and erratic way to improve one's health? Swiss Med Wkly. 2013;143:w13807.

46. Marques-Vidal P, Pecoud A, Hayoz D, Paccaud F, Mooser V, Waeber G, et al. Prevalence and characteristics of vitamin or dietary supplement users in Lausanne, Switzerland: the CoLaus study. Eur J Clin Nutr. 2009;63(2):273-81.

47. Publitest - mafo concept GmbH für die Forschungsgruppe Good Practice Gemeinschaftsgastronomie. Projekt "Gesundheitsfördernde Gemeinschaftsgastronomie"

Telefoninterviews in der Bevölkerung sowie mit Verpflegungsendanbietern und Produzenten/Lieferanten. Zürich: Publitest; 14. Juli 2011.

48. Imamura F, O'Connor L, Ye Z, Mursu J, Hayashino Y, Bhupathiraju SN, et al. Consumption of sugar sweetened beverages, artificially sweetened beverages, and fruit juice and incidence of type 2 diabetes: systematic review, meta-analysis, and estimation of population attributable fraction. BMJ. 2015;351:h3576.

49. de Ruyter JC, Olthof MR, Seidell JC, Katan MB. A trial of sugar-free or sugar-sweetened beverages and body weight in children. N Engl J Med. 2012;367(15):1397-406.

50. Singh GM, Micha R, Khatibzadeh S, Lim S, Ezzati M, Mozaffarian D, et al. Estimated Global, Regional, and National Disease Burdens Related to Sugar-Sweetened Beverage Consumption in 2010. Circulation. 2015;132(8):639-66.

51. World Health Organization (WHO). Global recommendations on physical activity for health. Geneva: WHO; 2010.

52. Hagstromer M, Oja P, Sjostrom M. The International Physical Activity Questionnaire (IPAQ): a study of concurrent and construct validity. Public Health Nutr. 2006;9(6):755-62.

53. Craft LL, Zderic TW, Gapstur SM, Vaniterson EH, Thomas DM, Siddique J, et al. Evidence that women meeting physical activity guidelines do not sit less: an observational inclinometry study. Int J Behav Nutr Phys Act. 2012;9:122.

54. Katzmarzyk PT, Church TS, Craig CL, Bouchard C. Sitting time and mortality from all causes, cardiovascular disease, and cancer. Med Sci Sports Exerc. 2009;41(5):998-1005.

55. Hamilton MT, Hamilton DG, Zderic TW. Role of low energy expenditure and sitting in obesity, metabolic syndrome, type 2 diabetes, and cardiovascular disease. Diabetes. 2007;56(11):2655-67.

56. Pandey A, Salahuddin U, Garg S, Ayers C, Kulinski J, Anand V, et al. Continuous Dose-Response Association Between Sedentary Time and Risk for Cardiovascular Disease: A Meta-analysis. JAMA Cardiol. 2016;1(5):575-83.

57. Ekelund U, Steene-Johannessen J, Brown WJ, Fagerland MW, Owen N, Powell KE, et al. Does physical activity attenuate, or even eliminate, the detrimental association of sitting time with mortality? A harmonised meta-analysis of data from more than 1 million men and women. Lancet. 2016;388(10051):1302-10.

58. Benatti FB, Ried-Larsen M. The Effects of Breaking up Prolonged Sitting Time: A Review of Experimental Studies. Med Sci Sports Exerc. 2015;47(10):2053-61.

59. Tudor-Locke C, Schuna JM, Jr., Frensham LJ, Proenca M. Changing the way we work: elevating energy expenditure with workstation alternatives. Int J Obes (Lond). 2014;38(6):755-65.

60. Buckley JP, Hedge A, Yates T, Copeland RJ, Loosemore M, Hamer M, et al. The sedentary office: an expert statement on the growing case for change towards better health and productivity. Br J Sports Med. 2015;49(21):1357-62.

61. Shrestha N, Kukkonen-Harjula KT, Verbeek JH, Ijaz S, Hermans V, Bhaumik S. Workplace interventions for reducing sitting at work. Cochrane Database Syst Rev. 2016;3:CD010912.
