# Supplementary material for: Dietary fibre intake in the adult Swiss population: a comprehensive analysis of timing and sources
Source: J Nutr Sci. 2025 Mar 24;14:e27. doi: 10.1017/jns.2025.6 (PMC11950702; doi:10.1017/jns.2025.6)
Supplement: von Blumenthal et al. supplementary material 1 — von Blumenthal et al. supplementary material [file S2048679025000060sup001.zip › AEE-2400061-Thesis Symosis (1).pdf]

Australian Journal of Environmental Education (2024), 1–4
doi:10.1017/aee.2024.61

CAMBRIDGE UNIVERSITY PRESS

THESIS SYNOPSIS

Culturally Oriented Environmental Identity Transitions: Migrant Indian Teachers in Aotearoa New Zealand Early Childhood Education

Devika Rathore

New Zealand Tertiary College, Tāmaki Makaurau Auckland, Aotearoa New Zealand
Email: devikarathore@gmail.com

(Received 05 September 2024; revised 12 September 2024; accepted 19 September 2024)

Keywords: Cultural identity; early childhood education; environmental education; environmental identity; Indian teachers; sustainability education

Thesis synopsis

There has been a growing awareness of the detrimental impact of human activity on the environment in the Anthropocene (Crutzen, 2002; Eames & Cutter-Mackenzie, 2017). This impact requires a reconsideration of human-environment relationships including an examination of our environmental identities. An environmental identity can be described as the way individuals view themselves or extend their sense of self in relation to the natural environment (Thomashow, 1995) mediated by personal and social experiences. To address this critical need to restore the human-environment relationship through the exploration of an environmental identity, education has emerged as a vital strategy, and culture has emerged as a significant resource.

In Aotearoa New Zealand, the natural environment is considered an integral part of the national identity, and early childhood has been recognised as an important stage when environmental and sustainability education should begin. The early childhood education (ECE) bicultural curriculum framework *Te Whāriki* (Ministry of Education [MoE], 2017), which is grounded in Indigenous Māori worldviews, acknowledges strong spiritual connections to land and place. The idea of respect for the natural world and kaitiakitanga (environmental stewardship as referred to in *Te Whāriki*) are included in the curriculum document.

Within the context of this superdiverse nation (Vertovec, 2019), and an increasingly multicultural ECE context (Chan & Ritchie, 2023), a significant number of migrant teachers may transfer their cultural and environmental knowledge, practice and identity as they transition into the Aotearoa New Zealand context. Migration to this country by people from India has increased significantly in number over the past few years (Stats NZ, 2018), and this growth is being reflected in the increasing numbers of migrant Indian teachers in ECE.

Within this context, as a migrant teacher myself, and a cultural insider with lived-familiarity of the teachers' experiences, I was keen to examine if the environment might hold different meanings and places in their home (Indian) and host (Aotearoa New Zealand) cultural systems. An exploration of my own culturally oriented environmental identity, concern for nurturing my young son's environmental identity and the want of environmental awareness among my own student–teacher cohort, eventually led me to this journey and shaped my research. Therefore, my

A thesis submitted for the award of Doctor of Philosophy in the School of Education at the University of Waikato.

© The Author(s), 2024. Published by Cambridge University Press on behalf of Australian Association for Environmental Education. This is an Open Access article, distributed under the terms of the Creative Commons Attribution licence (http://creativecommons.org/licenses/by/4.0/), which permits unrestricted re-use, distribution and reproduction, provided the original article is properly cited.

research explored migrant Indian teachers' understandings of their culturally oriented environmental identity development as they negotiated their cultural and environmental transitions into the multicultural and environmentally inclusive Aotearoa New Zealand ECE context. The aim of the research was to gain insights into the teachers' perceptions of the influence of cultural identity on their environmental identities within the ECE context.

A sociocultural theoretical perspective informed this study, drawing primarily on Sauvé's (2009) model of personal and social development of the self in relation to other humans and the environment. This was considered with reference to, and overlaps with, certain Indian environmental philosophical perspectives and Indigenous Māori environmental perspectives. Primary data for this interpretivist study were gathered through in-depth interviews with nine Indian ECE teacher participants. Interviews with their ECE setting managers/head teachers were conducted to support and supplement the teachers' practices and experiences. The audio-recorded interview data were transcribed and examined using thematic analysis (Braun & Clarke, 2006). Observations of teacher participants' workplaces were used to provide a contextual profile for each of the nine Indian teachers. I also obtained a small number of assessment documents in the form of Learning Stories (Carr, 2001), which the teachers had prepared, to analyse them for cultural and environmental themes and experiences.

Indian teacher participants' perceptions of their own culturally oriented environmental identities highlighted the significance of these teachers' own early childhood environmental experiences and home cultural context influences on their environmental identities. Teacher participants' perceptions shed light on their cultural and environmental identity transitions and acculturation process. These teachers brought their cultural and environmental identities from their home cultural context and used their cultural lens to interpret the connections between the host cultural context and environmental worldviews.

The Indian teacher participants perceived close connections between their cultural and environmental identities. At the same time, they recognised cross-cultural connections between their culturally oriented environmental identities and tikanga Māori (Māori ways of doing, including practices, customs and rituals) and te ao Māori (the Māori world). These perceived connections were most evident through the significance of Indian philosophies of spirituality, relationships, belonging and cultural recognition and exchange in their specific ECE contexts. These connections facilitated teachers' cultural and environmental identity transitions and had a positive impact on their cultural and environmental teaching practices, and in some cases, this was supported by the ECE setting and by the respective management/leadership.

The findings indicate a scope for increased opportunities for migrant teachers to explore, share, enhance and add to their context-specific environmental values and beliefs within initial teacher education programmes and their professional practice as qualified teachers. A more in-depth orientation to environmental and sustainability education values, philosophies, practices and programmes within Aotearoa New Zealand would provide the required support during the cultural crossover and environmental identity transition.

The teachers perceived cross-cultural and spiritual connections that could provide ideal spaces to encourage the inclusion and facilitation of migrant teachers' cultural and environmental identities. These connections could also become sites for cultural exchange where teachers from diverse cultures can share their unique environmental and sustainability perspectives, knowledge and experiences to enrich the ECE context. Migrant teachers bring their own funds of identity (Esteban-Guitart & Moll, 2014), cultural and environmental, which could facilitate sharing of ideas and approaches from various worldviews. One significant finding that emerged from the study is the spiritual connections that Indian teacher participants made between their home and host cultural identities as well as their cultural and environmental identities, which is an interesting and valuable focus that I hope to explore in the near future.

Cultural and environmental transitions for this steadily growing group of migrant teachers require further investigation to facilitate their cultural and environmental participation and

acculturation into the Aotearoa New Zealand ECE context. In these ways, the existing cultural, environmental and sustainability connections within ECE could be strengthened to foster children's culturally oriented environmental identities.

Citation and digital source for thesis

Rathore, D. (2024). *Culturally-Oriented Environmental Identity Transitions: Migrant Indian Teachers in Aotearoa New Zealand Early Childhood Education.* [Doctoral thesis, The University of Waikato]. The University of Waikato Research Commons. https://hdl.handle.net/10289/16502

Supervisors:

Associate Professor Chris Eames
The University of Waikato

Dr Janette Kelly-Ware
The University of Waikato

Acknowledgements. My sincere gratitude to my supervisors, the participants, my family and every being (human/more-than-human) who was part of my doctoral journey.

Financial support. None.

Ethical standards. Research ethics approval was granted by Te Kura Toi Tangata Division of Education Human Research Ethics Committee, the University of Waikato.

Publications arising from the thesis

Rathore, D., Eames, C., & Kelly-Ware, J. (2020). Indian teachers and environmental identity in Aotearoa New Zealand early childhood education. *Teachers and Curriculum, 20*(2020), 13–21. https://doi.org/10.15663/tandc.v20i1.350

Rathore, D. (2022). Overcoming data collection challenges and establishing trustworthiness: The need for flexibility and responsiveness in research. *Waikato Journal of Education 27*(2), 47–51. https://doi.org/10.15663/wje.v27i2.932

Rathore, D. (2022, October 12). *Identity and connection to place: Indian migrant teachers in Aotearoa New Zealand early childhood education*. New Zealand Association for Environmental Education Conference. https://www.nzaee.org.nz/conference-pages/speakers-presentations

Rathore, D. (2022, June 7). Identity, culture, and environment: Indian early childhood teachers in Aotearoa New Zealand. *Ipu Kererū Blog of the New Zealand Association for Research in Education*. https://nzareblog.wordpress.com/2022/06/07/indian-ece-teachers/

Rathore, D. (2023, May 23). Kaitiakitanga and the multicultural strands of Te Whāriki. *Ipu Kererū Blog of the New Zealand Association for Research in Education*. https://nzareblog.wordpress.com/2023/05/23/kaitiakitanga-and-the-multicultural-strands-of-te-whariki/

References

Braun, V., & Clarke, V. (2006). Using thematic analysis in psychology. *Qualitative Research in Psychology*, *3*(2), 77–101. DOI: 10.1191/1478088706qp063oa.

Carr, M. (2001). *Assessment in early childhood settings: Learning stories.* Paul Chapman.

Chan, A., & Ritchie, J. (2023). Exploring a Tiriti-based superdiversity paradigm within early childhood care and education in Aotearoa New Zealand. *Contemporary Issues in Early Childhood*, *24*(1), 5–19. DOI: 10.1177/1463949120971376.

Crutzen, P.J. (2002). The "anthropocene". *Journal de Physique IV (Proceedings)*, *12*(10), 1–5. DOI: 10.1051/jp4:20020447.

Eames, C.W., & Cutter-Mackenzie, A. (2017). Backyard perspectives: Considering a biogeographical, sociocultural and socioecological framing of environmental education in Australasia and Asia. *The Japanese Society of Environmental Education*, *26*(4), 65–70. DOI: 10.5647/jsoee.26.4_65.

Esteban-Guitart, M., & Moll, L.C. (2014). Funds of identity: A new concept based on the funds of knowledge approach. *Culture & Psychology*, *20*(1), 31–48. DOI: 10.1177/1354067X13515934.

Ministry of Education. (2017). Te Whāriki He whāriki mātauranga mō ngā mokopuna o Aotearoa Early childhood curriculum. https://tewhariki.tki.org.nz/en/key-documents/te-whariki-2017/.

Sauvé, L. (2009). Being here together. In M. McKenzie, P. Hart, H. Bai & B. Jickling (Eds.), *Fields of green: Restorying culture, environment, and education* (pp. 325–335). Hampton Press Inc.

Stats, N.Z. (2018). 2018 Census Indian ethnic group. https://www.stats.govt.nz/tools/2018-census-ethnic-group-summaries//Indian.

Thomashow, M. (1995). *Ecological identity*. Massachusetts Institute of Technology.

Vertovec, S. (2019). Talking around super-diversity. *Ethnic and Racial Studies*, *42*(1), 125–139. DOI: 10.1080/01419870.2017.1406128.

Author Biography

Devika Rathore is a lecturer in early childhood education at the New Zealand Tertiary College, Aotearoa New Zealand. She was engaged in tertiary education and research while pursuing her PhD from the University of Waikato. Her research explores the intersection of environment, culture and education. She is particularly interested in learning more about how people's connection to place, nature and the more-than-human shapes their environmental identities. Her research interests include environmental/nature-based/sustainability education, early childhood education, identity, cross-cultural studies and visual dissemination of research.

Cite this article: Rathore, D. (2024). Culturally Oriented Environmental Identity Transitions: Migrant Indian Teachers in Aotearoa New Zealand Early Childhood Education. *Australian Journal of Environmental Education*. https://doi.org/10.1017/aee.2024.61
