# Supplementary material for: Dietary fibre intake in the adult Swiss population: a comprehensive analysis of timing and sources
Source: J Nutr Sci. 2025 Mar 24;14:e27. doi: 10.1017/jns.2025.6 (PMC11950702; doi:10.1017/jns.2025.6)
Supplement: von Blumenthal et al. supplementary material 1 — von Blumenthal et al. supplementary material [file S2048679025000060sup001.zip › JNS-2300002.pdf]

QUERIES

AQ1: The distinction between surnames can be ambiguous, therefore to ensure accurate tagging for indexing purposes online (e.g. for PubMed entries), please check that the highlighted surnames have been correctly identified, that all names are in the correct order and spelt correctly.

AQ2: Please check that affiliations of all the authors and the corresponding author details are correctly set.

AQ3: Please check the sentence “Low cardiac output, which data from . . . ” for clarity.

AQ4: Please check if edit made to the sentence “Cognition was assessed via five episodic . . . ” is fine.

Journal of Nutritional Science

cambridge.org/jns

The role of fruit and vegetables in sustaining healthy cognitive function: evidence and issues

Crystal F. Haskell-Ramsay and Sarah Docherty

Department of Psychology, Northumbria University, Newcastle upon Tyne, UK

Review

Cite this article: Haskell-Ramsay CF and Docherty S (2023). The role of fruit and vegetables in sustaining healthy cognitive function: evidence and issues. *Journal of Nutritional Science*, page 1 of 9. doi: 10.1017/S0029665123002999

Received: 3 November 2022
Revised: 31 March 2023
Accepted: 6 April 2023

Keywords
Fruit and vegetables; Diet; Cognitive decline; Ageing

Abbreviations:
GWAS, genome-wide association study; IV, instrumental variable; IVW, inverse variance weighted; MR, Mendelian randomization; UKB, UK Biobank

Corresponding author:
Crystal F. Haskell-Ramsay, Email: crystal.haskell-ramsay@northumbria.ac.uk

Abstract

Modifiable lifestyle factors, such as improved nutrition, are crucial in maintaining cognitive health in older age. Fruit and vegetables represent healthy and sustainable sources of nutrients with the potential to prevent age-related cognitive decline. The aim of this review is to synthesise the available evidence, from epidemiological and randomised controlled trials (RCT), regarding the role of fruit and vegetables in sustaining healthy cognitive function. Epidemiological studies of combined fruit and vegetable intake suggest that increased consumption may sustain cognition in later life. The evidence appears to be stronger for an association between vegetables and cognition, particularly for green leafy and cruciferous vegetables. Specific benefits shown for berries, citrus fruits, avocado and nuts suggest fruit is worthy of further investigation in relation to cognition. Data from RCT indicate benefits to differing aspects of cognition following citrus and berry fruits, cocoa and peanuts, but the data are limited and there are a lack of studies exploring effects of vegetables. There is growing evidence for an association between fruit and vegetable intake and cognitive function, but this is not always consistent and the data from RCT are limited. Issues in previous research are highlighted, such as strict exclusion criteria, absence of baseline nutritional status data and lack of consideration of individual differences, which may explain the weaker findings from RCT. Inclusion of those most at risk for cognitive decline is recommended in future nutrition and cognition research.

The world's population is ageing. By 2030, one in six people will be aged 60 years or over[1]. However, average health span is not increasing at the same rate as lifespan, as indicated by the increasing prevalence of non-communicable diseases such as CVD and diabetes. Of relevance to this review, dementia has recently emerged as the leading cause of death in the UK. In 2019, 1 in every 14 of the UK population aged 65 years and over was diagnosed with dementia[2], and globally this condition affects more than twice as many women as men[3]. Despite the high prevalence and significant impact of dementia, such as Alzheimer's disease, there are currently no effective pharmaceutical treatments to slow down or reverse disease progression. It is, therefore, imperative that we develop ways to prevent, or at least delay, dementia and maintain cognitive health into older age.

Nutrition represents a crucial modifiable lifestyle factor in the quest to sustain health, including healthy cognitive function. The importance of nutrition is evident from the inverse relationships between non-communicable diseases and adherence to healthy dietary patterns such as the Mediterranean diet, Dietary Approaches to Stop Hypertension (DASH) diet, the Mediterranean-DASH Intervention for Neurodegenerative Delay (MIND), the Nordic diet and the Traditional Asian diet[4]. In fact, dietary risks were observed to be the leading cause of disease burden in the USA between 1990 and 2010[5], with low intake of fruit, vegetables and nuts listed in the top five leading dietary risk factors for death and disability-adjusted life years globally[6]. CVD was cited as the leading cause of diet-related death and disability-adjusted life years, highlighting the importance of dietary factors to cardiovascular health. Cardiovascular risk factors are also linked to cognition. For instance, high cholesterol at midlife is associated with increased risk of vascular dementia[7], as well as accelerated cognitive decline, including a specific association for LDL cholesterol[8]. Hypertension has been linked to dementia[9], including Alzheimer's disease[10], as well as steeper cognitive decline[11] and poor cognitive function[12]. There is also evidence for race[11] and sex differences[13] in these relationships. Prior to hypertension, arterial stiffness is associated with worse cognitive outcomes in older adults and in midlife[14]. Low cardiac output, which data from the Framingham Heart Study Offspring Cohort indicate one-third of the general adult population meets clinical criteria for[15], is associated with worse cognitive performance[16]. This is potentially mediated by the corresponding cerebral blood flow reductions observed in cardiac dysfunction, which can lead to synaptic dysfunction[17] and blood–brain barrier permeability[18]. Increased risk of dementia[19] and accelerated cognitive decline[20] are also observed in type 2 diabetes, which may be due to increased blood–brain barrier permeability[21]. Blood–brain barrier disruption has been linked to inflammation[22,23], which can increase the risk of atherosclerosis and insulin resistance[24]. Potential mechanisms of increased inflammation in older age and in chronic

© The Author(s), 2023. Published by Cambridge University Press on behalf of The Nutrition Society. This is an Open Access article, distributed under the terms of the Creative Commons Attribution licence (http://creativecommons.org/licenses/by/4.0/), which permits unrestricted re-use, distribution, and reproduction in any medium, provided the original work is properly cited.

The Nutrition Society

CAMBRIDGE UNIVERSITY PRESS

conditions include changes to gut microbiota and gut permeability [25], which are also linked to brain function[26] and to mental health conditions including depression[27]. The risk factors outlined above all have the potential to be impacted by diet and could therefore represent mechanisms for dietary effects on cognition. Obesity is also associated with a pro-inflammatory state[25], highlighting the role of diet, which is supported by studies showing beneficial effects of anti-inflammatory diets[28,29].

In meta-analyses, adherence to the Mediterranean diet was associated with better global cognition both cross-sectionally and longitudinally[30], while intervention with a Mediterranean diet was shown to improve cognition when compared with a low-fat control diet[31]. One aspect that is common across healthy diet patterns such as the Mediterranean, DASH and MIND diets is the recommendation to increase intake of fruit and vegetables. The EAT lancet commission report[32], which highlights food as the greatest prospect to optimise health and environmental sustainability, also recommends increased fruit and vegetable intake to improve human and planetary health. A recent review of the EAT-Lancet reference diet and cognition across the life course concluded that the current evidence base is weak, but high fruit and vegetable intake seems to benefit cognition, particularly in older adults[33].

The aim of this review is to synthesise the evidence for fruit and vegetable intake to sustain cognition in cognitively healthy older adults and to highlight challenges in this field of research. One issue that is immediately apparent is the use of dementia screening tools, such as the mini-mental state examination (MMSE), to assess cognition in cognitively healthy older adults. Due to the lack of sensitivity of these screening tools, this review will focus on studies that include measures of cognitive function. One of the most studied aspects of age-related cognitive decline is episodic memory, which reflects the ability to store and subsequently retrieve, and to recognise, previously presented verbal, visual or spatial information. Another aspect of cognition that deteriorates with age is the executive function. This involves complex or higher order thinking such as planning, problem-solving and reasoning and is underpinned by working memory as well as information processing. Sustained and selective attention are also important cognitive domains, which respectively reflect the ability to maintain attention on a stimulus or a task for a long period and the ability to respond to relevant stimuli (targets) while ignoring irrelevant stimuli (distractors). One of the difficulties here is that cognitive tasks are often mixed, so there is overlap in terms of the domains involved. For example, the Stroop task involves aspects of executive function while also assessing selective attention (see Table 1 for summary and evaluation of key cognitive domains).

In the following sections, data on the relationship between fruit and vegetable intake and cognitive performance are reviewed, followed by a review of randomised controlled trials (RCT) exploring the impact of daily fruit and vegetable intake on cognitive function in healthy older adults.

Epidemiological evidence

Epidemiological studies in this area that have focused on fruit and vegetables as a combined category have shown some positive findings. In the Whitehall II study, consuming <2 portions of fruit and vegetables per day was associated with increased risk of poor executive function and, to a lesser extent, poorer episodic memory [34]. This association was apparent for fruit and vegetable intake at midlife but was strongest when measured cross-sectionally at 15-year follow-up. The Supplementation with Antioxidant Vitamins and Minerals 2 (SU.VI.MAX 2) study also showed that higher fruit and vegetable intake was associated with better verbal memory at 13-year follow-up, with specific effects for fruit and for vitamin C-rich fruit and vegetables[35]. However, in this study the association with executive functioning was negative. One possible explanation for the discrepancy in findings relating to executive

Table 1. Summary and evaluation of key cognitive domains

Cognitive domain	Task requirements	Task examples	Evaluation
Episodic memory	The ability to store and subsequently retrieve, and to recognise, previously presented verbal, visual or spatial information	Immediate and delayed word recall, word recognition, paired associates learning test, letter memory, verbal memory tasks, object recognition, Corsi blocks task	Overlap in domains measured via tasks, scores on different tasks often do not correlate despite measuring the same domain
Executive functions	Complex or higher order thinking such as planning, problem-solving and reasoning	Task-switching test, trail making test, Stroop task	Overlap in domains measured via tasks, studies often do not measure all sub-domains of executive function as this is not done in one task, tasks are very susceptible to age, education and socio-economic status
Working memory	Retaining information temporarily to use for more complex cognitive functions	Numeric working memory task, digit span, spatial working memory task, Corsi blocks, serial subtractions, Sternberg memory scanning, trail-making test	
Information processing	Decoding incoming information and formulating a response	Rapid visual processing task, Stroop task, letter digit substitution test	
Sustained attention	Direct and focus cognitive activity on specific stimuli	Attention network task, rapid visual processing task, sustained attention task	Overlap in domains measured via tasks, only small to moderate correlations found between psychological measures of sustained attention and behavioural ratings
Selective attention	Select and focus on a particular stimulus for further processing while simultaneously suppressing irrelevant information	Stroop task. Modified attention network task, selective reminding test	Overlap in domains measured via tasks, some tasks have low construct validity and external validity

function is the classification of verbal fluency as memory tasks in the SU.VI.MAX 2 study[35], whereas this was categorised as executive function in the Whitehall II study[36]. It is also important to note that in both studies no measure of cognition was taken at baseline, so assessment of cognitive decline was not possible. Support for a relationship between fruit and vegetable intake in late midlife and cognition in later life also comes from the Health Professionals Follow-up Study, which showed that higher consumption of both fruit and vegetables was associated with lower odds of poor subjective cognitive function at 18–22-year follow-up[37]. While no objective measure was included in this study, the experience of cognitive function subjectively may be particularly important in terms of quality of life. Performance on cognitive tests assessing memory and executive function improved with increasing intake of fruit and vegetables in the Hordaland Health Study, with a marked dose–dependent relationship up to around 500 g/day. When analysed separately, fruit consumption was positively associated with performance on three out of five cognitive tests, whereas vegetable consumption was positively associated with performance on four tests[38]. Interestingly, data from the Boston Puerto Rican Health Study indicated that greater variety of fruit and vegetable intake but not quantity was associated with better cognition, and this association was strongest for vegetables[39].

Studies that have examined the role of vegetables separately from fruit have shown higher intake to be associated with smaller declines in processing speed and global cognitive function. This is despite vegetable intake being associated with lower information processing speed and worse cognitive flexibility at baseline, which may be driven by negative effects of allium (garlic, onion and leek) consumption[40]. Total vegetable intake was also inversely associated with cognitive decline in the Nurses' Health Study, despite no such association for fruit and vegetables combined. The highest intake of vegetable was equivalent to 1.5 years less decline at the 2-year follow-up[41]. Similarly, those in the highest two quintiles of vegetable intake in the Chicago Health and Aging Project showed a 40 % reduction in decline on a global cognition measure at 6-year follow-up when compared with the lowest quartile intake[42]. Moreover, a specific association between cognitive decline and green leafy vegetables was observed[42,43]. This is supported by the Nurses' Health Study showing not only the greatest benefit for green leafy vegetables but also a specific benefit for cruciferous vegetable, particularly for episodic memory[41]. In the Doetinchem Cohort Study, higher intake of cabbage was associated with better memory and better global cognitive function at baseline and with smaller decline in information processing speed at 5-year follow-up, while root vegetables were associated with smaller declines in cognitive flexibility and global cognition [40]. Support for the roles of root and cruciferous vegetables also comes from the Hordaland Health Study showing strongest associations not only for carrots, and cruciferous vegetables, but also for citrus fruits[38].

Effects of specific fruits have been explored with the Nurses' Health Study showing that greater intake of blueberries and strawberries was associated with better episodic memory scores and slower rates of cognitive decline for global cognition, equivalent to up to 2.5 years of ageing[44]. Consumers of avocado were also shown to have better episodic memory and global cognition than non-consumers[45]. While not always categorised as fruit, a specific benefit has also been shown for tree nuts. The Doetinchem Cohort Study observed that higher nut intake was related to better global cognition, flexibility, memory and processing speed[40]. Long-term (6-year) nut intake was linked to better global cognition in women aged ≥70 years but not with cognitive decline[46] and an association to 5-year cognitive decline in the Doetinchem Cohort Study was lost following adjustment for cardiovascular risk factors[40]. This indicates a role for cardiovascular effects, which is supported by cognitive improvements shown following Mediterranean diet plus mixed tree nuts in those at high cardiovascular risk[31].

Epidemiological data suggest a relationship between fruit and vegetable intake and cognition. The evidence appears to be stronger for an association between vegetables and cognition than for fruit, which may relate to the common inclusion of fruit juice in the fruit category, despite differences in their fibre content. Since fruit and fruit juice modulate different aspects of immune function, with juice potentially linked to pro-inflammatory pathway activation[47], the inclusion of fruit juice in the fruit category may be concealing effects of whole fruit. Specific benefits shown for berries, citrus fruits, avocado and nuts suggest fruit is worthy of further investigation in relation to cognition; nuts in particular represent a nutrient-dense food with less sugar than other fruits. In terms of vegetables, a specific benefit may be seen for cruciferous or green leafy vegetables. However, this is not consistently shown with one study showing benefits for both[41], while others show benefits for one or the other; a specific benefit for cabbage[40] may indicate that green and leafy vegetables such as kale and savoy cabbage may confer the most favourable effects, potentially due to the high levels of glucosinolates, carotenoids, vitamins, minerals and flavonoids. In an analysis focused on flavonoids, the Lothian Birth Cohort 1936 study failed to show effects of fruit and/or vegetables[48]. This potentially highlights the importance of covariates in epidemiological research since relationships between vegetable intake and cognition were lost after controlling for childhood IQ, while those for total fruit intake were lost with the additional inclusion of smoking, socio-economic status, education and the apoE 14 allele.

Randomised controlled trials

RCT in this area (see Table 2) have tended to focus on fruit, specifically flavonoid-rich fruit. The effects of orange juice consumption were assessed in a crossover trial of 60–81-year-olds. Cognition was assessed via five episodic memory tasks and six **AQ4** executive function tasks, before and after a 500-ml serving of 305 mg flavanones or placebo for 8 weeks. The findings revealed improvement in global cognitive function, with no effects on episodic memory or executive function when assessed separately [49]. Several studies have also assessed the effects of flavonoid-rich berries. Effects of 12 weeks' tart cherry juice were assessed in 65–80-year-olds on measures of episodic memory, sustained attention, information processing, executive function and subjective memory[50]. Compared with placebo, 480 ml tart cherry juice per day led to fewer errors on an episodic memory task and shorter movement reaction time, as well as increasing contentment with memory. Two studies have assessed effects of blueberry in older adults[51,52]. Twenty-four g/d freeze-dried blueberry extract (equivalent to one cup of fresh blueberries) led to improved episodic memory and executive function in 60–75-year-olds, with no effects on information processing or attention tasks[51]. Different doses and forms of blueberry have also been assessed in 65–80-year-olds[52]. Effects of 500 mg and 1000 mg wild blueberry powder and 100 mg of a purified extract were assessed on measures of episodic memory, executive function and selective attention. The purified extract led to improved episodic memory

Table 2. Randomised controlled trials of fruit and vegetable juices and extracts

Reference	Sample	Study Design	Intervention	Method of cognitive assessment	Other relevant assessments	Key findings
Babateen *et al.*[36]	*n* = 50 (61 % female), overweight or obese older adults (mean age 66 years)	Single-blind, parallel groups, feasibility trial. Tested at baseline and 13 weeks	High dose (800 mg/day), moderate (400 mg/day), low (200 mg/day) or nitrate-depleted beetroot juice	Word presentation, immediate word recall, numeric working memory, choice reaction time, Stroop, digit vigilance, computerised Corsi blocks, peg and ball, delayed word recall, word recognition, trail making test A and B. Combined to create accuracy and speed of attention, working memory, episodic memory and global cognition	Cerebral blood flow via quantitative near-IR spectroscopy	No significant effects on cognition or cerebral blood flow
Brickman *et al.*[56]	*n* = 37 (73 % female), healthy but sedentary, 50–69 years old (mean age 58 years)	Double-blind, parallel groups. Tested at baseline and 12 weeks	High flavanol – 900 mg of cocoa flavanols (138 mg of epicatechin); or low flavanol control – 10 mg cocoa flavanols (<2 mg epicatechin)	Modified-Benton task	High resolution fMRI to map precise site of age-related dentate gyrus function	High flavanol-reduced reaction time compared with control and increased cerebral blood volume in dentate gyrus
Chai *et al.*[50]	*n* = 37 (54 % female), 65–80 years old (mean age 70 years)	Parallel groups. Tested at baseline and 12 weeks	Montmorency tart cherry juice (480 ml tart cherry juice, 68 ml Montmorency tart cherry juice concentrate, 412 ml water), consumed twice per day. Placebo matched for carbohydrate content, colour and tartness	Paired associates learning, rapid visual information processing, reaction time test, spatial working memory test, digit span, subjective memory		Compared with placebo, cherry juice increased contentment with memory, reduced movement time, reduced errors in episodic visual memory, improved visual sustained attention and improved working memory
Crews *et al.*[54]	*n* = 47 (55 % female), ≥60 years with no history of dementia or neurocognitive impairment (mean age 69 years)	Double-blind, parallel groups. Tested at baseline and 6 weeks	Cranberry juice, sweetened with sucralose. 16 oz consumed twice per day. Placebo matched for appearance, smell, taste and vitamin C content)	Selective reminding test, Weschler memory scale-III faces I and faces II subtests, trail making test A and B, Stroop colour and word test, Weschler adults intelligence scale iii and digit symbol-coding subtest	Follow up self-report questionnaire to measure changes over the 6 weeks in overall ability to remember, thinking abilities, moods, energy levels and overall health	No effects of cranberry juice
Flanagan *et al.*[53]	*n* = 60 (58 % female) healthy older adults (mean age 65.5 years)	Double-blind, parallel groups. Tested at baseline and 12 weeks	Freeze-dried cranberry extract, 4.5 g sachet consumed twice per day, equivalent to 100 g fresh cranberries. Placebo matched for taste, colour, fructose, total sugar and energy content	Addenbrooke's cognitive examination III – composite global cognition, executive function and verbal episodic memory. Trail making test, digit span, Rey complex figure test. Supermarket test	MRI brain scans and blood measures of polyphenol metabolites	Cranberry extract improved episodic memory, compared with placebo and increased perfusion in key cerebral regions supporting memory consolidation and retrieval
Kean *et al.*[49]	*n* = 37 (65 % female) healthy older adults (mean age 67)	Double-blind, crossover. Each treatment consumed for 8 weeks with a 4-week washout	Orange juice drink either containing high flavanones (305 mg/day) or low flavanones (37 mg/day)	Go-NoGo, CERAD (Consortium to Establish a Registry for Alzheimer's Disease) immediate and delayed recall, letter memory, verbal paired associates, serial sevens, spatial working memory, digit symbol substitution test, letter fluency. tasks grouped into two domains: episodic memory and executive function		Global cognitive function better after flavanone-rich juice, compared with low-flavanone juice. High-flavanone juice attenuated general decline in cognitive performance

Table 2. (*Continued*)

Mastroiacovo *et al.*[55]	$n = 90$ (59 % female) elderly (mean age 65 years)	Double-blind, parallel groups. Tested at baseline and 8 weeks	Dairy-based cocoa drink; high flavanol (993 mg serving), intermediate flavanol (520 mg serving) and low/control flavanol (48 mg serving)	Mini-mental state exam, trail making test A and B, verbal fluency test. Overall cognitive function also calculated	Blood pressure at each testing visit and blood sample to measure lipid profile, fasting plasma glucose and insulin	Cocoa flavanols consumed for 8 weeks improved cognitive function, blood pressure and metabolic markers. Change in insulin resistance predicted cognitive change
Miller *et al.*[51]	$n = 37$ (68 % female) adults aged 60–75 years (mean age 67 years)	Double-blind, parallel groups. Tested at baseline, 6 weeks and 12 weeks	Blueberry freeze-dried extract. 12 g consumed twice per day, equivalent to one cup of fresh blueberries. Placebo consisting of colour-matched blueberry-flavoured placebo powder	Task-switching test, trail making test, California verbal learning test, digit span test, virtual Morris water maze attention and attention network task	Geriatric depression scale and profile of mood states. Mobility assessment via dynamic stance and gait analysis	Blueberry led to reduced repetition errors on word list recall and increased accuracy during task switching at 12 weeks
Neshatdoust *et al.*[57]	$n = 40$ (45 % female) healthy older adults (mean age 68 years)	Double-blind, crossover. Each treatment consumed for 4 weeks with a 4-week washout	High-flavanol cocoa drink (494 mg total flavanols) or low-flavanol drink (23 mg total flavanols). Standardised for their macro- and micronutrient content, energy content, theobromine and caffeine content	Go-NoGo task, Stroop, plus-minus, trail making tasks, letter memory task, free and delayed recall, word and face recognition, serial sevens, spatial delayed recall task, virtual 3D radial arm maze task, word stem completion task, digit symbol substitution task and rapid visual information processing	Serum brain-derived neurotrophic factor (BDNF)	High flavanol led to increased global cognitive function and increased serum BDNF levels
Whyte *et al.*[52]	$n = 122$ (61 % female), healthy adults aged 65–80 years (mean age 70 years)	Double-blind, parallel groups. Tested at baseline, 12 and 24 weeks	Wild blueberry powder 500 mg, wild blueberry powder 1000 mg, wild blueberry extract 100 mg and placebo powder (consisting of maltodextrin and food grade artificial dye) colour-matched to treatment	Rey auditory verbal learning task, object recognition task, Corsi blocks, Sternberg memory scanning task, modified attention network task and Stroop task	Positive and negative affect schedule-NOW to determine mood. Blood pressure and heart rate taken after the first half of the cognitive task battery	Purified blueberry extract improved episodic memory and visual memory span at 12 but not 24 weeks. Systolic blood pressure reduced at 12 and 24 weeks

and visual memory span at 12 but not 24 weeks compared with placebo[52]. Interestingly, the purified extract contained negligible fat, fibre, vitamin C and sugars, which were present in the other blueberry interventions. This indicates that the observed effects are attributable to the flavonoid content alone rather than a synergy with these other components. The lack of effects at 24 weeks is difficult to explain, particularly as systolic blood pressure was reduced at 12 and 24 weeks, but a tentative explanation is that this is reflective of a practice effect whereby improved strategy in performing the tasks has reduced task sensitivity. Twelve weeks' consumption of 4.9 g/day freeze-dried cranberry extract (equivalent to one cup of fresh cranberries) was also shown to improve episodic memory in 50–80-year-olds[53]. Reduced LDL cholesterol was also shown with some evidence for sex differences in response, as well as increased perfusion in specific regions of the right brain. This is contrary to an earlier study showing no effects of cranberry juice on a range of cognitive measures in healthy older adults[54]. These null findings may be due to the form or dose of cranberry administered, with a 909 ml beverage containing 27 % cranberry juice being delivered, or they could indicate that a 6-week intervention was not sufficient to elicit an effect on cognitive performance in this population.

The effects of cocoa should also be considered here. While not typically thought of as a fruit, cocoa represents the seed of the fruit of the cacao tree and has been studied for potential benefits due to its flavan-3-ol content. In one such study, high flavan-3-ol (993 mg), intermediate flavan-3-ol (520 mg) or low flavan-3-ol control (48 mg) were given for 8 weeks. Improvements to information processing and a measure of executive function were shown following both flavan-3-ol doses and the highest dose also improved verbal fluency[55]. Importantly, no effects were observed on the MMSE, highlighting the lack of sensitivity of this measure in short intervention trials of healthy adults. This study also attempted to ascertain the determinants of cognitive change, observing that insulin resistance explained 17 % of cognitive change. Systolic blood pressure and lipid peroxidation were not predictors despite blood pressure and several metabolic markers being improved by the intervention. Another interesting study of cocoa showed that high flavan-3-ol (900 mg) improved novel object recognition reaction time when compared with control. Moreover, the authors reported that the improvement observed following intake of high flavan-3-ol cocoa for 12 weeks was equivalent to three decades of ageing, shifting the slope in expected age-related decline. Imaging data also showed an increase in cerebral blood volume in the dentate gyrus region of the hippocampus, and this increase was inversely correlated with reaction time. This indicates a potential mechanism of action for improvements linked to increased blood flow, which may be specific to the hippocampus given the localised effect and the role of the hippocampus in the task assessed[56]. Significant improvements to global cognition were also observed in healthy elderly following 28 days' supplementation with 494 mg of cocoa flavan-3-ol. Interestingly, increases in serum brain-derived neurotrophic factor levels were also observed[57].

Studies of nuts have produced mixed findings. In the dual-centre Walnuts And Healthy Aging (WAHA) study, there were no effects of a 2-year walnut intervention[58]. However, a site-specific improvement to global cognition and fMRI differences indicative of improved neural efficiency in the walnut group were observed following 30–60 g/day walnuts over 2 years. A study assessing effects of 12-week almond consumption failed to find effects on cognition when compared with an isoenergetic carbohydrate-rich snack in overweight and obese older adults, despite improvements to aspects of cardiometabolic health[59]. However, 12-week peanut consumption was observed to enhance memory, verbal fluency and processing speed, as well as increasing cerebrovascular reactivity and small artery elasticity in healthy overweight adults[60]. Changes in cerebrovascular reactivity in the left middle cerebral artery were also positively correlated with changes in delayed memory and recognition. Although peanuts are legumes and therefore classified as vegetables, they are usually considered in the nut category due to their composition. These nut studies illustrate the issue of implementing a control in studies of whole foods. In two of the studies, the control arm consisted of habitual diet minus nuts[58,60], whereas in the other study nuts were compared with an isoenergetic snack[59]. The first option presents issues in that no intervention is present in the control arm, whereas the snack comparator makes interpreting findings difficult due to potential effects of the composition of the control employed.

RCT of vegetables are less prevalent than fruit, possibly due to the logistics of developing a control for the intervention. Fruit is typically studied in juice form, but vegetable juice is less commonly consumed and is potentially less likely to be accepted by participants where daily intake is required. For instance, there are a number of studies of beetroot juice, but only one of these has included repeated consumption in healthy older adults[36]. A 13-week trial in overweight and obese older adults assessed effects of high (800 mg/day), moderate (400 mg/day) and low (200 mg/day) nitrate administered in beetroot juice and compared effects to a nitrate-depleted beetroot juice. Despite a wide array of cognitive tasks, there was no evidence of any effects of beetroot juice on cognition and no effects on cerebral blood volume as assessed with near IR spectroscopy. This study was designed as a feasibility study and not intended to provide a definitive investigation of the effects of nitrate on cognition and cerebral blood flow, but the null findings are unexpected based upon the available evidence from short-term studies.

Evidence for cognitive benefits of fruit and vegetables from RCT is weaker than epidemiological data. There are generally only one or two RCT per food type, with the strongest evidence for cocoa where three studies exist. Although the available data are limited, the findings are generally positive despite potential issues with short duration of intervention and limited knowledge of optimal dosage. Not all studies show positive effects, the dual-centre WAHA study[58] failed to detect effects of a 2-year walnut intervention when analysed in the population as whole. However, subgroup analysis revealed a site-specific improvement. Compared with other sites, participants at the site where improvement was observed, reported lower education and lower intake of the plant n-3, α-linolenic acid[58]. These findings potentially suggest that those at risk, either due to poorer nutrition or poorer education, may be more susceptible to cognitive improvements following a fruit or vegetable intervention. A number of studies[53,55,56,60] indicate increased cerebral blood volume following fruit/legume intervention, as well as improved cardiometabolic markers, and in some cases a correlation between changes in cognition and cerebrovascular/metabolic markers. These findings may suggest that cognitive improvement is more likely in those who are at risk for cardiometabolic dysfunction.

However, RCT assessing effects of diet on cognition often impose strict exclusion criteria such as no clinically significant coexisting medical conditions, including CVD, cerebrovascular events, neurological disorders, inflammatory diseases, metabolic disorders, gastrointestinal abnormalities, mental illness including

depression; no prescription medication; no supplements; no current smokers and no individuals with BMI $\geq$30 kg/m^2. While these criteria are sensible in terms of study design and minimising ‘noise’ from uncontrolled variables, they present a problem in terms of representativeness. For instance, 63 % of adults in England aged 65–74 years reported a longstanding health condition in 2018 [61]. In adults aged $\geq$65 years, at least 80 % were shown to have taken a prescribed medication in the past week, and more than 1 in 10 took at least eight different prescribed medications per week[62], while 75 % of 65–74-year-olds were reported to be overweight or obese[61]. In addition to problems relating to generalisability of findings, these studies create somewhat of a paradox in that they are exploring the importance of nutrition to health yet recruiting unusually healthy people while assuming suboptimal nutrition that can be enhanced by intervention.

Nutrition status and self-selection

Inclusion of nutrition status measures, such as dietary intake measures and nutritional biomarkers, in RCT of nutrition interventions on cognition is increasing, but still rare. Failure to measure nutritional status in cognitive studies presents obvious problems in not being able to ascertain if any null findings are due to already optimal status in study participants[63]. Indeed, post hoc analyses indicate that beneficial effects of vitamins A, C and certain B-vitamins on cognitive decline may only be shown in those with low baseline intake (for review see[64]). However, due to self-selection bias, those who volunteer for RCT of nutrition are likely to be health conscious and potentially less likely to display suboptimal nutrition. This is nicely illustrated by the Memory and Attention Supplementation Trial where a validated (against blood markers) questionnaire was used to assess diet quality prior to enrolment, aiming for 50 % ‘optimal’ diet (a high intake of fruit, vegetables, legumes, olive oil and nuts, and a lower intake of processed foods) and 50 % suboptimal. Out of 501 volunteers, 461 (74.4 %) met the criterion for an ‘optimal’ diet indicative of elevated nutrient status[65]. When considered against the statistic that only 5.4 % of Australian adults meet the current recommendations for both fruit and vegetable intake, this again highlights the lack of representativeness of cognition-nutrition research volunteers. Since multivitamin use is more common in females and those with healthier lifestyle habits, higher educational attainment, higher socio-economic status and of lower BMI[66], it is potentially more likely that those who participate in nutrition research will share similar characteristics. This is supported by evidence from the Monongahela Valley Independent Elders Survey, where those who volunteered to participate were more likely to be women and had more formal years of education, higher cognitive test scores, higher Instrumental Activities of Daily Living ability and lower mortality rates than randomly selected participants[67]. This indicates a further issue with cognitive reserve since those with more years of formal education and who engage in more physical activity are at reduced risk of cognitive decline[68]. Higher cognitive test scores in those who volunteer may also indicate the presence of ceiling effects in study participants, again increasing the likelihood of null findings.

Conclusion

There is growing evidence for an association between fruit and vegetable intake and cognitive function, but this is not always consistent. Some of the differences observed in findings may relate to not only the populations studied but also the measures used to assess fruit and vegetable intake – for instance a fruit and vegetable-specific FFQ versus simply asking how many portions of fruit and vegetable are consumed per day, as well as the covariates included in the analyses. Nevertheless, epidemiological support for beneficial effects of fruit and vegetables tends to be stronger than that from RCT. This is not uncommon and is to be expected given the limited information available on dose and duration required to see a measurable effect in RCT. This is further complicated in older populations who may experience issues with absorption of certain micronutrients[69]. Restrictive inclusion/exclusion criteria may also impact findings since those who meet these requirements are likely to be already consuming a healthy diet and may not benefit from additional nutrient intervention. Given the large proportion of older adults who consume a suboptimal diet and who suffer ill health, it is imperative that steps are taken to ensure inclusion in RCT of those most at risk for cognitive decline, who are most likely to receive the greatest benefit. Racial diversity is also lacking in studies of nutrition and cognition, and this is particularly important when considering that race has been shown to modify relationships between cardiovascular factors and cognition[11] and between nutrition and cognition[70]. Sex has also been observed to modify the relationship between nutrition and cognition[70], and sex differences in cognition[71,72], nutrient requirements[73] and pathways by which dietary nutrients and gut microbes modify metabolism and immunity[74] all indicate that sex effects should be considered in dietary interventions on cognition but rarely are. This is particularly important in older adults due to the impact of not only menopause but also the higher prevalence of dementia in women than men[75]. Data showing that an increment of 100 g per day of fruit and vegetable consumption is related to approximately 13 % reduction in dementia risk[76] highlight the importance of research in this field and the necessity to ensure that those most at risk are included in this research. This review highlights epidemiological evidence for a relationship between fruit and vegetable intake and cognition, which appears to be stronger for vegetables than fruit. However, RCT exploring effects of vegetables on cognition are lacking. Further research is required exploring effects of a range of different fruits and vegetables, including different forms of fruits and vegetables, on cognition, particularly in those at risk for cognitive decline.

Acknowledgements. None.

Author contributions. Both authors contributed to the writing of this manuscript.

Financial support. This research received no specific grant from any funding agency, commercial or not-for-profit sectors.

Competing interests. None.

References

1. World Health Organization (2022) Ageing and health. https://wwwwhoint/news-room/fact-sheets/detail/ageing-and-health
2. Wittenberg R, Hu B, Barraza-Araiza L, *et al.* (2019) *Projections of Older People with Dementia and Costs of Dementia Care in the United Kingdom, 2019–2040*. London: Care Policy and Evaluation Centre, London School of Economics and Political Science.
3. Gong J, Harris K, Lipnicki DM, *et al.* (2023) Sex differences in dementia risk and risk factors: Individual-participant data analysis using 21 cohorts across six continents from the COSMIC consortium. *Alzheimers Dement* **19**, 3365–3378.

4. Cena H & Calder PC (2020) Defining a healthy diet: evidence for the role of contemporary dietary patterns in health and disease. *Nutrients* **12**, 334.
5. Murray CJ, Atkinson C, Bhalla K, *et al.* (2013) The state of US health, 1990–2010: burden of diseases, injuries, and risk factors. *JAMA* **310**, 591–608.
6. GBD 2017 Diet Collaborators (2019) Health effects of dietary risks in 195 countries, 1990–2017: a systematic analysis for the Global Burden of Disease Study 2017. *Lancet* **393**, 1958–1972.
7. Solomon A, Kåreholt I, Ngandu T, *et al.* (2009) Serum total cholesterol, statins and cognition in non-demented elderly. *Neurobiol Aging* **30**, 1006–1009.
8. Ma C, Yin Z, Zhu P, *et al.* (2017) Blood cholesterol in late-life and cognitive decline: a longitudinal study of the Chinese elderly. *Mol Neurodegener* **12**, 24.
9. Gottesman RF, Albert MS, Alonso A, *et al.* (2017) Associations between midlife vascular risk factors and 25-year incident dementia in the atherosclerosis risk in communities (ARIC) cohort. *JAMA Neurol* **74**, 1246–1254.
10. Kivipelto M, Helkala EL, Laakso MP, *et al.* (2001) Midlife vascular risk factors and Alzheimer's disease in later life: longitudinal, population based study. *BMJ* **322**, 1447–1451.
11. Gottesman RF, Schneider AL, Albert M, *et al.* (2014) Midlife hypertension and 20-year cognitive change: the atherosclerosis risk in communities neurocognitive study. *JAMA Neurol* **71**, 1218–1227.
12. Launer LJ, Masaki K, Petrovitch H, *et al.* (1995) The association between midlife blood pressure levels and late-life cognitive function. The Honolulu-Asia Aging Study. *JAMA* **274**, 1846–1851.
13. Gong J, Harris K, Peters SAE, *et al.* (2021) Sex differences in the association between major cardiovascular risk factors in midlife and dementia: a cohort study using data from the UK Biobank. *BMC Med* **19**, 110.
14. Tsao CW, Seshadri S, Beiser AS, *et al.* (2013) Relations of arterial stiffness and endothelial function to brain aging in the community. *Neurology* **81**, 984–991.
15. Jefferson AL, Beiser AS, Himali JJ, *et al.* (2015) Low cardiac index is associated with incident dementia and Alzheimer disease: the Framingham Heart Study. *Circulation* **131**, 1333–1339.
16. Sabayan B, van Buchem MA, Sigurdsson S, *et al.* (2015) Cardiac hemodynamics are linked with structural and functional features of brain aging: the age, gene/environment susceptibility (AGES)-Reykjavik Study. *J Am Heart Assoc* **4**, e001294.
17. Bolay H, Gürsoy-Ozdemir Y, Sara Y, *et al.* (2002) Persistent defect in transmitter release and synapsin phosphorylation in cerebral cortex after transient moderate ischemic injury. *Stroke* **33**, 1369–1375.
18. Sage JI, Van Uitert RL, & Duffy TE (1984) Early changes in blood brain barrier permeability to small molecules after transient cerebral ischemia. *Stroke* **15**, 46–50.
19. Whitmer RA, Sidney S, Selby J, *et al.* (2005) Midlife cardiovascular risk factors and risk of dementia in late life. *Neurology* **64**, 277–281.
20. Hassing LB, Grant MD, Hofer SM, *et al.* (2004) Type 2 diabetes mellitus contributes to cognitive decline in old age: a longitudinal population-based study. *J Int Neuropsychol Soc* **10**, 599–607.
21. Van Dyken P & Lacoste B (2018) Impact of metabolic syndrome on neuroinflammation and the blood-brain barrier. *Front Neurosci* **12**, 930.
22. Varatharaj A & Galea I (2017) The blood-brain barrier in systemic inflammation. *Brain Behav Immun* **60**, 1–12.
23. Huang X, Hussain B, & Chang J (2021) Peripheral inflammation and blood-brain barrier disruption: effects and mechanisms. *CNS Neurosci Ther* **27**, 36–47.
24. Soysal P, Arik F, Smith L, *et al.* (2020) Inflammation, frailty and cardiovascular disease. *Adv Exp Med Biol* **1216**, 55–64.
25. Ferrucci L & Fabbri E (2018) Inflammageing: chronic inflammation in ageing, cardiovascular disease, and frailty. *Nat Rev Cardiol* **15**, 505–522.
26. Cryan JF, O'Riordan KJ, Cowan CSM, *et al.* (2019) The microbiota-gut-brain axis. *Physiol Rev* **99**, 1877–2013.
27. Simpson CA, Diaz-Arteche C, Eliby D, *et al.* (2021) The gut microbiota in anxiety and depression - a systematic review. *Clin Psychol Rev* **83**, 101943.
28. Molendijk M, Molero P, Ortuño Sánchez-Pedreño F, *et al.* (2018) Diet quality and depression risk: a systematic review and dose-response meta-analysis of prospective studies. *J Affect Disord* **226**, 346–354.
29. Minihane AM, Vinoy S, Russell WR, *et al.* (2015) Low-grade inflammation, diet composition and health: current research evidence and its translation. *Br J Nutr* **114**, 999–1012.
30. Coelho-Júnior HJ, Trichopoulou A, & Panza F (2021) Cross-sectional and longitudinal associations between adherence to Mediterranean diet with physical performance and cognitive function in older adults: a systematic review and meta-analysis. *Ageing Res Rev* **70**, 101395.
31. Valls-Pedret C, Sala-Vila A, Serra-Mir M, *et al.* (2015) Mediterranean diet and age-related cognitive decline: a randomized clinical trial. *JAMA Intern Med* **175**, 1094–1103.
32. Willett W, Rockström J, Loken B, *et al.* (2019) Food in the anthropocene: the EAT-Lancet Commission on healthy diets from sustainable food systems. *Lancet* **393**, 447–492.
33. Dalile B, Kim C, Challinor A, *et al.* (2022) The EAT-Lancet reference diet and cognitive function across the life course. *Lancet Planet Health* **6**, e749–e759.
34. Sabia S, Nabi H, Kivimaki M, *et al.* (2009) Health behaviors from early to late midlife as predictors of cognitive function: the Whitehall II study. *Am J Epidemiol* **170**, 428–437.
35. Péneau S, Galan P, Jeandel C, *et al.* (2011) Fruit and vegetable intake and cognitive function in the SU.VI.MAX 2 prospective study. *Am J Clin Nutr* **94**, 1295–1303.
36. Babateen AM, Shannon OM, O'Brien GM, *et al.* (2022) Incremental doses of nitrate-rich beetroot juice do not modify cognitive function and cerebral blood flow in overweight and obese older adults: a 13-week pilot randomised clinical trial. *Nutrients* **14**, 1052.
37. Yuan C, Fondell E, Bhushan A, *et al.* (2019) Long-term intake of vegetables and fruits and subjective cognitive function in US men. *Neurology* **92**, e63–e75.
38. Nurk E, Refsum H, Drevon CA, *et al.* (2010) Cognitive performance among the elderly in relation to the intake of plant foods. The Hordaland Health Study. *Br J Nutr* **104**, 1190–1201.
39. Ye X, Bhupathiraju SN, & Tucker KL (2013) Variety in fruit and vegetable intake and cognitive function in middle-aged and older Puerto Rican adults. *Br J Nutr* **109**(3), 503–510.
40. Nooyens AC, Bueno-de-Mesquita HB, van Boxtel MP, *et al.* (2011) Fruit and vegetable intake and cognitive decline in middle-aged men and women: the Doetinchem Cohort Study. *Br J Nutr* **106**, 752–761.
41. Kang JH, Ascherio A, & Grodstein F. (2005) Fruit and vegetable consumption and cognitive decline in aging women. *Ann Neurol* **57**, 713–720.
42. Morris MC, Evans DA, Tangney CC, *et al.* (2006) Associations of vegetable and fruit consumption with age-related cognitive change. *Neurology* **67**, 1370–1376.
43. Morris MC, Wang Y, Barnes LL, *et al.* (2018) Nutrients and bioactives in green leafy vegetables and cognitive decline: prospective study. *Neurology* **90**, e214–e222.
44. Devore EE, Kang JH, Breteler MM, *et al.* (2012) Dietary intakes of berries and flavonoids in relation to cognitive decline. *Ann Neurol* **72**, 135–143.
45. Cheng FW, Ford NA, & Taylor MK (2021) US older adults that consume avocado or guacamole have better cognition than non-consumers: National Health and Nutrition Examination Survey 2011–2014. *Front Nutr* **8**, 746453.
46. O'Brien J, Okereke O, Devore E, *et al.* (2014) Long-term intake of nuts in relation to cognitive function in older women. *J Nutr Health Aging* **18**, 496–502.
47. Nicodemus-Johnson J & Sinnott RA (2017) Fruit and juice epigenetic signatures are associated with independent immunoregulatory pathways. *Nutrients* **9**, 752.
48. Butchart C, Kyle J, McNeill G, *et al.* (2011) Flavonoid intake in relation to cognitive function in later life in the Lothian Birth Cohort 1936. *Br J Nutr* **106**, 141–148.
49. Kean RJ, Lamport DJ, Dodd GF, *et al.* (2015) Chronic consumption of flavanone-rich orange juice is associated with cognitive benefits: an 8-wk, randomized, double-blind, placebo-controlled trial in healthy older adults. *Am J Clin Nutr* **101**, 506–514.

50. Chai SC, Jerusik J, Davis K, *et al.* (2019) Effect of Montmorency tart cherry juice on cognitive performance in older adults: a randomized controlled trial. *Food Funct* **10**, 4423–4431.
51. Miller MG, Hamilton DA, Joseph JA, *et al.* (2018) Dietary blueberry improves cognition among older adults in a randomized, double-blind, placebo-controlled trial. *Eur J Nutr* **57**, 1169–1180.
52. Whyte AR, Cheng N, Fromentin E, *et al.* (2018) A randomized, double-blinded, placebo-controlled study to compare the safety and efficacy of low dose enhanced wild blueberry powder and wild blueberry extract (ThinkBlue™) in maintenance of episodic and working memory in older adults. *Nutrients* **10**, 660.
53. Flanagan E, Cameron D, Sobhan R, *et al.* (2022) Chronic consumption of cranberries (Vaccinium macrocarpon) for 12 weeks improves episodic memory and regional brain perfusion in healthy older adults: a randomised, placebo-controlled, parallel-groups feasibility study. *Front Nutr* **9**, 849902.
54. Crews WD, Jr., Harrison DW, Griffin ML, *et al.* (2005) A double-blinded, placebo-controlled, randomized trial of the neuropsychologic efficacy of cranberry juice in a sample of cognitively intact older adults: pilot study findings. *J Altern Complement Med* **11**, 305–309.
55. Mastroiacovo D, Kwik-Uribe C, Grassi D, *et al.* (2015) Cocoa flavanol consumption improves cognitive function, blood pressure control, and metabolic profile in elderly subjects: the Cocoa, Cognition, and Aging (CoCoA) Study–a randomized controlled trial. *Am J Clin Nutr* **101**, 538–548.
56. Brickman AM, Khan UA, Provenzano FA, *et al.* (2014) Enhancing dentate gyrus function with dietary flavanols improves cognition in older adults. *Nat Neurosci* **17**, 1798–1803.
57. Neshatdoust S, Saunders C, Castle SM, *et al.* (2016) High-flavonoid intake induces cognitive improvements linked to changes in serum brain-derived neurotrophic factor: two randomised, controlled trials. *Nutr Healthy Aging* **4**, 81–93.
58. Sala-Vila A, Valls-Pedret C, Rajaram S, *et al.* (2020) Effect of a 2-year diet intervention with walnuts on cognitive decline. The Walnuts And Healthy Aging (WAHA) study: a randomized controlled trial. *Am J Clin Nutr* **111**, 590–600.
59. Coates AM, Morgillo S, Yandell C, *et al.* (2020) Effect of a 12-week almond-enriched diet on biomarkers of cognitive performance, mood, and cardiometabolic health in older overweight adults. *Nutrients* **12**, 1180.
60. Barbour JA, Howe PRC, Buckley JD, *et al.* (2017) Cerebrovascular and cognitive benefits of high-oleic peanut consumption in healthy overweight middle-aged adults. *Nutr Neurosci* **20**, 555–562.
61. Conolly A & Craig S (2019) Health Survey for England 2018. https://digitalnhsuk/data-and-information/publications/statistical/health-survey-for-england/2018
62. Moody A, Mindell J, & Faulding S (2017) Health Survey for England 2016. https://digitalnhsuk/data-and-information/publications/statistical/health-survey-for-england/health-survey-for-england-2016
63. Morris MC & Tangney CC (2011) A potential design flaw of randomized trials of vitamin supplements. *JAMA* **305**, 1348–1349.
64. Morris MC (2012) Nutritional determinants of cognitive aging and dementia. *Proc Nutr Soc* **71**, 1–13.
65. Young LM, Gauci S, Scholey A, *et al.* (2020) Self-selection bias: an essential design consideration for nutrition trials in healthy populations. *Front Nutr* **7**, 587983.
66. Comerford KB (2013) Recent developments in multivitamin/mineral research. *Adv Nutr* **4**, 644–656.
67. Ganguli M, Lytle ME, Reynolds MD, *et al.* (1998) Random versus volunteer selection for a community-based study. *J Gerontol A Biol Sci Med Sci* **53**, M39–M46.
68. Baumgart M, Snyder HM, Carrillo MC, *et al.* (2015) Summary of the evidence on modifiable risk factors for cognitive decline and dementia: a population-based perspective. *Alzheimers Dement* **11**, 718–726.
69. Soenen S, Rayner CK, Jones KL, *et al.* (2016) The ageing gastrointestinal tract. *Curr Opin Clin Nutr Metab Care* **19**, 12–18.
70. Gu Y, Guo J, & Moshfegh AJ (2021) Race/ethnicity and gender modify the association between diet and cognition in U.S. older adults: National Health and Nutrition Examination Survey 2011–2014. *Alzheimers Dement (N Y)* **7**, e12128.
71. Herlitz A & Rehnman J (2008) Sex differences in episodic memory. *Curr Dir Psychol Sci* **17**, 52–56.
72. Duff SJ & Hampson E (2001) A sex difference on a novel spatial working memory task in humans. *Brain Cogn* **47**, 470–493.
73. Prentice A (2021) Sex differences in requirements for micronutrients across the lifecourse. *Proc Nutr Soc* **80**, 356–364.
74. Israelian N & Danska JS (2017) Sex effects at the ramparts: nutrient- and microbe-mediated regulation of the immune-metabolic interface. *Adv Exp Med Biol* **1043**, 113–140.
75. Cao Q, Tan CC, Xu W, *et al.* (2020) The prevalence of dementia: a systematic review and meta-analysis. *J Alzheimers Dis* **73**, 1157–1166.
76. Jiang X, Huang J, Song D, *et al.* (2017) Increased consumption of fruit and vegetables is related to a reduced risk of cognitive impairment and dementia: meta-analysis. *Front Aging Neurosci* **9**, 18.
