# Supplementary material for: Dietary fibre intake in the adult Swiss population: a comprehensive analysis of timing and sources
Source: J Nutr Sci. 2025 Mar 24;14:e27. doi: 10.1017/jns.2025.6 (PMC11950702; doi:10.1017/jns.2025.6)
Supplement: von Blumenthal et al. supplementary material 1 — von Blumenthal et al. supplementary material [file S2048679025000060sup001.zip › menuCH_supplementary_information_012025.docx]

Dietary Fibre intake in the Adult Swiss Population: A Comprehensive Analysis of Timing and Sources

Flurina von Blumenthal^1^, Katja A. Schönenberger^1,2^, Valentina V. Huwiler^1,2^, Zeno Stanga^1^, Giulia Pestoni^3,4,^ *, David Faeh^4,5,^ *

^1^ Department of Diabetes, Endocrinology, Nutritional Medicine and Metabolism (UDEM), Inselspital Bern, University Hospital, Bern, Switzerland

^2^ Division of Clinical Pharmacy and Epidemiology, Department of Pharmaceutical Sciences, University of Basel, Basel, Switzerland

^3^ Nutrition Group, Health Department, Swiss Distance University of Applied Sciences (FFHS) / University of Applied Sciences and Arts of Southern Switzerland (SUPSI), Zurich, Switzerland

^4^ Division of Chronic Disease Epidemiology, Epidemiology, Biostatistics and Prevention Institute, University of Zurich, Zurich, Switzerland

^5^ Health Department, Bern University of Applied Sciences, Bern, Switzerland

* Joint senior authors: Giulia Pestoni, David Faeh

Corresponding author: Dr. Giulia Pestoni, giulia.pestoni@ffhs.ch

**ORCID ID**

F. von Blumenthal: 0009-0004-8793-1111; Katja A. Schönenberger: 0000-0002-2122-4746; Valentina V. Huwiler: 0000-0002-0357-6887; Zeno Stanga: 0000-0002-8630-2477; Giulia Pestoni: 0000-0002-5933-7439; David Faeh: 0000-0003-2630-8102

**Author contributions**

Conceptualization: Flurina von Blumenthal, Katja A. Schönenberger, Zeno Stanga, Giulia Pestoni, David Faeh; Data curation: Flurina von Blumenthal, Katja A. Schönenberger, Giulia Pestoni; Formal Analysis: Flurina von Blumenthal, Katja A. Schönenberger; Supervision: Katja A. Schönenberger, Giulia Pestoni, Zeno Stanga, David Faeh; Visualization: Flurina von Blumenthal, Katja A. Schönenberger; Writing – original draft: Flurina von Blumenthal; Writing – review & editing: Flurina von Blumenthal, Katja A. Schönenberger, Valentina V. Huwiler, Giulia Pestoni, David Faeh, Zeno Stanga

**Short title:** Sources and timing of dietary fibre intake

**Keywords:** dietary fibre, 24h dietary recall, whole grain, nutrition survey

**Supplementary Table 1:** Description of foods and beverages included in the 22 Categories

| **Food categories** | **Food items** |
| --- | --- |
|  |  |
| Non-alcoholic beverages | Soft drinks (e.g., soda, lemonade), flavored waters, alcohol-free beers, and juices (e.g., orange, apple, carrot). |
| Milk, milk products | Milk, yogurt (e.g., Activia), cheese (e.g., Swiss specialties), and kefir. |
| Alcoholic beverages | Spirits (e.g., absinthe, amaretto), liquors (e.g., Alpenbitter), alcopops, and aperitifs. |
| Refined Grains | White bread, bagels, shortcrust pastry, and sandwich rolls. |
| Vegetables | Leafy greens, root vegetables, mushrooms, and seaweed (e.g., nori). |
| Fruits | Fresh fruits (e.g., apples, bananas, apricots), tropical fruits (e.g., pineapples), dried fruits. |
| Meat, meat products | Processed meats (e.g., sausage, wurst), deli meats, and traditional Swiss meat dishes. |
| Potatoes, other starchy root tuber | Potatoes (e.g., mashed, fries), gnocchi, and root vegetables. |
| Seasoning, spices, herbs and sauces | Vinegars (e.g., balsamic), herbs (e.g., oregano), spices (e.g., curry), and condiment mixes. |
| Soups, bouillons | Prepared broths (e.g., chicken, vegetable), consommés, and instant soups. |
| Whole Grains | Whole-grain bread, muesli, porridge, and grain mixes (e.g., amaranth, spelt). |
| Cakes, patisserie | Cookies (e.g., amaretti), pastries (e.g., apple strudel), Swiss specialties (e.g., Leckerli). |
| Sugar, sweets, chocolate | Chocolates, syrups (e.g., maple, agave), and confectionery (e.g., candy bars). |
| Fish and seafood | Fish (e.g., tuna, cod), shellfish (e.g., shrimp, lobster), and processed seafood (e.g., fish sticks). |
| Fats, oils | Butter (salted, unsalted, clarified), plant-based spreads, and flavored butters (e.g., herb butter). |
| Eggs and egg products | Whole eggs, egg whites, yolks, and prepared egg dishes (e.g., omelets, flädli [soup strips]). |
| Salty snacks | Savory pastries, crackers, pretzels, and breadsticks. |
| Nuts, seeds | Nuts (e.g., chestnuts, peanuts), seeds (e.g., pumpkin seeds), and nut-based spreads. |
| Dairy substitutes | Plant-based milks (e.g., soy milk, rice milk), plant-based yogurts, and coconut milk. |
| Legumes | Beans (e.g., kidney, soy), lentils, and chickpeas. |
| Various foods | Sugar substitutes, energy bars, and miscellaneous (e.g., gelatin, falafel). |
| Meat substitutes | Processed meats (e.g., sausage, wurst), deli meats, and traditional Swiss meat dishes. |

This table is based on the work of Chatelan et al. ^(1)^

**Supplementary table 2:** Mean food intake from food categories in the *menuCH* population overall and by absolute and relative dietary fibre intake groups (n = 2,057)

| **Food category** | **Intake from Food category [g/day], mean (SD)** | | | | | | |
| --- | --- | --- | --- | --- | --- | --- | --- |
|  | **Overall**  **(n=2057)** | **Absolute dietary fibre intake** | | | **Relative dietary fibre intake** | | |
|  |  | **<15 g/day, (n=535)** | **15-<30 g/day, (n=1257)** | **>=30 g/day, (n=265)** | **<10**  **g/1000 kcal/day, (n=1206)** | **10-<14**  **g/1000 kcal/day, (n=606)** | **>= 14**  **g/1000 kcal/day, (n=245)** |
| Non-alcoholic beverages | 2,032 (817) | 1,914 (817) | 2,024 (782) | 2,290 (911) | 1,997 (812) | 2,025 (796) | 2,235 (866) |
| Milk, milk products | 242 (197) | 202 (164) | 250 (201) | 284 (219) | 257 (213) | 231 (172) | 196 (156) |
| Alcoholic beverages | 198 (312) | 198 (308) | 204 (321) | 171 (271) | 262 (368) | 122 (170) | 58 (120) |
| Refined Grains | 195 (134) | 153 (94) | 205 (131) | 226 (184) | 216 (135) | 177 (125) | 134 (124) |
| Vegetables | 173 (122) | 114 (79) | 175 (108) | 278 (167) | 141 (96) | 201 (118) | 272 (173) |
| Fruits | 171 (171) | 74 (86) | 171 (145) | 351 (240) | 106 (111) | 239 (180) | 340 (220) |
| Meat, meat products | 104 (87) | 117 (84) | 104 (85) | 81 (97) | 127 (90) | 77 (68) | 49 (65) |
| Potatoes, other starchy root tuber | 51 (68) | 35 (50) | 55 (69) | 62 (87) | 51 (67) | 53 (70) | 42 (71) |
| Seasoning, spices, herbs and sauces | 49 (50) | 40 (38) | 49 (49) | 65 (66) | 52 (49) | 48 (50) | 42 (50) |
| Soups, bouillons | 46 (101) | 41 (87) | 46 (103) | 59 (112) | 40 (92) | 53 (109) | 63 (120) |
| Whole Grains | 41 (56) | 14 (22) | 39 (44) | 106 (91) | 24 (36) | 60 (62) | 84 (83) |
| Cakes, patisserie | 37 (53) | 28 (40) | 40 (55) | 42 (58) | 43 (59) | 29 (39) | 28 (42) |
| Sugar, sweets, chocolate | 36 (35) | 25 (29) | 38 (35) | 45 (41) | 39 (38) | 33 (30) | 27 (28) |
| Fish and seafood | 21 (40) | 20 (38) | 21 (40) | 25 (43) | 21 (41) | 21 (40) | 22 (40) |
| Fats, oils | 20 (16) | 14 (12) | 21 (16) | 26 (20) | 20 (17) | 21 (16) | 17 (14) |
| Eggs and egg products | 13 (23) | 14 (21) | 12 (21) | 17 (32) | 14 (23) | 11 (19) | 12 (29) |
| Salty snacks | 10 (29) | 7 (18) | 11 (32) | 10 (33) | 12 (32) | 7 (25) | 6 (20) |
| Nuts, seeds | 8 (18) | 3 (6) | 7 (14) | 19 (36) | 5 (10) | 9 (17) | 19 (37) |
| Dairy substitutes | 7 (39) | 3 (22) | 6 (34) | 19 (70) | 3 (22) | 8 (37) | 27 (83) |
| Legumes | 5 (21) | 1 (7) | 4 (20) | 12 (37) | 2 (11) | 7 (26) | 14 (40) |
| Various foods | 3 (23) | 4 (30) | 1 (11) | 10 (43) | 2 (21) | 3 (24) | 8 (31) |
| Meat substitutes | 2 (11) | 1 (6) | 2 (10) | 5 (20) | 1 (8) | 2 (11) | 6 (22) |

Values are weighted for age group, sex, marital status, major region of Switzerland, nationality, household size, season, and weekdays following the *menuCH* weighting strategy ^(2)^.

# Sensitivity Analysis

**Supplementary Table 3:** Mean dietary fibre intake on food consumption occasions in the *menuCH* population overall and by absolute and relative dietary fibre intake groups excluding pregnant and lactating women (n = 2,030)

| **Food consumption occasion** | **Dietary fibre intake [g/day], mean (SD)** | | | | | | |
| --- | --- | --- | --- | --- | --- | --- | --- |
|  | **Overall**  **(*n* = 2,030)** | **Absolute dietary fibre intake** | | | **Relative dietary fibre intake** | | |
|  |  | **<15 g/day**, (n = 530) | **15-<30 g/day**, (n = 1239) | **>=30 g/day**, (n = 261) | **<10 g/1000 kcal/day**, (n = 1191) | **10-<14 g/1000 kcal/day**, (n = 597) | **>=14 g/1000 kcal/day**, (n = 242) |
| Total | 20.7 (8.8) | 11.4 (2.5) | 20.9 (3.9) | 37.1 (7.9) | 17.1 (6.1) | 24.1 (7.5) | 31.5 (11.3) |
| Before breakfast | 0.1 (0.5) | 0.1 (0.2) | 0.1 (0.6) | 0.1 (0.5) | 0.1 (0.4) | 0.2 (0.7) | 0.1 (0.5) |
| Breakfast | 4.1 (3.6) | 1.9 (1.7) | 4.1 (2.9) | 8.2 (5.0) | 3.0 (2.6) | 5.2 (3.6) | 7.3 (4.9) |
| During the morning | 1.2 (2.0) | 0.6 (1.0) | 1.2 (1.7) | 2.7 (3.7) | 0.9 (1.4) | 1.6 (2.4) | 2.0 (3.2) |
| Lunch | 6.0 (3.6) | 3.7 (2.0) | 6.1 (3.0) | 9.7 (5.0) | 5.1 (2.9) | 6.6 (3.6) | 9.0 (4.8) |
| During the afternoon | 1.9 (2.3) | 1.1 (1.2) | 2.0 (2.0) | 3.5 (3.8) | 1.6 (1.9) | 2.3 (2.6) | 2.8 (3.0) |
| Dinner | 6.4 (4.0) | 3.8 (1.9) | 6.5 (3.1) | 11.0 (5.7) | 5.5 (3.2) | 7.2 (4.0) | 8.9 (5.8) |
| After dinner | 1.0 (1.8) | 0.4 (0.8) | 1.1 (1.7) | 1.8 (2.9) | 0.9 (1.5) | 1.1 (2.0) | 1.3 (2.3) |

**Supplementary Table 4:** Mean dietary fibre intake from food categories in the *menuCH* population overall and by absolute and relative dietary fibre intake groups excluding pregnant and lactating women (n = 2,030)

| **Food category^a^** | **Dietary fibre intake from food category [g/day], mean (SD)** | | | | | | | **Standard fibre content of food category based on Swiss Food Composition Database [g/100g], mean^b^** |
| --- | --- | --- | --- | --- | --- | --- | --- | --- |
|  | **Overall**  **(*n* = 2,030)** | **Absolute dietary fibre intake** | | | **Relative dietary fibre intake** | | |  |
|  |  | **<15 g/day**, (n = 530) | **15-<30 g/day**, (n = 1,239) | **>=30 g/day** (*n* = 265) | **<15 g/day**, (n = 530) | **15-<30 g/day**, (n = 1,239) | **>=14 g/1000 kcal/day**  (*n* = 245) |  |
| Refined grains | 4.4 (3.3) | 3.1 (2.0) | 4.7 (3.2) | 5.5 (4.9) | 4.8 (3.3) | 4.0 (3.2) | 3.2 (3.5) | 5.5 |
| Whole grains | 3.0 (3.9) | 1.0 (1.5) | 2.8 (3.0) | 7.5 (6.4) | 1.7 (2.5) | 4.3 (4.2) | 6.0 (6.0) | 15.4 |
| Fruits | 3.8 (3.9) | 1.5 (1.7) | 3.7 (3.2) | 8.3 (5.5) | 2.2 (2.3) | 5.4 (3.8) | 8.1 (5.3) | 4.4 |
| Vegetables | 3.8 (2.9) | 2.3 (1.8) | 3.9 (2.5) | 6.2 (4.1) | 3.0 (2.1) | 4.5 (2.8) | 6.4 (4.2) | 3.3 |
| Potatoes, other starchy root tuber | 1.1 (1.4) | 0.7 (1.1) | 1.2 (1.5) | 1.3 (1.8) | 1.1 (1.4) | 1.1 (1.5) | 0.9 (1.5) | 2.3 |
| Cakes, cookies, patisserie | 0.8 (1.2) | 0.6 (0.8) | 0.9 (1.2) | 1.0 (1.7) | 0.9 (1.3) | 0.7 (1.1) | 0.6 (0.9) | 2.7 |
| Sugar, chocolate, sweets | 0.8 (1.0) | 0.4 (0.6) | 0.8 (1.0) | 1.0 (1.1) | 0.8 (1.0) | 0.7 (1.0) | 0.7 (0.8) | 2.9 |
| Nuts, seeds | 0.6 (1.6) | 0.2 (0.5) | 0.5 (1.1) | 1.7 (3.3) | 0.3 (0.7) | 0.7 (1.4) | 1.7 (3.5) | 10.4 |
| Seasoning, spices, herbs, sauces | 0.5 (0.7) | 0.3 (0.4) | 0.5 (0.6) | 0.8 (1.3) | 0.4 (0.6) | 0.5 (0.9) | 0.5 (0.9) | 5.5 |
| Non-alcoholic beverages | 0.5 (1.0) | 0.4 (0.4) | 0.5 (0.7) | 0.7 (2.3) | 0.5 (0.6) | 0.6 (1.3) | 0.6 (1.9) | 5.6 |
| Salty snacks | 0.4 (1.5) | 0.2 (0.5) | 0.3 (1.0) | 0.9 (3.5) | 0.4 (1.1) | 0.3 (1.9) | 0.6 (2.3) | 6.0 |
| Soups | 0.2 (0.6) | 0.2 (0.4) | 0.3 (0.7) | 0.3 (0.8) | 0.2 (0.5) | 0.3 (0.7) | 0.4 (0.8) | 1.2 |
| Milk products | 0.3 (0.7) | 0.2 (0.4) | 0.4 (0.8) | 0.4 (0.9) | 0.3 (0.6) | 0.3 (0.8) | 0.4 (1.0) | 0.1 |
| Legumes | 0.2 (1.1) | 0.1 (0.4) | 0.2 (1.0) | 0.6 (2.1) | 0.1 (0.6) | 0.3 (1.4) | 0.7 (1.9) | 11.5 |

**Supplementary table 5:** Mean food intake from food categories in the *menuCH* population overall and by absolute and relative dietary fibre intake groups excluding pregnant and lactating women (n = 2,030)

| **Food category** | **Intake from Food category [g/day], mean (SD)** | | | | | | |
| --- | --- | --- | --- | --- | --- | --- | --- |
|  | **Overall**  **(n=2030)** | **Absolute dietary fibre intake** | | | **Relative dietary fibre intake** | | |
|  |  | **<15 g/day**, (n = 530) | **15-<30 g/day**, (n = 1239) | **>= 30 g/day**, (n = 261) | **<10 g/1000 kcal/day**, (n = 1191) | **10-<14 g/1000 kcal/day**, (n = 597) | **>= 14 g/1000 kcal/day**, (n = 242) |
| Non-alcoholic beverages | 2,029 (817) | 1,913 (818) | 2,019 (781) | 2,290 (916) | 1,995 (813) | 2,018 (793) | 2,234 (870) |
| Milk, milk products | 242 (197) | 202 (164) | 249 (202) | 283 (220) | 256 (214) | 230 (172) | 196 (157) |
| Alcoholic beverages | 200 (313) | 199 (309) | 206 (322) | 173 (273) | 264 (369) | 123 (171) | 57 (120) |
| Refined Grains | 195 (134) | 153 (94) | 205 (131) | 227 (185) | 215 (136) | 177 (126) | 134 (124) |
| Vegetables | 173 (122) | 114 (80) | 174 (108) | 279 (167) | 141 (96) | 200 (118) | 274 (173) |
| Fruits | 171 (171) | 74 (87) | 171 (145) | 353 (241) | 106 (111) | 239 (180) | 341 (220) |
| Meat, meat products | 104 (87) | 117 (84) | 104 (85) | 82 (98) | 128 (90) | 77 (68) | 49 (65) |
| Potatoes, other starchy root tuber | 51 (68) | 35 (50) | 55 (69) | 62 (87) | 51 (67) | 53 (70) | 42 (71) |
| Seasoning, spices, herbs and sauces | 49 (50) | 40 (38) | 50 (49) | 66 (66) | 52 (49) | 48 (51) | 42 (50) |
| Soups, bouillons | 46 (100) | 41 (87) | 46 (102) | 59 (113) | 40 (92) | 53 (106) | 63 (120) |
| Whole Grains | 41 (56) | 14 (22) | 39 (44) | 104 (91) | 24 (36) | 60 (62) | 83 (83) |
| Cakes, patisserie | 38 (53) | 29 (40) | 40 (56) | 42 (58) | 43 (59) | 29 (39) | 28 (42) |
| Sugar, sweets, chocolate | 36 (35) | 25 (29) | 38 (35) | 45 (41) | 39 (38) | 33 (30) | 27 (28) |
| Fish and seafood | 21 (40) | 20 (38) | 21 (40) | 25 (43) | 21 (40) | 21 (40) | 22 (40) |
| Fats, oils | 20 (16) | 14 (12) | 21 (16) | 26 (20) | 20 (17) | 21 (16) | 16 (13) |
| Eggs and egg products | 13 (23) | 14 (21) | 12 (21) | 17 (31) | 14 (23) | 11 (19) | 12 (29) |
| Salty snacks | 10 (29) | 7 (18) | 11 (32) | 11 (33) | 12 (32) | 7 (26) | 6 (20) |
| Nuts and seeds | 8 (18) | 3 (6) | 7 (14) | 19 (37) | 4 (10) | 9 (17) | 19 (38) |
| Dairy substitutes | 7 (39) | 3 (22) | 6 (34) | 19 (69) | 3 (22) | 8 (37) | 27 (83) |
| Legumes | 5 (22) | 1 (7) | 5 (20) | 12 (38) | 2 (11) | 7 (26) | 14 (40) |
| Various foods | 3 (24) | 4 (30) | 1 (11) | 10 (43) | 2 (21) | 3 (25) | 8 (31) |
| Meat substitutes | 2 (11) | 1 (6) | 2 (10) | 5 (21) | 1 (8) | 2 (11) | 6 (22) |

**References**

1. Chatelan A, Beer-Borst S, Randriamiharisoa A *et al.* (2017) Major Differences in Diet across Three Linguistic Regions of Switzerland: Results from the First National Nutrition Survey menuCH. *Nutrients* 9.

2. Pasquier J CA, Bochud M (2017) Weighting strategy. Institute of Social and Preventive Medicine (IUMSP). [https://menuch.iumsp.ch/index.php/catalog/4/download/17](https://url.avanan.click/v2/r02/___https://menuch.iumsp.ch/index.php/catalog/4/download/17___.YXAxZTpjYW1icmlkZ2Vvcmc6YTpvOjRiNzJjOGE5MWI1NjA0NjkxNjc3N2RhNjNjMTQ0OWRhOjc6ODY1ZTpiYjY5NTYwYjk0YzhiMjZmMGM5MWFjYWQ2ZjM3YzRiYTlmMmEyYWUzZTNlZWUyNTZkY2YzNWU5ZDYzNjVhMjI2OnA6VDpG) (accessed 19 April 2023)

# Figure legends

**Supplementary Figure 1:** Dietary fibre intake on food consumption occasions for absolute (a) and relative (b) dietary fibre intake groups. The bar plot represents the contribution of food consumption occasions, shown as percentage (%) of the total intake of dietary fibre. Total dietary fibre intake refers to the mean dietary fibre intake of both 24 HDRs displayed in Table 2. Food consumption occasions contributing less than 5% to the total amount were not displayed.
