# Supplementary material for: Dietary fibre intake in the adult Swiss population: a comprehensive analysis of timing and sources
Source: J Nutr Sci. 2025 Mar 24;14:e27. doi: 10.1017/jns.2025.6 (PMC11950702; doi:10.1017/jns.2025.6)
Supplement: von Blumenthal et al. supplementary material 2 — von Blumenthal et al. supplementary material [file S2048679025000060sup002.zip › AEE-2300016 (1).pdf]

Australian Journal of Environmental Education (2023), 1–3
doi:10.1017/aee.2023.16

REVIEW

A review of "*Enhancing Environmental Education Through Nature-based Solutions*"

Vasconcelos, C., & Calheiros, C. S. (Eds.). (2022). Enhancing environmental education through nature-based solutions. Springer.

Review Authors
Ahbi Mahdianing Rum[1], Amsal Alhayat[2] and Alfin Anwar[2]

[1]Faculty of Mathematics and Science, Universitas Pendidikan Indonesia and [2]Faculty of Educational Science, Universitas Pendidikan Indonesia
Corresponding author: Ahbi Mahdianing Rum; Email: ahbimahdianingrum@upi.edu

Enhancing Environmental Education Through Nature-Based Solutions is a book edited by Clara Vasconcelos and Cristina S. C. Calheiros which specifically provides information about Environmental Education and how to develop it through Nature-based Solutions (NbS) including what action we should take in order to realise Environmental Education in productive and caring ways. This book was conceptualised under the belief that the younger generation is a great social innovator which will be the future's leaders. This book emphasises that Environmental Education is one of the important keys for us to protect Earth, not only from the inevitable catastrophism run by the rules of the universe but also mainly from our own attitudes and behaviours. Environmental protection for sustainable development can be gained through public awareness and mobilisation taught from schools and families, also non-governmental organisations and social media to reach a bigger audience. Because of this valuable context, Enhancing Environmental Education Through Nature-Based Solutions is mainly suitable for teachers in order to be able to create Environmental Education according to the models and solutions explained in this book. For this review, we aim to discuss Environmental Education, its teaching and how this book might be useful to environmental educators. Besides, through this review we will also point out the strengths and weaknesses that may be helpful for those who want to study this book.

The first part of this book has four chapters clarifying the definition and scope of Environmental Education. This section highlights opportunities for Environmental Education through providing students with knowledge about the components of nature, including both biotic and abiotic components. Thus, the negative environmental impact from humans can be reduced by understanding information about the environment. What makes this part interesting is how it provides the pedagogical interventions to be used in Environmental Education, including teaching geoethic to re-educate the relationship between humans and more-than-humans, and changing the economic growth paradigm to avoid ecosystem damage. This is in line with the belief that Environmental Education should adopt the Earth systems education approach because the outdoor learning environment has a big role in creating personal relevance so that it can help students to develop their high-order thinking skills. However, the authors in this section also share some difficulties and strategies to implement the abiotic component of Environmental Education,

© The Author(s), 2023. Published by Cambridge University Press on behalf of Australian Association for Environmental Education. This is an Open Access article, distributed under the terms of the Creative Commons Attribution licence (http://creativecommons.org/licenses/by/4.0), which permits unrestricted re-use, distribution and reproduction, provided the original article is properly cited.

especially with several activities developed in Spain. Therefore, it is necessary to improve both the current state of Earth science and geological material in compulsory education and the way that different educational stages are organised.

The second part of this book investigates the learning of Environmental Education, which is the primary focus of improving sustainable development. In this section, this book suggests the design of Environmental Education implementation integrated through the curriculum plans, which are obtained through studies conducted in Latin America, where the curriculum itself can offer solutions to environmental problems and students' needs after they graduate. In order to make the reader keep on the track, the authors emphasise that the curriculum needs to be structured so that it can influence students' behaviour to care for the environment and impact future opportunities. The appealing aspect about this section is it not only highlights the information from one point of view but also gives a wider perspective about several researchers conducting studies in different countries. What fascinates us even more about this section is how it can transport us to see the Cabo Verde (CV), a small island developing state with many challenges in the natural environment, and depict what kind of environmental challenges this island has faced. In order to survive and overcome the problems existing on that island, the authors point out the NbS to be applied even though they require a more sustained and coordinated effort. In addition, through the Interpretive Pedestrian Route in Portugal as a project of awareness, this book successfully shows that public awareness of geology strengthens skills in environmental protection, which is important in Environmental Education.

Meanwhile, the third part explains how social engagement can improve nature-based Environmental Education. Specifically, learning about the potential for biodiversity that can be contextually integrated by nature-based learning which aims to instil a sense of responsibility in students for the surrounding ecosystem. The authors suggest that a shared awareness is needed, regardless of whether they are teachers or students. Furthermore, social engagement in nature-based environmental improvement is detailed from a study conducted with Figueira da Foz residents through communication and consultation activities that stimulate interaction and proactive behaviour. Furthermore, in efforts to improve the nature-based environment, this part also provides a clear description of how successful the Portuguese government is in utilising the potential of their GeoPark so that it can be useful in nature-based education programmes. The programme focuses on sustainable geoscience education, which is considered quite effective in promoting local natural and cultural heritage, conservation, and sustainable development in the Portuguese territory.

In the last part of this book, the authors discuss how all challenges related to nature and sustainability can be overcomed by implementing NbS in several important sectors. The authors in this section provide various examples of good NbS practices from several countries and how they turn challenges into opportunities. Some of the interesting issues discussed in this book regarding the NbS strategy are how the authors describe in detail the strong economic potential of the green market in several countries such as Australia, Germany and the Czech Republic in recent years. Furthermore, it also provides some of the benefits of implementing NbS, such as how NbS can solve challenges related to sanitation in Portugal by utilising NbS principles in education campaigns and sustainable practices. Environment-based education is a strategy that is in line with the principles of NbS, and this fact is supported by the evidence presented in this book which gives an example of how the role of education can have a significant effect on reducing the amount of carbon emissions in the energy sector in Europe so that it can be used to promote control of water pollution. With the NbS and Environmental Education ideas discussed in this section, conventional education has a good chance of shifting away from its current paradigm and towards a more critical systemic thinking that will foster environmental citizenship as a foundation for environmental, socio-economic, political, economic and social change.

Overall, this book succeeds in synthesising diverse information from various perspectives. The principles, regulations and practices of a more sustainable environment are encouraged in this

book and can support changes in both individual and societal behaviour. Besides, this book not only gives the fundamentals on why Environmental Education for sustainable development is important to be conducted but also offers the methods to develop it. In addition, it succeeds in suggesting supporting factors for the achievement of environment-based learning. The presentation of data and various case studies in this book can serve as a basis for education policy-makers in various countries in perfecting and synchronising Environmental Education practices and NbS to address various challenges of natural change and sustainability issues. Unfortunately, this book does not explain in depth about the design and systematisation of an environment-based curriculum that can be implemented in certain conditions and situations.

Author Biographies

Ahbi Mahdianing Rum is a master's student in Mathematics Education in the Faculty of Mathematics and Science, Universitas Pendidikan Indonesia, Indonesia. Her research interests include mathematical literacy, development of media learning, learning and interaction, and education for sustainable development.

Amsal Alhayat is a master's student in Curriculum Development in the Faculty of Educational Science at Universitas Pendidikan Indonesia, Indonesia. His research interests include evaluation curriculum, STEM-based learning curriculum, chemistry learning and sustainable education.

Alfin Anwar is a master's student in Curriculum Development in the Faculty of Educational Sciences at Universitas Pendidikan Indonesia, Indonesia. His research interests include curriculum studies, environmental education and education for sustainable development.

Cite this article: Rum, A.M., Alhayat, A., & Anwar, A. (2023). A review of "*Enhancing Environmental Education Through Nature-based Solutions*". *Australian Journal of Environmental Education.* https://doi.org/10.1017/aee.2023.16
