# Supplementary material for: Dietary fibre intake in the adult Swiss population: a comprehensive analysis of timing and sources
Source: J Nutr Sci. 2025 Mar 24;14:e27. doi: 10.1017/jns.2025.6 (PMC11950702; doi:10.1017/jns.2025.6)
Supplement: von Blumenthal et al. supplementary material 2 — von Blumenthal et al. supplementary material [file S2048679025000060sup002.zip › AEE-2400022-Article (1).pdf]

QUERIES

AQ1: The distinction between surnames can be ambiguous, therefore to ensure accurate tagging for indexing purposes online (e.g. for PubMed entries), please check that the highlighted surnames have been correctly identified, that all names are in the correct order and spelt correctly.

AQ2: Please check that affiliations of all the authors and the corresponding author details are correctly set.

AQ3: This reference is cited in the body of the text but does not have a corresponding reference in the reference list. Please supply the complete reference for Oxford Economics, 2019.

AQ4: This reference is cited in the body of the text but does not have a corresponding reference in the reference list. Please supply the complete reference for Colucci-Gray & Burnard, 2019.

AQ5: This reference is cited in the body of the text but does not have a corresponding reference in the reference list. Please supply the complete reference for Harris & DeBruin, 2018.

AQ6: This reference is cited in the body of the text but does not have a corresponding reference in the reference list. Please supply the complete reference for Malone et al., 2020.

AQ7: This reference is cited in the body of the text but does not have a corresponding reference in the reference list. Please supply the complete reference for Haraway, 2016.

AQ8: Please check sentence "In so doing, we . . . " for clarity.

AQ9: The sentence "This was especially apparent . . . " seems to just a repetition of the previous sentence, hence deleted. Please check if okay.

AQ10: This reference is cited in the body of the text but does not have a corresponding reference in the reference list. Please supply the complete reference for Brand & Triplett, 2012.

AQ11: This reference is cited in the body of the text but does not have a corresponding reference in the reference list. Please supply the complete reference for Cunningham, 2018.

AQ12: This reference is cited in the body of the text but does not have a corresponding reference in the reference list. Please supply the complete reference for Hickey & Riddle, 2022.

AQ13: This reference is cited in the body of the text but does not have a corresponding reference in the reference list. Please supply the complete reference for Beattie, 1995.

AQ14: This reference is cited in the body of the text but does not have a corresponding reference in the reference list. Please supply the complete reference for Abbot, 2002.

AQ15: This reference is cited in the body of the text but does not have a corresponding reference in the reference list. Please supply the complete reference for Phillips, 2018.

AQ16: This reference is cited in the body of the text but does not have a corresponding reference in the reference list. Please supply the complete reference for Phillips et al., 2018.

AQ17: This reference is cited in the body of the text but does not have a corresponding reference in the reference list. Please supply the complete reference for Drake et al., 2021.

AQ18: This reference is cited in the body of the text but does not have a corresponding reference in the reference list. Please supply the complete reference for Haraway, 2016.

AQ19: This reference is cited in the body of the text but does not have a corresponding reference in the reference list. Please supply the complete reference for Burnard et al., 2021.

AQ20: This reference is cited in the body of the text but does not have a corresponding reference in the reference list. Please supply the complete reference for Haraway, 2016.

AQ21: This reference is cited in the body of the text but does not have a corresponding reference in the reference list. Please supply the complete reference for Savage, 2016.

AQ22: Please provide Ethical standard statement.

AQ23: Reference 'Manathunga (2009)' is incomplete. Please provide any of the relevant missing information: VOLUME.

Australian Journal of Environmental Education (2024), 1–16
doi:10.1017/aee.2024.22

CAMBRIDGE UNIVERSITY PRESS

ARTICLE

A Water[shed] Moment for Articulating a Professional Practice of Education Resource Creation

Abbey MacDonald and Kim Beasy AQ1

Faculty of Education, University of Tasmania, Launceston, Tasmania, Australia AQ2
Corresponding author: Abbey MacDonald; Email: abbey.macdonald@utas.edu.au

(Received 29 November 2023; revised 01 May 2024; accepted 01 May 2024)

Abstract
Teachers are grappling with increased pressure and expectations to facilitate transformative education experiences, the kinds of experiences that cultivate dispositions and skillsets essential for young peoples' preparedness to imagine and create sustainable futures. As expectations for teachers grow, so too do initiatives intended to assist their efforts, such as the advent of classroom-ready education resources. The rise of educational resources gives cause for closer examination of how they are developed, particularly with respect to the ways they situate content in the deployment of curricular, methodological and pedagogical concepts. This article presents a practice and process of education resource creation using multi-modal content that entangles global education and conservation agendas. Through the mediating lens of UNESCO's pillars of education, a critical discussion of the utility of these for enabling and inhibiting the articulation of a professional practice for education resource creation is offered. With the imperative for sustainability-focused education and prevalence of education resources being produced to support this, we scrutinise the importance of demystifying the professional practice of education resource creation. In doing so, we point to insights that become available when the curricular, pedagogical and methodological concepts informing education resource creation are made transparent and accessible.

Keywords: Education resources; education resource creation; interdisciplinarity; sustainable futures; teacher education

Introduction
Research indicates that having access to quality educational resources plays a critical role in helping teachers rise to the challenge of facilitating authentic and integrous learning experiences, particularly during periods of educational reform (Glasnović Gracin & Jukić Matić, 2021). For this article, we consider an education resource to be any tool, material or entity that facilitates learning, teaching or educational activities (see Tuomi [2013] for a comprehensive discussion of Open Education Resources).

While transitioning towards sustainability-focused education is an international imperative (Beasy, Smith & Watson, 2023), such transition is impeded by ongoing reforms in education, which contribute to the perpetual reconfiguration of teachers' roles. For decades already, "educational reform in Australia has been a quagmire of political and educational agendas, with a myriad of known factors (of which change fatigue is a part) that have enhanced or hindered implementation" (Dilkes, Cunningham & Gray, 2014, p. 46). When we couple this with humanity having become a geophysical force capable of fundamentally altering planetary ecological systems (Rousell *et al.*, 2017; Steffen *et al.*, 2015), we can come to better appreciate how teachers are becoming increasingly enmeshed in wicked problems. In the context of discussion across this

© The Author(s), 2024. Published by Cambridge University Press on behalf of Australian Association for Environmental Education. This is an Open Access article, distributed under the terms of the Creative Commons Attribution licence (http://creativecommons.org/licenses/by/4.0/), which permits unrestricted re-use, distribution and reproduction, provided the original article is properly cited.

article, we consider wicked problems as those that comprise an evolving set of interlocking issues, constraints and possibilities (Conklin, 2003; Rittel & Webber, 1973).

Bleazby, Thornton, Burgh and Graham (2023) describe that despite the scientific consensus regarding anthropogenic climate change (Cook *et al.*, 2016), the issue continues to be socially and politically controversial (Mcpherson *et al.*, 2023), particularly with respect to how teachers' successfully and sensitively cultivate dispositions and skillsets essential for imagining and creating sustainable futures. This situation poses a dilemma for teachers, where they can risk being accused of indoctrination if they teach from the evidence base of climate change in a directive manner or criticised for adopting more impartial approaches that risk undermining key aims of climate education (Bleazby et al., 2023). For teachers, this combination of choice dilemma, reform fatigue and expectations to attend to compliance-driven agendas impact upon the core business of teaching and learning (Gavin, McGrath-Champam, Wilson, Fitzgerald & Stacey, 2021). This in turn, we posit, inhibits teachers' ability to meaningfully engage with international global imperatives concerned with planetary survival. As teachers grapple with mounting pressures to demonstrate accountability in their practice to multiple agendas, it comes as no surprise that they are increasingly turning to external education resources to help meet these (Silver, 2022). The rise of education resources unfolds in relation and parallel to this.

Education resources: Production, utility and opportunities

In developing this article, we initially surveyed relevant literature in order to better understand the prevalence and context of existing scholarship regarding education resource creation. Examination of three databases (Scopus, ERIC, Education Source) revealed a paucity of peer-reviewed research explicitly addressing K-12 education resource creation and its underlying professional practices. Narrowing our search, we looked for literature that might offer ancillary insights into the production of education resources, particularly examples that were speaking from or that might be perceived as adjacent to environmental education settings. Noteworthy examples included review of empirical studies on K-16 climate education (Bhattacharya *et al.*, 2021), investigation of the practices of curriculum curation (Dezuanni & Zagami, 2017; Mahon, 2016) and entanglement of pedagogical and learning possibilities (Jukes *et al.*, 2022). Further to this, we also identified works on curriculum and pedagogical concepts that were congruent with or that could be contextualised to climate change education settings (Brennan, 2022; Jukes & Reeves, 2020). While some examples of literature discuss teachers' increased use of resources (CooperGibson Research, 2018; Glasnović Gracin & Jukić Matić, 2021), we found limited literature specifically addressing the motivations and methods for education resource production.

Engagement with education resources offers a dual purpose; first, in providing tangible materials for everyday classroom practice (Usiskin, 2013), and second, they can inadvertently function as tools that foster and support professional development (Glasnović Gracin & Jukić Matić, 2021). For example, Cool.org, an Australian organisation, offers sustainability-focused, curriculum-integrated resources, with an independent study revealing their positive impact on teachers and students, enhancing confidence and skills in teaching environmental and social issues for 91% of surveyed teachers (Lonergan & Labour, 2020). More recently, Ngarrngga (2023) is developing resources made by educators for educators in collaboration with Indigenous knowledge experts, with the vision for all Australian students to have the opportunity to connect with Aboriginal and Torres Strait Islander knowledge systems, histories and cultures (Ngarrngga, 2023). With teachers' uptake of such resources and subsequent evaluations that will no doubt follow, it is fair to surmise that teachers' vicarious and direct engagement with corporate entities through resources and professional development will become increasingly commonplace (Andrée & Hansson, 2021).

Further to this, GLAM institutions (galleries, libraries, archives and museums) are actively contributing education resources and professional learning for teachers and tailored tours for school groups. GLAM settings provide means for cultural collections, natural history displays and

contemporary artworks to be curated into complex narratives about the people and places from whom these materials were produced (MacDonald *et al.*, 2024). These initiatives have rich potential to connect communities and provide complex yet accessible opportunities for learning (Baguley *et al.*, 2018). In addition to this, GLAM institutions are — albeit slowly and tenuously — becoming more transparent in acknowledging and accounting for the problematic means by which their collections and exhibited materials have been acquired (Rimmer & Taylor, 2023). Education resources created in and for GLAM settings are becoming more concerned with detailing processes that can help audiences become more aware of "the significance of representation and the power of symbols to carry meaning, to signal identity and to invoke social and cultural alignments" (Ruanglertbutr, 2014, p. 5). With research evidence pointing to teachers' increasing utilisation of GLAM institutions' websites and social media portals for education purposes (Oxford Economics, 2019), it is affirming to see the awareness of the need and efforts being made in these spaces to support teachers.

Teachers' production, engagement with and uptake of education resources

While we could point to manifold examples of initiatives working to develop quality education resources for teachers, it seems that teachers' readiness to seek out and uptake education resources can be impeded by factors such as reform fatigue, role recalibration and compliance priorities (Stacey, Gavin, Fitzgerald, McGrath-Champ & Wilson, 2023). Factors of accessibility, adaptability and currency, coupled with standardisation of curriculum, run parallel to the rise in the production of online resources that can reach a mass audience with shared needs (Silver, 2022). Keeping up with curriculum change and rolling reforms can weigh heavily on teachers' readiness to seek out and embrace change (Dilkes *et al.*, 2014), particularly in the context of seeking out and incorporating new education resources into their programmes. This is an important insight, as while it points to the utility of education resources, it is clear that usefulness alone will not resolve the conflating challenges teachers are working with to utilise them effectively.

In looking at the practices that underlie education resource creation and making these transparent, we can better understand how education resource creation engages with — for example — compliance agendas, accurate climate science, pro-environmental values and attitudes and civic actions necessary for addressing climate change (Bleazby *et al.*, 2023; Lehtonen, Salonen, Cantell & Riuttanen, 2018). This is where the potential lies for education resources and their means of production to be both demonstrative and educative in their deployment of relational and ecological approaches that work intra-actively (Barad, 2014) in fostering connections between curricular, methodological and pedagogical agents (Brooke, MacDonald, & Hunter 2024; Harris, 2016). Across this article, we work with Karen Barad's (2007) concept of intra-action, where we pay attention to the ways that curricular, methodological and pedagogical agents become co-constitutively entangled in our articulation of a professional practice for education resource creation.

Curricular, methodological and pedagogic considerations for education resource creation

As specialists in curriculum and pedagogy deployment within their respective education settings, teachers are well versed in fostering relationality between and in their enactment of curriculum and pedagogy; this can be conceived as their becoming "curricula-pedagogic" (Ball, 1990; Brennan, 2022; Brooke *et al.*, 2024). They also engage and work incisively with discipline-specific content knowledge and its associated methodologies and pedagogies in ways that can be described as "metho-pedagogic" (Gallagher *et al.*, 2022; Healy *et al.*, 2022; MacDonald *et al.*, 2022). In saying this, researchers and curriculum developers continue to grapple with how to best support teachers to traverse and draw together different disciplines in education settings (MacDonald, Hunter, Wise & Fraser 2019; Wise, MacDonald, Badham, Brown & Rankin, 2022). This is pertinent, given

that at the highest levels of governance, there is a recognition of the need to draw on multiple disciplinary perspectives to combat wicked problems such as those that inhibit the ambitions of sustainability (Lehtonen *et al.*, 2018).

In education settings, conceptual strategic reforms can occur rapidly, while their implementation on the ground happens more slowly. It is widely recognised that teachers continue to grapple with their enactment of evolving disciplinarities and derivative discipline acronyms, such as STEM and STEAM (Colucci-Gray & Burnard, 2019; Harris & DeBruin, 2018; Hunter, 2024; MacDonald *et al.*, 2019, 2020). Increasingly diverse concepts of disciplinarity are being touted as essential for engaging people in sustainability issues (Gavari-Starkie *et al.*, 2022), and each evolution of disciplinarity is highly iterative and often contextually contingent. Education resources created in GLAM settings often explicitly engage with discipline acronyms (Lawson *et al.*, 2018; Park & Cho, 2022; Wise *et al.*, 2022). They point to curricular, methodological and pedagogical considerations that invite disciplinary integration opportunities pursuable in and beyond GLAM settings.

Before we move into discussion of our professional practice of education resource creation, we start at the place and moment which gave cause for the resource explored in this article to be produced — this being Lake Pedder and the water[shed] project. While this article seeks to alight the why and how for demystifying the professional practice of education resource creation, it is appropriate to start at the impetus for the cause, need and purpose of the education resource creation being examined.

Lake Pedder and the water[shed] project

At the heart of the watershed project lies Lake Pedder, once a stunning feature of Tasmania's Southwest Wilderness area (OUTSIDE THE BOX/Earth Arts Rights, 2023). Designated as National Park in 1955, its protected status was revoked in 1967 for the Gordon hydroelectric power scheme development, sparking significant local and international protests (Restore Pedder, 2023). The flooding of Lake Pedder serves as a stark reminder of the fragility of conservation efforts and points to the importance of education in fostering essential conservation values required to prepare and inspire future leaders to protect, act upon and uphold global restoration goals.

The water[shed] project was conceived by OUTSIDE THE BOX/Earth Arts Rights and presented in collaboration with Bett Gallery, in Hobart, Tasmania, Australia, to support the Restore Pedder campaign. While it was designed around a physical art exhibition which was time limited, it maintains a digital presence (OUTSIDE THE BOX/Earth Arts Rights, 2023). The digital archive houses the deliverables of the water[shed] project, which included major creative outputs in the form of an art exhibition, featuring the works of 50 significant Australian and international artists. Accompanying this exhibition is a substantial book publication featuring critical commentary from significant Australian and Aboriginal art historians, curators and essayists.

The water[shed] education resource

A comprehensive education resource (MacDonald & Beasy, 2022) was produced in complement to these major creative outputs and made available as a printed booklet as well as digitally. Digital copies were made (and remain) available as a free download from the OUTSIDE THE BOX/Earth Arts Rights website (OUTSIDE THE BOX/Earth Arts Rights, 2023). In complement to the digital longevity of the water[shed] project, the education resource was designed for use both in and beyond the physical exhibition timeline, across the United Nations Decade on Ecosystem Restoration (2021–2030). In alignment with this global initiative, the resource seeks to inform classroom discourse pertaining to how we can teach and learn about the degradation of ecosystems and the intra-acting environmental and agential factors (Malone *et al.*, 2020) that permeate these.

The resource offers rich, evocative accounts in the form of assembled excerpts from artists and essayists' visual and textual imaginings of Lake Pedder. For example, water[shed] artist, Sue Lovegrove, describes in her *Mapping the invisible* (2021) water[shed] artist statement how "an invisible lake lying beneath the surface of the water is a compelling image to imagine" (Lovegrove, as cited in MacDonald & Beasy, 2022, p. 41). The conversation that ensues in *A tale of loss and hope* (MacDonald & Beasy, 2022, p. 45) between Julie Gough's water[shed] artwork *Determined* (as cited in MacDonald & Beasy, 2022, p. 34) and Greg Lehman's water[shed] essay prompts remembrance that "this story is not political or historical. It's a cultural reimagining . . . It is the same for the Lake. It is a story of coming of age, of ceremony, and deep connection and respect for Country and all of its citizens" (Lehman, as cited in MacDonald & Beasy, 2022, p. 34). These are just some of the excerpts from the water[shed] project that serve as a reminder that "the original lake is not forgotten. It lies quietly waiting, just 15 m beneath the dark, brooding body of water still officially gazetted as Lake Pedder" (water[shed], 2022, n.d).

The exhibition was shown over three weeks from 5 to 27 August 2022, coinciding with the 50th anniversary (1972) of Lake Pedder being subsumed into the flood waters of the Huon-Serpentine Impoundment in 1972. We encourage you to explore the multi-modal storying of the water[shed] exhibition materials as they are situated in the education resource and in relation to the professional practice of education resource creation offered here (Figure 1).

The water[shed] exhibition includes the work of artists who encounter the concept of landscape and Country from diverse cultural perspectives, including works from Aboriginal and Torres Strait Islander artists. "Landscape" is a term laden with European ideological connotations (Delphin & MacDonald, 2018), which substantially differ from the complex spiritual Aboriginal conceptualisations of a country being more than a physical place and a living entity with its own agency and spirit (Bawaka Country *et al.*, 2015; Langton, 2021; Moreton-Robinson, 2020). Research shows that non-Indigenous teachers often feel varying levels of uncertainty in teaching diverse cultural perspectives and histories appropriately and with integrity (Bishop, 2020; Bodkin-Andrews *et al.*, 2013; Ngarrngga, 2023b; Riley *et al.*, 2019). We carefully considered the opportunity before us to alight the importance of working with an informed awareness of these tensions. This led us to scrutinise our choice and decisions pertaining to discourse and vernacular adopted across the resource (Hogarth, 2017; MacDonald, 2019). The resource deliberately limits using the term "landscape" throughout to respect the diversity of cultural perspectives featured in the exhibition, the historical storying and the deep history of Lake Pedder.

The following section of the article speaks to the context in which the water[shed] education resource was created, the aims and ambitions of the project and the positionality of this authorship team. As the authors of this article are the authors of the water[shed] education resource, we move between third- and first-person stance, where "we" is adopted to indicate and entwine our parallel authorship roles.

Context and positionality

To understand the convergence of contextual backgrounds underpinning the process of interdisciplinary education resource creation described in this article, it is important to acquaint readers with the authorship teams' individual positionality and our professional contexts and how these permeate our entwinement of disciplinary narratives and subsequent meaning-making.

Abbey is a non-Indigenous Australian woman of Scottish ancestry (Clan Donald; Macdonalds of Sleat). Abbey grew up near Murita[1]/Port Sorell on the north-west coast of Lutruwita (Tasmania), unceded lands of the Palawa people. As an artist, teacher and volunteer, Abbey brings to all aspects of her work a strong personal focus on arts and interdisciplinary education advocacy,

[1]In palawa kani, the language of Tasmanian Aborigines (Tasmanian Aboriginal Centre, n.d) https://tacinc.com.au/pulingina-to-lutruwita-tasmania-place-names-map/

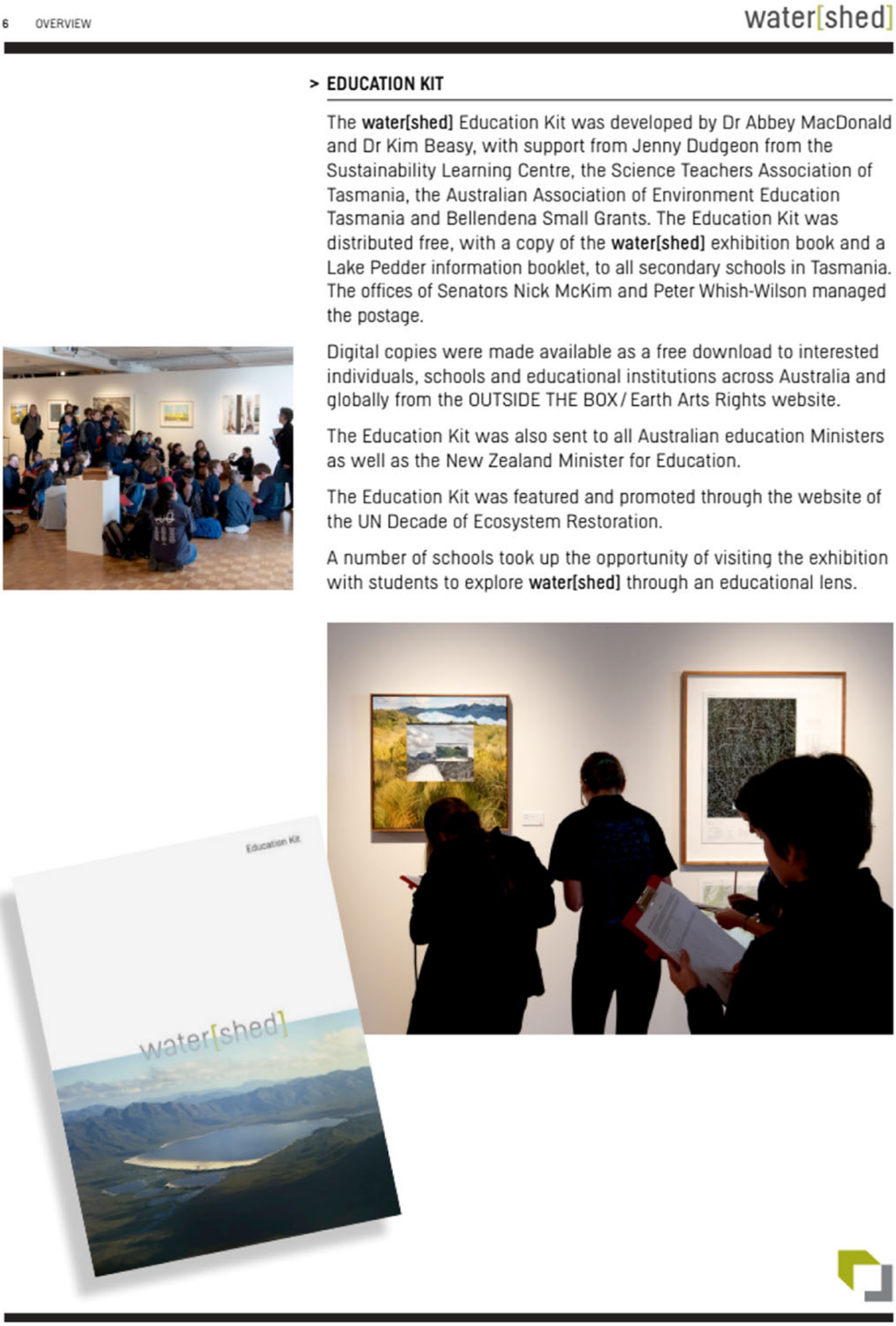

6 OVERVIEW water[shed]

> EDUCATION KIT

The **water[shed]** Education Kit was developed by Dr Abbey MacDonald and Dr Kim Beasy, with support from Jenny Dudgeon from the Sustainability Learning Centre, the Science Teachers Association of Tasmania, the Australian Association of Environment Education Tasmania and Bellendena Small Grants. The Education Kit was distributed free, with a copy of the **water[shed]** exhibition book and a Lake Pedder information booklet, to all secondary schools in Tasmania. The offices of Senators Nick McKim and Peter Whish-Wilson managed the postage.

Digital copies were made available as a free download to interested individuals, schools and educational institutions across Australia and globally from the OUTSIDE THE BOX / Earth Arts Rights website.

The Education Kit was also sent to all Australian education Ministers as well as the New Zealand Minister for Education.

The Education Kit was featured and promoted through the website of the UN Decade of Ecosystem Restoration.

A number of schools took up the opportunity of visiting the exhibition with students to explore **water[shed]** through an educational lens.

Figure 1. The water[shed] education resource, shared with permission from *OUTSIDE THE BOX/Earth Arts Rights*. Embedded images shared with permission from *Bett Gallery,* Hobart. https://outsidethebox.org.au/assets/projects/watershed-restore-pedder/Watershed_Education_Kit.pdf.

fostering teacher agency, community engagement and multi-stakeholder collaboration. Her research is used to inform the design, development and evaluation of content, curriculum and education resources for diverse education settings in school, museum, gallery and tertiary education contexts. She loves working with creative industries and philanthropic organisations looking to collaborate with education transformation stakeholders.

Kim is a non-Indigenous Australian woman with a convict and colonial settler heritage. While born on the lands of the Wamba Wamba, Latji Latji, Tatti Tatti, Waddi Waddi and Barapa Barapa peoples, her family moved to the lands of the Palawa people (Lutruwita) during her childhood. Here, Kim trained as a physical geographer and spent time knee-deep in Tasmania's salt marshes before arriving into social science ways of understanding the world. For the last decade, Kim's teaching and research work strongly features community connection and place-based inquiries centring on being of and for nature.

This authorship team lives in the Australian island state of Lutruwita (Tasmania). They share the parallel of growing up on this island in coastal towns and find kinship in these experiences of living and learning in proximity to waterways. They know there is still much to learn, unlearn and learn anew (McLeod *et al.*, 2020). They take this into their work together in the School of Education at the University of Tasmania.

Story and inquiry lines

As the authors of this journal article and the water[shed] education resource, we share a background of conducting qualitative research with teachers and students in education contexts, part of which involves their using creative, arts-based and storied methods for generating and analysing visual, spoken and written texts. As two teacher educators working in an Australian context, we acknowledge that our approach to doing this is invariably informed by our familiarity with the Australian Curriculum (Australian Curriculum, Assessment and Reporting Authority [ACARA] n.d). In addition to this, we have sought to leverage our familiarity with this particular curriculum to identify synergies with globally resonant education and conservation agendas, including

- The UNESCO Principles of the Decade of Restoration (2021–2030)
- United Nations Sustainable Development Goals (2016)
- Four [reworking] Pillars of Education to Sustain the Commons (Sobe, 2021)

In exploring the intra-activity of these, we networked materials of practice and curricular, pedagogical and methodological agents, enacting these into what became our professional practice of education resource creation. These permeate the water[shed] education resource, and we remind readers of the invitation and encouragement to explore the resource concurrently with this article.

The development of the water[shed] education resource draws from our relational paralleling and connecting with a global breadth and depth of education curriculum, pedagogy and policy agendas. While the resource identifies connections to global education agendas and specific curriculum, subject or learning areas, we found particular resonance with Sobe's (2021) reworking of UNESCO's four pillars of education as a globally accessible interpolating device for helping teachers find and leverage multifaceted learning opportunities and legacies of Lake Pedder, as captured in the water[shed] project.

Through these four pillars of education, we co-created meaning from the water[shed] materials using visual-textual assemblages for interdisciplinary inquiry. Drawing from Duke's (2010) insights on curatorial practice, we aimed to facilitate nuanced learning encounters rather than prescribe specific lessons. Our resource invites teachers to adapt our assemblages to their contexts,

emphasising that there's no singular interpretation. We have no say, nor do we seek to claim control over the direction and emphasis of classroom inquiries that can be pursued; we trust in teachers' agency, their responsibility and response-ability (Bozalek, Bayat, Gachago, Motala & Mitchell, 2018) to do the work required to adapt and contextualise as appropriate.

There is an absence of reference to specific curriculum frameworks in the anchorage of this education resource. Given the potential for global appeal of the water[shed] project, it is important that its' education resource resonate with broad education agendas. In seeking to augment globally attuned entry points for classroom inquiry, we sought to emphasise what we felt were prime opportunities for inter and transdisciplinary inquiry. In mapping to global education and conservation agendas, we therefore sidestep approaches to curriculum mapping that can be static and overlook metho-pedagogical considerations pertaining to the role of place in the nexus spaces between curriculum/pedagogy, teaching/learning and people/place.

Mapping education resources to any particular curricular framework involves more than simply specifying links between content and context. When curriculum interpretation and enactment is seen as an ongoing process teachers actively engage in, contribute to and drive, we affirm their capacity to maintain the openness and fluidity that is essential for embracing new ideas and the practices inherent to realising these ideas (MacDonald & Beasy, 2022). The storied assemblages of the water[shed] education resource are offered as connectible and open to further modification, where teachers and students can use these as impetus entry points for their own personally situated interdisciplinary inquiries.

Methodology

Lisa Grocott's (2022) metho-pedagogical "playdate" approach was used to facilitate our working together to craft the education resource. Embracing the playdate's principles of surrendering, contesting and iterating ideas, our collaborative process acknowledged the inherent unpredictability and multiplicity that comes with deconstructive/reconstructive thinking in a trusting and playful space (Grocott, 2022, p. 179). Our collective aim, manifested through two-hour weekly get-togethers over three months, mirrored the playdate's commitment to improvisation, communal belonging and the establishment of a social encounter that resonated with the diversity of our perspectives. We embraced messy tabletop mind-mapping alongside working synchronously in shared documents (Google Docs) to converge our parallel play experience.

Working with the water[shed] exhibition materials, curricular-metho-pedagogic agents and our parallel wisdoms brought to the project enabled the deconstruction of individual authorial voices (Grocott, 2022, p. 179) and curation of these into the storylines described across the education resource. We approached this work with a deep sense of responsibility and response-ability (Bozalek *et al*., 2018; Haraway, 2016) to acknowledge our own teacher/teacher educator selves and teachers as agents in their own right who are capable of shaping their own assemblages of learning and inquiry. Active listening combined with a sense of curiosity and provocation enabled us to draw confluence between disciplinary differences and find new ways of encouraging interconnected meaning-making with curricula-metho-pedagogic agents. We also utilised the key tenets of the playdate framework to facilitate our address of article revisions.

In developing the water[shed] education resource, we engaged in individual and shared processes of reflection, learning and listening for ways to make sense and meaning of an increasingly fast-changing world. Our own wanderings and wonderings lead us to discover that UNESCO's four pillars of education were reviewed and updated in 2021. These provided a globally accessible mediating device for articulating learning about the legacies of Lake Pedder, as captured in the water[shed] project.

Drawing from the UNESCO four pillars of education to sustain the commons

In reorienting the four pillars of education towards building capacity for the common good and action, the updated pillars offer a framework for charting lines of inquiry into and through the collective challenges we face today and into the decades to come (Sobe, 2021).

The four original and reoriented pillars are

learning to know > learning to study, inquire and co-construct together

learning to do > learning to collectively mobilise

learning to live together > learning to live in a common world

learning to be > learning to attend and care

Below, we detail our playful interpretations of working *with* and *through* the reoriented pillars. In this, we concurrently unfold and interrogate our working *through* the pillars to create an education resource. In so doing, we reveal the potential of these pillars google

Learning to study, inquire and co-construct together

In learning to study, inquire and co-construct together, we considered the intra-active possibilities of working with a global breadth and depth of education curriculum, pedagogy and policy agendas and their inherent agents (Barad, 2007). We came together as two teacher educators from different disciplinary backgrounds to share and make meaning from the suite of creative, textual materials that the water[shed] project offered. With its explicit focus on environmental ethics, multispecies, culture and place of Lake Pedder, we set about considering different ways of knowing (Butler & Sinclair, 2020; Rousell, 2020) across the water[shed] artists' and writers' contributions. This saw us considering diverse perspectives, including (but not limited to) disciplinary, cultural, environmental, socio-economic and political to make meaning.

Considering the evolving curriculum landscape and the water[shed] project's alignment with the UN Decade of Restoration (2021–2031), we carefully crafted a resource to foster transformative learning (UNESCO, 2021) beyond specific curriculum cycles. In Australia, curriculum typically undergoes review every six years (ACARA, 2023), but teachers' readiness to adopt new resources can be hindered by factors like reform fatigue (Stacey *et al.*, 2023). Thus, we sought to story global education and conservation agendas in ways that engaged agents of pedagogy and curriculum intra-actively, such as but not limited to curriculum and pedagogical content and discipline knowledge.

We set about identifying key themes, curiosities and points of interest that emerged via our engagement with multiplicity and diverse agents of cultural and disciplinary ways of knowing, being and doing (MacDonald & Beasy, 2022; Warren, Vossoughi, Rosebery, Bang & Taylor, 2020) across the water[shed] exhibition materials. We observed the water[shed] artists' statements and catalogue essays adopting discourses akin to working across and between different disciplines (i.e. cross and interdisciplinarity), rather than beyond them (transdisciplinarity). This was especially apparent with respect to discourse of working relationally with matter and agents of art, science and geography. We shared our formative interpretations along the way with contributors and supporters of the water[shed] project, intra-actively drawing their feedback and affirmations back into our own becoming ecological process (Brooke *et al.*, 2024) for education resource creation.

In our learning to co-construct this education resource, we actively took carriage of the labour required to engage meaningfully and relationally with global and local curriculum, pedagogy and

policy agendas, knowing that such deep engagement takes time and space that teachers have increasingly less capacity for (Stacey *et al.*, 2023). By actively attending to this learning together, interdisciplinary meaning-making and question creation, we co-constructed a series of storied assemblages — described throughout the resource as "tales." The notion of tales is a nod to the storying that we made with the water[shed] materials. Rather than specifying what teachers could do in response, we described the intent of the storied assemblages as a catalyst, impetus and provocation for classroom inquiry. In doing this, we model outcomes of our own relational engagement with and meaning made of water[shed] while concurrently recognising the deep expertise that teachers' possess and bring to their contextualisation and working with education resources. It was this process of learning to study, inquire and co-construct together that informed our storying of the "tales" that thread across the water[shed] resource.

Learning to collectively mobilise

Teachers and students exhibit remarkable resilience, creativity and adaptability in navigating environmental, social and political challenges (UNESCO, 2021), contributing actively to collective efforts despite systemic obstacles. We too found cause to support efforts and contribute to this collective mobilisation in our education resource creation.

The necessity for education and industry stakeholders to pool resources for sustainable education initiatives is widely acknowledged (Beasy *et al.*, 2023; Bleazby *et al.*, 2023). When learning to collectively mobilise, we discovered being clear and sincere in the communication of aspirations, agendas and goal setting was important to attend to at the outset. Doing so helped us to build rapport with key stakeholders involved in the creation of visual and textual materials for the water[shed] exhibition (artists, writers, environmental and social change organisations, curators, designers, teachers),and to create an education resource that met their expectations. When education resources acknowledge the deep expertise of teachers, they provide means for teachers to contribute to the call to collectively mobilise. In our own learning to collectively mobilise, we too felt compelled to contribute to the significant global imperative of sustainability and living in support of the common good (Sobe, 2021).

In our own learning to collectively mobilise, we came to appreciate how multi-stakeholder and multi -disciplinary collaboration can be a complex endeavour to mobilise, particularly when pursued in education settings. We recognised that drawing together disciplinary and other ways of knowing and doing indicative of different curriculum learning areas and subjects can facilitate greater conceptual understanding in students than learning content from each subject in isolation (Brand & Triplett, 2012). To enable this, we attuned to the interactions and flows between agents (such as, but not limited to, curriculum, methodology and pedagogy) in ways that sought to show rather than tell in our storying. The water[shed] artist statements and catalogue essays detail the mobilisation of methodological approaches and philosophical stances that permeate the cultural and visual art practices featured in the exhibition. From these, we wove curricular, methodological and pedagogical considerations into our education resource storying in ways that integrated disciplines, issues, ideas, problems and possibilities presented in the water[shed] exhibition. In articulating overarching storylines of complex interrelated themes that form the basis of concern and radical hope of water[shed], we sought to create space then for teachers and students to consider how they might mobilise their capacities for collective action in the meaning they make from their own and others' lived experiences.

Learning to live in a common world

The themes we identified emerged by entwining diverse perspectives to articulate synergies between different ways of knowing. Increased awareness of these can lead to shifts in responses to questions of multispecies justice amid climate change and mass extinction common to us all

(Rousell, 2020; United Nations, 2021). In looking into, across and between the artworks and artist statements, we noticed explicit engagement and working with agents of interdisciplinarity, such as people, methods and tools and art, science and geographically oriented matter and materials.

The convergence of different disciplines in education settings serves various functions and dysfunctions. For example, education research continues to grapple with a common and enduring curiosity to define intra, multi, cross, inter, trans and further prefixes for disciplinarity (Cunningham, 2018; MacDonald *et al.*, 2019). These are constantly being reimagined and renegotiated, particularly in onto-epistemological and axiological scholarship in, from and for education settings broadly (Mainardes, 2022; Whatman *et al.*, 2023). However, there can be a lag in teachers' uptake of new initiatives into curriculum deployment (MacDonald *et al.*, 2019). The entrenched disciplinary siloing in Australian schooling, especially in secondary education, hampers interdisciplinary enactments. Standardised testing pressures often deter teachers from taking risks and embracing curiosity (MacDonald *et al.*, 2019). To this end, we are aware of the affordances that education resource creation enables for risk-taking and experimentation that might not be common for all.

Recognising interdisciplinary practices in the water[shed] materials, we positioned our education resource creation and subsequent discussion within the discourse of interdisciplinarity. While we appreciate and are open to the rich potential of transformation and transdisciplinarity, there is adequate cause to be mindful of the work that needs to precede this and that creates the foundation upon which decolonisation of disciplines (Manathunga, 2009; Warren *et al.*, 2020) and transdisciplinarity can flourish. While the water[shed] education resource can be adapted to accommodate a range of disciplinary aspirations, we found common ground in the decision to think, act and co-create the resource out of the common liminal spaces between and across diverse disciplinary cultures. We sought to work with the same onto-epistemic openness being asked of teachers and industry collaborators as agentive thinkers who actively participate in and contribute to reimaginings of disciplinarity discourse (Warren *et al.*, 2020).

Learning to attend and care

Education resource developers must keep across evolving priorities and the factors influencing teachers' energy and capacities to engage. Sustainability education agendas should balance aspirations for authentic experiences with awareness of external pressures affecting teaching and learning (Gavin *et al.*, 2021). Depending on how resources balance these considerations, they can either enable or hinder teachers' ability to become ecological in their relational curriculum and pedagogy enactment (Hickey & Riddle, 2022).

In attending to this, we took care with and carriage of the disciplinary identity, skills and knowledge brought to our encountering of the water[shed] materials and were mindful of the opportunity education resource creation affords for learning and making meaning with different disciplinary discourses and practices (Manathunga, 2009). To avoid inadvertently further contributing to teachers' reform fatigue, shifts in the vernacular of disciplinarity and the acronyms intended to encourage interaction and integration were carefully considered. This is our commitment to working with a relational and ecological approach that calls for development and deployment of pedagogical practices that leverage both this *and* that, rather than this *or* that (Brooke *et al.*, 2024). While the themes of our "tales" may at first appear polar or binary on the surface, it is in the space between that we broaden and deepen understandings of our own lived experience through storying. In this respect, our attending to care diffracts collaborative, qualitative approaches where intra-acting storylines reverberate careful meanings of teaching, learning and professional interactions (Beattie, 1995).

In our attentive approach, we delved deeply into relational ontologies and pedagogies of place, during which we came to better appreciate how pedagogies of place are central to Indigenous ways of living, learning and knowing (Bawaka Country *et al.*, 2015). Stories are intrinsic to human

expression, shaping our understanding of the world (Abbot, 2002). Aboriginal and Torres Strait Islander cultures have long embraced embodied storytelling (Bunda and Phillips, 2018), predating Western narrative concepts (Phillips *et al.*, 2018). When storying with this in mind, we carefully consider, negotiate and navigate what works and, subsequently, find ourselves positioned to better understand who and what our knowledge creation can be in service of (Drake *et al.*, 2021). The process of generating storied assemblages of the water[shed] visual and textual materials allowed us to speculatively wonder and search for inclusive and non-alienating ways and means to story care for people and place.

Relationality enabled with and through the UNESCO four pillars of education

Barad (2007, p. 170) reminds us that "matter's dynamism is generative . . . in the sense of bringing forth new worlds, of engaging in an ongoing reconfiguring of the world." Those that produce education resources have the responsibility and response-ability to critically engage with the motives, the context and the broader curricular-metho-pedagogical agents that entwine in the making and reconfiguring of matter — to honour the intra-active and co-constitutive qualities of curation (Barad, 2007). It was our sense of *response-ability* that we sought to "attend and care" with and for the teachers who are entangled inseparably from developing education resources. Understanding and enacting a "collective knowing and doing, an ecology of practices through our capacity to respond" (Haraway, 2016, p. 34) led us to situating the assembled stories in the resource in interdisciplinary spaces.

The UNESCO pillars *acted* and *intra-acted* with us too, directing and challenging us to step outside of ourselves and be conscious of the ecosystem in which the education resource was seeking to inhabit. When we came together to create teachable moments with the visual and textual water[shed] materials, we found that diffractive patterns of discussion and sharing were generated with the undulations in our approaches. These emerged necessarily and concurrently as resonance and dissonance to ideas explored through the process (Barad, 2007). In working diffractively in the process and practice of education resource creation, we can and should work with this *and* that. We are not only working with curricular agents; we are working with methodological methods and pedagogical tools for meaning-making in disciplinary subject and in the human subject (Burnard *et al.*, 2021). Being open to sitting in the discomfort (Haraway, 2016) of not knowing or not fully grasping was part of our navigation of interdisciplinary terrain.

As the world increasingly becomes defined by precarity as a consequence of environmental, social and economic crises, we must find ways to support and embrace approaches to and for creating and engaging with the shifting imperatives of education. We recognise the indispensable role that education — and education resources — can have in empowering teachers and students and nurturing future leaders to actively pursue and attain global restoration goals and contribute towards sustainable life. Through engagement with the pillars, we came together to draw insights through one another as we attended and responded to the details and specificities of relations into a complex web (Barad, 2007; UNESCO, 2021). We did this not only with the thoughts and perspectives of each other but in relation to and with the ecology of the ecosystem we were immersed in for education resource creation.

Teachers continue to navigate shifting policy priorities — often politically motivated and amidst reform fatigue (Dilkes *et al.*, 2014; Savage, 2016; Stacey *et al.*, 2023). Attending to ontological and epistemological shifts requires time and space for grappling and sense-making. In our entanglements with the pillars, what emerged was a recognition that teachers and students are being invited to "collectively mobilise," which demands for us as entwined educators/education resource creators to support and enable this in ways that properly account for their lived experience and situational complexities. As response-able agents, the creation of the resource was sensitive to the intellectual demands and tensions that must be traversed (Barad, 2007). We made the decision to be responsive to the contextuality of the water[shed] artists, essayists and the

teachers we seek to engage and support and attune our storying to interdisciplinary opportunities and curiosities.

In assembling stories, we recognised and sought to value teachers' agentic capacity to respond with their own curiosities that may be kindled through engaging with the education resource. And so, rather than providing templates or bounded products for teachers and students to "complete," we offered our own curricular-metho-pedagogically informed professional practice of education resource creation.

At the same time, the pillars guided us towards undertaking a dynamic process through expressed values of "co-construction, collective mobilisation and attending and caring." In working through UNESCO's four pillars of education (2021) with the intra-active nature of materiality, we attended to relationships, seeking out and reverberating responsibly and response-ably feedback from the Science Teachers Association, water[shed] artists, essayists, curators, Aboriginal Education Services, artists and academics. We did so with an awareness of the imperative for relational engagement with the water[shed] knowledge makers and holders (Australian Institute of Aboriginal and Torres Strait Islander Studies [AIATSIS], 2022). This iterative process of drawing in and with relationality is a testament to our genuine readiness to engage with, hear and better understand diverse perspectives, histories and possibilities for the future of Lake Pedder.

The campaign to restore Lake Pedder is a powerful symbol of hope in increasingly troubling times.

The plan to restore Lake Pedder is courageous and visionary.

Education in all forms, across all fronts, has its clarion call, a watershed moment to contribute.

As a gesture of our commitment to contribute to the above, this article offers a rationale and practical approach for demystifying the professional practices that underlie education resource creation. Professionals engaged in the practice of education resource creation can work curatorially in their assemblage of education and conservation agendas and curricular-metho-pedagogical agents to create constellations of ecological perspectives and educative opportunities (MacDonald *et al*., 2024). The four reworked pillars of education (Sobe, 2021) can help mediate intra-activity between and guide curation of curricula, methodological and pedagogical agents in education resource creation. In making the professional practice of education resource creation transparent, we hope to make it easier for teachers to identify the positional motivations (who, why) and methods (what, how) that underpin the production of education resources they might consider utilising in their classrooms.

Acknowledgements. The authors would like to thank OUTSIDE THE BOX/Earth Arts Rights, the Education Sustainability Learning Centre (Department for Education, Children and Young People), the Australian Association of Environmental Education Tasmania and the Science Teachers Association of Tasmania for generously supporting the production of the water[shed] Education Kit. We also acknowledge and give thanks for the ongoing advocacy efforts of the Restore Lake Pedder campaign.

Financial support. This research received no specific grant from any funding agency, commercial or not-for-profit sectors.

Competing interests. The authors are reporting on their process of creating the water[shed] education resource. We are cognisant of the potential this creates to speak favourably in want of promoting the resource. Concurrently, as the people responsible for the resources' creation, we are best placed to speak to the practice and experience of producing it. To accommodate these co-potentialities (working with *this and that*), we reflected carefully upon the intra-activity of our personal/professional practices and engagement with the agents explored across the article. At the time of writing this article, Author 1 is seconded to the role of Senior Academic Fellow (Indigenous Exhibition Learning) at the University of Tasmania, working AQ22 directly with Ngarrngga. Ngarrngga is noted as one of the education resource examples in the front matter of this article.

References

Andrée, M., & Hansson, L. (2021). Industry, science education, and teacher agency: A discourse analysis of teachers' evaluations of industry-produced teaching resources. *Science Education*, *105*(2), 353–383.

Australian Curriculum Assessment and Reporting Authority [ACARA] (2023). Review of the Australian curriculum. https://acara.edu.au/curriculum/curriculum-review

Australian Curriculum Assessment and Reporting Authority [**ACARA**] (n.d.). The Australian Curriculum Version 9.0. https://v9.australiancurriculum.edu.au/

Australian Institute of Aboriginal and Torres Strait Islander Studies [**AIATSIS**] (2022). AIATSIS Guide to evaluating and selecting education resources. https://aiatsis.gov.au/education/guide-evaluating-and-selecting-education-resources

Baguley, M., MacDonald, A., & Jackett, A. (2018). Creative leadership and the Hadley's Art Prize Hobart (HAPH). *Australian Art Education*, *39*(2), 288–306.

Ball, S. (1990). *Politics and policy making in education: Explorations in policy sociology*. Routledge.

Barad, K. (2007). *Meeting the universe halfway: Quantum physics and the entanglement of matter and meaning*. Duke University Press.

Barad, K. (2014). Invertebrate visions: Diffractions of the brittlestar. In E. Kirksey (Eds.), *The multispecies salon* (pp. 221–241). Duke University Press.

Beasy, K., Smith, C., & Watson, J.E. (2023). *Education and the UN sustainable development goals: Praxis within and beyond the classroom*. Springer.

Bhattacharya, D., Carroll Steward, K., & Forbes, C.T. (2021). Empirical research on K-16 climate education: A systematic review of the literature. *Journal of Geoscience Education*, *69*(3), 223–247.

Bishop, M. (2020). "I spoke about Dreamtime - I ticked a box": Teachers say they lack confidence to teach indigenous perspectives. *Science Education News*, *69*(2), 73–74.

Bleazby, J., Thornton, S., Burgh, G., & Graham, M. (2023). Responding to climate change 'controversy' in schools: Philosophy for children, place-responsive pedagogies & critical indigenous pedagogy. *Educational Philosophy and Theory*, *55*(10), 1096–1108.

Bodkin-Andrews, G.H., Denson, N., & Bansel, P. (2013). Teacher racism, academic self-concept, and multiculturation: Investigating adaptive and maladaptive relations with academic disengagement and self-sabotage for Indigenous and non-Indigenous Australian students. *Australian Psychologist*, *48*(3), 226–237.

Bozalek, V., Bayat, A., Gachago, D., Motala, S., & Mitchell, V. (2018). A pedagogy of response-ability. In *Socially just pedagogies in higher education: Critical posthumanist and new feminist materialist perspectives*, 97–112.

Brennan, M. (2022). Teachers and students as researchers: Rebuilding curriculum inquiry for the future. *Curriculum Perspectives*, *42*(1), 85–89.

Brooke, S., MacDonald., A., & Hunter, M.A. (2024). *Becoming ecological: The contribution of collaborative a/r/tography to generalist primary teachers' agency in arts education*. Qualitative Inquiry.

Butler, A., & Sinclair, K.A. (2020). Place matters: A critical review of place inquiry and spatial methods in education research. *Review of Research in Education*, *44*(1), 64–96.

Conklin, J. (2003). *Dialogue mapping: Building shared understanding of wicked problems*. Wiley.

Cook, J., Oreskes, N., Doran, P.T., Anderegg, W.R., Verheggen, B., Maibach, E.W., & Rice, K. (2016). Consensus on consensus: A synthesis of consensus estimates on human-caused global warming. *Environmental Research Letters*, *11*(4), 1–7.

CooperGibson Research. (2018). *Use and perceptions of curriculum support resources in schools. Research report - July 2018*. Social Science in Government.

Country, B., Wright, S., Suchet-Pearson, S., Lloyd, K., Burarrwanga, L., Ganambarr, R., Ganambarr-Stubbs, M., Ganambarr, B., Maymuru, D. (2015). Working with and learning from Country: Decentring human author-ity. *Cultural Geographies*, *22*(2), 269–283.

Delphin, T., & MacDonald, A. (2018). Sanitising landscapes: Implications for meaning making. *Journal of Artistic and Creative Education*, *12*(1), 6–20.

Dezuanni, M., & Zagami, J. (2017). Curating the curriculum with digital games. In C. Beavis (Eds.), *Serious play: Literacy, learning and digital games* (pp. 67–82). Routledge.

Dilkes, J., Cunningham, C., & Gray, J. (2014). The new Australian curriculum, teachers and change fatigue. *Australian Journal of Teacher Education*, *39*(11), 4.

Duke, L. (2010). The museum visit: It's an experience, not a lesson. *Curator: The Museum Journal*, *53*(3), 271–279.

Gallagher, K., Cardwell, N., Denichaud, D., & Valve, L. (2022). The ecology of global, collaborative ethnography: Metho-pedagogical moves in research on climate change with youth in pandemic times. *Ethnography and Education*, *17*(3), 259–274.

Gavari-Starkie, E., Espinosa-Gutiérrez, P.T., & Lucini-Baquero, C. (2022). Sustainability through STEM and STEAM Education Creating Links with the Land for the Improvement of the Rural World. *Land*, *11*(10), 1869.

Gavin, M., McGrath-Champam, S., Wilson, R., Fitzgerald, S., & Stacey, M. (2021). Teacher workload in Australia. In A.H. S.Riddle & D. Bright (Eds.), *New perspectives on education for democracy* (pp. 110–123). Routledge.

Glasnović Gracin, D., & Jukić Matić, L. (2021). Use of textbooks and other resources in curriculum reform. A longitudinal case study. *ZDM Mathematics Education*, *53*(6), 1373–1385.

Grocott, L. (2022). *Design for transformative learning : A practical approach to memory-making and perspective-shifting*. Routledge.

Harris, A. (2016). *Creativity, education and the arts*. Palgrave Macmillan.

Healy, S., Mayes, E., Flynn, A., & Edwards, A. (2022). Entering into sympogogies. *Cultural and Pedagogical Inquiry*, *14*(1), 166–188.

Hogarth, M. (2017). Speaking back to the deficit discourses: A theoretical and methodological approach. *The Australian Educational Researcher*, *44*(1), 21–34.

Hunter, J.L. (2024). Invitations, impact, and involvement: Tales from successful school-university partnerships in STEM and STEAM in Australian Schools. In *Creating, sustaining, and enhancing purposeful school-university partnerships: Building connections across diverse educational systems* (pp. 39–57). Springer Nature Singapore.

Jukes, S., & Reeves, Y. (2020). More-than-human stories: Experimental co-productions in outdoor environmental education pedagogy. *Environmental Education Research*, *26*(9-10), 1294–1312.

Jukes, S., Stewart, A., & Morse, M. (2022). Following lines in the landscape: Playing with a posthuman pedagogy in outdoor environmental education. *Australian Journal of Environmental Education*, *38*(3-4), 345–360.

Lane, J.L., Boggs, B.J., Chen, Z., & Torphy, K.T. (2019). Conceptualizing virtual instructional resource enactment in an era of greater centralization, specification of quality instructional practices, and proliferation of instructional resources. *Teachers College Record*, *121*(14), 1–36.

Langton, M. (2021). *Marcia Langton: Welcome to Country 2nd edition: Fully Revised & Expanded, A Travel Guide to Indigenous Australia*. Hardie Grant Publishing.

Lawson, C.A., Cook, M., Dorn, J., & Pariso, B. (2018). A STEAM-focused program to facilitate teacher engagement before, during, and after a fieldtrip visit to a children's museum. *Journal of Museum Education*, *43*(3), 236–244.

Lehtonen, A., Salonen, A., Cantell, H., & Riuttanen, L. (2018). A pedagogy of interconnectedness for encountering climate change as a wicked sustainability problem. *Journal of Cleaner Production*, *199*, 860–867.

Lonergan, C., & Labour, D. (2020). Cool Australia's social impact research report. Sydney. https://cool.org/about/impact.

MacDonald, A. (2019). Talking to and about teachers: Resisting deficit discourses. *Professional Educator*, *21*(2), 22–25.

MacDonald, A., & Beasy, K. (2022). Water[shed] education kit. https://outsidethebox.org.au/projects/watershed-restore-pedder/.

MacDonald, A., Coleman, K., Healy, S., & Diener, M. (2022). How does pedagogical slipperiness enable speculation in/for teacher professional learning?. In R.E. Ferdig, E. Baumgartner, R. Hartshorne, R. Kaplan-Rakowski & C. Mouza (Eds.), *A retrospective of teaching, technology, and teacher education during the COVID-19 pandemic* (pp. 45–49). Association for the Advancement of Computing in Education.

MacDonald, A., Hunter, J., Wise, K., & Fraser, S. (2019). STEM and STEAM and the spaces between: An overview of education agendas pertaining to 'disciplinarity' across three Australian states. *Journal of Research in STEM Education*, *5*(1), 75–92.

MacDonald, A., Wise, K., Tregloan, K., Fountain, W., Wallis, L., & Holmstrom, N. (2020). Designing STEAM education: Fostering relationality through design-led disruption. *International Journal of Art & Design Education*, *39*(1), 227–241.

MacDonald., A., Rees, A., Hogan, J., & Richardson, B. (2024). "Becoming Ecological" for nature conservation: Insights from two museums in the Island State of lutruwita/Tasmania, Australia. In A. Sinner, P. Osler & B. White (Eds.), *Propositions for Museum Education*. Intellect.

Mahon, K.L. (2016). Personalizing curriculum: Curation and creation. In *Handbook on personalized learning for states, districts, and schools* (pp. 117–130).

Mainardes, J. (2022). Contributions from the ethico-onto-epistemological perspective for research in the field of education policy. *Education Policy Analysis Archives*, *30*, 146–146.

Manathunga, C. (2009). Post-colonial perspectives on interdisciplinary researcher identities. *Academic Research and Researchers*, 131–145.

McLeod, K., Thakchoe, S., Hunter, M.A., Vincent, K., Baltra-Ulloa, A.J., & MacDonald, A. (2020). Principles for a pedagogy of unlearning. *Reflective Practice*, *21*(2), 183–197.

Mcpherson, A., Forster, D., & Kerr, K. (2023). Controversial issues in the Australian educational context: Dimension of politics, policy and practice. *Asia-Pacific Journal of Teacher Education*, *51*(2), 113–127.

Moreton-Robinson, A. (2020). I still call Australia home: Indigenous belonging and place in a white postcolonizing society. In *Uprootings/regroundings* (pp. 23–40). Routledge.

Ngarrngga. (2023). Welcome to Ngarrngga. https://www.ngarrngga.org/.

Ngarrngga. (2024). Building a Ngarrngga curriculum. https://www.ngarrngga.org/stories-news/building-a-ngarrngga-curriculum.

OUTSIDE THE BOX / Earth Arts Rights (2023). Water[shed] Restore Pedder. https://outsidethebox.org.au/projects/watershed-restore-pedder/.

Park, W., & Cho, H. (2022). The interaction of history and STEM learning goals in teacher-developed curriculum materials: Opportunities and challenges for STEAM education. *Asia Pacific Education Review*, *23*(3), 457–474.

Restore Pedder (2023). Restore Pedder. https://lakepedder.org/.

Riley, T., Monk, S., & VanIssum, H. (2019). Barriers and breakthroughs: Engaging in socially just ways towards issues of indigeneity, identity, and whiteness in teacher education. *Whiteness and Education*, *4*(1), 88–107.

Rimmer, Z., & Taylor, R. (2023). An analysis of the 2021 apologies by the Royal Society of Tasmania and the Tasmanian Museum and Art Gallery to the Tasmanian Aboriginal Community. *Australian Historical Studies*, *54*(1), 77–90.

Rittel, H.W., & Webber, M.M. (1973). Dilemmas in a general theory of planning. *Policy Sciences*, *4*(2), 155–169.

Rousell, D. (2020). Doing little justices: Speculative propositions for an immanent environmental ethics. *Environmental Education Research*, *26*(9-10), 1391–1405.

Rousell, D., Cutter-Mackenzie, A., & Foster, J. (2017). Children of an earth to come: Speculative fiction, geophilosophy and climate change education research. *Educational Studies*, *53*(6), 654–669.

Ruanglertbutr, P. (Ed.). (2014). *Gallery and museum education: Purpose, pedagogy and practice*. Purnima Ruanglertbutr.

Silver, D. (2022). A theoretical framework for studying teachers' curriculum supplementation. *Review of Educational Research*, *92*(3), 455–489.

Sobe, N.W. (2021). *Reworking four pillars of education to sustain the commons*. UNESCO Futures of Education Ideas LAB.

Stacey, M., Gavin, M., Fitzgerald, S., McGrath-Champ, S., & Wilson, R. (2023). Reducing teachers' workload or deskilling 'core' work? Analysis of a policy response to teacher workload demands. In *Discourse: Studies in the cultural politics of education* (pp. 1–13).

Steffen, W., Broadgate, W., Deutsch, L., Gaffney, O., & Ludwig, C. (2015). The trajectory of the Anthropocene: The great acceleration. *Anthropocene Review*, *2*(1), 81–98.

Tasmanian Aboriginal Centre (n.d.). Pulingina to lutruwita (Tasmania) Place Names Map. https://tacinc.com.au/pulingina-to-lutruwita-tasmania-place-names-map/.

Tuomi, I. (2013). Open educational resources and the transformation of education. *European Journal of Education*, *48*(1), 58–78.

UNESCO (2021). *Reimagining our futures together: A new social contract for education*. Educational and Cultural Organization of the United Nations.

United Nations [UN]. (2016). Sustainable Development Goals (SDGs). Goal 4: Quality Education. https://www.un.org/%20sustainabledevelopment/education/.

United Nations [UN]. (2021). Climate Change 'Biggest Threat Modern Humans Have Ever Faced', World-Renowned Naturalist Tells Security Council, Calls for Greater Global Cooperation. Meetings coverage and press releases. https://www.un.org/press/en/2021/sc14445.doc.htm.

Usiskin, Z. (2013). Studying textbooks in an information age—A United States perspective. *ZDM Mathematics Education*, *45*(5), 713–723. DOI: 10.1007/s11858-013-0514-6.

Warren, B., Vossoughi, S., Rosebery, A.S., Bang, M., & Taylor, E.V. (2020). Multiple ways of knowing*: Re-imagining disciplinary learning. In *Handbook of the cultural foundations of learning* (pp. 277–294). Routledge.

Whatman, S., Wilkinson, J., Kaukko, M., Vedeler, G.W., Blue, L.E., & Reimer, K.E. (2023). *Researching practices across and within diverse educational sites: Onto-epistemological considerations*. Emerald Publishing Limited.

Wise, K., MacDonald, A., Badham, M., Brown, N., & Rankin, S. (2022). Interdisciplinarity for social justice enterprise: Intersecting education, industry and community arts perspectives. *The Australian Educational Researcher*, *49*(3), 595–615.

Author Biographies

Abbey MacDonald is a Senior Lecturer in Arts Education at the University of Tasmania. Her interdisciplinary arts-based research is used to inform arts, cultural and education policy development and museum and gallery education resources. She partners with environmental conservation and social change organisations to tackle some of the most pressing challenges educators face today.

Kim Beasy is a Senior Lecturer in Curriculum and Pedagogy at the University of Tasmania. Kim centres her work in education for sustainability, and her teaching and research emphasise engaging diverse communities in social, environmental, economic and cultural dimensions of sustainability.

Cite this article: MacDonald, A., & Beasy, K. (2024). A Water[shed] Moment for Articulating a Professional Practice of Education Resource Creation. *Australian Journal of Environmental Education*. https://doi.org/10.1017/aee.2024.22
