# Supplementary figures and images for: Dietary fibre intake in the adult Swiss population: a comprehensive analysis of timing and sources
Source: J Nutr Sci. 2025 Mar 24;14:e27. doi: 10.1017/jns.2025.6 (PMC11950702; doi:10.1017/jns.2025.6)

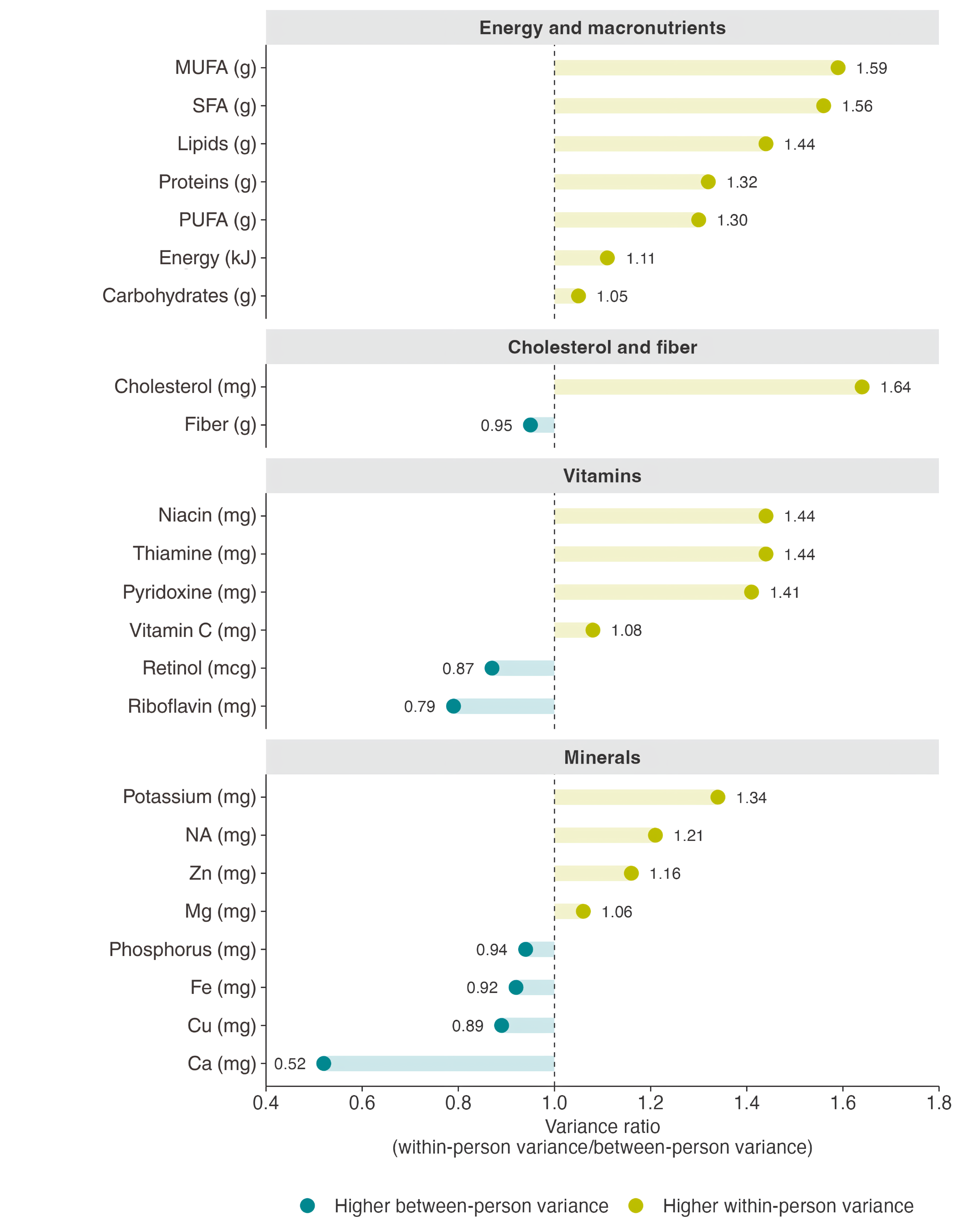

Supplement: von Blumenthal et al. supplementary material 2 — von Blumenthal et al. supplementary material [file S2048679025000060sup002.zip › Figure 1_AU (1).png]

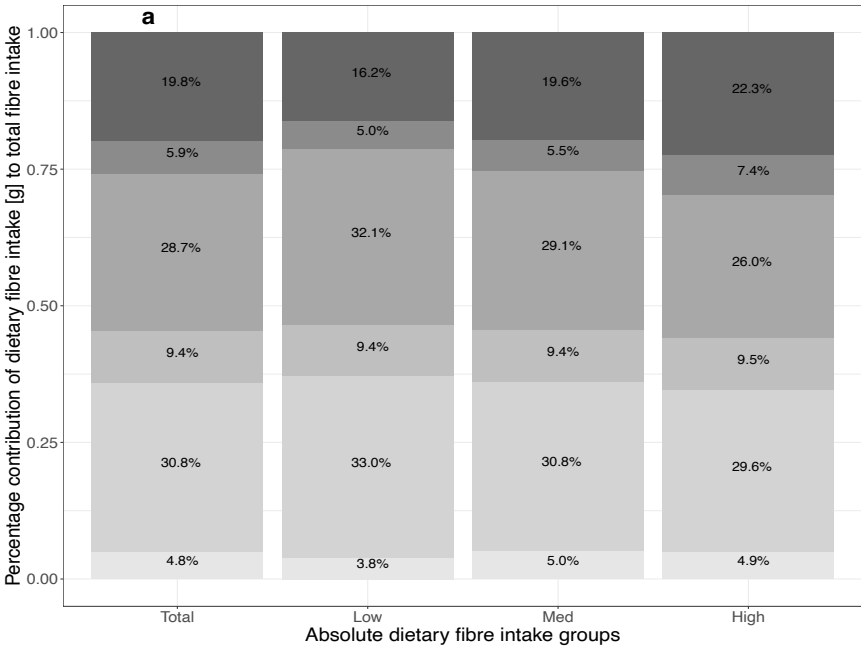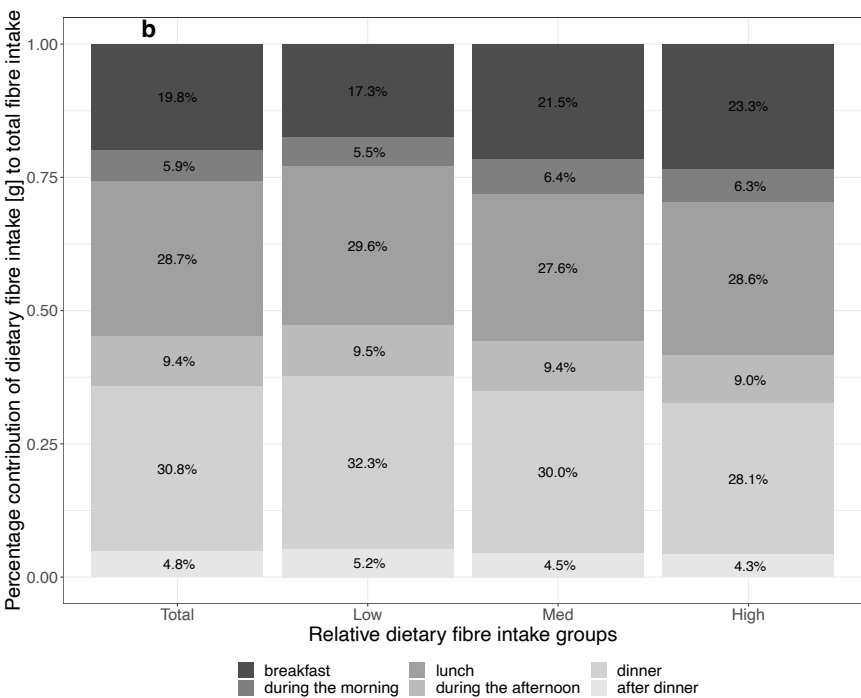

Supplement: von Blumenthal et al. supplementary material 2 — von Blumenthal et al. supplementary material [file S2048679025000060sup002.zip › Supplementary_Figure12025.pdf]
