# Supplementary material for: Dietary fibre intake in the adult Swiss population: a comprehensive analysis of timing and sources
Source: J Nutr Sci. 2025 Mar 24;14:e27. doi: 10.1017/jns.2025.6 (PMC11950702; doi:10.1017/jns.2025.6)
Supplement: von Blumenthal et al. supplementary material 1 — von Blumenthal et al. supplementary material [file S2048679025000060sup001.zip › wet_2500007_AM (1).zip!/wet_2500007_AM/AM PO_wet_2500007.pdf]

Cambridge Journals

29 January

Purchase order for Accepted Manuscript Open Access

Please refer to the proofing instructions for this journal
Proofs MUST be delivered by the time agreed in the service level agreement

article no.	WT-D-24-00159	setters no.	2500007	wet	0	running order
first named author	Alice Wright	author email:	alice.wright@usda.gov,alice.wright@usda.gov			
corresp au	Alice Wright					
title	Evaluation of spring herbicide programs during a three-year sugarcane (Saccharum spp. hybrids) cropping cycle					
doi	10.1017/wet.2025.7	no. of book revs		open_access_article	Y	

submission dates	received	revised	accepted
	31/10/24	10/1/25	26/1/25

article type **Research Article**

print colour **no** web colour **no**

copyright line © Agricultural Research Service, United States Departement of Agriculture (USDA-ARS), [YYYY]. Th
work of the US Government and is not subject to copyright protection within the United States. Publis
by Cambridge University Press on behalf of Weed Science Society of America. This is an Open Acce
article, distributed under the terms of the Creative Commons Attribution licence (http://creativecommc
org/licenses/by/4.0), which permits unrestricted re- use, distribution and reproduction, provided the or
article is properly cited.

notes for setter

supplementary materials Yes Table S1.xlsx Table S2.xlsx

Purchase order for Accepted Manuscript Open Access

funding body	author country	assoc ed
