# Supplementary material for: Dietary fibre intake in the adult Swiss population: a comprehensive analysis of timing and sources
Source: J Nutr Sci. 2025 Mar 24;14:e27. doi: 10.1017/jns.2025.6 (PMC11950702; doi:10.1017/jns.2025.6)
Supplement: von Blumenthal et al. supplementary material 1 — von Blumenthal et al. supplementary material [file S2048679025000060sup001.zip › wet_2500007_AM (1).zip!/wet_2500007_AM/WT-D-24-00159.pdf]

Weed Technology

Evaluation of spring herbicide programs during a three-year sugarcane (Saccharum spp. hybrids) cropping cycle

--Manuscript Draft--

**Manuscript Number:**	WT-D-24-00159R1
**Full Title:**	Evaluation of spring herbicide programs during a three-year sugarcane (Saccharum spp. hybrids) cropping cycle
**Short Title:**	Sugarcane herbicide programs
**Article Type:**	Research Article
**Section/Category:**	Weed Management - Other Crops/Areas
**Keywords:**	HPPD-inhibitor; itchgrass; sugarcane
**Corresponding Author:**	Alice Ann Wright, Ph.D.<br>USDA-ARS: USDA Agricultural Research Service<br>UNITED STATES
**Corresponding Author Secondary Information:**	
**Corresponding Author's Institution:**	USDA-ARS: USDA Agricultural Research Service
**Corresponding Author's Secondary Institution:**	
**First Author:**	Alice Ann Wright, Ph.D.
**First Author Secondary Information:**	
**Order of Authors:**	Alice Ann Wright, Ph.D.
	Douglas J. Spaunhorst
	Eric Petrie
**Order of Authors Secondary Information:**	
**Abstract:**	A limited number of herbicides, and modes of action, are registered for use in sugarcane in Louisiana. Repeated use of the same modes of action can lead to the evolution of herbicide resistance in weeds. Therefore, it is critical to evaluate additional modes of action to provide growers with options for rotating herbicides to reduce the risk of resistance. Topremazone, indaziflam, and a formulation including mesotrione, bicyclopyrone, atrazine, and S-metolachlor along with more common herbicide applications (pendimethalin and metribuzin, clomazone and diuron), were evaluated in the spring for injury to sugarcane, weed control, sugarcane yield, and sugar yield. Of these treatments, clomazone applied with diuron was the only herbicide combination to consistently injure the crop, with injury estimates ranging from 11 to 36%, which frequently resulted in reduced sugar yield with losses between 2.3% to 24.1% of the non-treated control. In most treatments, an increase in itchgrass (Rottboellia cochinchinensis) counts was observed between harvests, indicating that additional control strategies will be needed in fields infested with this weed. However, topramezone alone and with triclopyr was well tolerated by sugarcane with injuries ranging from 0 to 11% two weeks after treatment. Indaziflam and combined application of mesotrione, bicyclopyrone, atrazine and S-metolachlor injury was at or under 10% at two weeks after treatment. The tolerance of sugarcane for these herbicides suggests that they can be incorporated into weed management strategies in sugarcane. Use of these herbicides would increase the modes of action available to be applied in sugarcane and help mitigate the risk of herbicide resistant weeds.

Powered by Editorial Manager® and ProduXion Manager® from Aries Systems Corporation

Click here to access/download
Response to Reviewers
Response to reviewer comments.docx

Manuscript (ONE file with all tables and figures but not supplementary files)

Click here to access/download;Manuscript (ONE file with all tables and figures but not supplementary files);WT-D-24-

Short Title: Sugarcane herbicide programs

Evaluation of spring herbicide programs during a three-year sugarcane (*Saccharum* spp. hybrids) cropping cycle

Alice A. Wright[1], Douglas J. Spaunhorst[2] and Eric Petrie[3]

[1] Research Agronomist (0000-0003-4814-4372), U.S. Department of Agriculture, Agricultural Research Service, Sugarcane Research Unit, Houma, LA, 70360; [2] Research Agronomist, U.S. Department of Agriculture, Agricultural Research Service, Sugarcane Research Unit, Houma, LA, 70360; current position: Director of Agronomy Services, MFA Incorporated, Columbia, MO, 65201; [3] Research Technician, Retired, U.S. Department of Agriculture, Agricultural Research Service, Sugarcane Research Unit, Houma, LA, 70360

Author for correspondence:

Alice Wright; alice.wright@usda.gov

Abstract

A limited number of herbicides and sites of action are registered for use in sugarcane in Louisiana. Repeated use of the same sites of action can lead to the evolution of herbicide resistance in weeds. Therefore, it is critical to evaluate additional sites of action to provide growers with options for rotating herbicides to reduce the risk of resistance. Topramezone, indaziflam, and a formulation including mesotrione, bicyclopyrone, atrazine, and *S*-metolachlor, along with more

common herbicide applications (pendimethalin, and metribuzin, clomazone, and diuron), were evaluated in the spring for injury to sugarcane, weed control, sugarcane yield, and sugar yield. Of these treatments, clomazone applied with diuron was the only herbicide combination to consistently injure the crop, with injury estimates ranging from 11 to 36%, which frequently resulted in reduced sugar yield with losses between 2.3% to 24.1% of the non-treated control. In most treatments, an increase in itchgrass counts was observed between harvests, indicating that additional control strategies will be needed in fields infested with this weed. However, topramezone alone and with triclopyr was well tolerated by sugarcane, with injuries ranging from 0 to 11% two weeks after treatment. Indaziflam and combined application of mesotrione, bicyclopyrone, atrazine, and *S*-metolachlor injury was at or under 10% two weeks after treatment. The tolerance of sugarcane for these herbicides suggests that they can be incorporated into weed management strategies in sugarcane. These herbicides would increase the sites of action available to be applied in sugarcane and help mitigate the risk of herbicide-resistant weeds.

Nomenclature: Atrazine; bicyclopyrone; clomazone; diuron; indaziflam; mesotrione; metribuzin; pendimethalin; *S*-metolachlor; topramezone; triclopyr; itchgrass, *Rottboellia cochinchinensis* (Lour.) W.D. Clayton, sugarcane, *Saccharum* spp. hybrids.

Keywords: HPPD-inhibitor, itchgrass, sugarcane

Introduction

Sugarcane is a perennial grass crop that, in Louisiana, is planted in August or September and harvested in the fall of the following year with two or more ratoon crops harvested in subsequent years before replanting. Weed management in sugarcane relies primarily on tillage and herbicide application. However, few herbicides and fewer sites of action are registered for use (Orgeron and Wright 2023). This can lead to growers applying the same herbicides year after year, setting the stage for the evolution of herbicide-resistant weeds. A more diversified herbicide program in sugarcane is needed.

As sugarcane is a perennial grass crop, the most difficult-to-manage weeds are grasses and sedges, including itchgrass and bermudagrass [*Cynodon dactylon* (L.) Pers.], johnsongrass [*Sorghum halepense* (L.) Pers.], yellow nutsedge (*Cyperus esculentus* L.), and purple nutsedge (*Cyperus rotundus* L.). These weeds can cause significant yield losses if left unchecked. Itchgrass is one of the worst weeds in sugarcane, with severe infestations causing up to 43% reduction in sugar yields (Lencse and Griffin 1991) or more (Millhollon 1992). Bermudagrass infestations, when severe, can reduce the number of harvestable stalks, thereby decreasing yield. These yield reductions can range from 8 to 32% depending upon the harvest year for the crop (Richard and Dalley 2007). If not managed early, bermudagrass interference can have a cumulative effect, with yields declining after subsequent crop harvests (Richard 1993). Purple nutsedge infestations can also reduce yield: in pot studies, sugarcane shoot counts and shoot height decreased as nutsedge tuber density increased (Etheredge et al. 2010a). In addition to grasses and sedges, morningglory (*Ipomoea* sp.) is a problem as it can twine around the mature stalks and interfere with harvest. Controlling these weeds early with a preemergence herbicide, prior to canopy closure, is critical,

as morningglory can germinate after canopy closure. When left uncontrolled red morningglory (*Ipomoea coccinea* L.) can reduce yield by 27% (Jones and Griffin 2009).

Growers rely on applications of pre-emergence herbicides after planting, and twice more in early spring and in May or June before canopy closure. Preemergence herbicides are critical as post-emergence options for grass control in sugarcane are few, mostly being limited to asulam herbicide alone (Millhollon 1976, Richard 1990, Richard and Griffin 1993) or applied with a sulfonylurea (Dalley and Richard 2008). Paraquat can be applied in the late winter for weed control without substantially affecting yield (Griffin et al. 2004). However, resistance to this herbicide has been confirmed in Italian ryegrass (*Lolium multiflorum* Lam.) (Coco 2022). Common pre-emergence applications include pendimethalin and metribuzin; pendimethalin is generally effective against itchgrass (Millhollon 1993). Metribuzin can provide enough suppression of bermudagrass to prevent yield reductions throughout the multi-year life-cycle of the crop (Richard 1993). A combination of reduced or conventional tillage with broadcast applications of pendimethalin and metribuzin have been found to be most effective at reducing bermudagrass cover (Dalley et al. 2013). Clomazone and diuron applied in early spring can cause up to 85% injury in bermudagrass (Spaunhorst 2021). Triclopyr applied with a PSII inhibitor, such as hexazinone or diuron, caused injury to this weed at similar rates when applied in early spring (Spaunhorst 2021). For management of red morningglory at layby, atrazine, diuron and hexazinone, or flumioxazin applied as a post-emergence application provided 90% control. As a pre-emergence herbicide, sulfentrazone provided the longest control of red morningglory with 82% control at 77 days after treatment (Jones and Griffin 2008). In another study, pre-emergence azafenidin and sulfentrazone separately provided 90% or greater control of red morningglory,

however this control decreased in the absence of rain after herbicide application (Viator et al. 2002).

HPPD inhibitors registered for use in sugarcane provide an alternative site of action to the dinitroanalines and PSII inhibitors that are frequently applied. Registered chemistries include mesotrione and topramezone (Jhala et al. 2023). These herbicides inhibit the 4-hydroxyphenylpyruvate dioxygenase enzyme, resulting in bleaching of susceptible plants and eventual plant death (Schulz et al. 1993). Another recently registered chemistry, indaziflam, acts as a cellulose biosynthesis inhibitor (Brabham et al. 2014). Both topramezone and indaziflam have been used with success outside the United States. Topramezone was well tolerated in sugarcane varieties planted in China and was effective in controlling common grasses and broadleaf weeds (Ma et al. 2023). In Iran, testing of multiple rates of indaziflam showed an increase in sugarcane yield and a reduction in weed biomass (Sharafizadeh and Nikpay 2023). Indaziflam was also effective against morninglory and itchgrass in sugarcane production in Brazil (de Castro 2024). Rotating these herbicides with current herbicide strategies in Louisiana sugarcane production would broaden the sites of action applied to sugarcane and reduce the risk of weeds evolving herbicide resistance. To that end, herbicide programs incorporating HPPD inhibitors or indaziflam were evaluated alongside more commonly used herbicide programs for their weed control efficacy and effects on yield.

Materials and Methods

Experimental Location, Design, and Field Preparation Description

Field studies were conducted from 2016 to 2020 at the USDA-ARS Sugarcane Research Unit Ardoyne Farm in Schriever, LA (29.64°N, 90.84°W) having HoCP 96-540 (Tew et al. 2005)

and L 01-299 (Gravois et al. 2011) sugarcane planted as separate trials with two replicates (test 1 and test 2) for each variety, planted a year apart. HoCP 96-540 and L 01-299 were selected as they were the predominant varieties in the industry at the time, covering approximately 37% and 22% of the acreage of sugarcane in 2014 (Gravois and Legendre 2014). Herbicide treatments were arranged in a randomized complete block design with four replications. Each plot was three rows wide (5.5 m) by 9.1 m long, and rows were spaced 1.8-m apart. The whole stalks of each variety were hand planted with three stalks placed parallel to each other in the furrow and overlapping the next set by about 10% to reduce the potential for gaps. Once in the furrows, the stalks were covered with 7-8 cm of soil by pulling soil from each edge of the furrow using disk blades and packed with a land roller implement. Plots were maintained according to standard practice: furrows were cultivated in mid-March and 32% liquid urea ammonium nitrate was knifed in at 134 kg ha^{-1} and immediately incorporated in mid to late April. Herbicide was applied in mid-March and sugarcane was harvested in the fall (Table 1). Plots were grown for two subsequent years as ratoon crops.

Herbicide application

Herbicides were applied to plots in the spring after sugarcane emerged from winter dormancy which typically occurred when the most recently formed leaf collar measured 5-cm tall. A total of twelve different treatments, including a non-treated (weedy) control, were evaluated (Table 2). A crop oil concentrate, Grounded (Helena Agri-Enterprises, LLC, Collierville, TN), was added at a 1% v/v to treatments containing topramezone. Herbicides were applied from a multi-boom sprayer attached to the three-point hitch on a tractor. XR11003 VS flat-fan nozzle tips (TeeJet®, Spraying Systems Co., Glendale Heights, IL) were used and the sprayer was calibrated for 187 L ha^{-1}. Treatment dates are provided in Table 1. An additional treatment of 2130 g ai ha^{-1} of pendimethalin (Prowl H_2O, BASF, Research Triangle Park, NC) and 840 g ai ha^{-1} of metribuzin

(Tricor DF, UPL, Cary, NC) was applied at the end of May prior to canopy closure. In plant cane, the crop was clipped in early spring and the mowed cane leaves were incorporated with cultivation prior to the herbicide applications. Due to the severity of winter annual weeds in the second ratoon crops, dicamba and 2,4-D (Weedmaster, Nufarm, Alsip, IL) were applied at 140 and 400 g ae ha^{-1}, respectively, in mid-February.

Data collection

Crop injury was visibly assessed two weeks after treatment and scored on a scale of 0 to 100 with 0 being no injury and 100 being plant death. Weed density was assessed in August each year, five months after herbicide application. At approximately the center of each plot on the hipped bed, two 0.3 m^2 quadrants were placed adjacent to the sugarcane. Weed density and species present were recorded. Stalk counts for each plot were recorded in the summer each year, three months after herbicide application. For each plot, the height of 12 random stalks was recorded in July each year, four months after herbicide application. Plots were harvested using a combine chopper harvester and cane collected in a modified dump wagon with load cells in the axle and tongue that recorded total sugarcane yield (Johnson and Richard, 2005). The dump wagon enabled collecting a sample of the billets being harvested that was later processed for sucrose content. Billets were crushed in a roller mill and the juice collected for Brix and pol determination using a refractometer and saccharimeter. Theoretical recoverable sucrose (TRS) was calculated according to Chen and Chou (1993). Total sugar yield per plot was estimated by multiplying sugarcane yield by TRS.

Statistical Analysis

All statistical analyses were performed in R (v 4.3.1) using the tidyverse and ggplots2 packages. Where there were no significant differences, duplicate test years were combined for each

harvest for individual varieties. Where this could not be done, data were presented separately as either test 1 or test 2. Data were checked for normality and equal variance using a Shapiro-Wilk test and an F-test, respectively. When these conditions were met, ANOVA was performed followed by Tukey's HSD where the ANOVA detected significant differences. Where normality was not met (this was often the case for crop injury), a Kruskal-Wallis test was performed.

Results and Discussion

Crop response to herbicide treatment

Crop response to herbicide application varied between test years for both varieties and could not be grouped together for analysis (Table 3). Overall, treatment with clomazone and diuron caused the most injury to the crop. Injury two weeks after application ranged from 15% to 36% for L 01-299 and from 11 to 31% for HoCP 96-540. It has been well established that clomazone can cause injury to sugarcane and potentially reduce yield (Richard 1996). Some older varieties of sugarcane have shown injury and yield loss to high rates of diuron (Millhollon and Matherne 1968). Other herbicide treatments also caused injury, but this was less severe and varied between test years. Treatment with Acuron (S-metolachlor, atrazine, mesotrione, and bicyclopyrone) caused minor injury, 3 to 10 %, across all years for both tests for L 01-299 and for all but the second test in plant cane in HoCP 96-540. In HoCP 96-540, the higher rate of topramezone with triclopyr caused mild injury, 1 to 9% across all years in both test years. Topramezone alone caused mild to no injury: 0 to 8% at 22.4 g ai ha^{-1} and 0 to 10% at 56.1 gi ha^{-1}. Indaziflam injury was low, ranging from 0 to 8% in L 01-299 and 0 to 6% in HoCP 96-540.

Weed response to herbicide treatment

Weed counts and species were evaluated in each plot in August, five months after herbicide application. Overall, there were similar shifts in species composition over time for both varieties (Figure 1). Initially, the plant cane chamber-bitter *(Phyllanthus urinaria* L.) was the predominant weed. However, this weed decreased in incidence with subsequent harvests and was absent by the third harvest, except for test 1 for HoCP 96-540. Purple nutsedge increased in incidence from the first harvest to the third, except in test 1 for HoCP 96-540, where incidence decreased from the second harvest to the third harvest. Of particular concern is the increase in itchgrass incidence following subsequent harvests for L 01-299 (Figure 1). Closer examination of individual treatments did show some significant differences between harvest years for both itchgrass and purple nutsedge (Figures 2 and 3). While there were no statistically significant differences between harvests in test 1 (Figure 2A), there were increases in the number of itchgrass plants over subsequent harvests for topramezone at 22.4 g ai ha^{-1}, clomazone and diuron, Acuron (S-metolachlor, atrazine, mesotrione, and bicyclopyrone), and indaziflam. In test 2, the increase in the number of itchgrass plants with subsequent harvests was more pronounced and observed in all treatments (Figure 2B). This may be due to heavy rainfall and flooding in October of 2019 that may have contributed to the spread of itchgrass seed that year, leading to heavier infestations the following year. As test 1 concluded in fall of 2019 but test 2 did not conclude until fall of 2020, this flooding may account for the difference in significance for itchgrass counts between the two tests. The increases in itchgrass counts in the third harvest from previous harvests were significant for topramezone at 22.4 g ai ha^{-1}, metribuzin at 1680 g ai ha^{-1}, metribuzin at 2520 g ai ha^{-1}, topramezone at 44.9 g ai ha^{-1} with triclopyr, and Acuron. This suggests that supplemental control strategies will be needed in fields where itchgrass is a problem. These supplemental control options are principally asulam applied post-emergence or pendimethalin applied pre-emergence

(Millhollon 1993). While not as stark as the increase from harvest year 1 to harvest year 3 for itchgrass, purple nutsedge showed a similar trend across treatments (Figure 3). In both tests, treatment 4, pendimethalin and atrazine, showed a significant increase in purple nutsedge counts by harvest year 3. Purple nutsedge is difficult to manage in sugarcane and severe infestations require postemergence treatment with a sulfonylurea, such as halosulfuron (Etheredge et al. 2010b).

Crop Yield

Stalk counts and heights were assessed for each plot. There were no significant differences for stalk counts, but there were for stalk heights (Table S1). Plots treated with clomazone and diuron frequently had the shortest stalks, which likely is due to enhanced crop injury observed in sugarcane following herbicide treatment. Stalk height was otherwise not consistent and varied between treatment and crop year for each variety.

Plot weights were collected for each plot at harvest. There were no significant differences in plot weights for HoCP 96-540, however there were for L 01-299 (Table S2). Across both test years and all harvests, plots treated with clomazone and diuron consistently had the lowest plot weight, although this difference was not always significant. This decrease in weight is likely due to the shorter stalks and the herbicide injury to the crop.

For most harvests across both test years and varieties, TRS was not significant (data not shown). However, for total sugar per hectare (combining plot weight and TRS), there were no significant differences between treatments for L 01-299 (Table 4). For HoCP 96-540, significant differences were only observed in test 1 of the plant cane and for first ratoon. In both instances, the lowest yield was for plots treated with clomazone and diuron, although for first ratoon, this was not significantly different from treatment with pendimethalin and atrazine. The reduced yield

for sugarcane treated with clomazone and diuron is likely a result of the shorter stalks (Table S1) and lower plot weight (Table S2).

These data reiterate the need to exercise caution when applying clomazone to sugarcane in the spring after dormancy as this treatment can negatively impact sucrose yield more than weed competition alone. However, the findings also suggest that the herbicides examined here are viable options for weed management in sugarcane. While topramezone and triclopyr caused mild injury, there was no effect on yield. For growers with bermudagrass infestations, these herbicides could be incorporated into a weed management strategy. Triclopyr, when paired with an HPPD inhibitor like topramezone or mesotrione, can suppress bermudagrass (Brosnan and Breeden 2013, Spaunhorst 2021). Spaunhorst (2021) observed up to 62% injury, which may be sufficient suppression to allow for canopy closure before bermudagrass can interfere with the sugarcane to affect yield. Although not an HPPD inhibitor, indaziflam was included in this study as Alion® was recently registered for use in sugarcane. Indaziflam alone has not been found effective in preventing purple nutsedge emergence but was effective in managing doveweed (Ramanathan et al. 2023).

The limited number of herbicides registered for use in sugarcane (Orgeron and Wright 2023) highlights the need to diversify herbicide programs as much as possible to reduce the risk posed by herbicide-resistant weeds. HPPD inhibitors such as topramezone are ideal for this as resistance has been reported in few species, limited thus far too wild radish (*Raphanus raphanistrum* L.), waterhemp (*Amaranthus tuberculatus* (Moq.) Sauer), and Palmer Amaranth (*Amaranthus palmeri* L.) (Busi et al. 2022, Hausman et al. 2011, Jhala et al. 2014). The wild radish population was selected for in Australia by repeated applications of pyrasulfotole but was also resistant to mesotrione and topramezone (Busi et al. 2022). In Illinois, resistant waterhemp was

observed after annual HPPD inhibitor applications, either mesotrione, topramezone, or tembotrione. Most concerning is that this population was also resistant to atrazine, which was also applied with an HPPD inhibitor for several years (Hausman et al. 2011). HPPD inhibitor and triazine resistance was also confirmed in Palmer Amaranth (Jhala et al. 2014). Indaziflam resistance thus far has only been reported for annual bluegrass (*Poa annua* L.) (Brosnan et al. 2020). While these weed species are currently not problematic in Louisiana sugarcane, topramezone and indaziflam should be used in rotation with other herbicides to diversify sites of action and reduce the risk for resistance evolution.

Most interestingly, the shift in weed species overall highlights the need to rotate herbicides between years. Special care will need to be taken with respect to itchgrass. Itchgrass is one of the worst weeds in the world, in part due to its ability to self-pollinate and its prolific seed production (Holm et al. 1997, Millhollon and Burner 1993). As this highly competitive weed can significantly decrease sugarcane yield (Lencse and Griffin 1991, Millhollon 1992) growers will need to be vigilant in scouting for it in fields and surrounding areas. The herbicide treatment strategies here will need to be adjusted for managing itchgrass in fields where it is established. It would also be of interest to, on a larger scale, examine shifts in weed populations under different management strategies. Sugarcane is unique among row crops in that, as a perennial, it is kept in the ground for four years or longer. As weed pressure can cause a decrease in yield over subsequent harvests, it is important to understand how weed species adapt to sugarcane production and how weed management strategies need to be tailored to reduce the effect of those weeds and promote crop longevity. The impact of weather and how it contributes to weed seed dispersal, as was suspected of playing a role in the increased incidence of switchgrass in test 2, also needs to be considered.

Practical Implications

Louisiana sugarcane growers currently have a limited number of herbicides and sites of action registered for use. This increases the likelihood that herbicide resistance will evolve in weeds. Any additional sites of action, like HPPD-inhibitors or cellulose biosynthesis inhibitors, can help diversify herbicide application programs and reduce the risk for resistance. In addition, itchgrass is the worst weed currently facing Louisiana sugarcane growers. This research shows that growers cannot rely on a single site of action alone year after year as weed pressure, especially itchgrass, will increase in subsequent ratoon crops, requiring that the field be rotated into a fallow period prior to replanting. Ratoon longevity is a priority among growers due to the expense of replanting and weed management, particularly with respect to aggressive weeds like itchgrass, is a critical component of extending ratoon longevity and delaying replanting.

Funding

This research was funded by the USDA-ARS Sugarcane Research Unit. Mention of trade names or commercial products in this publication is solely for the purpose of providing specific information and does not imply recommendation or endorsement by the U.S. Department of Agriculture. USDA is an equal opportunity provider and employer.

Competing Interests: the authors declare none.

References

Brabham C, Lei L, Bu Y, Stork J, Barrett M, DeBolt S (2014) Indaziflam herbicidal action: a potent cellulose biosynthesis inhibitor. Plant Phys 166:1177-1185

Brosnan JT, Breeden GK (2013) Bermudagrass (*Cynodon dactylon*) control with topramezone and triclopyr. Weed Technol 27:138-142

Brosnan JT, Vargas JJ, Spesard B, Netzband D, Zobel JM, Chen J, Patterson EL (2020) Annual bluegrass resistance to indaziflam applied early-postemergence. Pest Manag Sci 76:2049-2057.

Busi R, Zhang B, Goggin D, Bryant G, Beckie HJ (2022) Identification of field resistance to HPPD-inhibiting herbicides in wild radish (Raphanus raphanistrum). Weed Sci 70:529-536

Chen JCP, Chou CC (1993) Cane sugar handbook: A manual for cane sugar manufactures and their chemists. 12th ed. New York: J. Wiley & Sons

Coco AB, III (2022) Paraquat-resistant Italian ryegrass (*Lolium multiflorum*) confirmed in Louisiana. M.S. Thesis. Baton Rouge, LA: Louisiana State University

Dalley CD, Richard Jr. EP (2008) Control of rhizome Johnsongrass (*Sorghum halepense*) in sugarcane with trifloxysulfuron and asulam. Weed Technol 22:397-401

Dalley CD, Viator RP, Richard Jr. EP (2013) Integrated management of bermudagrass (*Cynodon dactylon*) in sugarcane. Weed Sci 61:482-490

De Castro RA, de Castro SGQ, de Castro SAQ, Piassa A, Soares GOdN, Tropaldi L, Christofoletti PJ (2024) Optimizing herbicide selection for pre-emergence control of itchgrass and cypressvine morningglory in sugarcane. J Env Sci and Health 59:350-360

Etheredge LM, Griffin JL, Boudreaux JM (2010a) Purple nutsedge (*Cyperus rotundus*) competition with sugarcane and response to shade. J Am Soc Sugar Cane 30:89-103

Etheredge LM, Griffin JL, Boudreaux JM (2010b) Nutsedge (*Cyperus* spp.) control programs in sugarcane. J Am Soc Sugar Cane 30:67-80

Gravois KA, Bischoff KP, Pontif MJ, LaBorde CM, Hoy JW, Reagan TE, Kimbeng CA, Legendre BL, Hawkins GL, Sexton DR, Fontenot DP (2011) Registration of 'L 01-299' sugarcane. J Plant Registrations 5:191-195

Gravois KA, Legendre BL (2014) The 2014 Louisiana sugarcane variety survey. Pages 102-105 *in* Sugarcane research annual progress report 2014. LSU AgCenter. Retrieved from https://www.lsuagcenter.com/portals/our_offices/research_stations/sugar/features/annual_reports/2014-sugarcane-annual-progress-report.

Griffin JL, Miller DK, Ellis JM, Clay PA (2004) Sugarcane tolerance and Italian ryegrass (*Lolium multiflorum*) control with paraquat. Weed Technol 18:555-559

Hausman NE, Singh S, Tranel PJ, Riechers DE, Kaundun SS, Polge ND, Thomas DA, Hager AG (2011) Resistance to HPPD-inhibiting herbicides in a population of waterhemp (*Amaranthus tuberculatus*) from Illinois, United States. Pest Manag Sci 67:258-261

Holm LG, Plucknett DL, Pancho JV, Herberger JP (1977) "*Rottboellia exaltata*". Pages 139-144 *in* The world's worst weeds. Honolulu, HI: The University Press of Hawaii

Jhala AJ, Kumar V, Yadav R, Jha P, Jugulam M, Williams MM II, Hausman NE, Dayan FE, Burton PM, Dale RP, Norsworthy JK (2023) 4-Hydroxyphenylpyruvate dioxygenase (HPPD)-inhibiting herbicides: past, present, and future. Weed Technol 37:1-14

Jhala AJ, Sandell LD, Rana N, Kruger GR, Knezevic S (2014) Confirmation and control of triazine and 4-hydroxyphenylpyruvate dioxygenase-inhibiting herbicide-resistant Palmer Amaranth (*Amaranthus palmeri*) in Nebraska. Weed Technol 28:28-38

Johnson RM, Richard EP Jr (2005) Sugarcane yield, sugarcane quality, and soil variability in Louisiana. Agron J 97:760-771

Jones CA, Griffin JL (2008) Residual red morningglory (*Ipomoea coccinea*) control with foliar- and soil- applied herbicides. Weed Technol 22:402-407

Jones CA, Griffin JL (2009) Red morningglory (*Ipomoea coccinea*) control and competition in sugarcane. J Am Soc Sugar Cane 29:25-35

Lencse RJ, Griffin JL (1991) Itchgrass (*Rottboellia cochinchinensis*) interference in sugarcane (*Saccharum* sp.). Weed Technol 5:396-399

Ma Y-L, Guo C-L, Wang Y-H, Gao Y-Y, Qin J-L, Wei J-L (2023) Herbicidal activity evaluation of topramezone and its safety to sugarcane. Sugar Tech 25:698-704.

Millhollon RW (1976) Asulam for Johnsongrass control in sugarcane. Weed Sci 24:496-499

Millhollon RW, Burner DM (1993) Itchgrass (*Rottboellia cochinchinensis*) biotypes in world populations. Weed Sci 41:379-387

Millhollon R, Matherne RJ (1968) Tolerance of Sugarcane Varieties to Herbicides. Weed Technol 16:300-303

Millholon RW (1992) Effect of itchgrass (*Rottboellia cochinchinensis*) interference on growth and yield of sugarcane (*Saccharum* spp. hybrids). Weed Sci 40:48-53

Millhollon RW (1993) Preemergence control of itchgrass (*Rottboellia cochinchinensis*) and Johnsongrass (*Sorghum halepense*) in sugarcane (*Saccharum* spp hybrids) with pendimethalin and prodiamine. Weed Sci 41:621-626

Orgeron A, Wright A (2023) Sugarcane Weed Management. In 2023 Louisiana Suggested Chemical Weed Control Guide. LSU AgCenter. Pp68-88

Ramanathan SS, Gannon TW, Maxwell PJ (2023) Dose-response of five weed species to indaziflam and oxadiazon. Weed Technol 37:302-312

Richard Jr. EP (1990) Timing effects on johnsongrass (*Sorghum halepense*) control with asulam in sugarcane (*Saccharum* sp.). Weed Technol 4:81-86

Richard Jr. EP (1993) Preemergence herbicide effects on bermudagrass (*Cynodon dactylon*) interference in sugarcane (*Saccharum* spp. hybrids). Weed Technol 7:578-584

Richard Jr. EP (1996) Sugarcane (*Saccharum* spp. hybrids) tolerance to clomazone. Weed Technol 10:90-94

Richard Jr. EP, Dalley CD (2007) Sugarcane response to bermudagrass interference. Weed Technol 21:941-946

Richard Jr. EP, Griffin JL (1993) Johnsongrass (*Sorghum halepense*) control in sugarcane (*Saccharum* sp.) with asulam applied alone and in mixtures. Weed Technol 7:657-662

Schulz A, Ort O, Beyer P, Kleinig H (1993) SC-0051, a 2-benzoyl-cyclohexane-1,3-dione bleaching herbicide, is a potent inhibitor of the enzyme p-hydroxyphenylpyruvate dioxygenase. FEBS Lett 318:162-166

Sharafizadeh P, Nikpay A (2023) Investigating different concentrations of Alion® (indaziflam) herbicide in sugarcane fields in Iran. Proc. Int. Society Sugar Cane Technologists 31:1369-1371

Spaunhorst DJ (2021) Spring-applied triclopyr mixtures for bermudagrass (*Cynodon dactylon*) suppression in Louisiana sugarcane. Weed Technol 35:589-597

Tew TL, White WH, Legendre BL, Grisham MP, Dufrene EO, Garrison DD, Veremis JC, Pan Y-B, Richard EP, Miller JD (2005) Registration of ‘HoCP 96-540’ Sugarcane. Crop Sci. 45:785-786

Viator BJ, Griffin JL, Ellis JM (2002) Red morningglory (*Ipomoea coccinea*) control with sulfentrazone and azafenidin applied at layby in sugarcane (*Saccharum* spp.). Weed Technol 16:142-148

Table 1. Dates of sugarcane planting, herbicide application, and harvest at the Ardoyne Farm from 2015 to 2020.

Variety	Planting	Herbicide Application	Harvest
L 01-299	Sep 2016	Mar 17 2017	Nov 20 2017
		Mar 7 2018	Oct 31 2018
		Mar 21 2019	Nov 4 2019
	Aug 2017	Mar 8 2018	Nov 19 2018
		Mar 21 2019	Nov 6 2019
		Mar 11 2020	Sep 30 2020
HoCP 96-540	Aug 2015	Apr 8 2016	Dec 14 2016
		Mar 17 2017	Nov 17 2017
		Mar 7 2018	Oct 30 2018
	Sep 2016	Mar 17 2017	Nov 16 2017
		Mar 8 2018	Oct 31 2018
		Mar 21 2019	Nov 5 2019

Table 2. Herbicides applied in the spring.

Treatment Number	Herbicides	Product(s)	Rate g ai ha^{-1}	Manufacturer	City, State
1	Topramezone	Armezon	22.4	BASF	Research Triangle Park, NC
2	Topramezone	Armezon	56.1	BASF	Research Triangle Park, NC
3	Clomazone and Diuron	Command 3ME	1260	FMC Corporaton	Philadelphia, PA
		Direx 4L	2,800	Drexel Chemical Co.	Memphis, TN
4	Pendimethalin and Atrazine	Prowl H2O	3,200	BASF	Research Triangle Park, NC
		Atrazine 4L	2,240	Drexel Chemical Co.	Memphis, TN
5	Metribuzin	Tricor DF	1680	UPL	Cary, NC
6	Metribuzin	Tricor DF	2,520	UPL	Cary, NC
7	Pendimethalin and Metribuzin	Prowl H2O	3,200	BASF	Research Triangle Park, NC
		Tricor	2,520	UPL	Cary, NC
8	Topramezone and Triclopyr	Armezon	22.4	BASF	Research Triangle Park, NC
		Trycera	1,120	Helena Agri-Enterprises, LLC	Collierville, TN
9	Topramezone and Triclopyr	Armezon	44.9	BASF	Research Triangle Park, NC
		Trycera	1,120	Helena Agri-Enterprises, LLC	Collierville, TN
10	S-metolachlor, Atrazine, Mesotrione, and Bicyclopyrone	Acuron	2,900	Syngenta Crop Protection	Greensboro, NC
11	Indaziflam	Alion	36.6	Bayer Crop Science	Creve Coeur, MO

Table 3. Visual estimates of percent injury to crop two weeks after herbicide treatment. Letters indicate significant differences ($p < 0.05$) between treatments within a harvest year for each test run per variety.

		L 01-299						HoCP 96-540					
		Plant Cane		1st Ratoon		2nd Ratoon		Plant Cane		1st Ratoon		2nd Ratoon	
Treatment Number	Treatment	Test 1	Test 2	Test 1	Test 2	Test 1	Test 2	Test 1	Test 2	Test 1	Test 2	Test 1	Test 2
1	Topramezone (22.4g)	0 a	4 b	5 bc	7 bc	8 d	2 b	4 ab	0 a	0 a	3 abc	5 b	3 ab
2	Topramezone (56.1g)	0 a	7 abc	6 bc	10 c	8 cd	2 abc	6 ab	0 a	0 a	8 c	7 bc	6 b
3	Clomazone and Diuron	29 b	29 d	36 d	16 c	15 e	19 d	11 b	20 b	19 b	28 d	31 d	14 c
4	Pendimethalin and Atrazine	0 a	8 bc	6 b	9 c	4 bcd	2 abc	3 a	0 a	1 a	2 abc	3 ab	4 b
5	Metribuzin (1680 g)	3 a	10 bc	8 bc	7 bc	5 bcd	3 abc	0 a	0 a	1 a	7 abc	7 c	4 ab
6	Metribuzin (2520 g)	4 a	9 c	10 bc	7 b	6 bcd	3 b	0 a	1 a	0 a	8 abc	6 bc	6 ab
7	Pendimethalin and Metribuzin	4 a	14 c	12 c	10 c	8 cd	5 c	1 a	3 a	0 a	8 bc	11 c	7 b
8	Topramezone (22.4g) and Triclopyr	0 a	7 bc	7 bc	8 bc	5 bc	1 ab	2 a	3 a	0 a	3 abc	6 bc	4 ab
9	Topramezone (44.9 g) and Triclopyr	0 a	5 abc	11 c	8 bc	8 cd	1 ab	3 ab	1 a	1 a	3 b	9 c	6 b
10	S-metolachlor, Atrazine, Mesotrione, and Bicyclopyrone	3 a	6 bc	9 c	9 c	10 de	1 ab	6 ab	0 a	1 a	7 c	8 c	6 b
11	Indaziflam	2 a	5 b	8 bc	5 b	2 ab	0 a	0 a	0 a	0 a	6 a	6 b	1 a
12	Non-treated control	0 a	0 a	0 a	0 a	0 a	0 a	0 a	0 a	0 a	0 a	0 a	0 a

Table 4. Extrapolated sugar yield for plots in kg ha^{-1}. Where there were significant differences ($p < 0.05$) between treatments within harvest year, these differences are indicated by a letter. The absence of a letter means there were no significant differences between treatments for that harvest.

		L 01-299					HoCP 96-540						
		Plant Cane	1st Ratoon		2nd Ratoon		Plant Cane			1st Ratoon		2nd Ratoon	
Treatment Number	Treatment		Test 1	Test 2	Test 1	Test 2	Test 1		Test 2			Test 1	Test 2
1	Topramezone (22.4g)	11440	12110	8620	10030	6670	15640	ab	12190	12150	ab	12110	7470
2	Topramezone (56.1g)	11970	12380	9410	9530	6520	14000	ab	12180	12330	ab	11850	7800
3	Clomazone and Diuron	10490	10770	6840	7780	5840	13790	b	10910	11270	b	10970	7520
4	Pendimethalin and Atrazine	11700	12190	9110	9260	7090	15260	a	12840	11570	b	11680	8130
5	Metribuzin (1680 g)	12090	12510	9880	9080	6900	13930	ab	11780	12380	ab	12110	7790
6	Metribuzin (2520 g)	11910	12130	8710	10880	6520	14540	ab	12330	12100	ab	12060	8140
7	Pendimethalin and Metribuzin	12520	12680	8620	9090	7220	15400	a	12480	12610	ab	11860	8310
8	Topramezone (22.4g) and Triclopyr	12270	11950	8780	9780	7720	15530	a	12160	12870	ab	12000	8310
9	Topramezone (44.9 g) and Triclopyr	11380	12450	8910	9430	7110	15010	ab	11370	12470	ab	11800	8040
10	S-metolachlor, Atrazine, Mesotrione, and Bicyclopyrone	10870	12750	9250	9740	7530	14220	ab	12150	13580	a	11440	7590
11	Indaziflam	12430	12670	9860	10530	7970	15570	ab	10540	12130	ab	11650	8560
12	Non-treated control	12300	11780	9010	9660	7650	14420	ab	11280	12000	ab	11230	7010

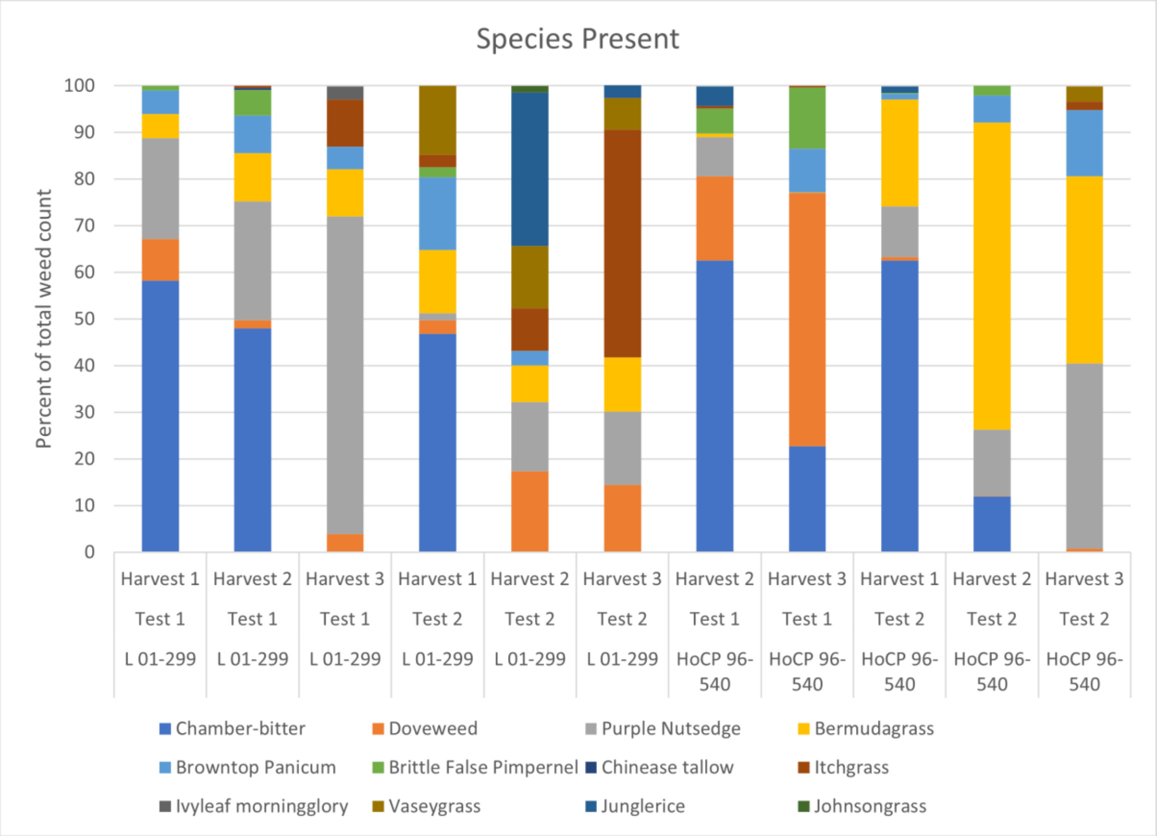

Figure 1. Compilation of weed counts across all treatments for each variety, test, and harvest year. Weed counts were not recorded for Harvest 1 of Test 1 for HoCP 96-540.

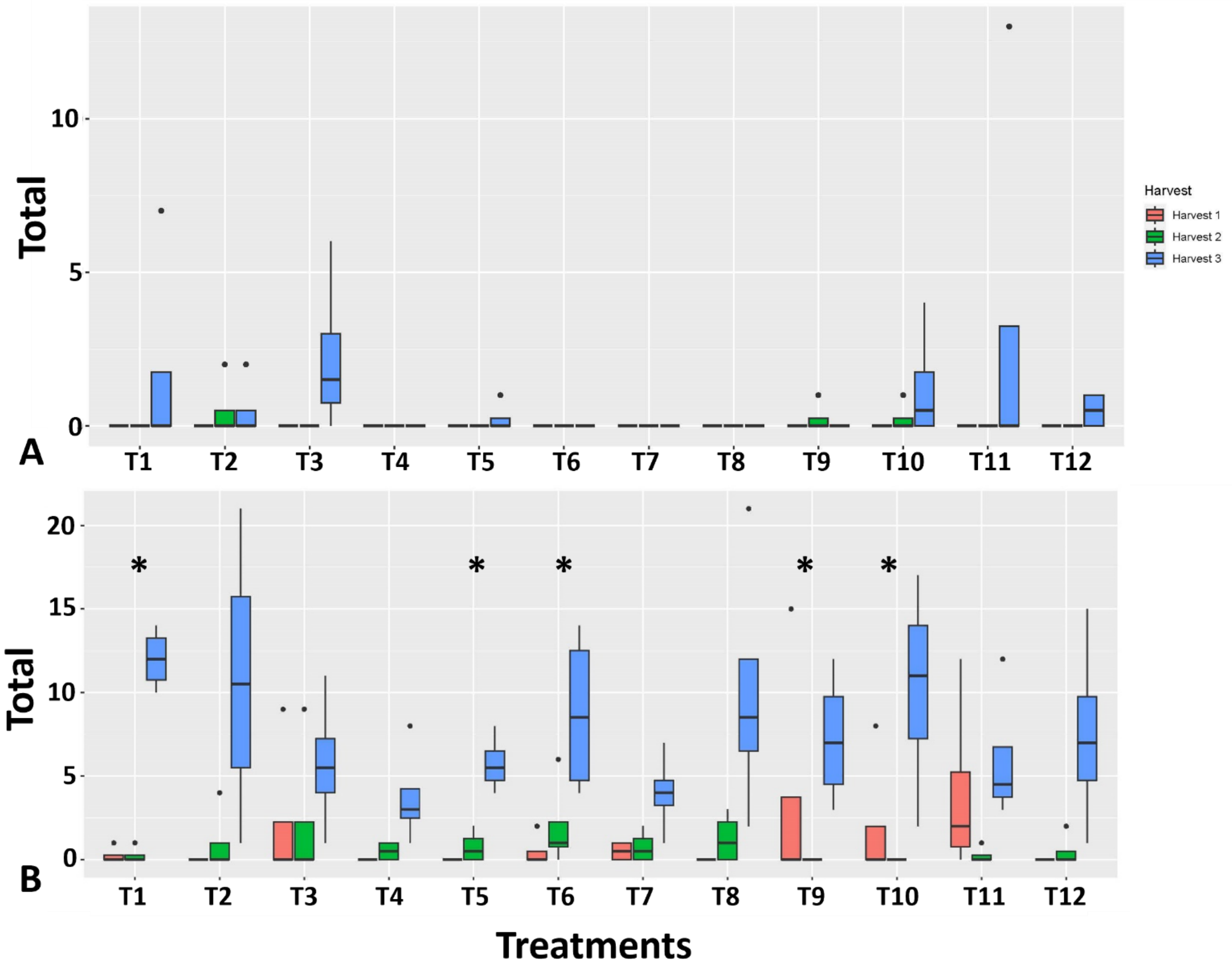

Figure 2. Box plot of itchgrass counts across harvests of L 01-299 for A) Test 1 and B) Test 2. Statistically significant increases are marked with an asterisk. Treatment numbers match those in Table 2.

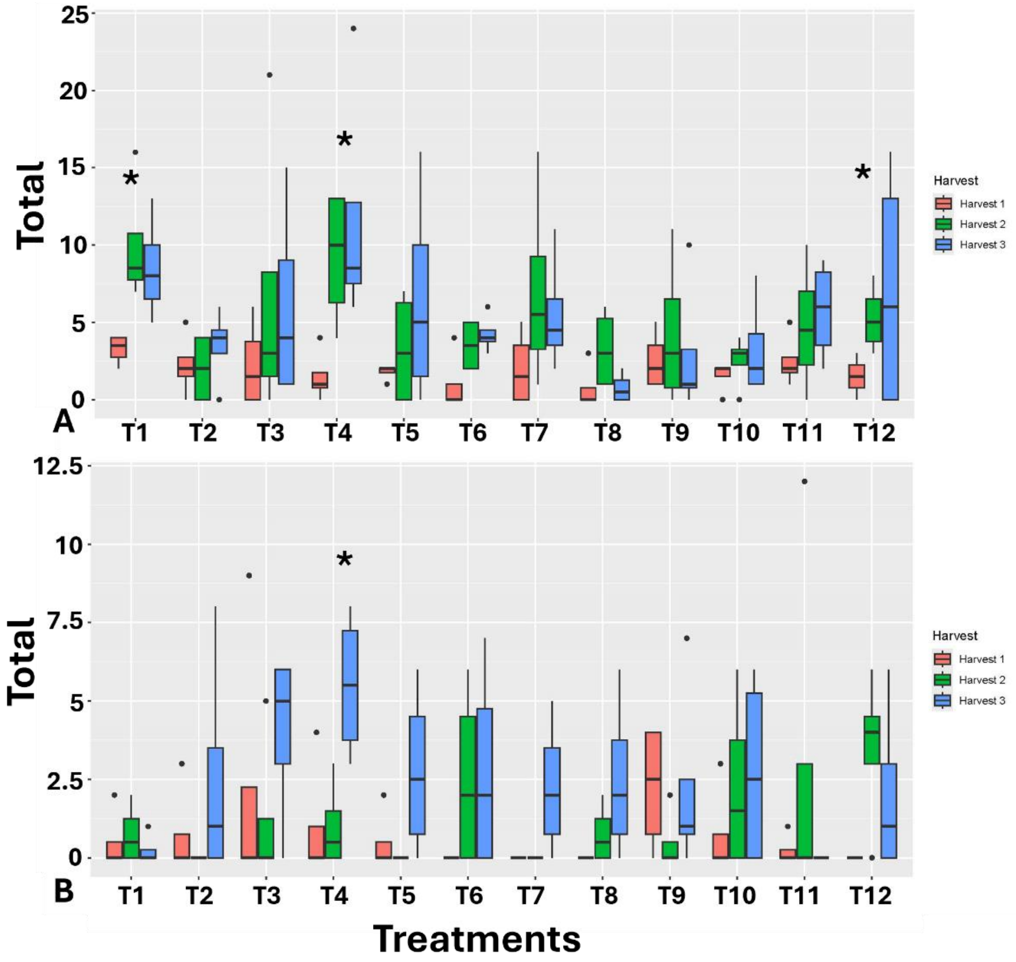

Figure 3. Box plots of purple nutsedge counts across harvest years for L 01-299 A) test 1 and B) test 2. Treatments in which there were significant difference between harvest years are marked with an asterisk. Treatment numbers match those in Table 2.

Table S1

Click here to access/download
Supplemental Material
Table S1.xlsx

Table S2

Click here to access/download
Supplemental Material
Table S2.xlsx
