# Supplementary material for: Dietary fibre intake in the adult Swiss population: a comprehensive analysis of timing and sources
Source: J Nutr Sci. 2025 Mar 24;14:e27. doi: 10.1017/jns.2025.6 (PMC11950702; doi:10.1017/jns.2025.6)
Supplement: von Blumenthal et al. supplementary material 1 — von Blumenthal et al. supplementary material [file S2048679025000060sup001.zip › BJN-2500020.zip!/bjn_2500020_ES/Editing and TypeSetting PO_bjn_2500020.pdf]

Cambridge Journals

26 February

Purchase order for copy editing and setting

Please refer to the proofing instructions for this journal
Proofs MUST be delivered by the time agreed in the service level agreement

article no.	BJN-2024-0762	setters no.	2500020	bjn	0	running order
first named author	Eduarda Silva	author email:	juliana.vaz@gmail.com			
corresp au	Juliana Vaz					
title	Nutrient intake variability and number of days needed to estimate usual intake in children and adolescents with a spectrum disorder					
doi	10.1017/S0007114525000200	no. of book revs				

submission dates	received	revised	accepted
	19/7/24	4/12/24	15/1/25

article type **Research Article**

print colour **no** web colour **no** N/A

copyright line © The Author(s), [YYYY]. Published by Cambridge University Press on behalf of The Nutrition Society

notes for setter: Please note and tag with subject category

supplementary materials Yes Tables with additional analysis

Cambridge Journals

26 February

Purchase order for copy editing and setting

funding body Coordenação de Aperfeiçoamento de Pessoal de Nível author country assoc ed
