# Supplementary material for: Dietary fibre intake in the adult Swiss population: a comprehensive analysis of timing and sources
Source: J Nutr Sci. 2025 Mar 24;14:e27. doi: 10.1017/jns.2025.6 (PMC11950702; doi:10.1017/jns.2025.6)
Supplement: von Blumenthal et al. supplementary material 1 — von Blumenthal et al. supplementary material [file S2048679025000060sup001.zip › BJN-2500020.zip!/bjn_2500020_ES/bjn_2500020_ES/bjn-2024-0762-20250206173532.zip!/bjn-2024-0762-20250206173532/doc/Manuscript (2) 23.1.25.docx]

***Research Article***

**Nutrient intake variability and number of days needed to estimate usual intake in children and adolescents with autism spectrum disorder**

***Nutrient variability in patients with autism***

Eduarda Silva^1^; Laura Hoffmann¹; Kamila Castro¹^,^²; Eliseu Verly Junior³; Sandra Valle^1,4^; Juliana Vaz^1,4^

^1^Programa de Pós-Graduação em Nutrição e Alimentos, Universidade Federal de Pelotas – Pelotas (RS), Brasil.

^2^Serviço de Neuropediatria do Hospital de Clínicas de Porto Alegre - Porto Alegre (RS), Brasil.

^3^Departamento de Epidemiologia, Instituto de Medicina Social, Universidade do Estado do Rio de Janeiro - Rio de Janeiro (RJ), Brasil.

^4^Faculdade de Nutrição, Universidade Federal de Pelotas - Pelotas (RS), Brasil.

Key words: Autism Spectrum Disorder: Dietary Intake: Between-Person Variation: Within-Person Variation

**Abbreviations:** *ASD* Autism spectrum disorder, *24HR* 24-hour food recall, *FR* Foor record, *FFQ* Food frequency questionnaire, *MSM* Multiple source method, *VR* Variance ratio

Correspondence concerning this article should be addressed to Juliana dos Santos Vaz, PhD.

Email: juliana.vaz@gmail.com

Telephone: +55 (53) 32843835

Mailing address: Rua Gomes Carneiro, 01. Centro, Pelotas, RS, Brasil. CEP 96010-610. Sala 228, Bloco A.

**Abstract**

Children and adolescents diagnosed with autism spectrum disorder (ASD) present feeding problems that may influence food acceptance and refusal. However, data regarding dietary intake variability in this population are either not available in the literature or not well-known. This cross-sectional study aimed to identify the within-person and between-person variability, and the number of days needed for a 24-hour recall (24HR) to estimate the usual intake of children and adolescents with ASD. Data were collected from 284 patients assisted at a public neuropediatric health service in the city of Pelotas, Southern Brazil. Food consumption was assessed using three non-consecutive 24HR. Within-person (S²_w_) and between-person (S²_b_) variances, the variance ratio (VR), and the distribution of energy, macronutrients, and micronutrients were obtained using the Multiple Source Method® (version 1.0.1). The number of days of 24HR needed was calculated for correlation coefficients of 0.7, 0.8, and 0.9. For most nutrients, S²_w_ was greater than S²_b_, resulting in RV>1, except for age-group analyses where children up to 5 years old showed VR<1. Two to three days of 24HR were estimated for most nutrients, considering a correlation coefficient of 0.8. Differences were observed according to sex and age group, with adolescents requiring more days of 24HR. These findings indicate the need to assess dietary variability among individuals with ASD according to the characteristics of this investigated population.

Key words: Autism Spectrum Disorder: Dietary Intake: Between-Person Variation: Within-Person Variation

**Introduction**

Autism spectrum disorder (ASD) is a neurodevelopmental disorder associated with difficulties in social interaction and communication, repetitive behaviour, and restrictive interests^(1)^. Studies investigating nutritional aspects in ASD have found a high prevalence of feeding difficulties, which are associated with different factors, such as feeding behaviour problems and sensory processing alterations^(2–5)^. These factors result in selective or excessive food consumption, impacting daily dietary repertoire and energy and nutrient intake^(6,7)^.

In studies assessing food consumption, estimating usual intake (i.e. intake over a long period) of energy and nutrients is essential for investigating associations between diet and health. However, the method of data collection and day-to-day variability in consumption must be considered^(8)^. A recent systematic review highlighted the need for more detailed information on the methods used to assess dietary intake in individuals with ASD, as well as the inclusion of adjustments for estimating usual intake in their analyses^(9)^.

Understanding the variation in food consumption from day-to-day for each individual (within-person variability) and between individuals (between-person variability) is required to obtain the necessary adjustments in analyses aimed at identifying the prevalence of inadequacies in intake^(8,10,11)^. Studies have shown that the variability of energy, macronutrient, and micronutrient intake varies according to age, sex, and country^(12–14)^.

Food frequency questionnaires (FFQ), 24-hour recalls (24HR), and food records (FR) are the most used dietary instruments for assessing usual intake. All these instruments have strengths and biases discussed in the literature^(8)^. Short-term instruments, such as the 24HR and FR, when applied correctly, have a lower systematic error. However, they do not capture within-person variation and do not allow the usual intake to be estimated. Also, although this limitation may be mitigated by repeating the application of the instrument over several days, the number of repetitions required differs according to within and between-person variances, which are inherent to the nutrients of interest in the study population^(8,10,15)^.

The number of studies investigating intake variability in typically developing children and adolescents is relatively limited, and until now, no study has reported these findings for individuals with ASD^(12,14–16)^. The particularities of the diagnostic and difficulties associated with eating in ASD directly affect food acceptance and refusal and, consequently, may contribute to lower dietary variability in these individuals^(4,17,18)^. The absence of data on dietary intake variability in this population represents a significant gap in the design of studies involving the collection and interpretation of dietary data. Additionally, factors including the heterogeneity observed among individuals with ASD, the higher prevalence of boys in the samples, and the different age groups are rarely considered in analyses of nutritional studies. As a result, the impact of these characteristics on the variation in food consumption remains unclear^(19,20)^.

In this context, the main objective of this study was to estimate the within-person and between-person variability and the number of days of 24HR needed to estimate the usual intake of energy and nutrients in a sample of children and adolescents with ASD from southern Brazil. To our knowledge, this study constitutes the first investigation on nutrient variability within a population of children and adolescents with autism. Examining these aspects of dietary intake offers novel insights into our understanding of dietary aspects in this group, enriching the existing body of knowledge on nutrient variability and dietary assessment methods.

**Methods**

***Design and study sample***

A cross-sectional study was conducted with baseline data collected between July 2021 and April 2024, from a larger study with patients diagnosed with ASD from 2 to under 19 years, who were assisted at the public neuropediatric health service of a public university in the city of Pelotas, Southern Brazil. Two paediatric neurology professors, responsible for periodic care at this service, conducted the diagnosis of ASD. Out of 311 participants, 24 were excluded from the analyses for using dietary supplements, and another 3 were excluded due to missing dietary data, resulting in a final sample of 284.

***Data collection and study variables***

Data were collected in three interviews carried out by research nutritionists trained for this study. A standard questionnaire including sociodemographic, educational, clinical, and anthropometric questions was applied during previously scheduled face-to-face interviews.

The caregivers were asked to answer three non-consecutive days of the 24HR, with a one-week interval between each collection, regarding the dietary intake of the subject under assessment. The first and third R24H were administered face-to-face and covered weekdays. The second recall was collected by telephone interview and corresponded to a weekend day. Caregivers were instructed to report all foods and beverages consumed the day before the interviews, including meal and snack times and quantities consumed.

To improve data quality, the multi-pass method was used during the application of the 24HR^(21)^. In addition, a photo book of commonly used measurements was used to support the reporting of portions^(22)^. Regarding school meals, caregivers provided information about the food sent from home for consumption at school, as well as any leftovers. A total of 232 caregivers completed three days of 24HR at the end of the study, while 36 completed two days and 16 completed one day. The energy, macronutrient, micronutrient and fiber composition of the reported foods was calculated using the Brazilian Table of Food Composition (TBCA, version 7.2, Universidade de São Paulo, Food Research Center, 2023)^(23)^.

The anthropometric data of weight (Kg) and height (cm) were measured using a digital scale with a capacity of 150 kg and accuracy of 100 g (TRENTIN, RS, Brazil) and a stadiometer coupled to the scale (maximum measurement of 213 cm and accuracy of 0.1 cm). For children with height under 100 cm, a horizontal stadiometer was used (maximum measurement of 100 cm and accuracy of 0.1 cm). These data were used to evaluate nutritional status using the Z-score of body mass index for age, and overweight was assessed according to the cutoff of +1 Z-score, established by the World Health Organization^(24,25)^.

The following variables were used to describe the sample: age categorized by years (2-5, 6-9, 10-18); sex (female, male); skin colour (white, black, brown, or indigenous); per capita family income (US$) (< ½ , ½ - <1, ≥1 minimum wage), considering the Brazilian minimum wage according to the years of data collection; primary caregiver (mother, others); primary caregiver's years of schooling (≤8, ≥9); use of antipsychotic medication (yes, no) and overweight (yes, no).

***Data analysis***

The data were double entered into the EpiData® version 3.1 program (EpiData, version 3.1; The EpiData Association, Odense, Denmark, 2003-2005). All data were imported to STATA® statistical software version 15.1 (Stata Statistical Software, version 15.1. College Station, Texas, United States of America) for analysis.

The presence of outliers in energy and nutrient estimates was investigated by graphical visualization of the distribution and review of extreme values for each dietary variable. The identified inconsistencies, primarily related to errors in household measurements, were addressed and corrected without excluding any participants.

The standardized within-person (S²_w_) and between-person (S²_b_) variances, variance ratio (VR = S²_w_ /S²_b_), the distribution of the measured intake, and the usual intake of energy and nutrients were obtained through analyses conducted in the Multiple Source Method ® version 1.0.1 (MSM) web application (Department of Epidemiology at the German Institute of Human Nutrition Potsdam-Rehbrücke) for each dietary variable investigated^(26)^*.* MSM is a statistical method developed to remove within-person random error in estimates of usual consumption distribution from data collected by short-term dietary assessment instruments. Details of the method are available in the publication by Haubrock *et al.*^(27)^.

The equation proposed by Black *et al*.^(28)^ was used to estimate the number of days needed to estimate usual intake: d = (r²/1-r²) x (VR) (equation 1),

where *d* is the number of days, *r* is the correlation coefficient required between measured intake and usual intake, and *VR* corresponds to the ratio between S²_w_ and S²_b_. Correlation coefficients of 0.7, 0.8, and 0.9 were considered acknowledging that the higher the r, the greater the correlation between the information collected and usual intake^(15,29)^.

The analyses were stratified according to age group and sex.

***Ethical considerations***

This study was conducted according to the guidelines laid down in the Declaration of Helsinki and all procedures involving human subjects were approved by the Research Ethics Committee of the Faculty of Medicine of the Federal University of Pelotas (CAEE: 94253518.0.0000.5317). Written informed consent was obtained from all those responsible for the participants.

**Results**

The mean age of the participants was 7.4 (SD 3.5) years, and 75.7% were children between 2 and 9 years old. Most of participants were male (82.8%), white (77.8%), and from families with a per capita income of under US$247.00 (88.4%). Almost half were using antipsychotic drugs (54.2%) and 66.7% were overweight. Approximately 73% of caregivers had 9 years or more of schooling and, for the greater part of the participants, mothers were the main caregivers (91.9%) (Table 1).

***Distribution of energy and nutrient intake***

Table 2 shows the distribution of energy and nutrient intakes according to the crude and usual estimates. The mean intakes were similar in both estimates for all dietary variables, with a reduction in the standard deviation and differences in the distribution of intakes. In the analyses stratified by age group and sex, higher mean intakes were observed in the groups of boys and the older age group (10-18 years) for most of the nutrients (Supplementary material 1 and 2).

***Variance components and days required for calculating usual intakes***

In general, S²_w_ was higher than between-person variance (S²_b_) for most of the dietary variables, resulting in VR values of >1. Exceptions were observed for fiber, retinol, riboflavin, calcium, iron, phosphorus, and copper (RV<1). The highest VR values were observed for cholesterol (1.64), monosaturated fatty acids (1.59), and saturated fatty acids (1.56), while the lowest values were obtained for calcium (0.52) and riboflavin (0.79) (Figure 1). The number of days of 24HR required to estimate usual intake, considering the correlation coefficient of 0.8, ranged from two to three for most nutrients, except calcium and riboflavin, for which a single day may be sufficient for obtaining an estimate of usual intake. For the 0.7 and 0.9 correlation coefficients, the range varied from one to two days, and two to seven days, respectively (Table 3).

Analyses conducted with male participants showed similar results to the total sample, with VR≥1 for most of the variables. For girls, the VR was predominantly <1. There were differences between sexes in the values of S²w, S²b, and VR; however, no pattern was observed directed at a specific sex. The group of girls, compared to the boys, had lower VR values for most of the dietary variables. Higher VR values were obtained for saturated fatty acids (1.86), monosaturated fatty acids (1.72), and potassium (1.71) for boys, and for niacin (2.27), cholesterol (2.07), and zinc (1.29) in the group of girls (Supplementary material 3). Based on the correlation coefficient of 0.8, most of the variables required more days of 24HR in the group of boys (one to three days) compared to the group of girls (one to four days) (Table 4).

There were differences in S²_w_ and S²_b_ values between age groups, with higher VR values for most of the variables in 10 to 18 age group (0.93 - 3.69), except for lipids, saturated fatty acids, monosaturated fatty acids, thiamine, niacin, phosphorus, sodium, and potassium, whose VR values were higher in the 6 to 9 age group (0.82 - 3.61). For the group between 2 and 5 years old, the VR ranged from 0.38 to 1.34 and the S²_b_ values were higher than the S²w values for 15 of the 23 variables analysed (VR<1), with the exception of proteins, monosaturated fatty acids, polyunsaturated fatty acids, cholesterol, niacin, vitamin C, sodium and zinc. In the other age groups, VR>1 was obtained for almost all the nutrients; however, the opposite was observed for retinol, vitamin C, calcium, and iron for children between 6 and 9 years old, and only for calcium in the older age group (Table 5) (Supplementary material 4).

A correlation coefficient of 0.8 indicates that one to two days of 24HR were needed to estimate usual intake in children aged 2 to 5 years, while one to six days were required for older children (6-9 years). In the group of adolescents (10-18 years), the range of days, using the same coefficient, varied from two to seven days (Table 6).

**Discussion**

The present study had three main findings. Firstly, it was identified that children and adolescents with ASD have higher S²_w_ values for most dietary variables. The second finding indicated differences in S²_w_ and S²_b_ for different nutrients when stratified by sex and age group, with higher S²_b_ values observed in children up to 5 years old and an increase in S²_w_ for older children and adolescents. However, it was not possible to identify the direction of these alterations according to sex. Finally, based on the obtained VRs, two to three days of 24HR are sufficient to obtain food consumption with a good correlation with usual intake (0.8). To our knowledge, this is the first study to examine nutrient variability and the number of days needed to estimate the usual intake of children and adolescents with autism, and to provide evidence on how sex and age group affect the variance.

Previous studies have identified S²_w_ and S²_b_ variability of energy and nutrients in samples of typically developing children and adolescents in different countries, with observed VR values above and below one^(10,12,13,15,30,31)^. In the current study, S²_w_ was higher than S²_b_ for most nutrients, in analyses not stratified by sex or age. Additionally, the VR values obtained in our sample were lower than those observed in other studies^(12,29)^. Several factors may contribute to these results. We believe that behavioural fluctuations, medication use, and variations in the quantities of food consumed, despite a restricted feeding repertoire, could reflect a higher S²_w_.

The assessment of food consumption during childhood presents challenges due to the use of reporting by caregivers and day-to-day variation, as quantity and variety of foods consumed change throughout different stages of growth and development^(14,32)^. In our sample, different S²_w_ and S²_b_ values were obtained according to age group, with higher S²_b_ values in the 2- to 5-year-old group and increased S²_w_ in subsequent age groups. These findings may be explained by the reduced variety of foods consumed by children and the increase of the food repertoire as age progresses^(30,33,34)^. Furthermore, evidence indicates that individuals with ASD often have more limited food repertoires compared to control groups^4,6,36^. However, studies on feeding difficulties in ASD have not comprehensively investigated the influence of age on food refusal^(4,17,36)^.

The influence of age on the increase in VR values in typically developed children has been previously observed in the literature^(10,13,14,30,31)^. However, discrepant results compared to our study were reported by Ollberding *et al.*^(16)^, who found that the highest VR values were observed in the group of children (6 to 11 years), compared to the group of adolescents (12 to 17 years). In Brazil, studies have been carried out on different age groups; however, they are also limited to samples of typically developing children and adolescents and from different regions of the country, primarily from the northeast and southeast. Padilha *et al*.^(15)^ (13 to 32 months) and Salles-Costa *et al*.^(37)^ (6 to 30 months) found VR<1 for most of the nutrients analysed, except for polyunsaturated fatty acids, energy, fiber^(15)^, and vitamin C^(15,37)^. Conversely, a study with preschool children (1 to 6 years) obtained VR>1 values, including data from participants across all five macro-regions of Brazil (south, southeast, central west, north, and northeast)^(14)^. However, the inclusion of a few cities per region affects the representativeness of the data.

As for adolescents, studies conducted in São Paulo and Rio de Janeiro found higher S²_w_ values compared to S²_b_ for most nutrients (VR>1)^(29,38,39)^. Different results were presented in the study by Lima *et al*.^(40)^, carried out with individuals aged 10 to 19 years residing in the city of Natal (northeast Brazil), where higher S²_b_ values were obtained (VR<1).

Our study sample had a higher proportion of boys, consistent with the latest prevalence estimate of ASD from the Centers for Disease Control and Prevention^(41)^. Differences in nutrient intake variability have also been observed in studies with typically developed children and adolescents. Caswell *et al.*^(12)^ found lower VRs in a samples of Zambian girls (4 to 8 years old) compared to boys, corroborating with our findings. In contrast, other studies have reported higher VRs for females when analysing nutrient intake variability^(30,39,40)^. Other studies reported lack of consistent findings that could indicate possible discrepancies in within-person and between-person variances among boys and girls^(13,16,31)^.

The use of within and between-person variance to determine the number of days of 24HR is widely recognised and recommended^(8,28,42)^. Results observed in samples of Brazilian children and adolescents presented intervals ranging from 1 to 16 days for younger children (aged 1 to 6 years)^(14,15,30)^, from 4 to 14 days for older children (6 to 11 years)^(16)^, and 2 to 16 days for adolescents (12 to 18 years)^(16,39)^, for correlation coefficients ≥0.8. Intervals of 1 to 5 days were obtained considering coefficients of 0.9 for samples of children and adolescents^(37,40)^. However, most studies have estimated a greater number of days for coefficients ≥0.9^(14,15,29–31,38,39)^.

In the current study sample, a range of 1 to 3 days of 24HR was required to estimate the intake of most nutrients to obtain correlations of 0.7 and 0.8 with usual intake. In addition, it was highlighted the necessity to consider the characteristics of the target population in estimating the required number of 24HR days. The increase in the number of days, proportional to higher VR values, observed for older children and adolescents, as well as variations between girls and boys in different nutrients, has been previously reported in studies with children and adolescents without a diagnosis of ASD^(30,31,39)^.

Although the application of several days of 24HR increases the precision of food consumption estimates, it results in higher costs and requires greater availability from the interviewees to report the data^(8,34)^. The findings of this study have relevant implications for the design and interpretation of data from research on children and adolescents with ASD. Considering that validation studies of FFQs developed for this age group generally found correlations ≤0.7, the results indicate that 1 to 2 days of 24HR may provide similar estimates to those obtained with FFQs commonly used in epidemiological studies with samples with and without ASD diagnoses^(43–45)^.

The presented results provide support for the planning of methodologies for dietary data collection for children and adolescents with ASD. It also provides data for adjustments in analyses of the distribution and adequacy of food consumption in this population. The use of external dietary variability data is supported by previous studies, which indicate the applicability of previously collected values of S²_w_ in order to correct estimates of the distribution of usual intake in studies with only one day of 24HR, on the condition that these data come from a sample with characteristics similar to those of the investigated population^(11,46)^. However, the application of repeated 24HR is still the most recommended method^(47,48)^.

Our study has limitations inherent to the dietary method applied, such as the potential for underestimation or overestimation of food portions which can impact dietary intake estimates. To minimize this bias, we used a photo book of household measurements, and all nutritionists were trained to identify potential inconsistencies during the 24HR interviews. Additionally, sample restrictions should be considered, as the results were derived from children and adolescents attending a public health service in a city in Southern Brazil, which restricts the external validity of the study. Therefore, the interpretation and use of current findings require caution, as dietary habits and variability are influenced by multiple sociodemographic factors and may differ between populations^(8)^. Furthermore, there is a need for similar investigations with the adult population with ASD, as evidence suggests the presence of feeding problems in this age group as well^(49)^.

**Conclusion**

In this study, the within-personal variance was predominantly higher than the between-person variance for most of the nutrients analysed, resulting in estimates of two to three days of 24HR needed to assess usual intake, considering the correlation coefficient of 0.8. The findings differed according to age and sex, suggesting the importance of including these aspects in the design and interpretation of results related to food consumption in ASD. Future studies may analyse how socioeconomic characteristics, behavioural, and clinical factors may influence food consumption and variability of nutrient intake in individuals with ASD.

**Acknowledgments**

The authors thank the families who took part in the study, staff, and personnel for their assistance in the recruitment and schedule the appointments. The authors acknowledge the essential work and dedication of all research assistants during the data collection of this study.

**Financial support**

This study was financed in part by the Coordination for the Improvement of Higher Education Personnel (CAPES) - Finance Code 001, and the Brazilian National Research Council (CNPq) - Finance Code 308213/2021–1 and 407237/2021–6.

**Conflict of interest**

The authors declare that there are no conflicts of interest.

**Authorship**

The author's responsibilities were as follows: E.S. participated in the designing of the study, statistical analysis, interpreting of data, and writing the manuscript. E.V.J. supported the plan of statistical analysis and interpretation of data. L.H., K.C., S.V., and J.V. participated in designing the study and interpretation of data. All authors contributed to the writing or revising of the manuscript and approved the final version.

**REFERENCES**

1.	American Psychiatric Association (2022) Diagnostic and Statistical Manual of Mental Disorders, Fifth Edition, Text Revision (DSM-5-TR). 5th ed. Washington, D.C: American Psychiatric Association.

2.	Leader G, Tuohy E, Chen JL *et al.* (2020) Feeding Problems, Gastrointestinal Symptoms, Challenging Behavior and Sensory Issues in Children and Adolescents with Autism Spectrum Disorder. *J Autism Dev Disord* **50**, 1401–10.

3.	Marí-Bauset S, Zazpe I, Mari-Sanchis A *et al.* (2014) Food selectivity in autism spectrum disorders: a systematic review. *J Child Neurol* **29**, 1554–61.

4.	Chistol LT, Bandini LG, Must A *et al.* (2017) Sensory Sensitivity and Food Selectivity in Children with Autism Spectrum Disorder. *Journal of Autism and Developmental* *Disorders* **48**, 583–91.

5.	Alibrandi A, Zirilli A, Loschiavo F *et al.* (2023) Food Selectivity in Children with Autism Spectrum Disorder: A Statistical Analysis in Southern Italy. *Children (Basel)* **10**, 1553.

6.	Castro K, Faccioli LS, Baronio D *et al.* (2016) Feeding behavior and dietary intake of male children and adolescents with autism spectrum disorder: A case-control study. *Int J Dev Neurosci* **53**, 68–74.

7.	Nadeau MV, Richard E, Wallace GL (2022) The Combination of Food Approach and Food Avoidant Behaviors in Children with Autism Spectrum Disorder: ‘Selective Overeating’. *J Autism Dev Disord* **52**, 987–94.

8.	Willett W (2012) Nutritional Epidemiology. Third Edition. Oxford, New York: Oxford University Press.

9.	de Souza Silva E, Castro K, Valle SC *et al.* (2023) Dietary Assessment Methods Applied in Clinical and Epidemiological Studies in Children and Adolescents with Autism Spectrum Disorder: a Systematic Review. *Rev J Autism Dev Disord*. Published online: 9 January 2023. DOI: 10.1007/s40489-022-00353-3

10.	Nel JH, Steyn NP, Senekal M (2022) Illustration of the Importance of Adjustment for within- and between-Person Variability in Dietary Intake Surveys for Assessment of Population Risk of Micronutrient Deficiency/Excess Using an Example Data Set. *Nutrients* **14**.

11.	Verly Junior E, Cesar CLG, Fisberg RM *et al.* (2013) Variância intrapessoal da ingestão de energia e nutrientes em adolescentes: correção de dados em estudos epidemiológicos. *Rev bras epidemiol* **16**, 170–7.

12.	Caswell BL, Talegawkar SA, Siamusantu W *et al.* (2020) Within-person, between-person and seasonal variance in nutrient intakes among 4- to 8-year-old rural Zambian children. *Br J Nutr* **123**, 1426–33.

13.	Jahns L, Carriquiry A, Arab L *et al.* (2004) Within- and between-person variation in nutrient intakes of Russian and U.S. children differs by sex and age. *J Nutr* **134**, 3114–20.

14.	de Castro MA, Verly EJ, Fisberg M *et al.* (2014) Children’s nutrient intake variability is affected by age and body weight status according to results from a Brazilian multicenter study. *Nutr Res* **34**, 74–84.

15.	Padilha LL, França AKTDC, da Conceição SIO *et al.* (2017) Nutrient intake variability and the number of days needed to estimate usual intake in children aged 13-32 months. *Br J Nutr* **117**, 287–94.

16.	Ollberding NJ, Couch SC, Woo JG *et al.* (2014). Within- and between-individual variation in nutrient intake in children and adolescents. *J Acad Nutr Diet* **114**, 1749-1758.e5.

17.	Bandini LG, Curtin C, Phillips S *et al.* (2017) Changes in Food Selectivity in Children with Autism Spectrum Disorder. *J Autism Dev Disord* **47**, 439–46.

18.	Sharp WG, Postorino V, McCracken CE *et al.* (2018) Dietary Intake, Nutrient Status, and Growth Parameters in Children with Autism Spectrum Disorder and Severe Food Selectivity: An Electronic Medical Record Review. *J Acad Nutr Diet* **118**, 1943–50.

19.	Bicer AH, Alsaffar AA (2013) Body mass index, dietary intake and feeding problems of Turkish children with autism spectrum disorder (ASD). *Res Dev Disabil* **34**, 3978–87.

20.	Tsujiguchi H, Miyagi S, Nguyen TTT *et al.* (2020) Relationship between Autistic Traits and Nutrient Intake among Japanese Children and Adolescents. *Nutrients* **12**.

21.	Johnson RK, Driscoll P, Goran MI (1996) Comparison of multiple-pass 24-hour recall estimates of energy intake with total energy expenditure determined by the doubly labeled water method in young children. *J Am Diet Assoc* **96**, 1140–4.

22.	Vitolo MR (2014) Nutrição – da Gestação ao Envelhecimento. Editora Rubio.

23.	Tabela Brasileira de Composição de Alimentos (TBCA), versão 7.2 (2023).

24. World Health Organization (2006) WHO child growth standards: length/height-for-age, weight-for-age, weight-for-length, weight-for-height and body mass index-for-age: methods and development. Published online. Available from: https://www.who.int/publications/i/item/924154693X

25.	de Onis M, Onyango AW, Borghi E *et al.* (2007) Development of a WHO growth reference for school-aged children and adolescents. *Bull World Health Organ*. **85**, 660–7.

26.	Harttig U, Haubrock J, Knüppel S *et al.* (2011) The MSM program: web-based statistics package for estimating usual dietary intake using the Multiple Source Method. *Eur J Clin Nutr* **65**, S87–91.

27.	Haubrock J, Nöthlings U, Volatier JL *et al.* (2011) Estimating Usual Food Intake Distributions by Using the Multiple Source Method in the EPIC-Potsdam Calibration Study1–3. *The Journal of Nutrition* **141**, 914–20.

28.	Black AE, Cole TJ, Wiles SJ *et al.* (1983) Daily variation in food intake of infants from 2 to 18 months. *Hum Nutr Appl Nutr* **37**, 448–58.

29.	Verly Junior E, Fisberg RM, Cesar CLG *et al.* (2010) Sources of variation of energy and nutrient intake among adolescents in São Paulo, Brazil. *Cad saúde pública*. **26**, 2129–37.

30.	Erkkola M, Kyttälä P, Takkinen HM *et al.* (2011) Nutrient intake variability and number of days needed to assess intake in preschool children. *Br J Nutr* **106**, 130–40.

31.	Huybrechts I, De Bacquer D, Cox B *et al.* (2008) Variation in energy and nutrient intakes among pre-school children: implications for study design. *European journal of public health* **18**, 509–16.

32.	Livingstone MBE, Robson PJ, Wallace JMW (2004) Issues in dietary intake assessment of children and adolescents. *Br J Nutr* **92** Suppl 2, S213-222.

33.	Cooke LJ, Wardle J (2005) Age and gender differences in children’s food preferences. *Br J Nutr* **93**, 741–6.

34.	Lanigan JA, Wells JCK, Lawson MS *et al.* (2004) Number of days needed to assess energy and nutrient intake in infants and young children between 6 months and 2 years of age. *Eur J Clin Nutr* **58**, 745–50.

35.	Bandini L, Anderson S, Curtin C *et al.* (2010) Food Selectivity in Children with Autism Spectrum Disorders and Typically Developing Children. *Journal of Pediatrics* **157**, 259–64.

36.	Curtin C, Hubbard K, Anderson SE *et al.* (2015) Food selectivity, mealtime behavior problems, spousal stress, and family food choices in children with and without autism spectrum disorder. *Journal of Autism and Developmental Disorders* **45**, 3308–15.

37.	Salles-Costa R, Barroso GDS, Mello MA de *et al.* (2010) Sources of variation in energy and nutrient intakes among children from six to thirty months old in a population-based study. *Cad Saude Publica* **26**, 1175–86.

38.	Costa M de MF, Takeyama L, Voci SM *et al.* (2008) Within- and between-person variations as determinant factors to calculate the number of observations to estimate usual dietary intake of adolescents. *Rev bras epidemiol* **11**, 541–8.

39.	Pereira RA, Araujo MC, Lopes T de S *et al.* (2010) How many 24-hour recalls or food records are required to estimate usual energy and nutrient intake? *Cad Saúde Pública* **26**, 2101–11.

40.	Lima SCVC, Lyra CO, Sena-Evangelista KCM *et al.* (2013) Variance sources and ratios to estimate energy and nutrient intakes in a sample of adolescents from public schools, Natal, Brazil. *Rev nutr* **26**, 159–66.

41.	Maenner MJ, Warren Z, Williams AR *et al.* (2023) Prevalence and Characteristics of Autism Spectrum Disorder Among Children Aged 8 Years — Autism and Developmental Disabilities Monitoring Network, 11 Sites, United States, 2020. *MMWR Surveill Summ* **72**, 1-14.

42.	Nelson M, Black AE, Morris JA *et al.* (1989) Between- and within-subject variation in nutrient intake from infancy to old age: estimating the number of days required to rank dietary intakes with desired precision. *Am J Clin Nutr* **50**, 155–67.

43.	Rockett HR, Breitenbach M, Frazier AL *et al.* (1997) Validation of a youth/adolescent food frequency questionnaire. *Prev Med* **26**, 808–16.

44.	Cullen KW, Watson K, Zakeri I (2008) Relative reliability and validity of the Block Kids Questionnaire among youth aged 10 to 17 years. *J Am Diet Assoc* **108**, 862–6.

45.	Saravia L, Miguel-Berges ML, Iglesia I *et al.* (2021) Relative validity of FFQ to assess food items, energy, macronutrient and micronutrient intake in children and adolescents: a systematic review with meta-analysis. *British Journal of Nutrition* **125**, 792–818.

46.	Jahns L, Arab L, Carriquiry A *et al.* (2005) The use of external within-person variance estimates to adjust nutrient intake distributions over time and across populations. *Public Health Nutr* **8**, 69–76.

47.	Luo H, Dodd KW, Arnold CD *et al.* (2019) A New Statistical Method for Estimating Usual Intakes of Nearly-Daily Consumed Foods and Nutrients Through Use of Only One 24-hour Dietary Recall. *J Nutr* **149**, 1667–73.

48.	French CD, Arsenault JE, Arnold CD *et al.* (2021) Within-Person Variation in Nutrient Intakes across Populations and Settings: Implications for the Use of External Estimates in Modeling Usual Nutrient Intake Distributions. *Advances in Nutrition* **12**, 429–51.

49.	Demartini B, Nisticò V, Bertino V *et al.* (2021) Eating disturbances in adults with autism spectrum disorder without intellectual disabilities. *Autism Res* **14**, 1434–43.

**Figure 1.** Variance ratio of energy, macronutrients, and micronutrients in children and adolescents with autism spectrum disorder. Pelotas, Brazil, PANA Study (n=284)

**Supplementary material 3.** Variance ratio of energy, macronutrients, and micronutrients according to sex of children and adolescents with autism spectrum disorder. Pelotas, Brazil, PANA Study (n=284)

**Supplementary material 4.** Variance ratio of energy, macronutrients, and micronutrients according to age group of children and adolescents with autism spectrum disorder. Pelotas, Brazil, PANA Study (n=284)

| **Table 1**. Socioeconomic and demographic characteristics of children and adolescents with autism spectrum disorder. Pelotas, Brazil, PANA Study (n=284) | | |
| --- | --- | --- |
| **Variables** | **n** | **%** |
| *Age (Years)* |  |  |
| 2 - 5 | 118 | 41.6% |
| 6 - 9 | 97 | 34.1% |
| 10-18 | 69 | 24.3% |
| *Sex* |  |  |
| Male | 235 | 82.8% |
| *Skin colour* |  |  |
| White | 221 | 77.8% |
| Black, indigenous, or mixed race | 63 | 22.2% |
| *Primary caregiver* |  |  |
| Mother | 261 | 91.9% |
| Others* | 23 | 8.1% |
| *Primary caregiver's education (years)^†^* |  |  |
| ≤ 8 | 77 | 27.4% |
| ≥ 9 | 204 | 72.6% |
| *Family income per capita (US$)* |  |  |
| < 123,52 | 132 | 46.5% |
| 123,52 a < 247,00 | 119 | 41.9% |
| ≥ 247,00 | 33 | 11.6% |
| *Use of antipsychotic medication* |  |  |
| Yes | 154 | 54.2% |
| *Overweight*^‡^ |  |  |
| Yes | 176 | 66.7% |
| *Other caregivers include fathers, grandparents, foster parents, and other family members.  ^†^Missing data for 3 participants.  ^‡^Assessed based on body mass index for age. Missing data for 20 participants. | | |

| **Table 2.** Distribution of crude and usual estimates of energy, macronutrient, and micronutrient intake in children and adolescents with autism spectrum disorder. Pelotas, Brazil, PANA Study (n=284) | | | | | | | | | | | |
| --- | --- | --- | --- | --- | --- | --- | --- | --- | --- | --- | --- |
| **Variables** | **Estimated intake** | | | | |  | **Usual intake** | | | | |
|  | **Mean** | **SD** | **P25** | **P50** | **P75** |  | **Mean** | **SD** | **P25** | **P50** | **P75** |
| Energy (Kcal) | 1781.2 | 571.5 | 1367.1 | 1726.7 | 2146.8 |  | 1782.7 | 480.8 | 1434.9 | 1742.4 | 2080.0 |
| Carbohydrates (g) | 248.4 | 87.4 | 189.5 | 239.7 | 305.4 |  | 248.6 | 73.9 | 195.8 | 243.8 | 298.1 |
| Proteins (g) | 59.2 | 21.7 | 43.2 | 57.3 | 69.4 |  | 59.3 | 17.8 | 46.6 | 58.4 | 69.1 |
| Lipids (g) | 61.2 | 26.5 | 40.9 | 55.8 | 75.4 |  | 61.4 | 21.3 | 45.6 | 57.6 | 72.9 |
| Saturated fatty acids (g) | 21.5 | 9.1 | 14.8 | 20.0 | 27.0 |  | 21.5 | 7.2 | 16.4 | 20.5 | 25.6 |
| Monosaturated fatty acids (g) | 18.8 | 9.2 | 11.7 | 16.7 | 23.7 |  | 18.8 | 7.1 | 13.5 | 17.7 | 23.0 |
| Polyunsaturated fatty acid (g) | 13.4 | 8.0 | 8.1 | 11.6 | 17.0 |  | 13.5 | 6.7 | 8.8 | 11.9 | 16.9 |
| Cholesterol (mg) | 192.6 | 121.7 | 103.4 | 166.4 | 258.8 |  | 193.4 | 97.9 | 123.3 | 174.6 | 252.3 |
| Fiber (g) | 16.5 | 9.1 | 9.8 | 14.4 | 21.5 |  | 16.6 | 7.9 | 11.0 | 15.3 | 20.2 |
| Retinol (mcg) | 310.5 | 272.3 | 137.3 | 264.5 | 399.9 |  | 305.4 | 199.8 | 161.7 | 280.3 | 410.7 |
| Thiamine (mg) | 1.1 | 0.5 | 0.8 | 1.1 | 1.4 |  | 1.1 | 0.4 | 0.9 | 1.1 | 1.4 |
| Riboflavin (mg) | 1.5 | 0.8 | 1.1 | 1.4 | 2.0 |  | 1.5 | 0.7 | 1.1 | 1.5 | 1.9 |
| Pyridoxine (mg) | 0.9 | 0.5 | 0.6 | 0.8 | 1.2 |  | 0.9 | 0.4 | 0.7 | 0.9 | 1.2 |
| Niacin (mg) | 11.3 | 6.8 | 6.7 | 10.3 | 14.8 |  | 11.3 | 5.1 | 8.0 | 10.6 | 14.4 |
| Vitamin C (mg) | 82.7 | 80.2 | 20.7 | 59.2 | 116.2 |  | 83.9 | 69.1 | 32.3 | 64.5 | 116.5 |
| Calcium (mg) | 744.3 | 412.1 | 422.8 | 682.7 | 993.4 |  | 745.3 | 375.5 | 441.3 | 685 | 990.6 |
| Magnesium (mg) | 214.7 | 77.8 | 159.8 | 208.9 | 251.2 |  | 215 | 66.1 | 168.1 | 209.1 | 248.5 |
| Iron (mg) | 8.3 | 3.8 | 5.7 | 7.7 | 10.5 |  | 8.3 | 3.3 | 6.1 | 7.8 | 10.1 |
| Phosphorus (mg) | 1021.4 | 348.5 | 774.3 | 992.4 | 1222 |  | 1022.2 | 300.1 | 816.7 | 1001.3 | 1202.9 |
| Sodium (mg) | 2166 | 893.2 | 1521 | 2046.3 | 2695.6 |  | 2168.9 | 734.7 | 1658.3 | 2086.7 | 2614.9 |
| Potassium (mg) | 1969.5 | 660.0 | 1539.1 | 1886.4 | 2378.9 |  | 1971.7 | 538 | 1599.7 | 1927.2 | 2320.7 |
| Copper (mg) | 0.9 | 0.5 | 0.6 | 0.8 | 1.1 |  | 0.9 | 0.4 | 0.6 | 0.8 | 1.1 |
| Zinc (mg) | 8.6 | 4.1 | 5.8 | 7.9 | 10.3 |  | 8.6 | 3.4 | 6.3 | 8.2 | 10.2 |
| Note: values estimated using the Multiple Source Method® program. SD = standard deviation. P = percentil. | | | | | | | | | | | |

| **Table 3.** Variances of within-person and between-person variability and the number of days of 24-hour dietary recall needed to estimate usual energy, macronutrient, and micronutrient intake in children and adolescents with autism spectrum disorder. Pelotas, Brazil, PANA Study (n=284) | | | | | | |
| --- | --- | --- | --- | --- | --- | --- |
| **Variables** | **Dietary variability** | |  | **Number of days needed** | | |
|  | **S^2^_w_** | **S^2^_b_** |  | **r 0.7** | **r 0.8** | **r 0.9** |
| Energy (Kcal) | 11.522 | 10.386 |  | 1 | 2 | 5 |
| Carbohydrates (g) | 3.727 | 3.538 |  | 1 | 2 | 4 |
| Proteins (g) | 0.891 | 0.674 |  | 1 | 2 | 6 |
| Lipids (g) | 0.137 | 0.095 |  | 1 | 3 | 6 |
| Saturated fatty acids (g) | 0.114 | 0.073 |  | 1 | 3 | 7 |
| Monosaturated fatty acids (g) | 0.211 | 0.133 |  | 2 | 3 | 7 |
| Polyunsaturated fatty acid (g) | 0.250 | 0.193 |  | 1 | 2 | 5 |
| Cholesterol (mg) | 0.289 | 0.176 |  | 2 | 3 | 7 |
| Fiber (g) | 0.409 | 0.432 |  | 1 | 2 | 4 |
| Retinol (mcg) | 0.238 | 0.273 |  | 1 | 2 | 4 |
| Thiamine (mg) | 0.163 | 0.113 |  | 1 | 3 | 6 |
| Riboflavin (mg) | 0.155 | 0.197 |  | 1 | 1 | 3 |
| Pyridoxine (mg) | 0.164 | 0.116 |  | 1 | 3 | 6 |
| Niacin (mg) | 0.209 | 0.146 |  | 1 | 3 | 6 |
| Vitamin C (mg) | 4.498 | 4.157 |  | 1 | 2 | 5 |
| Calcium (mg) | 10.684 | 20.669 |  | 1 | 1 | 2 |
| Magnesium (mg) | 1.226 | 1.155 |  | 1 | 2 | 5 |
| Iron (mg) | 0.606 | 0.661 |  | 1 | 2 | 4 |
| Phosphorus (mg) | 81.986 | 87.292 |  | 1 | 2 | 4 |
| Sodium (mg) | 1.057 | 0.872 |  | 1 | 2 | 5 |
| Potassium (mg) | 3.361 | 2.499 |  | 1 | 2 | 6 |
| Copper (mg) | 0.144 | 0.162 |  | 1 | 2 | 4 |
| Zinc (mg) | 0.136 | 0.117 |  | 1 | 2 | 5 |
| Note: variability values were estimated using the Multiple Source Method® program. Results for the number of days were obtained using the formula proposed by Black *et al*.^(29)^, considering the correlation coefficients of 0.7, 0.8, and 0.9. r = correlation coefficient. S^2^_w_ = within-person variance. S^2^_b_ = between-person variance. VR= S²_w_/S²_b_. | | | | | | |

| **Table 4.** Variances of intra- and inter-individual variability, variance ratios, and number of days needed for 24-hour recall to estimate usual energy, macronutrient, and micronutrient intake according to sex of children and adolescents with autism spectrum disorder. Pelotas, Brazil, PANA Study (n=284). | | | | | | | | | | | | | |
| --- | --- | --- | --- | --- | --- | --- | --- | --- | --- | --- | --- | --- | --- |
| **Variables** | **Boys (n=235)** | | | | | |  | **Girls (n=49)** | | | | | |
|  | **Dietary variability** | | | **Number of days needed** | | |  | **Dietary variability** | | | **Number of days needed** | | |
|  | **S²_w_** | **S²_b_** | **VR** | **r 0.7** | **r 0.8** | **r 0.9** |  | **S²_w_** | **S²_b_** | **VR** | **r 0.7** | **r 0.8** | **r 0.9** |
| Energy (Kcal) | 1.113 | 0.857 | 1.30 | 1 | 2 | 6 |  | 0.068 | 0.080 | 0.84 | 1 | 1 | 4 |
| Carbohydrates (g) | 1.194 | 1.035 | 1.15 | 1 | 2 | 5 |  | 0.508 | 0.493 | 1.03 | 1 | 2 | 4 |
| Proteins (g) | 0.880 | 0.609 | 1.44 | 1 | 3 | 6 |  | 0.307 | 0.224 | 1.37 | 1 | 2 | 6 |
| Lipids (g) | 0.426 | 0.259 | 1.65 | 2 | 3 | 7 |  | 0.147 | 0.139 | 1.06 | 1 | 2 | 5 |
| Saturated fatty acids (g) | 0.355 | 0.191 | 1.86 | 2 | 3 | 8 |  | 0.085 | 0.092 | 0.92 | 1 | 2 | 4 |
| Monosaturated fatty acids (g) | 0.217 | 0.127 | 1.72 | 2 | 3 | 7 |  | 0.199 | 0.149 | 1.34 | 1 | 2 | 6 |
| Polyunsaturated fatty acid (g) | 0.230 | 0.164 | 1.40 | 1 | 2 | 6 |  | 0.433 | 0.342 | 1.26 | 1 | 2 | 5 |
| Cholesterol (mg) | 0.266 | 0.167 | 1.60 | 2 | 3 | 7 |  | 0.446 | 0.216 | 2.07 | 2 | 4 | 9 |
| Fiber (g) | 0.492 | 0.444 | 1.11 | 1 | 2 | 5 |  | 0.187 | 0.260 | 0.72 | 1 | 1 | 3 |
| Retinol (mcg) | 0.227 | 0.258 | 0.88 | 1 | 2 | 4 |  | 0.270 | 0.340 | 0.79 | 1 | 1 | 3 |
| Thiamine (mg) | 0.151 | 0.089 | 1.70 | 2 | 3 | 7 |  | 0.160 | 0.170 | 0.94 | 1 | 2 | 4 |
| Riboflavin (mg) | 0.177 | 0.199 | 0.89 | 1 | 2 | 4 |  | 0.146 | 0.296 | 0.49 | 1 | 1 | 2 |
| Pyridoxine (mg) | 0.167 | 0.106 | 1.58 | 2 | 3 | 7 |  | 0.125 | 0.109 | 1.15 | 1 | 2 | 5 |
| Niacin (mg) | 0.208 | 0.150 | 1.39 | 1 | 2 | 6 |  | 0.684 | 0.302 | 2.27 | 2 | 4 | 10 |
| Vitamin C (mg) | 4.702 | 4.200 | 1.12 | 1 | 2 | 5 |  | 5.523 | 6.003 | 0.92 | 1 | 2 | 4 |
| Calcium (mg) | 10.979 | 19.060 | 0.58 | 1 | 1 | 2 |  | 10.471 | 29.812 | 0.35 | 1 | 1 | 1 |
| Magnesium (mg) | 0.434 | 0.333 | 1.30 | 1 | 2 | 6 |  | 0.347 | 0.476 | 0.73 | 1 | 1 | 3 |
| Iron (mg) | 0.603 | 0.604 | 1.00 | 1 | 2 | 4 |  | 0.364 | 0.387 | 0.94 | 1 | 2 | 4 |
| Phosphorus (mg) | 95.983 | 77.812 | 1.23 | 1 | 2 | 5 |  | 73.970 | 164.068 | 0.45 | 1 | 1 | 2 |
| Sodium (mg) | 0.512 | 0.350 | 1.46 | 1 | 3 | 6 |  | 7.089 | 7.717 | 0.92 | 1 | 2 | 4 |
| Potassium (mg) | 0.593 | 0.347 | 1.71 | 2 | 3 | 7 |  | 11.597 | 17.403 | 0.67 | 1 | 1 | 3 |
| Copper (mg) | 0.150 | 0.152 | 0.98 | 1 | 2 | 4 |  | 0.148 | 0.197 | 0.75 | 1 | 1 | 3 |
| Zinc (mg) | 0.132 | 0.106 | 1.25 | 1 | 2 | 5 |  | 0.179 | 0.139 | 1.29 | 1 | 2 | 5 |
| Note: Variability values were estimated using the Multiple Source Method® program. Results for the number of days were obtained using the formula proposed by Black *et al*.^(29)^, considering the correlation coefficients of 0.7, 0.8, and 0.9. r= correlation coefficient. S^2^_w_= Within-person variance. S^2^_b_ = Between-person variance. VR= S²_w_/S²_b_. | | | | | | | | | | | | | |

| **Table 5.** Variances of within-person and between-person variability of energy, macronutrient, and micronutrient according to age group of children and adolescents with autism spectrum disorder. Pelotas, Brazil, PANA Study (n=284). | | | | | | | | | | | |
| --- | --- | --- | --- | --- | --- | --- | --- | --- | --- | --- | --- |
| **Variaveis** | **2-5 years (n= 118)** | | |  | **6-9 years (n= 97)** | | |  | **10-18 years (n=69)** | | |
|  | **S²_w_** | **S²_b_** | **VR** |  | **S²_w_** | **S²_b_** | **VR** |  | **S²_w_** | **S²_b_** | **VR** |
| Energy (Kcal) | 9.814 | 12.216 | 0.80 |  | 0.402 | 0.249 | 1.62 |  | 1.002 | 0.492 | 2.04 |
| Carbohydrates (g) | 3.461 | 4.262 | 0.81 |  | 0.218 | 0.177 | 1.23 |  | 0.261 | 0.092 | 2.85 |
| Proteins (g) | 0.999 | 0.764 | 1.31 |  | 6.769 | 5.018 | 1.35 |  | 2.088 | 1.304 | 1.60 |
| Lipids (g) | 1.249 | 1.268 | 0.99 |  | 0.183 | 0.071 | 2.57 |  | 0.126 | 0.083 | 1.52 |
| Saturated fatty acids (g) | 0.978 | 1.130 | 0.87 |  | 0.135 | 0.037 | 3.61 |  | 0.171 | 0.103 | 1.66 |
| Monosaturated fatty acids (g) | 0.413 | 0.370 | 1.12 |  | 0.255 | 0.084 | 3.02 |  | 0.230 | 0.121 | 1.89 |
| Polyunsaturated fatty acid (g) | 0.233 | 0.215 | 1.08 |  | 0.319 | 0.255 | 1.25 |  | 1.135 | 0.307 | 3.69 |
| Cholesterol (mg) | 0.343 | 0.269 | 1.28 |  | 0.912 | 0.622 | 1.47 |  | 13.427 | 3.946 | 3.40 |
| Fiber (g) | 0.323 | 0.457 | 0.71 |  | 0.856 | 0.841 | 1.02 |  | 1.136 | 0.588 | 1.93 |
| Retinol (mcg) | 10.070 | 16.954 | 0.59 |  | 0.247 | 0.285 | 0.87 |  | 0.559 | 0.301 | 1.86 |
| Thiamine (mg) | 0.124 | 0.174 | 0.71 |  | 0.163 | 0.046 | 3.59 |  | 0.233 | 0.124 | 1.88 |
| Riboflavin (mg) | 0.130 | 0.250 | 0.52 |  | 0.226 | 0.198 | 1.14 |  | 0.270 | 0.232 | 1.17 |
| Pyridoxine (mg) | 0.173 | 0.190 | 0.91 |  | 0.090 | 0.052 | 1.72 |  | 0.205 | 0.093 | 2.21 |
| Niacin (mg) | 0.563 | 0.419 | 1.34 |  | 0.195 | 0.095 | 2.05 |  | 0.214 | 0.108 | 1.99 |
| Vitamin C (mg) | 4.525 | 3.665 | 1.23 |  | 6.645 | 7.902 | 0.84 |  | 15.733 | 10.085 | 1.56 |
| Calcium (mg) | 100.426 | 261.309 | 0.38 |  | 75.560 | 93.247 | 0.82 |  | 1.518 | 1.627 | 0.93 |
| Magnesium (mg) | 0.655 | 0.904 | 0.73 |  | 0.391 | 0.298 | 1.31 |  | 27.524 | 20.407 | 1.35 |
| Iron (mg) | 0.335 | 0.419 | 0.80 |  | 0.171 | 0.176 | 0.97 |  | 1.498 | 0.641 | 2.34 |
| Phosphorus (mg) | 74.330 | 113.463 | 0.66 |  | 64.155 | 47.197 | 1.36 |  | 96.188 | 83.645 | 1.15 |
| Sodium (mg) | 0.374 | 0.337 | 1.11 |  | 8.426 | 4.095 | 2.06 |  | 7.240 | 3.802 | 1.90 |
| Potassium (mg) | 13.633 | 16.017 | 0.85 |  | 0.360 | 0.157 | 2.30 |  | 17.719 | 12.545 | 1.41 |
| Copper (mg) | 0.144 | 0.193 | 0.75 |  | 0.146 | 0.124 | 1.18 |  | 0.148 | 0.108 | 1.37 |
| Zinc (mg) | 0.156 | 0.138 | 1.13 |  | 0.103 | 0.094 | 1.09 |  | 0.151 | 0.102 | 1.48 |
| Note: Variability values were estimated using the Multiple Source Method® program. S^2^_w_= Within-person variance. S^2^_b_ = Between-person variance. VR= S²_w_/S²_b_. | | | | | | | | | | | |

| **Table 6.** Number of days of 24-hour dietary recall required to estimate the usual energy, macronutrient, and micronutrient intake according to age group of children and adolescents with autism spectrum disorder. Pelotas, Brazil, PANA Study (n=284). | | | | | | | | | | | |
| --- | --- | --- | --- | --- | --- | --- | --- | --- | --- | --- | --- |
| **Variables** | **2-5 years (n= 118)** | | |  | **6-9 years (n= 97)** | | |  | **10-18 years (n=69)** | | |
|  | **r 0.7** | **r 0.8** | **r 0.9** |  | **r 0.7** | **r 0.8** | **r 0.9** |  | **r 0.7** | **r 0.8** | **r 0.9** |
| Energy (Kcal) | 1 | 1 | 3 |  | 2 | 3 | 7 |  | 2 | 4 | 9 |
| Carbohydrates (g) | 1 | 1 | 3 |  | 1 | 2 | 5 |  | 3 | 5 | 12 |
| Proteins (g) | 1 | 2 | 6 |  | 1 | 2 | 6 |  | 2 | 3 | 7 |
| Lipids (g) | 1 | 2 | 4 |  | 2 | 5 | 11 |  | 1 | 3 | 6 |
| Saturated fatty acids (g) | 1 | 2 | 4 |  | 3 | 6 | 15 |  | 2 | 3 | 7 |
| Monosaturated fatty acids (g) | 1 | 2 | 5 |  | 3 | 5 | 13 |  | 2 | 3 | 8 |
| Polyunsaturated fatty acid (g) | 1 | 2 | 5 |  | 1 | 2 | 5 |  | 4 | 7 | 16 |
| Cholesterol (mg) | 1 | 2 | 5 |  | 1 | 3 | 6 |  | 3 | 6 | 14 |
| Fiber (g) | 1 | 1 | 3 |  | 1 | 2 | 4 |  | 2 | 3 | 8 |
| Retinol (mcg) | 1 | 1 | 3 |  | 1 | 2 | 4 |  | 2 | 3 | 8 |
| Thiamine (mg) | 1 | 1 | 3 |  | 3 | 6 | 15 |  | 2 | 3 | 8 |
| Riboflavin (mg) | 1 | 1 | 2 |  | 1 | 2 | 5 |  | 1 | 2 | 5 |
| Pyridoxine (mg) | 1 | 2 | 4 |  | 2 | 3 | 7 |  | 2 | 4 | 9 |
| Niacin (mg) | 1 | 2 | 6 |  | 2 | 4 | 9 |  | 2 | 4 | 8 |
| Vitamin C (mg) | 1 | 2 | 5 |  | 1 | 1 | 4 |  | 1 | 3 | 7 |
| Calcium (mg) | 1 | 1 | 2 |  | 1 | 1 | 3 |  | 1 | 2 | 4 |
| Magnesium (mg) | 1 | 1 | 3 |  | 1 | 2 | 6 |  | 1 | 2 | 6 |
| Iron (mg) | 1 | 1 | 3 |  | 1 | 2 | 4 |  | 2 | 4 | 10 |
| Phosphorus (mg) | 1 | 1 | 3 |  | 1 | 2 | 6 |  | 1 | 2 | 5 |
| Sodium (mg) | 1 | 2 | 5 |  | 2 | 4 | 9 |  | 2 | 3 | 8 |
| Potassium (mg) | 1 | 2 | 4 |  | 2 | 4 | 10 |  | 1 | 3 | 6 |
| Copper (mg) | 1 | 1 | 3 |  | 1 | 2 | 5 |  | 1 | 2 | 6 |
| Zinc (mg) | 1 | 2 | 5 |  | 1 | 2 | 5 |  | 1 | 3 | 6 |
| Note: Results for the number of days obtained using the formula proposed by Black *et al*.^(29)^, considering the correlation coefficients of 0.7, 0.8, and 0.9. r= correlation coefficient. | | | | | | | | | | | |
