# Supplementary material for: Dietary fibre intake in the adult Swiss population: a comprehensive analysis of timing and sources
Source: J Nutr Sci. 2025 Mar 24;14:e27. doi: 10.1017/jns.2025.6 (PMC11950702; doi:10.1017/jns.2025.6)
Supplement: von Blumenthal et al. supplementary material 1 — von Blumenthal et al. supplementary material [file S2048679025000060sup001.zip › BJN-2500020.zip!/bjn_2500020_ES/bjn_2500020_ES/bjn-2024-0762-20250206173532.zip!/bjn-2024-0762-20250206173532/graphic/Figure 1.pdf]

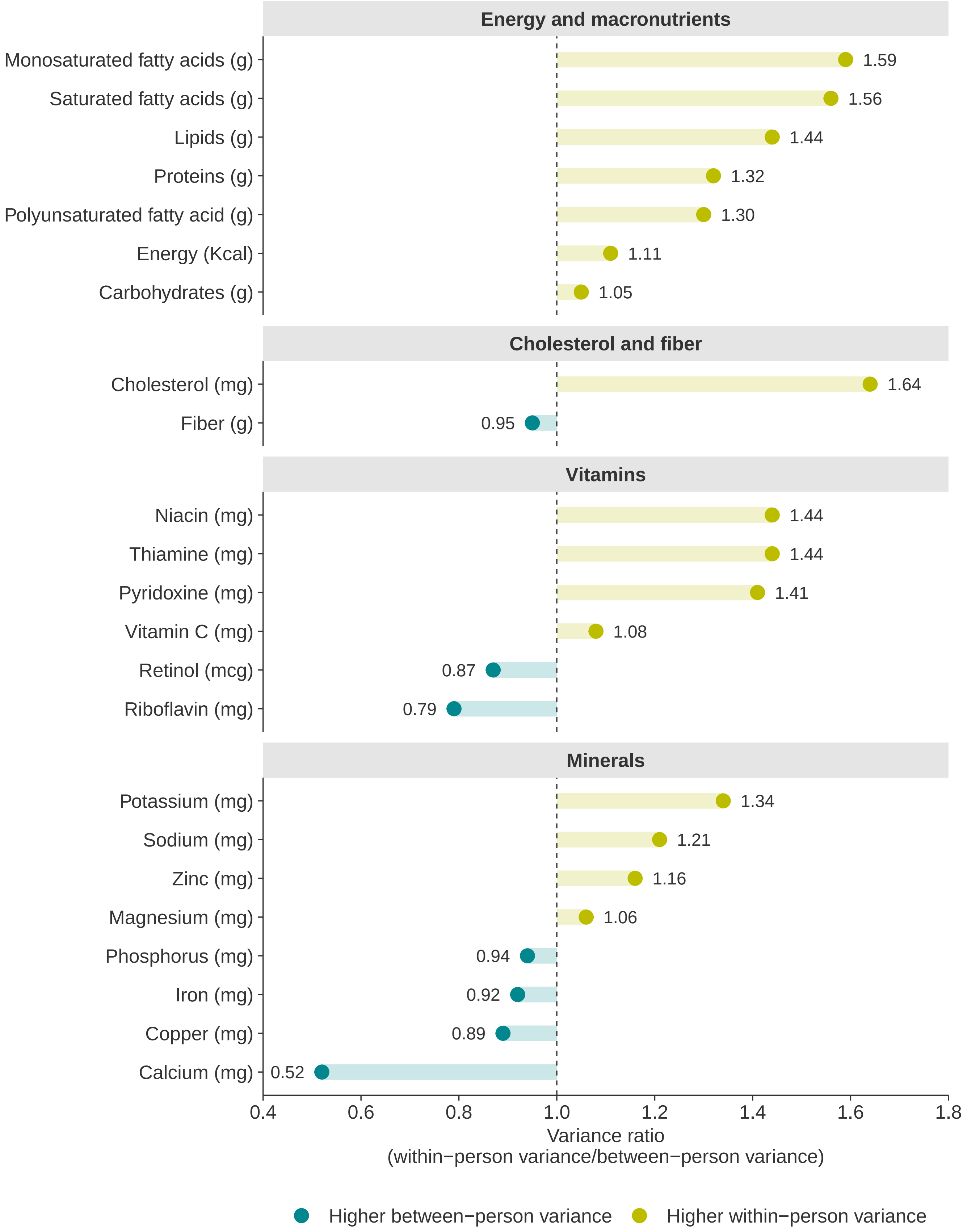

Energy and macronutrients
Monosaturated fatty acids (g)
1.59
Saturated fatty acids (g)
1.56
Lipids (g)
1.44
Proteins (g)
1.32
Polyunsaturated fatty acid (g)
1.30
Energy (Kcal)
1.11
Carbohydrates (g)
1.05
Cholesterol and fiber
Cholesterol (mg)
1.64
Fiber (g)
0.95
Vitamins
Niacin (mg)
1.44
Thiamine (mg)
1.44
Pyridoxine (mg)
1.41
Vitamin C (mg)
1.08
Retinol (mcg)
0.87
Riboflavin (mg)
0.79
Minerals
Potassium (mg)
1.34
Sodium (mg)
1.21
Zinc (mg)
1.16
Magnesium (mg)
1.06
Phosphorus (mg)
0.94
Iron (mg)
0.92
Copper (mg)
0.89
Calcium (mg)
0.52
0.4
0.6
0.8
1.0
1.2
1.4
1.6
1.8
Variance ratio
(within−person variance/between−person variance)
Higher between−person variance
Higher within−person variance
