# Supplementary material for: Dietary fibre intake in the adult Swiss population: a comprehensive analysis of timing and sources
Source: J Nutr Sci. 2025 Mar 24;14:e27. doi: 10.1017/jns.2025.6 (PMC11950702; doi:10.1017/jns.2025.6)
Supplement: von Blumenthal et al. supplementary material 1 — von Blumenthal et al. supplementary material [file S2048679025000060sup001.zip › BJN-2500020.zip!/bjn_2500020_ES/bjn_2500020_ES/bjn-2024-0762-20250206173532.zip!/bjn-2024-0762-20250206173532/suppl_data/Supplementary material 1-2.pdf]

British Journal of Nutrition

Nutrient intake variability and number of days needed to estimate usual intake in children and adolescents with autism spectrum disorder

Nutrient variability in patients with autism

Supplementary material 1-2

Eduarda Silva[1]; Laura Hoffmann[1]; Kamila Castro[1,2]; Eliseu Verly Junior[3]; Sandra Valle[1,4]; Juliana Vaz[1,4]

[1]Programa de Pós-Graduação em Nutrição e Alimentos, Universidade Federal de Pelotas – Pelotas (RS), Brasil.
[2]Serviço de Neuropediatria do Hospital de Clínicas de Porto Alegre - Porto Alegre (RS), Brasil.
[3]Departamento de Epidemiologia, Instituto de Medicina Social, Universidade do Estado do Rio de Janeiro - Rio de Janeiro (RJ), Brasil.
[4]Faculdade de Nutrição, Universidade Federal de Pelotas - Pelotas (RS), Brasil.

Supplementary material 1. Distribution of usual energy, macronutrient, and micronutrient intake according to sex of children and adolescents with autism spectrum disorder. Pelotas, Brazil, PANA Study (n=284).

**Variables**	**Boys (n=235)**					**Girls (n=49)**				
	**Mean**	**SD**	**P25**	**P50**	**P75**	**Mean**	**SD**	**P25**	**P50**	**P75**
Energy (Kcal)	1841.7	460.4	1497.4	1815.3	2149.8	1500.8	475.5	1134.6	1395.5	1736.5
Carbohydrates (g)	257.5	72.7	205.0	249.7	309.2	206.0	64.5	156.9	196.1	257.1
Proteins (g)	61.4	17.4	49.5	59.7	70.7	49.3	16.0	37.2	47.8	57.2
Lipids (g)	63.1	20.3	48.2	60.3	73.6	53.4	23.3	37.0	49.2	59.5
Saturated fatty acids (g)	22.1	6.8	17.0	21.1	26.4	18.8	8.1	12.7	18.3	23.5
Monosaturated fatty acids (g)	19.4	7.0	14.1	18.4	23.8	16.2	7.1	11.3	14.2	18.7
Polyunsaturated fatty acid (g)	13.9	6.4	9.5	12.6	17.0	11.6	7.5	7.0	9.0	12.9
Cholesterol (mg)	197	97.9	125.5	182.7	257.1	173.9	91.3	110.3	149.2	220.5
Fiber (g)	17.3	7.6	15.9	21.4	27.3	12.9	8.1	7.5	10.5	18.2
Retinol (mcg)	311.8	207	165.1	282.4	415.9	273.8	156.2	133.3	264.8	372.3
Thiamine (mg)	1.2	0.4	0.9	1.1	1.4	1.0	0.4	0.7	0.9	1.2
Riboflavin (mg)	1.6	0.7	1.2	1.5	2.0	1.3	0.7	0.8	1.3	1.7
Pyridoxine (mg)	1.0	0.4	0.7	0.9	1.2	0.7	0.3	0.5	0.7	0.9
Niacin (mg)	11.8	5.4	8.2	11.2	14.9	9.0	3.3	7.0	8.6	10.3
Vitamin C (mg)	86.9	71.6	33.9	66	118.8	69.0	53.5	25.3	58.7	98.8
Calcium (mg)	760.8	364.1	483.4	699.1	1004.7	672.3	422.1	347.1	587.2	868.2
Magnesium (mg)	223.4	63.0	178.6	218.2	252.1	174.7	64.4	135.5	160.1	209.4
Manganese (mg)	1.0	0.4	0.7	1.0	1.3	0.8	0.4	0.6	0.7	0.9
Iron (mg)	8.7	3.3	6.4	8.2	10.7	6.4	2.5	4.4	5.9	7.8
Phosphorus (mg)	1052.2	273.7	858.3	1026.3	1232.6	878.7	370.8	590.6	902.2	1081.8
Sodium (mg)	2265.5	699.2	1736.5	2193.3	2722.1	1710.1	717.8	1153.7	1652.3	2021.2
Potassium (mg)	2021.2	499.9	1681.5	1955.8	2342.5	1736.7	636.8	1335.2	1665.4	2084.6
Copper (mg)	0.9	0.4	0.7	0.9	1.1	0.7	0.4	0.5	0.7	0.9
Zinc (mg)	9.0	3.4	6.6	8.5	10.5	6.9	2.7	4.9	6.4	8.4

Note: Values estimated using the Multiple Source Method® program. Abbreviations: SD = standard deviation; P = percentil.

Supplementary material 2. Distribution of usual energy, macronutrient, and micronutrient intake according to age group of children and adolescents with autism spectrum disorder. Pelotas, Brazil, PANA Study (n=284).

**Variables**	**2-5 years (n=118)**					**6-9 years (n=97)**					**10-18 years (n=69)**				
	**Mean**	**SD**	**P25**	**P50**	**P75**	**Mean**	**SD**	**P25**	**P50**	**P75**	**Mean**	**SD**	**P25**	**P50**	**P75**
Energy (Kcal)	1602.4	482.6	1281.5	1517.1	1908.9	1830.7	432.5	1524.5	1803	2049.3	2024.6	413.4	1767.2	2021.2	2306.9
Carbohydrates (g)	218.2	71.4	169.2	205.9	255.8	257	71.9	207.1	254.5	291.2	289	55.3	247.5	286.7	324.7
Proteins (g)	55.2	16.7	42.7	53.5	65.3	57.9	16.2	48	57	66.5	68.2	19.3	55.1	66.8	78.6
Lipids (g)	56.7	21.8	39.8	52.9	71.4	63.7	17.8	50.6	59.6	73.2	66.3	23	49.8	61.5	79
Saturated fatty acids (g)	21.5	8.1	15.6	20.5	26.9	21.4	5	18	20.7	24.5	21.9	8.2	16.2	20.5	26.2
Monosaturated fatty acids (g)	16.7	6.9	11.7	15	21.3	19.8	5.8	14.9	19	23.9	21.1	7.8	15.9	19.3	25.8
Polyunsaturated fatty acid (g)	11.3	6.2	7.3	10.1	13.7	14.8	7.8	9.6	12.2	18.9	15.5	4.3	12.4	15.4	17.8
Cholesterol (mg)	178.9	101.1	113.2	162.1	209	199.5	104.6	123.7	190.8	257.4	209.2	76.4	159.1	206.9	259
Fiber (g)	14.6	7.8	9	12.7	19.2	16.6	7.3	11.1	15.8	19.7	19.8	7.4	14.6	18.9	21.8
Retinol (mcg)	337.9	203.7	189.2	311.6	463.4	293	207.6	167.4	260	395.8	266.1	159	143.9	234.3	385.7
Thiamine (mg)	1.1	0.5	0.8	1	1.4	1.1	0.3	0.9	1.1	1.3	1.2	0.4	0.9	1.2	1.4
Riboflavin (mg)	1.7	0.8	1.1	1.7	2.2	1.5	0.6	1.1	1.4	1.7	1.3	0.5	0.9	1.3	1.6
Pyridoxine (mg)	0.9	0.4	0.6	0.8	1.1	1	0.4	0.7	0.9	1.1	1	0.3	0.8	1	1.2
Niacin (mg)	9.4	4.5	5.7	8.9	12.4	11.5	4.3	8.4	10.6	14.2	14.4	5.7	10.7	13.7	17.1
Vitamin C (mg)	64.5	54	27.9	45.9	90.3	87.1	75.8	31.3	66.5	127.7	111.1	68.1	65.4	97.5	154.9
Calcium (mg)	901.4	438.4	603.6	863.8	1163.2	677.3	275.1	478.2	665.7	845.3	573.7	271.8	370.9	538.8	688
Magnesium (mg)	205.5	66.7	158.9	195.9	239	213.3	62.1	175	203.1	243.8	233.3	68.1	185.3	226	257.4
Manganese (mg)	0.8	0.4	0.5	0.7	1	1	0.4	0.7	1	1.2	1.2	0.4	0.9	1.2	1.5
Iron (mg)	7.3	3.3	4.9	6.6	9.1	8.5	3.3	6.2	7.9	10.7	9.7	2.3	8.2	9.5	11.2
Phosphorus (mg)	1063	341.6	806.7	1023.7	1300	979.6	249.1	837.9	973.6	1120.6	1012.1	288	812.8	994.6	1210.2
Sodium (mg)	1805.6	625.3	1368.7	1764.8	2277.1	2233.9	606.2	1789.1	2178.8	2646.2	2702.1	725.1	2089	2633.3	3243
Potassium (mg)	1962.4	596.7	1572.6	1867.4	2328.2	1930.8	437.8	1678.9	1857	2189.8	2045.2	568.9	1585.3	2073.9	2435.9
Copper (mg)	0.8	0.4	0.5	0.7	1	0.9	0.4	0.7	0.9	1.2	1.1	0.4	0.9	1	1.2
Zinc (mg)	7.9	3.1	5.7	7.7	9.3	8.7	3.5	6.3	8.5	10.1	9.8	3.5	7.5	9.2	11.1

Note: Values estimated using the Multiple Source Method® program. Abbreviations: SD = standard deviation; P = percentil.
