# Supplementary material for: Dietary fibre intake in the adult Swiss population: a comprehensive analysis of timing and sources
Source: J Nutr Sci. 2025 Mar 24;14:e27. doi: 10.1017/jns.2025.6 (PMC11950702; doi:10.1017/jns.2025.6)
Supplement: von Blumenthal et al. supplementary material 1 — von Blumenthal et al. supplementary material [file S2048679025000060sup001.zip › BJN-2500020.zip!/bjn_2500020_ES/bjn_2500020_ES/bjn-2024-0762-20250206173532.zip!/bjn-2024-0762-20250206173532/bjn-2024-0762-manifest.html]

Manifest file for export bjn-2024-0762-20250206173532


|  |  |
| --- | --- |
| File name: bjn-2024-0762-20250206173532 | |
| Export Date: 06-Feb-2025 | |
| Output Format: XML (ScholarOne DTD) | |
| bjn-2024-0762-20250206173532/doc/Manuscript (2) 23.1.25.docx | Version 1.0 |
| bjn-2024-0762-20250206173532/graphic/Figure 1.pdf | Version 1.0 |
| bjn-2024-0762-20250206173532/suppl\_data/Supplementary material 1-2.pdf | Version 1.0 |
| bjn-2024-0762-20250206173532/suppl\_data/Supplementary material 3-4.pdf | Version 1.0 |
| bjn-2024-0762-20250206173532/bjn-2024-0762-metadata.xml |  |
| bjn-2024-0762-20250206173532/s1.dtd |  |
| manifest.html | This document |
